# Bicyclopentylation of Alcohols with Thianthrenium Reagents

**DOI:** 10.1021/jacs.3c10024

**Published:** 2023-11-27

**Authors:** Zibo Bai, Beatrice Lansbergen, Tobias Ritter

**Affiliations:** Max-Planck-Institut für Kohlenforschung, Kaiser-Wilhelm-Platz 1, D-45470 Mülheim an der Ruhr, Germany

## Abstract

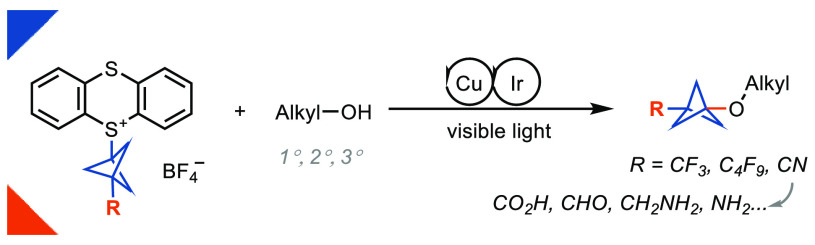

Herein we present the first method for the synthesis of bicyclo[1.1.1]pentyl (BCP) alkyl ethers from alcohols. The reaction uses BCP–thianthrenium reagents and is catalyzed by a dual copper/photoredox catalyst system. Unlike known alkylations of tertiary alcohols via carbocation intermediates, our Cu-mediated radical process circumvents the labile BCP carbocations. The approach demonstrates a broad tolerance for functional groups when applied to primary, secondary, and even tertiary alcohols. In addition, we highlight the utility of this method in late-stage functionalizations of both natural products and pharmaceuticals as well as in the rapid construction of BCP analogs of known pharmaceuticals that would otherwise be difficult to access.

Approximately 45% of marketed small-molecule pharmaceuticals contain phenyl rings.^1^ In modern medicinal chemistry, replacement of planar phenyl rings with sp^3^-rich bioisosteres can lead to increased metabolic stability, membrane permeability, and increased solubility.^2^ Since the first study of the BCP analogue of (*S*)-(4-carboxyphenyl)glycine was described by Pellicciari and co-workers in 1996,^3^ the rigid three-dimensional 1,3-disubstituted BCPs have emerged as promising bioisosteres for para-substituted benzenes in drug development that maintain the exit vectors in a 180° dihedral angle.^4^ Over the last 15 years, numerous efforts have been devoted to the development of substituted BCPs, such as BCP amines,^5^ BCP arenes,^5h,6^ BCP alkanes,^6f,7^ and BCP aryl ethers^5h^ via the functionalization of [1.1.1]propellane^8^ or cross-coupling reactions of BCP-based reagents.^5h,6c,6d,7b,9^*However, alkylation of alcohols with BCP scaffolds has remained elusive.* Such a new method would enable access to BCP analogs of alkyl aryl ethers that are prevalent in pharmaceuticals, such as in the selective serotonin reuptake inhibitor fluoxetine and the Parkinson’s medication safinamide. Here we report the first bicyclopentylation of alcohols using BCP–thianthrenium (TT^+^) reagents. Distinct from well-established alkylation of alcohols with tertiary electrophiles such as *tert*-butyl bromide via carbocation intermediates,^10^ our method proceeds through a metal-mediated radical process that can bypass the unstable BCP cations. The favorable high reduction potential and rapid mesolytic cleavage rate of BCP–TT^+^ allow the reaction to proceed under mild conditions with a wide variety of functional groups present and alcohols used as limiting reagents. Mechanistic studies imply that TT may potentially act as an electron-transfer mediator between the photoredox catalyst and copper catalyst. The substituted BCP–TT^+^ reagents can be used as structural linkers or end groups, which allows for rapid preparation of promising BCP analogs of pharmaceutically relevant ethers. To the best of our knowledge, this study represents the first example of directing-group-free transition-metal (TM)-catalyzed alkylation of tertiary alcohols that does not involve classic carbocation intermediates.

Alkyl aryl ethers are important structural motifs in pharmaceutically active compounds,^11^ such as in fluoxetine,^12^ butoxycaine,^13^ safinamide,^14^ and pranlukast.^15^ The current approach to BCP alkyl ethers involves the alkylation of a hydroxyl-substituted BCP with reactive alkylating reagents. Therefore, the approach is limited to reactive primary electrophiles, such as benzyl bromide, in the presence of a strong base for a conventional Williamson ether synthesis (Figure 1A, left).^6c^ Alternatively, a conceptually distinct approach is the bicyclopentylation of alcohols with BCP-based reagents, which remains challenging due to the sluggish development of syntheses of tertiary alkyl ethers (Figure 1A, right). The Baran^16a^ and Ohmiya^16b^ groups reported elegant syntheses of hindered dialkyl ethers from tertiary alkyl carboxylic acids and alcohols via electrochemistry and organophotoredox catalysis, respectively. Key to the success lies in the generation of tertiary carbocations or carbocation-like intermediates under nonacidic conditions, which can be subsequently trapped by alcohol nucleophiles. However, the BCP carboxylic acid, as a bridgehead 3° carbocation precursor, does not afford the corresponding product under these conditions (Figure 1B),^16a^ possibly because of the low stability of BCP cation intermediates (energy barrier to ring opening: ∼19 kcal/mol).^17^ The use of transition metals to catalyze the bicyclopentylation of alcohols involving kinetically stable BCP radical intermediates (energy barrier to ring opening: ∼26 kcal/mol),^18^ may avoid the skeletal rearrangement of the BCP intermediates. To date, BCP metals,^6b^ BCP halides,^6d^ BCP boronates,^6c,7b,9^ BCP amines,^5a−5d^ and BCP carboxylate derivatives^6a,6e^ are the most used reagents for the construction of BCPs. However, none of them have been reported for the functionalization of alcohols to construct ether bonds. Considering the potential of BCP alkyl ethers in drug discovery, we sought to develop the first TM-catalyzed bicyclopentylation of alcohols using BCP–TT^+^ reagents (Figure 1C).

**Figure 1:**
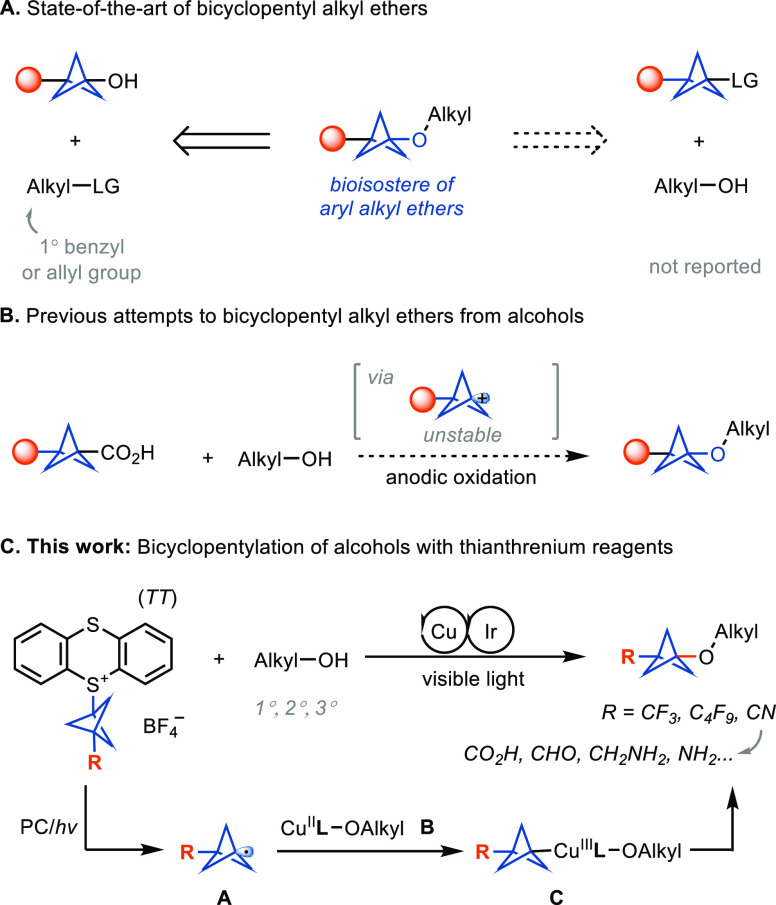
Synthesis of BCP alkyl ethers. LG, leaving group; PC, photocatalyst.

We have previously demonstrated that the cationic BCP–TT^+^ reagents can serve as BCP radical sources, which can engage in TM-mediated functionalization of phenols, various N-nucleophiles, and (het)aryl bromides.^5h^ However, these conditions failed to deliver the desired product when alcohols were used as substrates due to the inherent reactivity differences between alcohols and the other, softer nucleophiles, like phenols and amines. For instance, metal alkoxides, crucial intermediates in many ether syntheses, exhibit both higher basicity and stronger reducing power compared to metal phenoxides,^19,20^ and alcohols are less nucleophilic than many N-nucleophiles. Consequently, in many of the TM-catalyzed C–O cross-coupling reactions involving alcohols, excess or even solvent amounts of alcohol are required.^21^ As part of our ongoing endeavors on BCP and thianthrenium chemistry and encouraged by recent Cu-catalyzed C–O cross-coupling reactions of alcohols,^22,23^ we sought the functionalization of alcohols with BCP–TT^+^ salts. The reaction could proceed by the reductive generation of BCP radical **A** (Figure 1C). Subsequently, the BCP radical would be intercepted by metal alkoxide intermediate **B** to form Cu(III) intermediate **C**, which undergoes reductive elimination for C–O bond formation.

Based on our working hypothesis, we found that the reaction between 4-methoxyphenethyl alcohol (**1a**) and trifluoromethyl bicyclopentyl thianthrenium salt (CF_3_-BCP–TT^+^BF_4_^–^, **1b**) occurred with Cu(acac)_2_ as the catalyst, Na_2_CO_3_ as the base, and Ir[dF(CF_3_)ppy]_2_(dtbbpy)PF_6_ as the photocatalyst under 460 nm irradiation to give **1** in 85% yield (Figure 2A). The use of Cu(acac)_2_ was crucial for the high yield, possibly because the β-diketonate ligand may facilitate the oxidative ligation of BCP radical **A** or stabilize the high-valent metal alkoxides **C** (entries 1–4).^5d,23a,24,25^ While Cu(TMHD)_2_ (TMHD = 2,2,6,6-tetramethyl-3,5-heptanedionate) can afford higher yields, we only opted for this more expensive copper source when Cu(acac)_2_ afforded less than 60% yield (entry 5). Control experiments showed that the copper catalyst, iridium photocatalyst, 3 Å molecular sieves, and blue LED light irradiation are necessary for efficient reaction (entries 9–15).^26^ The addition of 2.0 equiv of the radical trapping reagent 2,2,6,6,-tetramethylpiperidin-1-oxyl (TEMPO) suppressed the formation of **1**, and a TEMPO–BCP radical adduct was observed (entry 16), consistent with the formation of BCP radicals.

**Figure 2:**
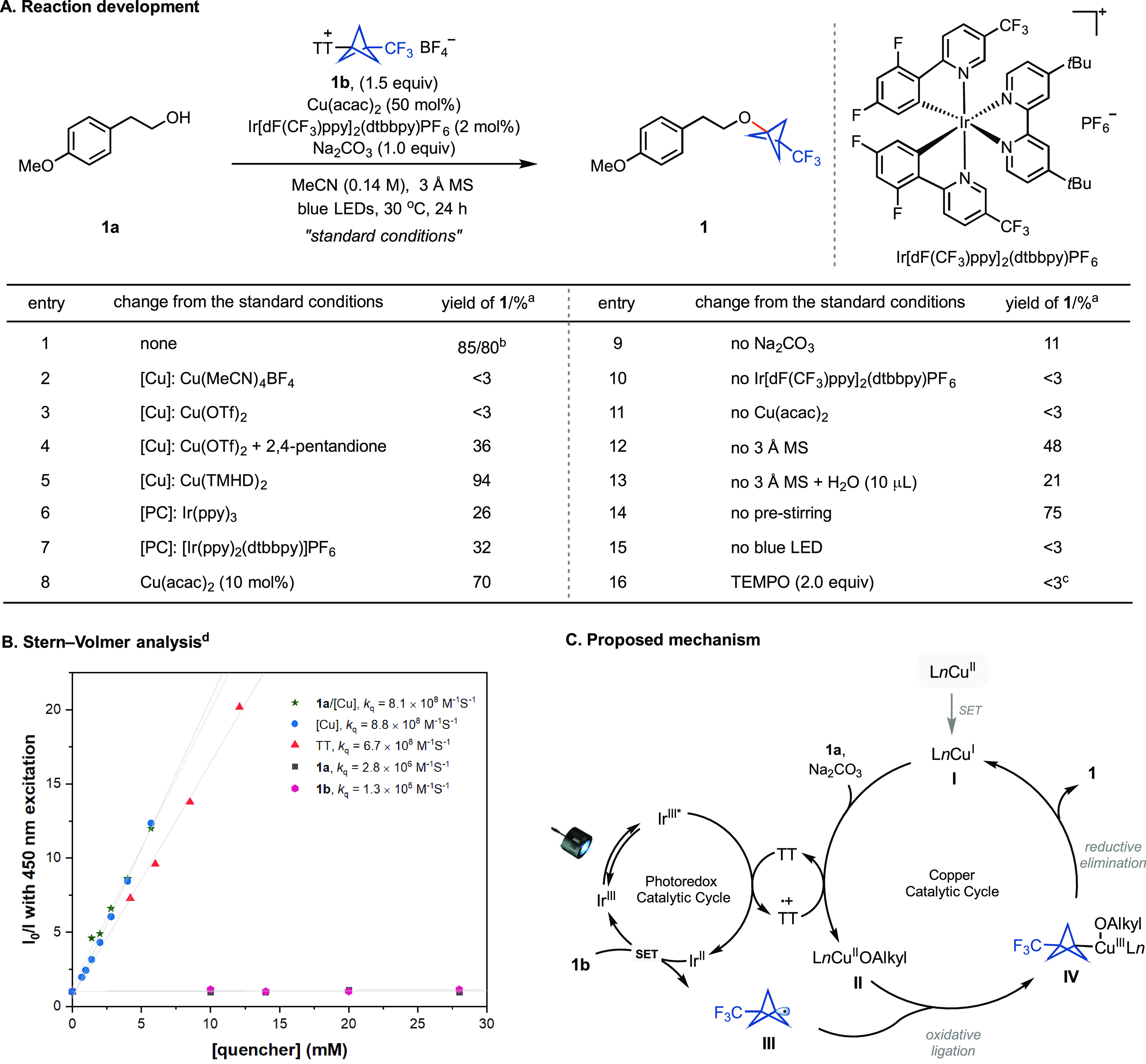
Reaction development and a mechanistic investigation. ^a^Yields were determined by ^19^F NMR. ^b^Isolated yield. ^c^The TEMPO–BCP adduct was observed by HRMS. ^d^The Stern–Volmer plot of Cu(acac)_2_ was corrected due to the inner filter effect.

A Stern–Volmer analysis revealed an effective quenching of the photoexcited sensitizer’s (Ir[dF(CF_3_)ppy]_2_(dtbbpy)PF_6_) luminescence by Cu(acac)_2_ and thianthrene (TT) (Figure 2B). We propose that Cu(I) may act as an active catalyst formed in situ by initial single electron transfer (SET) to the Cu(II) precatalyst from the excited photoredoxcatalyst (Ir^IV^/*Ir^III^ = −0.9 V, *E*_1/2_ = −1.0 V for Cu(acac)_2_, both versus SCE in MeCN).^5d,27,28^ After induction, the reaction may proceed by reductive quenching of the excited Ir(III) photoredoxcatalyst by TT (*Ir^III^/Ir^II^ = +1.21 V, *E*_1/2_ = +1.26 V for TT, both versus SCE in MeCN).^29,30^ Based on this analysis and previous literature,^5,28,31^ a plausible catalytic cycle is shown in Figure 2C. After quenching of the excited Ir(III) photoreodoxcatalyst, the ensuing Ir(II) species can reduce the BCP–TT^+^ salt (Ir^III^/Ir^II^ = −1.4 V, *E*_1/2_ = −1.4 V for **1b**, versus SCE in MeCN),^5h^ generating BCP radical **III**. The TT radical cation can oxidize the Cu(I) catalyst to generate Cu(II) (supported by EPR analysis; Figure S8), which then undergoes ligand exchange with the O-nucleophile. Oxidative ligation with intermediate **III** affords the Cu(III) complex **IV**. Finally, the desired product is formed via reductive elimination from **IV**. All experimental observables are consistent with the proposed mechanism.^32^

Due to the mild conditions, a large variety of alcohols can now be bicyclopentylated to afford BCP alkyl ethers that would otherwise be challenging to synthesize by other methods (Figure 3). For example, no synthetic procedure for secondary alkyl BCP ethers is currently documented. Overall, the reaction shows broad scope and proceeds efficiently with primary (for example, **5**, **8**, and **15**), cyclic (for example, **17**, **19**, and **20**) and acyclic (for example, **22** and **25**) secondary, and tertiary (**18**) alcohols. Various synthetically useful functional groups, such as esters (**2**, **17**, **20**, **22**), alkenes (**4**, **11**, **19**), alkyne (**6**), amides (**9**, **12**, **13**), halides (**8**, **15**, **25**), ketones (**19**), and lactam (**25**) are tolerated, providing the desired products in 61–90% yield. Coordinating groups such as pyridines (**8**, **9**), pyrimidine (**21**), and tertiary amine (**10**), which may cause catalyst poisoning, do not inhibit the desired C–O cross-coupling reactions. Notably, exclusive chemoselectivity was observed when the substrate contained an N-nucleophile (e.g., **13**; vide infra). The high functional group tolerance renders this method applicable for late-stage functionalizations (LSFs) of natural and medicinal molecules such as (−)-nopol (**4**), tropicamide (**9**), perphenazin (**10**), pregnenolone (**19**), podophyllotoxin (**20**), and ezetimibe (**25**). Aldols (**22**), which are prone to dehydration under both acidic and basic conditions, can also be functionalized under the conditions reported here. Replacing Cu(acac)_2_ with Cu(TMHD)_2_ is crucial for the efficient bicyclopentalytion of secondary and tertiary alcohols (**19**). The reactivity of CN-BCP–TT^+^ (**1c**) is usually lower than for CF_3_-BCP–TT^+^ reagent **1b** (compare, for example, **16** and **26** as well as **13** and **27**). This difference in reactivity may be a consequence of electronic through-space interactions of the more electron-withdrawing cyano substituent, which lower the nucleophilicity of the bridgehead carbon radical toward the copper center. Although copper can be used as a catalyst (turnover number (TON) up to 7; Figure 2A, entry 8), 0.5 equiv of copper results in higher yields. Considering the complex starting materials typical for LSFs, the use of a simple copper salt in substoichiometric amounts may be acceptable.

**Figure 3:**
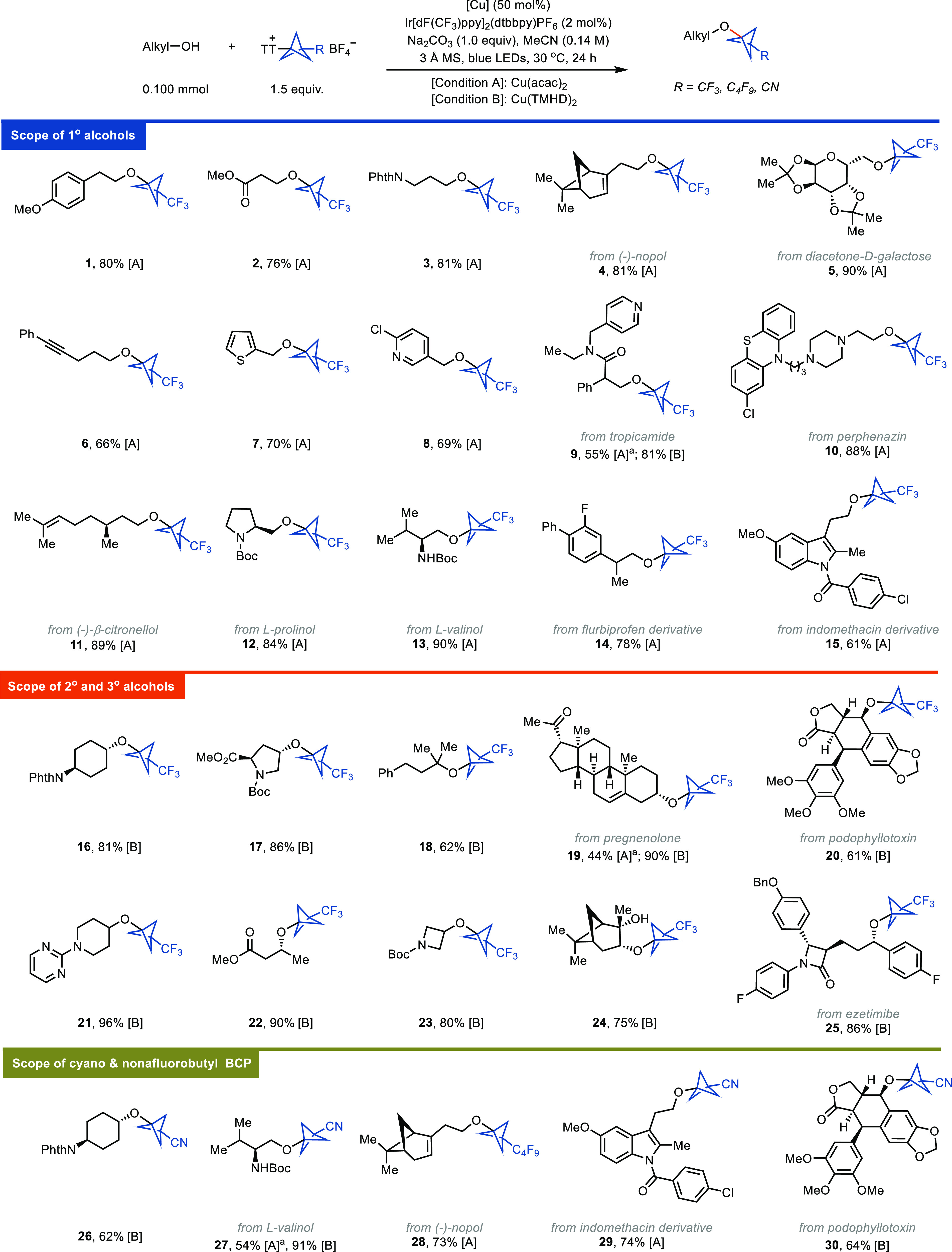
Substrate scope. ^a^Yields were determined by ^19^F NMR or ^1^H NMR.

While the scope for the alcohol is large, the scope of the BCP–TT^+^ reagents is limited to a rather small subset. Two aspects are conceptually challenging to expand the diversity of these reagents, at least in the chemistry presented here. Based on the data, it appears most likely that the reaction proceeds through a radical chain transfer, in which after photochemical initiation a TT radical cation is transferred from the starting material to the product (Figure 4A). For chain initiation and propagation, homolytic cleavage of the C–S bond in CF_3_-TT^+^ (**31**) is facile, which is the case for a rather stabilized CF_3_ radical. However, other substituents such as the methyl substituent in Me-TT^+^ (**33**) are bound more strongly, as supported by DFT calculations (Figure 4B). Second, other compounds that also feature a small bond dissociation energy (BDE) and would possibly engage in productive chain transfer could not be synthesized based on their lack of stability. For example, the difluoromethyl analogue of **31** (**32**; Figure 4 C) is unstable, presumably due to fast elimination from the cationic sulfonium salt. The cyano-substituted BCP compound **1c** was synthesized through a different procedure^5h^ and displays a unique synthesis that could not be extended to other nucleophiles, presumably due to competitive redox chemistry with oxidation of nucleophiles other than cyanide by the thianthrenium radical cation.^30,33^

**Figure 4:**
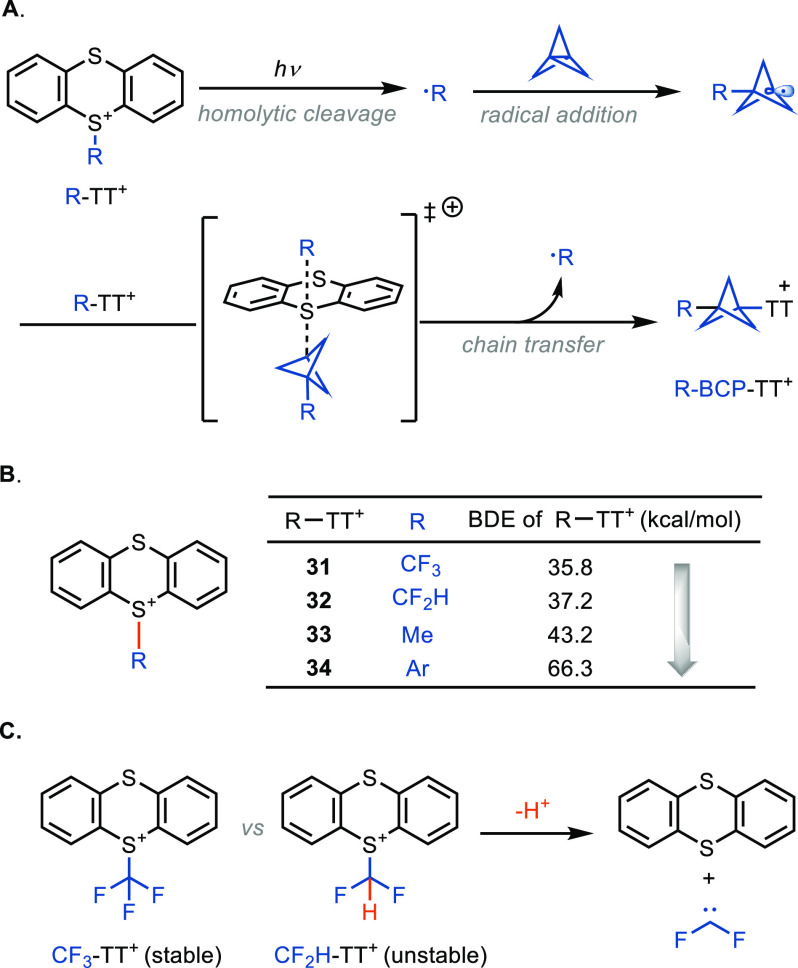
Mechanistic analysis of the synthesis of BCP–TT^+^ reagents. (A) Chain process of synthesis of BCP–TT^+^ reagents. (B) BDEs of different TT^+^ reagents. (C) Comparison of CF_3_-TT^+^ (**31**) and CF_2_H-TT^+^ (**32**). Ar = 3-fluoro-4-methoxyphenyl.

While the scope of the BCP–TT^+^ reagents is limited to only a few cases, we have highlighted the synthetic utility of our methodology to drug discovery with the few but relevant substituents in four BCP analogs of known pharmaceuticals (Figure 5A–D). The BCP analogue of fluoxetine hydrochloride, **36**, was synthesized in only two steps and 85% overall yield from **1b** and readily available starting material **35a**. The cyano group of BCP reagent **1c** can serve as a linchpin for the synthesis of other 1,3-disubstituted BCPs. For instance, cyano-BCP butyl ether (**37**) was obtained in 71% yield. Subsequently, hydrolysis of the cyano group to the corresponding carboxylic acid followed by alkylation afforded BCP-butoxycaine (**38**). Similarly, **41** underwent hydrolysis and amide condensation to give BCP-pranlukast (**42**). The cyano-BCP can also be selectively reduced to a BCP aldehyde, enabling reductive amination for the synthesis of BCP-safinamide (**40**). To the best of our knowledge, none of these analogs have been reported before. In addition, the cyano group in the BCP scaffold gives access to methylcarboxylate, methylamino, amide, and amino substituents (Figure 5E).

**Figure 5:**
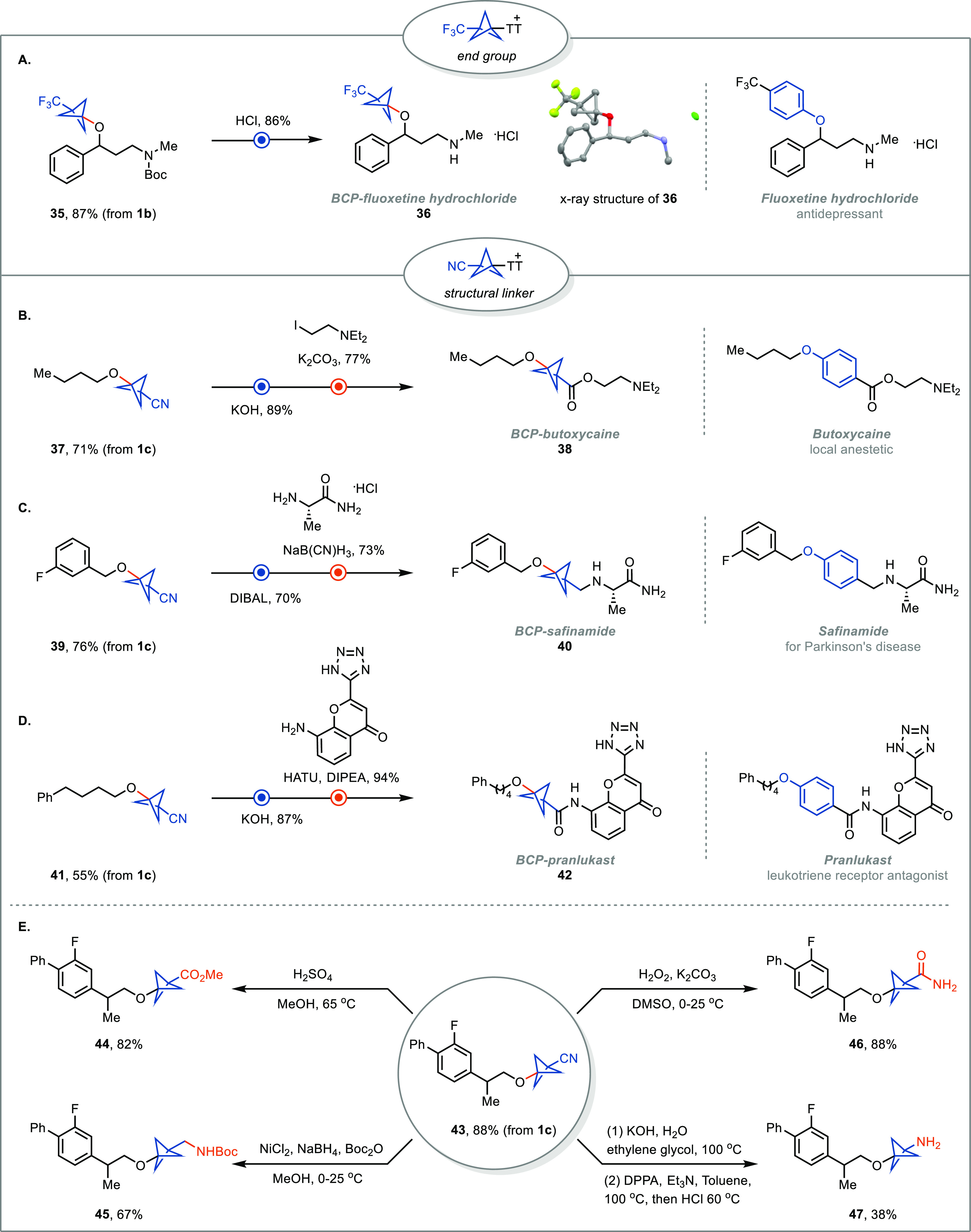
Synthesis of BCP pharmaceutical analogs. (A) Synthesis of BCP-fluoxetine hydrochloride. (B) Synthesis of BCP-butoxycaine. (C) Synthesis of BCP-safinamide. (D) Synthesis of BCP-pranlukast. (E) Further transformations of **43**.

In conclusion, we have described an efficient bicyclopentylation of alcohols with BCP–thianthrenium reagents, providing a variety of BCP alkyl ether products, even at a late stage. Importantly, several bioisosteric replacements of aryl rings in small-molecule drugs were realized, which would otherwise be difficult to access currently by other methods. We anticipate that our approach can facilitate the development of saturated analogs of alkyl aryl ether drugs in the pharmaceutical industry.

## Supporting Information Available

Experimental procedures, spectroscopic data, and NMR spectra of all products (PDF)
