# Supplementary material for: Bicyclopentylation of Alcohols with Thianthrenium Reagents
Source: J Am Chem Soc. 2023 Nov 27;145(48):25954–61. doi: 10.1021/jacs.3c10024 (PMC10704608; doi:10.1021/jacs.3c10024)
Supplement: Supplementary file 1 — ja3c10024_si_001.pdf [file ja3c10024_si_001.pdf]

SUPPORTING INFORMATION

Bicyclopentylation of Alcohols with Thianthrenium Reagents

Zibo Bai, Beatrice Lansbergen, and Tobias Ritter*

Max-Planck-Institut für Kohlenforschung, Kaiser-Wilhelm-Platz 1, D-45470 Mülheim an der Ruhr, Germany.

*E-mail: ritter@kofo.mpg.de

TABLE OF CONTENTS

TABLE OF CONTENTS 1

MATERIALS AND METHODS 8

EXPERIMENTAL DATA 9

General procedure for bicyclopentylation of primary alcohols with thianthrenium reagents 9

General procedure for bicyclopentylation of secondary and tertiary alcohols with thianthrenium reagents 9

Bicyclo[1.1.1]pentylether **1** 15

Bicyclo[1.1.1]pentylether **2** 15

Bicyclo[1.1.1]pentylether **3** 16

Bicyclo[1.1.1]pentylether **4** 17

Bicyclo[1.1.1]pentylether **5** 18

Bicyclo[1.1.1]pentylether **6** 18

Bicyclo[1.1.1]pentylether **7** 19

Bicyclo[1.1.1]pentylether **8** 20

Bicyclo[1.1.1]pentylether **9** 21

Bicyclo[1.1.1]pentylether **10** 22

Bicyclo[1.1.1]pentylether **11** 23

Bicyclo[1.1.1]pentylether **12** 23

Bicyclo[1.1.1]pentylether **13** 24

Bicyclo[1.1.1]pentylether **14** 25

Bicyclo[1.1.1]pentylether **15** 26

Bicyclo[1.1.1]pentylether **16** 27

Bicyclo[1.1.1]pentylether **17** 27

Bicyclo[1.1.1]pentylether **18** 28

Bicyclo[1.1.1]pentylether **19** 29

Bicyclo[1.1.1]pentylether **20** 30

Bicyclo[1.1.1]pentylether **21** 31

Bicyclo[1.1.1]pentylether **22** 31

Bicyclo[1.1.1]pentylether **23** 32

Bicyclo[1.1.1]pentylether **24** 33

Bicyclo[1.1.1]pentylether **25** 34

Bicyclo[1.1.1]pentylether **26** 35

Bicyclo[1.1.1]pentylether **27** 35

Bicyclo[1.1.1]pentylether **28** 36

Bicyclo[1.1.1]pentylether **29** 37

Bicyclo[1.1.1]pentylether **30** 38

Synthesis of BCP pharmaceutical analogs 39

Synthesis of BCP-fluoxetine hydrochloride (**36**) 39

Synthesis of BCP-butoxycaine (**38**) 40

Synthesis of BCP-safinamide (**40**) 42

Synthesis of BCP-pranlukast (**42**) 44

Bicyclo[1.1.1]pentylether **43** 46

Bicyclo[1.1.1]pentylester **44** 47

Bicyclo[1.1.1]pentylalkylamine **45** 47

Bicyclo[1.1.1]pentylamide **46** 48

Bicyclo[1.1.1]pentylamine **47** 49

Unsuccessful substrates 50

Mechanistic investigations 51

Radical trapping experiment 51

Stern-Volmer luminiscence quenching studies 51

EPR analysis 55

Alternative mechanism without TT involved 57

Unsuccessful attempts to other substituted BCP thianthrenium reagents 58

DFT calculations 58

Methods 58

Summary of calculation results 59

X-Ray Crystallographic Data (CCDC 2286412): 74

SPECTROSCOPIC DATA 79

^{1}H NMR of bicyclo[1.1.1]pentylether **1** 79

^{13}C NMR of bicyclo[1.1.1]pentylether **1** 80

^{19}F NMR of bicyclo[1.1.1]pentylether **1** 81

^{1}H NMR of bicyclo[1.1.1]pentylether **2** 82

^{13}C NMR of bicyclo[1.1.1]pentylether **2** 83

^{19}F NMR of bicyclo[1.1.1]pentylether **2** 84

^{1}H NMR of bicyclo[1.1.1]pentylether **3** 85

^{13}C NMR of bicyclo[1.1.1]pentylether **3** 86

^{19}F NMR of bicyclo[1.1.1]pentylether **3** 87
^{1}H NMR of bicyclo[1.1.1]pentylether **4** 88
^{13}C NMR of bicyclo[1.1.1]pentylether **4** 89
^{19}F NMR of bicyclo[1.1.1]pentylether **4** 90
^{1}H NMR of bicyclo[1.1.1]pentylether **5** 91
^{13}C NMR of bicyclo[1.1.1]pentylether **5** 92
^{19}F NMR of bicyclo[1.1.1]pentylether **5** 93
^{1}H NMR of bicyclo[1.1.1]pentylether **6** 94
^{13}C NMR of bicyclo[1.1.1]pentylether **6** 95
^{19}F NMR of bicyclo[1.1.1]pentylether **6** 96
^{1}H NMR of bicyclo[1.1.1]pentylether **7** 97
^{13}C NMR of bicyclo[1.1.1]pentylether **7** 98
^{19}F NMR of bicyclo[1.1.1]pentylether **7** 99
^{1}H NMR of bicyclo[1.1.1]pentylether **8** 100
^{13}C NMR of bicyclo[1.1.1]pentylether **8** 101
^{19}F NMR of bicyclo[1.1.1]pentylether **8** 102
^{1}H NMR of bicyclo[1.1.1]pentylether **9** 103
^{13}C NMR of bicyclo[1.1.1]pentylether **9** 104
^{19}F NMR of bicyclo[1.1.1]pentylether **9** 105
^{1}H NMR of bicyclo[1.1.1]pentylether **10** 106
^{13}C NMR of bicyclo[1.1.1]pentylether **10** 107
^{19}F NMR of bicyclo[1.1.1]pentylether **10** 108
^{1}H NMR of bicyclo[1.1.1]pentylether **11** 109
^{13}C NMR of bicyclo[1.1.1]pentylether **11** 110
^{19}F NMR of bicyclo[1.1.1]pentylether **11** 111
^{1}H NMR of bicyclo[1.1.1]pentylether **12** 112
^{13}C NMR of bicyclo[1.1.1]pentylether **12** 113
^{19}F NMR of bicyclo[1.1.1]pentylether **12** 114

^{1}H NMR of bicyclo[1.1.1]pentylether **13** 115

^{13}C NMR of bicyclo[1.1.1]pentylether **13** 116

^{19}F NMR of bicyclo[1.1.1]pentylether **13** 117

^{1}H NMR of bicyclo[1.1.1]pentylether **14** 118

^{13}C NMR of bicyclo[1.1.1]pentylether **14** 119

^{19}F NMR of bicyclo[1.1.1]pentylether **14** 120

^{1}H NMR of bicyclo[1.1.1]pentylether **15** 121

^{13}C NMR of bicyclo[1.1.1]pentylether **15** 122

^{19}F NMR of bicyclo[1.1.1]pentylether **15** 123

^{1}H NMR of bicyclo[1.1.1]pentylether **16** 124

^{13}C NMR of bicyclo[1.1.1]pentylether **16** 125

^{19}F NMR of bicyclo[1.1.1]pentylether **16** 126

^{1}H NMR of bicyclo[1.1.1]pentylether **17** 127

^{13}C NMR of bicyclo[1.1.1]pentylether **17** 128

^{19}F NMR of bicyclo[1.1.1]pentylether **17** 129

^{1}H NMR of bicyclo[1.1.1]pentylether **18** 130

^{13}C NMR of bicyclo[1.1.1]pentylether **18** 131

^{19}F NMR of bicyclo[1.1.1]pentylether **18** 132

^{1}H NMR of bicyclo[1.1.1]pentylether **19** 133

^{13}C NMR of bicyclo[1.1.1]pentylether **19** 134

^{19}F NMR of bicyclo[1.1.1]pentylether **19** 135

^{1}H NMR of bicyclo[1.1.1]pentylether **20** 136

^{13}C NMR of bicyclo[1.1.1]pentylether **20** 137

^{19}F NMR of bicyclo[1.1.1]pentylether **20** 138

^{1}H NMR of bicyclo[1.1.1]pentylether **21** 139

^{13}C NMR of bicyclo[1.1.1]pentylether **21** 140

^{19}F NMR of bicyclo[1.1.1]pentylether **21** 141

^{1}H NMR of bicyclo[1.1.1]pentylether **22** 142

^{13}C NMR of bicyclo[1.1.1]pentylether **22** 143
^{19}F NMR of bicyclo[1.1.1]pentylether **22** 144
^{1}H NMR of bicyclo[1.1.1]pentylether **23** 145
^{13}C NMR of bicyclo[1.1.1]pentylether **23** 146
^{19}F NMR of bicyclo[1.1.1]pentylether **23** 147
^{1}H NMR of bicyclo[1.1.1]pentylether **24** 148
^{13}C NMR of bicyclo[1.1.1]pentylether **24** 149
^{19}F NMR of bicyclo[1.1.1]pentylether **24** 150
^{1}H NMR of bicyclo[1.1.1]pentylether **25** 151
^{13}C NMR of bicyclo[1.1.1]pentylether **25** 152
^{19}F NMR of bicyclo[1.1.1]pentylether **25** 153
^{1}H NMR of bicyclo[1.1.1]pentylether **26** 154
^{13}C NMR of bicyclo[1.1.1]pentylether **26** 155
^{1}H NMR of bicyclo[1.1.1]pentylether **27** 156
^{13}C NMR of bicyclo[1.1.1]pentylether **27** 157
^{1}H NMR of bicyclo[1.1.1]pentylether **28** 158
^{13}C {1H, 19F} NMR of bicyclo[1.1.1]pentylether **28** 159
^{19}F NMR of bicyclo[1.1.1]pentylether **28** 160
^{1}H NMR of bicyclo[1.1.1]pentylether **29** 161
^{13}C NMR of bicyclo[1.1.1]pentylether **29** 162
^{1}H NMR of bicyclo[1.1.1]pentylether **30** 163
^{13}C NMR of bicyclo[1.1.1]pentylether **30** 164
^{1}H NMR of bicyclo[1.1.1]pentylether **35** 165
^{13}C NMR of bicyclo[1.1.1]pentylether **35** 166
^{19}F NMR of bicyclo[1.1.1]pentylether **35** 167
^{1}H NMR of bicyclo[1.1.1]pentylether **36** 168
^{13}C NMR of bicyclo[1.1.1]pentylether **36** 169
^{19}F NMR of bicyclo[1.1.1]pentylether **36** 170

^{1}H NMR of bicyclo[1.1.1]pentylether **37** 171
^{13}C NMR of bicyclo[1.1.1]pentylether **37** 172
^{1}H NMR of bicyclo[1.1.1]pentylether **37-1** 173
^{13}C NMR of bicyclo[1.1.1]pentylether **37-1** 174
^{1}H NMR of bicyclo[1.1.1]pentylether **38** 175
^{13}C NMR of bicyclo[1.1.1]pentylether **38** 176
^{1}H NMR of bicyclo[1.1.1]pentylether **39** 177
^{13}C NMR of bicyclo[1.1.1]pentylether **39** 178
^{19}F NMR of bicyclo[1.1.1]pentylether **39** 179
^{1}H NMR of bicyclo[1.1.1]pentylether **39-1** 180
^{13}C NMR of bicyclo[1.1.1]pentylether **39-1** 181
^{19}F NMR of bicyclo[1.1.1]pentylether **39-1** 182
^{1}H NMR of bicyclo[1.1.1]pentylether **40** 183
^{13}C NMR of bicyclo[1.1.1]pentylether **40** 184
^{19}F NMR of bicyclo[1.1.1]pentylether **40** 185
^{1}H NMR of bicyclo[1.1.1]pentylether **41** 186
^{13}C NMR of bicyclo[1.1.1]pentylether **41** 187
^{1}H NMR of bicyclo[1.1.1]pentylether **41-1** 188
^{13}C NMR of bicyclo[1.1.1]pentylether **41-1** 189
^{1}H NMR of bicyclo[1.1.1]pentylether **42** 190
^{13}C NMR of bicyclo[1.1.1]pentylether **42** 191
^{1}H NMR of bicyclo[1.1.1]pentylether **43** 192
^{13}C NMR of bicyclo[1.1.1]pentylether **43** 193
^{19}F NMR of bicyclo[1.1.1]pentylether **43** 194
^{1}H NMR of bicyclo[1.1.1]pentylether **44** 195
^{13}C NMR of bicyclo[1.1.1]pentylether **44** 196
^{19}F NMR of bicyclo[1.1.1]pentylether **44** 197
^{1}H NMR of bicyclo[1.1.1]pentylether **45** 198

^{13}C NMR of bicyclo[1.1.1]pentylether **45** 199

^{19}F NMR of bicyclo[1.1.1]pentylether **45** 200

^{1}H NMR of bicyclo[1.1.1]pentylether **46** 201

^{13}C NMR of bicyclo[1.1.1]pentylether **46** 202

^{19}F NMR of bicyclo[1.1.1]pentylether **46** 203

^{1}H NMR of bicyclo[1.1.1]pentylether **47** 204

^{13}C NMR of bicyclo[1.1.1]pentylether **47** 205

^{19}F NMR of bicyclo[1.1.1]pentylether **47** 206

REFERENCES 207

MATERIALS AND METHODS

All reactions were carried out under an ambient atmosphere unless otherwise stated and monitored by thin-layer chromatography (TLC). Air- and moisture-sensitive manipulations were performed using standard Schlenk- and glove-box techniques under an atmosphere of argon or dinitrogen. High-resolution mass spectra were obtained using *Q Exactive Plus* from *Thermo*. Concentration under reduced pressure was performed by rotary evaporation at 25–40 °C at an appropriate pressure. Purified compounds were further dried under high vacuum (0.010–0.005 mBar). Yields refer to purified and spectroscopically pure compounds, unless otherwise stated.

Solvents

Anhydrous DCE and DMA were purchased from Acros Organics and Sigma Aldrich. Other anhydrous solvents were obtained from Phoenix Solvent Drying Systems. All deuterated solvents were purchased from Euriso-Top.

Chromatography

Thin layer chromatography (TLC) was performed using EMD TLC plates pre-coated with 250 µm thickness silica gel 60 F_{254} plates and visualized by fluorescence quenching under UV light and $KMnO_4$ stain. Flash column chromatography was performed using silica gel (40–63 µm particle size) purchased from Geduran®.

Photochemistry

All *O*-alkylation reactions were performed with blue LEDs (Kessil A160WE Tuna Blue (460 nm), LED lighting, 40 W).

Spectroscopy and Instruments

NMR spectra were recorded on a Bruker Ascend™ 500 spectrometer operating at 500 MHz, 471 MHz and 126 MHz, for ^{1}H, ^{19}F and ^{13}C acquisitions, respectively; or on a Varian Unity/Inova 600 spectrometer operating at 600 MHz and 151 MHz for ^{1}H and ^{13}C acquisitions, respectively. Chemical shifts are reported in ppm with the solvent residual peak as the internal standard. For ^{1}H NMR: $CDCl_3$, δ 7.26; CD_3CN, δ 1.96; CD_2Cl_2, δ 5.32; DMSO-d_6, δ 2.50. For ^{13}C NMR: $CDCl_3$, δ 77.16; CD_3CN, δ 1.32; CD_2Cl_2, δ 53.84; DMSO-d_6, δ 39.52.[1] ^{19}F NMR spectra were referenced using a unified chemical shift scale based on the ^{1}H resonance of tetramethylsilane (1% v/v solution in the respective solvent).[2] Data is reported as follows: s = singlet, d = doublet, t = triplet, q = quartet, quin = quintet, sext = sextet, sept = septet, m = multiplet, bs = broad singlet; coupling constants are reported in Hz.

Starting materials

All substrates were used as received from commercial suppliers, unless otherwise stated. Trifluoromethylbicyclo[1.1.1]pentyl thianthrenium salt (CF_3BCP-TT^+ BF_4^-), nonafluorobutylbicyclo[1.1.1] pentyl

thianthrenium salt (C_4F_9BCP-TT$^+$ BF_4^-), and cyanobicyclo[1.1.1]pentyl thianthrenium salt (CNBCP-TT$^+$ BF_4^-) were prepared according to the literature.[3] Cu(acac)$_2$ (97%) was purchased from Sigma-Aldrich. Cu(TMHD)$_2$ (99.9%) was purchased from Alfa Aesar. Na_2CO_3 (99.6%) was purchased from Acros Organics.

EXPERIMENTAL DATA

General procedure for bicyclopentylation of primary alcohols with thianthrenium reagents

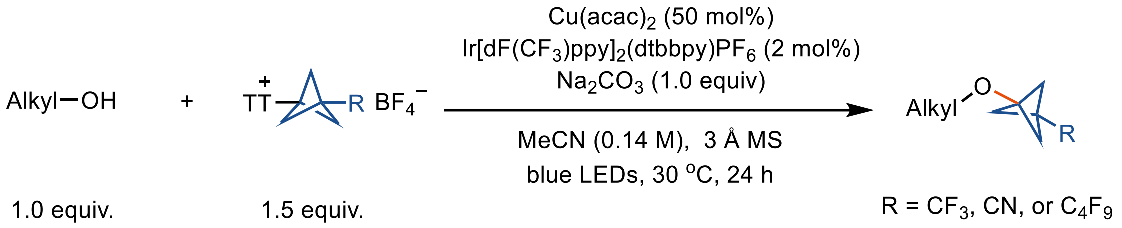

Under nitrogen atmosphere, to a 4 mL borosilicate vial equipped with a magnetic stir bar were added alcohol (0.100 mmol, 1.00 equiv.), BCP-TT$^+$ BF_4^- reagent (0.150 mmol, 1.50 equiv.), Ir[dF(CF_3)ppy]$_2$(dtbbpy)PF_6 (2 mg, 2 µmoL, 2 mol%), Cu(acac)$_2$ (13 mg, 50 µmoL, 50 mol%), Na_2CO_3 (10.6 mg, 0.100 mmol, 1.00 equiv.), 3Å molecular sieves (120 mg), and anhydrous MeCN (0.70 mL, c = 0.14 M). The vial was sealed with a septum-cap. Then, the mixture was stirred for 1 h at 25 °C, and placed 5 cm away from two blue LEDs (Kessil A160WE Tuna Blue (460 nm), LED lighting, 40 W). The mixture was irradiated for 24 h while maintaining the temperature at approximately 30 °C through cooling with a fan. After irradiation, the mixture was concentrated under reduced pressure. The residue was purified by flash column chromatography on silica gel to afford the desired product.

Note: The reaction is air- and water sensitive. Schlenk technique was used to avoid air and water. For simplicity, in our research, we have opted to execute the transformation for most compounds by using a glovebox. Control experiments showed that the yields were within error of measurement if the reaction was carried out using a glovebox or Schlenk technique.

Safety statement: Blue light is harmful to the retina. Safety glasses should be worn when using these lamps.

General procedure for bicyclopentylation of secondary and tertiary alcohols with thianthrenium reagents

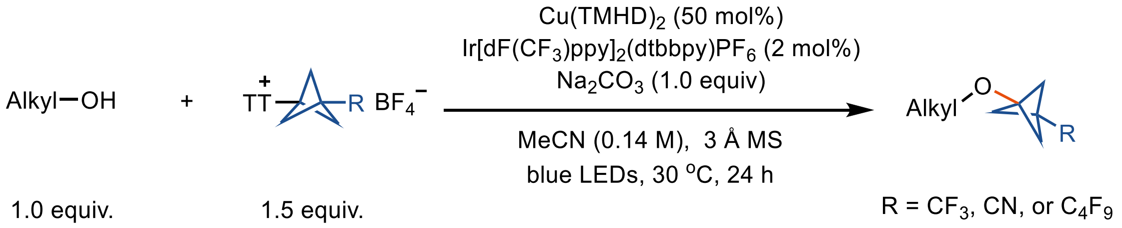

Under nitrogen atmosphere, to a 4 mL borosilicate vial equipped with a magnetic stir bar were added alcohol (0.100 mmol, 1.00 equiv.), BCP-TT$^+$ BF_4^- reagent (0.150 mmol, 1.50 equiv.), Ir[dF(CF_3)ppy]$_2$(dtbbpy)PF_6 (2 mg, 2 µmoL, 2 mol%), Cu(TMHD)$_2$ (22 mg, 50 µmoL, 50 mol%), Na_2CO_3 (10.6 mg, 0.100 mmol, 1.00 equiv.), 3Å molecular sieves (120 mg), and anhydrous MeCN (0.70 mL, *c* = 0.14 M). The vial was sealed with a septum-cap. Then, the mixture was stirred for 1 h at 25 °C, and placed 5 cm away from two blue LEDs (Kessil A160WE Tuna Blue (460 nm), LED lighting, 40 W). The mixture was irradiated for 24 h while maintaining the temperature at approximately 30 °C through cooling with a fan. After irradiation, the mixture was concentrated under reduced pressure. The residue was purified by flash column chromatography on silica gel to afford the desired product.

Note: The reaction is air- and water sensitive. Schlenk technique was used to avoid air and water. For simplicity, in our research, we have opted to execute the transformation for most compounds by using a glovebox. Control experiments showed that the yields were within error of measurement if the reaction was carried out using a glovebox or Schlenk technique.

Table S1. Conditions optimization of bicyclopentylation of **1a** with **1b**[a]

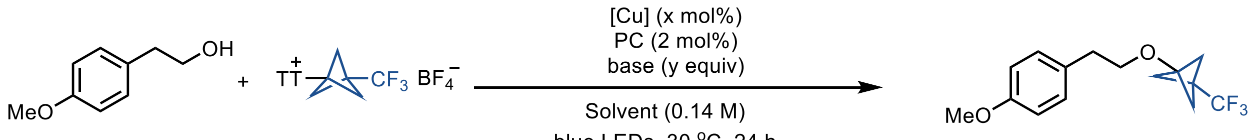

1a **1b** (1.5 equiv.) **1**

Entry	[Cu] (equiv.)	PC	base (equiv.)	solvent	3Å MS	Pre-strring	Yield of **1** (%)
1	CuTc (1.0)	Ir(ppy)$_3$	Na_2CO_3 (1.0)	MeCN	–	–	17
2	Cu(acac)$_2$ (1.0)	Ir(ppy)$_3$	Na_2CO_3 (1.0)	MeCN	–	–	17
3	CuCl (1.0)	Ir(ppy)$_3$	Na_2CO_3 (1.0)	MeCN	–	–	<3
4	Cu(MeCN)$_4$BF$_4$ (1.0)	Ir(ppy)$_3$	Na_2CO_3 (1.0)	MeCN	–	–	<3
5	CuOTf (1.0)	Ir(ppy)$_3$	Na_2CO_3 (1.0)	MeCN	–	–	<3
6	CuBr (1.0)	Ir(ppy)$_3$	Na_2CO_3 (1.0)	MeCN	–	–	<5
7	Cu(acac)$_2$ (0.5)	Ir(ppy)$_3$	Na_2CO_3 (2.0)	MeCN	–	40 min	36
8	Cu(acac)$_2$ (0.5)	Ir(ppy)$_3$	Cs_2CO_3 (2.0)	MeCN	–	40 min	18

9	Cu(acac)$_2$ (0.5)	Ir(ppy)$_3$	K_3PO_4 (2.0)	MeCN	–	40 min	31
10	Cu(acac)$_2$ (0.5)	Ir(ppy)$_3$	^{t}BuOK (2.0)	MeCN	–	40 min	4
11	Cu(acac)$_2$ (0.5)	Ir(ppy)$_3$	DBU (2.0)	MeCN	–	40 min	14
12	Cu(acac)$_2$ (0.5)	Ir(ppy)$_3$	DIPEA (2.0)	MeCN	–	40 min	18
13	Cu(acac)$_2$ (0.5)	Ir(dtbbpy)ppy$_2$PF$_6$	Na_2CO_3 (2.0)	MeCN	–	40 min	16
14	Cu(acac)$_2$ (0.5)	Ir[dF(CF$_3$)ppy]$_2$(dtbbpy)PF$_6$	Na_2CO_3 (2.0)	MeCN	–	40 min	41
15	Cu(acac)$_2$ (0.5)	Ir[dF(CF$_3$)ppy]$_2$(dtbbpy)PF$_6$	Na_2CO_3 (2.0)	MeCN	+	2h	64
16	Cu(acac)$_2$ (0.5)	Ir[dF(CF$_3$)ppy]$_2$(dtbbpy)PF$_6$	Na_2CO_3 (2.0)	DCE	+	2h	57
17	Cu(acac)$_2$ (0.5)	Ir[dF(CF$_3$)ppy]$_2$(dtbbpy)PF$_6$	Na_2CO_3 (2.0)	1,4-dioxane	+	2h	4
18	Cu(acac)$_2$ (0.5)	Ir[dF(CF$_3$)ppy]$_2$(dtbbpy)PF$_6$	Na_2CO_3 (2.0)	acetone	+	2h	36
19	Cu(acac)$_2$ (0.5)	Ir[dF(CF$_3$)ppy]$_2$(dtbbpy)PF$_6$	Na_2CO_3 (2.0)	DMA	+	2h	11
20	Cu(acac)$_2$ (0.5)	Ir[dF(CF$_3$)ppy]$_2$(dtbbpy)PF$_6$	Na_2CO_3 (2.0)	^{t}BuCN	+	2h	36
21	Cu(acac)$_2$ (0.25)	Ir[dF(CF$_3$)ppy]$_2$(dtbbpy)PF$_6$	Na_2CO_3 (2.0)	MeCN	+	2h	36
22	Cu(acac)$_2$ (0.75)	Ir[dF(CF$_3$)ppy]$_2$(dtbbpy)PF$_6$	Na_2CO_3 (2.0)	MeCN	+	2h	33
23	Cu(acac)$_2$ (0.5)	Ir[dF(CF$_3$)ppy]$_2$(dtbbpy)PF$_6$	Na_2CO_3 (1.0)	MeCN	+	2h	72
24	Cu(acac)$_2$ (0.5)	Ir[dF(CF$_3$)ppy]$_2$(dtbbpy)PF$_6$	Na_2CO_3 (1.0)	MeCN	+	1h	85
25	Cu(acac)$_2$ (0.5)	Ir[dF(CF$_3$)ppy]$_2$(dtbbpy)PF$_6$	Na_2CO_3 (1.0)	MeCN	+	3h	73
26	Cu(MeCN)$_4$BF$_4$ (0.5)	Ir[dF(CF$_3$)ppy]$_2$(dtbbpy)PF$_6$	Na_2CO_3 (1.0)	MeCN	+	1h	<3
27	Cu(MeCN)$_4$BF$_4$ (0.5)[b]	Ir[dF(CF$_3$)ppy]$_2$(dtbbpy)PF$_6$	Na_2CO_3 (1.0)	MeCN	+	1h	32
28	Cu(OTf)$_2$ (0.5)	Ir[dF(CF$_3$)ppy]$_2$(dtbbpy)PF$_6$	Na_2CO_3 (1.0)	MeCN	+	1h	<3
29	Cu(OTf)$_2$ (0.5)[b]	Ir[dF(CF$_3$)ppy]$_2$(dtbbpy)PF$_6$	Na_2CO_3 (1.0)	MeCN	+	1h	36

30	$Cu(TMHD)_2$ (0.5)	$Ir[dF(CF_3)ppy]_2(dtbbpy)PF_6$	Na_2CO_3 (1.0)	MeCN	+	1h	94
31[c]	$Cu(acac)_2$ (0.5)	$Ir[dF(CF_3)ppy]_2(dtbbpy)PF_6$	Na_2CO_3 (1.0)	MeCN	+	1h	17
32[cd]	$Cu(acac)_2$ (0.5)	$Ir[dF(CF_3)ppy]_2(dtbbpy)PF_6$	Na_2CO_3 (1.0)	MeCN	+	1h	15

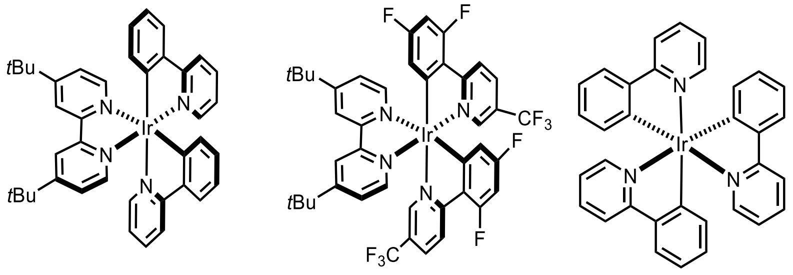

$Ir(dtbbpy)(ppy)_2PF_6$ $Ir[dF(CF_3)ppy]_2(dtbbpy)PF_6$ $Ir(ppy)_3$

[a]4-methoxyphenethyl acohol (0.05 mmol, 1 equiv), **CF_3BCP-TT$^+$ BF_4^-** (0.075 mmol, 1.5 equiv.). ^{19}F NMR yield using $PhCF_3$ as an internal standard. [b]2,4-pentandione (1.0 equiv.) was added. [c]The reaction time is 1 h. [d]20 mol% TT was added as additive.

Procedure: Under nitrogen atmosphere, to a 4 mL borosilicate vial equipped with a magnetic stir bar were added 4-methoxyphenethyl acohol (7.5 mg, 0.050 mmol, 1.0 equiv.), CF_3BCP-TT$^+$ BF_4^- (33 mg, 0.075 mmol, 1.5 equiv.), photocatalyst (1 µmoL, 2 mol%), [Cu] (x mol%), base (y equiv.), and solvent (0.35 mL, *c* = 0.14 M). The vial was sealed with a septum-cap. Then, the mixture was stirred for 0–2 h at 25 °C, and placed 5 cm away from two blue LEDs (Kessil A160WE Tuna Blue (460 nm), LED lighting, 40 W). The mixture was irradiated for 24 h while maintaining the temperature at approximately 30 °C through cooling with a fan. After irradiation, the mixture was dilutued with $CDCl_3$ (ca. 0.5 mL), and $PhCF_3$ (7.3 mg, 0.050 mmol, 1.0 equiv) was added as the internal standard. Then, ca. 200 µL of the mixture was transferred into the NMR tube by a syringe with a syringe filter, and dilutued with $CDCl_3$ (ca. 0.5 mL) for ^{19}F NMR analysis. A singlet peak at –63.72 ppm ($PhCF_3$) was set as 1.00. Yield of **1** = integration of peak [δ: –70.12 (s, 3F)] × 100%.

Table S2. Control experiments of bicyclopentylation of **1a** with **1b**[a]

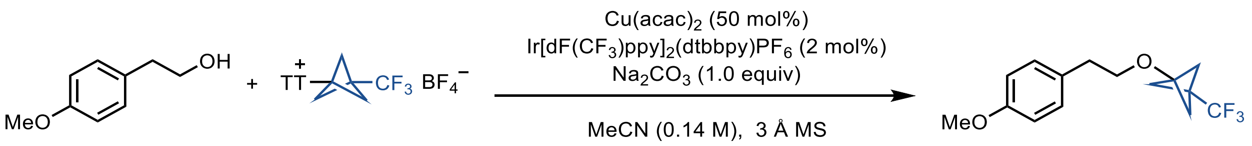

1a **1b**, 1.5 equiv. **1**

entry	change from the standard conditions	Yield of **1** (%)
1	None	85/80[b]

2	No Na_2CO_3	11
3	No Ir[dF(CF_3)ppy]$_2$(dtbbpy)PF_6	<3
4	No Cu(acac)$_2$	<3
5	No 3Å MS	48
6	No 3Å MS + H_2O (10 µL)	21
7	No blue LEDs	<3
8	Cu(acac)$_2$ (10 mol%)	70
9	No blue LEDs and PC	<3
10	No blue LEDs and PC, Cu(acac)$_2$ → CuCl	<3
11	No blue LEDs and PC, Cu(acac)$_2$ → CuTc	<3
12	No blue LEDs and PC, Cu(acac)$_2$ → Cu(MeCN)$_4$BF$_4$	<3

[a]4-methoxyphenethyl acohol (0.05 mmol, 1 equiv), **CF_3BCP-TT$^+$ BF_4^-** (0.075 mmol, 1.5 equiv.), Ir[dF(CF_3)ppy]$_2$(dtbbpy)PF_6 (2 mol%), Cu(acac)$_2$ (50 mol%), Na_2CO_3 (1 equiv), MeCN (0.14 M), 30 °C, 24 h, blue LED. ^{19}F NMR yield using $PhCF_3$ as an internal standard. [b]isolated yield based on 4-methoxyphenethyl acohol (0.100 mmol).

Procedure: Under nitrogen atmosphere, to a 4 mL borosilicate vial equipped with a magnetic stir bar were added 4-methoxyphenethyl acohol (7.5 mg, 0.050 mmol, 1.0 equiv.), CF_3BCP-TT$^+$ BF_4^- (33 mg, 0.075 mmol, 1.5 equiv.), Ir[dF(CF_3)ppy]$_2$(dtbbpy)PF_6 (1 mg, 1 µmoL, 2 mol%), Cu(acac)$_2$ (6.5 mg, 25 µmoL, 50 mol%), Na_2CO_3 (5.3 mg, 0.10 mmol, 1.0 equiv.), 3Å molecular sieves (60 mg), and anhydrous MeCN (0.35 mL, c = 0.14 M). Then, the mixture was stirred for 1 h at 25 °C, and placed 5 cm away from two blue LEDs (Kessil A160WE Tuna Blue (460 nm), LED lighting, 40 W). The mixture was irradiated for 24 h while maintaining the temperature at approximately 30 °C through cooling with a fan. After irradiation, the mixture was dilutued with $CDCl_3$ (ca. 0.5 mL), and $PhCF_3$ (7.3 mg, 0.050 mmol, 1.0 equiv) was added as the internal standard. Then, ca. 200 µL of the mixture was transferred into the NMR tube by a syringe with a syringe filter, and dilutued with $CDCl_3$ (ca. 0.5 mL) for ^{19}F NMR analysis. A singlet peak at –63.72 ppm ($PhCF_3$) was set as 1.00. Yield of **1** = integration of peak [δ: –70.12 (s, 3F)] × 100%.

Table S3. Conditions optimization of bicyclopentylation of **19a** with **1b**[a]

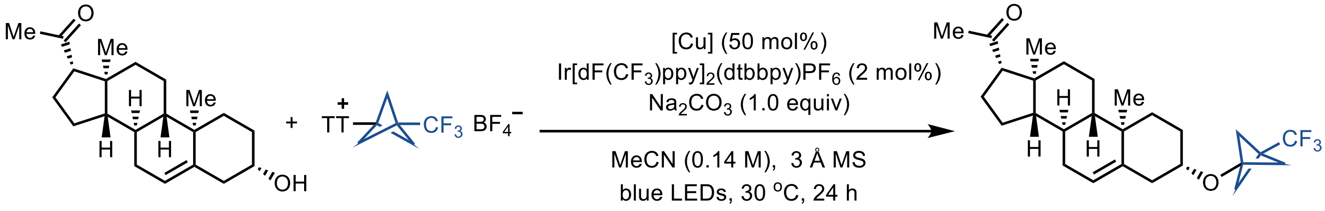

19a **1b**, 1.5 equiv. **19**

entry	Cu catalyst	Yield of **19** (%)
1	Cu(acac)$_2$	44
2	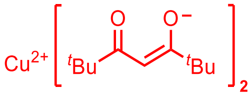	95/90[b]
3	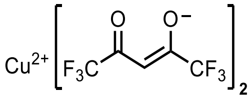	<3
4	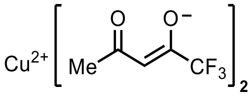	<3

[a]pregnenolone (0.05 mmol, 1 equiv), **CF_3BCP-TT$^+$ BF_4^-** (0.075 mmol, 1.5 equiv.). ^{19}F NMR yield using $PhCF_3$ as an internal standard.

Procedure: Under nitrogen atmosphere, to a 4 mL borosilicate vial equipped with a magnetic stir bar were added pregnenolone (18 mg, 0.050 mmol, 1.0 equiv.), CF_3BCP-TT$^+$ BF_4^- (33 mg, 0.075 mmol, 1.5 equiv.), Ir[dF(CF_3)ppy]$_2$(dtbbpy)PF_6 (1 mg, 1 µmoL, 2 mol%), [Cu] (25 µmoL, 50 mol%), Na_2CO_3 (5.3 mg, 0.10 mmol, 1.0 equiv.), 3Å molecular sieves (60 mg), and anhydrous MeCN (0.35 mL, *c* = 0.14 M). The vial was sealed with a septum-cap. Then, the mixture was stirred for 1 h at 25 °C, and placed 5 cm away from two blue LEDs (Kessil A160WE Tuna Blue (460 nm), LED lighting, 40 W). The mixture was irradiated for 24 h while maintaining the temperature at approximately 30 °C through cooling with a fan. After irradiation, the mixture was dilutued with $CDCl_3$ (ca. 0.5 mL), and $PhCF_3$ (7.3 mg, 0.050 mmol, 1.0 equiv) was added as the internal standard. Then, ca. 200 µL of the mixture was transferred into the NMR tube by a syringe with a syringe filter, and dilutued with $CDCl_3$ (ca. 0.5 mL) for ^{19}F NMR analysis. A singlet peak at –63.72 ppm ($PhCF_3$) was set as 1.00. Yield of **19** = integration of peak [δ: –70.08 (s, 3F)] × 100%.

Bicyclo[1.1.1]pentylether 1

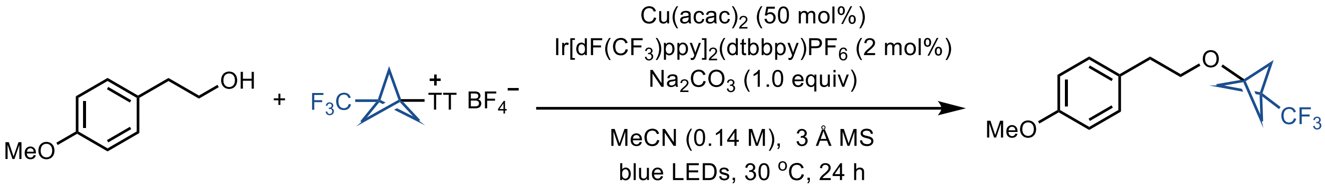

1.0 equiv. 1.5 equiv. **80%** **1**

Under nitrogen atmosphere, to a 4 mL borosilicate vial equipped with a magnetic stir bar were added 4-methoxyphenethyl acohol (15.2 mg, 0.100 mmol, 1.00 equiv.), CF_3BCP-TT^+ BF_4^- (65.7 mg, 0.150 mmol, 1.50 equiv.), $Ir[dF(CF_3)ppy]_2(dtbbpy)PF_6$ (2 mg, 2 µmoL, 2 mol%), $Cu(acac)_2$ (13 mg, 50 µmoL, 50 mol%), Na_2CO_3 (10.6 mg, 0.100 mmol, 1.00 equiv.), 3Å molecular sieves (120 mg), and anhydrous MeCN (0.70 mL, *c* = 0.14 M). The vial was sealed with a septum-cap. Then, the mixture was stirred for 1 h at 25 °C, and placed 5 cm away from two blue LEDs (Kessil A160WE Tuna Blue (460 nm), LED lighting, 40 W). The mixture was irradiated for 24 h while maintaining the temperature at approximately 30 °C through cooling with a fan. After irradiation, the mixture was concentrated under reduced pressure. The residue was purified by flash column chromatography on silica gel eluting with EtOAc/pentane (0:100–1:40 (v/v)) to afford the title compound **1** as a colorless oil (22.9 mg, 80.0 µmol, 80%).

R_f = 0.58 (pentane/EtOAc, 20:1 (v/v)).

NMR Spectroscopy:

^{1}H NMR (500 MHz, $CDCl_3$, 298 K, δ): 7.13 (d, *J* = 8.7 Hz, 2H), 6.84 (d, *J* = 8.7 Hz, 2H), 3.79 (s, 3H), 3.62 (t, *J* = 7.3 Hz, 2H), 2.83 (t, *J* = 7.3 Hz, 2H), 2.09 (s, 6H).

^{13}C NMR (151 MHz, $CDCl_3$, 298 K, δ): 158.4, 130.4, 130.0, 124.5 (q, *J* = 273.4 Hz), 114.0, 68.2, 66.3 (q, *J* = 2.2 Hz), 55.4, 50.7 (q, *J* = 1.9 Hz), 35.4, 31.2 (q, *J* = 39.7 Hz).

^{19}F NMR (565 MHz, $CDCl_3$, 298 K, δ): –70.12 (s).

HRMS-ESI (m/z) calc'd for $C_{15}H_{17}O_2F_3Na^+$ $[M+Na]^+$, 309.1073; found, 309.1074; deviation: –0.4 ppm.

Bicyclo[1.1.1]pentylether 2

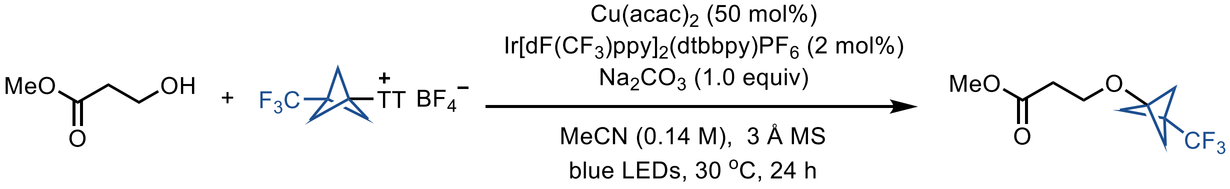

1.0 equiv. 1.5 equiv. **76%** **2**

Under nitrogen atmosphere, to a 4 mL borosilicate vial equipped with a magnetic stir bar were added 3-hydroxy-propanoicacidmethylester (10.4 mg, 0.100 mmol, 1.00 equiv.), CF_3BCP-TT^+ BF_4^- (65.7 mg, 0.150 mmol, 1.50 equiv.), $Ir[dF(CF_3)ppy]_2(dtbbpy)PF_6$ (2 mg, 2 µmoL, 2 mol%), $Cu(acac)_2$ (13 mg, 50 µmoL, 50

mol%), Na_2CO_3 (10.6 mg, 0.100 mmol, 1.00 equiv.), 3Å molecular sieves (120 mg), and anhydrous MeCN (0.70 mL, *c* = 0.14 M). The vial was sealed with a septum-cap. Then, the mixture was stirred for 1 h at 25 °C, and placed 5 cm away from two blue LEDs (Kessil A160WE Tuna Blue (460 nm), LED lighting, 40 W). The mixture was irradiated for 24 h while maintaining the temperature at approximately 30 °C through cooling with a fan. After irradiation, the mixture was concentrated under reduced pressure. The residue was purified by flash column chromatography on silica gel eluting with EtOAc/pentane (0:100–1:15 (v/v)) to afford the title compound **2** as a colorless oil (18.1 mg, 76.0 µmol, 76%).

R_f = 0.60 (pentane/EtOAc, 10:1 (v/v)).

NMR Spectroscopy:

^{1}H NMR (500 MHz, $CDCl_3$, 298 K, δ): 3.73 (t, *J* = 6.4 Hz, 2H), 3.70 (s, 3H), 2.59 (t, *J* = 6.4 Hz, 2H), 2.12 (s, 6H).

^{13}C NMR (126 MHz, $CDCl_3$, 298 K, δ): 171.7, 124.4 (q, *J* = 273.6 Hz), 66.3 (d, *J* = 2.4 Hz), 62.4, 51.9, 50.7 (d, *J* = 1.2 Hz), 34.7, 31.1 (q, *J* = 39.9 Hz).

^{19}F NMR (471 MHz, $CDCl_3$, 298 K, δ): –70.11 (s).

HRMS-ESI (m/z) calc'd for $C_{10}H_{13}O_3F_3Na^+$ $[M+Na]^+$, 261.0709; found, 261.0711; deviation: –0.8 ppm.

Bicyclo[1.1.1]pentylether 3

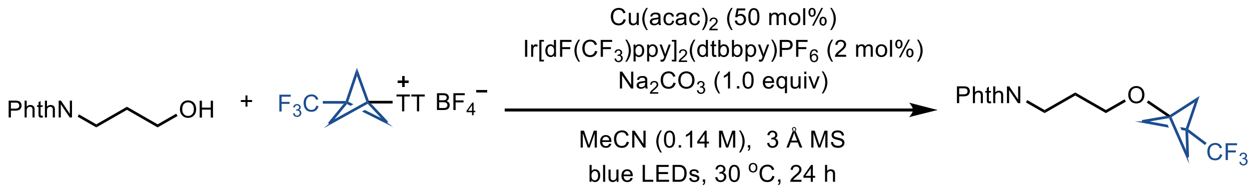

1.0 equiv. 1.5 equiv. **81%** **3**

Under nitrogen atmosphere, to a 4 mL borosilicate vial equipped with a magnetic stir bar were added N-(3-hydroxypropyl)phthalimide (20.5 mg, 0.100 mmol, 1.00 equiv.), CF_3BCP-TT$^+$ BF_4^- (65.7 mg, 0.150 mmol, 1.50 equiv.), Ir[dF(CF_3)ppy]$_2$(dtbbpy)PF_6 (2 mg, 2 µmoL, 2 mol%), Cu(acac)$_2$ (13 mg, 50 µmoL, 50 mol%), Na_2CO_3 (10.6 mg, 0.100 mmol, 1.00 equiv.), 3Å molecular sieves (120 mg), and anhydrous MeCN (0.70 mL, *c* = 0.14 M). The vial was sealed with a septum-cap. Then, the mixture was stirred for 1 h at 25 °C, and placed 5 cm away from two blue LEDs (Kessil A160WE Tuna Blue (460 nm), LED lighting, 40 W). The mixture was irradiated for 24 h while maintaining the temperature at approximately 30 °C through cooling with a fan. After irradiation, the mixture was concentrated under reduced pressure. The residue was purified by flash column chromatography on silica gel eluting with EtOAc/pentane (0:100–1:20 (v/v)) to afford the title compound **3** as a colorless oil (27.5 mg, 81.0 µmol, 81%).

R_f = 0.33 (pentane/EtOAc, 10:1 (v/v)).

NMR Spectroscopy:

^{1}H NMR (600 MHz, $CDCl_3$, 298 K, δ): 8.02 – 7.76 (m, 2H), 7.75 – 7.58 (m, 2H), 3.80 (t, J = 6.8 Hz, 2H), 3.50 (t, J = 6.0 Hz, 2H), 2.04 (d, J = 0.5 Hz, 6H), 2.01 – 1.83 (m, 2H).

^{13}C NMR (151 MHz, $CDCl_3$, 298 K, δ): 168.5, 134.1, 132.3, 124.5 (q, J = 273.4 Hz), 123.3, 66.1 (d, J = 2.2 Hz), 64.6, 50.6 (d, J = 1.9 Hz), 35.6, 31.0 (q, J = 39.7 Hz), 28.5.

^{19}F NMR (565 MHz, $CDCl_3$, 298 K, δ): –70.13 (s).

HRMS-ESI (m/z) calc'd for $C_{17}H_{16}NO_3F_3Na^+$ [M+Na]$^+$, 362.0974; found, 362.0976; deviation: –0.5 ppm.

Bicyclo[1.1.1]pentylether 4

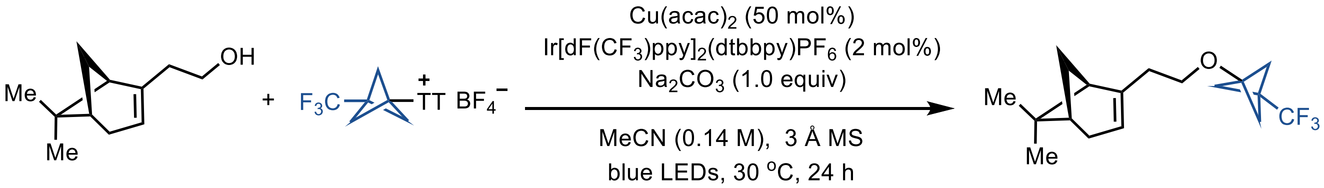

1.0 equiv. 1.5 equiv. **81%** **4**

Under nitrogen atmosphere, to a 4 mL borosilicate vial equipped with a magnetic stir bar were added (-)-nopol (16.6 mg, 0.100 mmol, 1.00 equiv.), CF_3BCP-TT$^+$ BF_4^- (65.7 mg, 0.150 mmol, 1.50 equiv.), Ir[dF(CF_3)ppy]$_2$(dtbbpy)PF_6 (2 mg, 2 µmoL, 2 mol%), Cu(acac)$_2$ (13 mg, 50 µmoL, 50 mol%), Na_2CO_3 (10.6 mg, 0.100 mmol, 1.00 equiv.), 3Å molecular sieves (120 mg), and anhydrous MeCN (0.70 mL, c = 0.14 M). The vial was sealed with a septum-cap. Then, the mixture was stirred for 1 h at 25 °C, and placed 5 cm away from two blue LEDs (Kessil A160WE Tuna Blue (460 nm), LED lighting, 40 W). The mixture was irradiated for 24 h while maintaining the temperature at approximately 30 °C through cooling with a fan. After irradiation, the mixture was concentrated under reduced pressure. The residue was purified by flash column chromatography on silica gel eluting with EtOAc/pentane (0:100–1:50 (v/v)) to afford the title compound **4** as a colorless oil (24.3 mg, 81.0 µmol, 81%).

R_f = 0.76 (pentane/EtOAc, 20:1 (v/v)).

NMR Spectroscopy:

^{1}H NMR (500 MHz, $CDCl_3$, 298 K, δ): 5.27 (dt, J = 3.1, 1.5 Hz, 1H), 3.44 (t, J = 7.2 Hz, 2H), 2.36 (dt, J = 8.5, 5.6 Hz, 1H), 2.30 – 2.15 (m, 4H), 2.14 – 2.05 (m, 7H), 2.02 (t, J = 4.8 Hz, 1H), 1.27 (s, 3H), 1.15 (d, J = 8.5 Hz, 1H), 0.82 (s, 3H).

^{13}C NMR (151 MHz, $CDCl_3$, 298 K, δ): 144.7, 124.5 (q, J = 273.5 Hz), 118.5, 66.2 (d, J = 2.2 Hz), 65.4, 50.7 (q, J = 1.6 Hz), 45.9, 40.9, 38.2, 37.0, 31.8, 31.5, 31.2 (q, J = 39.7 Hz), 26.5, 21.4.

^{19}F NMR (565 MHz, $CDCl_3$, 298 K, δ): –70.15 (s).

HRMS-CI (m/z) calc'd for $C_{17}H_{24}OF_3^+$ [M+H]$^+$, 301.1774; found, 301.1775; deviation: –0.4 ppm.

Bicyclo[1.1.1]pentylether 5

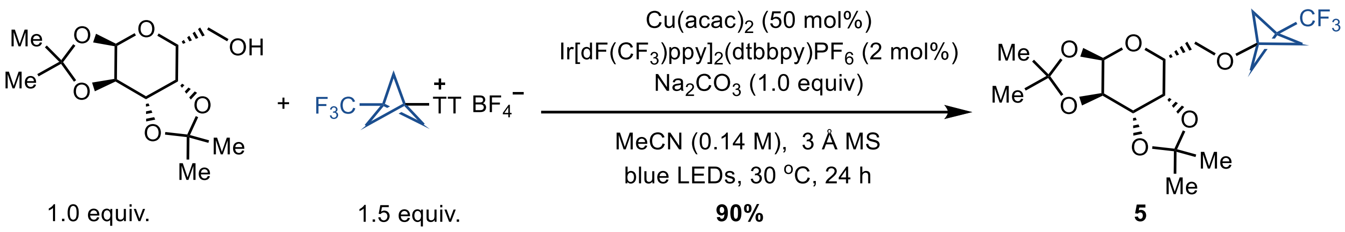

Under nitrogen atmosphere, to a 4 mL borosilicate vial equipped with a magnetic stir bar were added diaceton-*D*-galactose (26.0 mg, 0.100 mmol, 1.00 equiv.), CF_3BCP-TT$^+$ BF_4^- (65.7 mg, 0.150 mmol, 1.50 equiv.), Ir[dF(CF_3)ppy]$_2$(dtbbpy)PF_6 (2 mg, 2 µmoL, 2 mol%), Cu(acac)$_2$ (13 mg, 50 µmoL, 50 mol%), Na_2CO_3 (10.6 mg, 0.100 mmol, 1.00 equiv.), 3Å molecular sieves (120 mg), and anhydrous MeCN (0.70 mL, *c* = 0.14 M). The vial was sealed with a septum-cap. Then, the mixture was stirred for 1 h at 25 °C, and placed 5 cm away from two blue LEDs (Kessil A160WE Tuna Blue (460 nm), LED lighting, 40 W). The mixture was irradiated for 24 h while maintaining the temperature at approximately 30 °C through cooling with a fan. After irradiation, the mixture was concentrated under reduced pressure. The residue was purified by flash column chromatography on silica gel eluting with EtOAc/pentane (0:100–1:20 (v/v)) to afford the title compound **5** as a colorless oil (35.5 mg, 90.0 µmol, 90%).

R_f = 0.30 (pentane/EtOAc, 10:1 (v/v)).

NMR Spectroscopy:

^{1}H NMR (500 MHz, $CDCl_3$, 298 K, δ): 5.53 (d, *J* = 5.0 Hz, 1H), 4.60 (dd, *J* = 7.9, 2.4 Hz, 1H), 4.31 (dd, *J* = 5.0, 2.4 Hz, 1H), 4.23 (dd, *J* = 7.9, 2.1 Hz, 1H), 4.07 – 3.86 (m, 1H), 3.65 (dd, *J* = 10.1, 5.8 Hz, 1H), 3.59 (dd, *J* = 10.1, 6.8 Hz, 1H), 2.13 (s, 6H), 1.53 (s, 3H), 1.44 (s, 3H), 1.33 (d, *J* = 6.0 Hz, 6H).

^{13}C NMR (126 MHz, $CDCl_3$, 298 K, δ): 124.5 (q, *J* = 273.0 Hz), 109.5, 108.8, 96.5, 71.1, 70.8, 70.6, 66.7, 66.6 (d, *J* = 2.4 Hz), 65.9, 50.8 (d, *J* = 1.3 Hz), 31.1 (q, *J* = 39.9 Hz), 26.2, 26.1, 25.0, 24.6.

^{19}F NMR (565 MHz, $CDCl_3$, 298 K, δ): –70.06 (s).

HRMS-ESI (m/z) calc'd for $C_{18}H_{25}O_6F_3Na^+$ $[M+Na]^+$, 417.1495; found, 417.1497; deviation: –0.3 ppm.

Bicyclo[1.1.1]pentylether 6

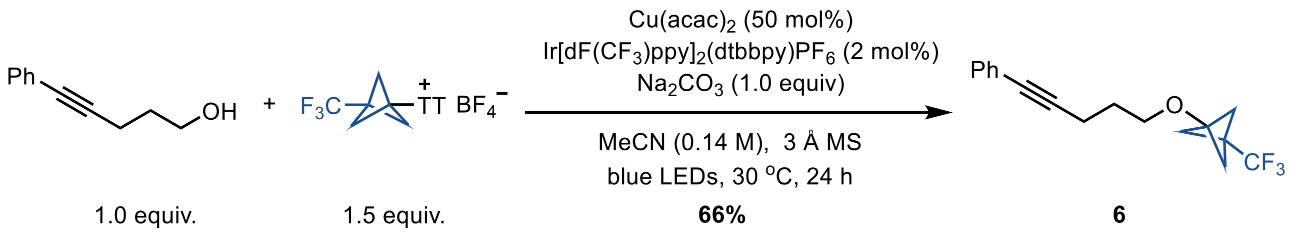

Under nitrogen atmosphere, to a 4 mL borosilicate vial equipped with a magnetic stir bar were added phenylpentynol (16.0 mg, 0.100 mmol, 1.00 equiv.), CF_3BCP-TT$^+$ BF_4^- (65.7 mg, 0.150 mmol, 1.50 equiv.),

Ir[dF(CF_3)ppy]$_2$(dtbbpy)PF_6 (2 mg, 2 µmoL, 2 mol%), Cu(acac)$_2$ (13 mg, 50 µmoL, 50 mol%), Na_2CO_3 (10.6 mg, 0.100 mmol, 1.00 equiv.), 3Å molecular sieves (120 mg), and anhydrous MeCN (0.70 mL, *c* = 0.14 M). The vial was sealed with a septum-cap. Then, the mixture was stirred for 1 h at 25 °C, and placed 5 cm away from two blue LEDs (Kessil A160WE Tuna Blue (460 nm), LED lighting, 40 W). The mixture was irradiated for 24 h while maintaining the temperature at approximately 30 °C through cooling with a fan. After irradiation, the mixture was concentrated under reduced pressure. The residue was purified by flash column chromatography on silica gel eluting with EtOAc/pentane (0:100–1:50 (v/v)) to afford the title compound **6** as a colorless oil (19.4 mg, 66.0 µmol, 66%).

R_f = 0.65 (pentane/EtOAc, 20:1 (v/v)).

NMR Spectroscopy:

^{1}H NMR (500 MHz, $CDCl_3$, 298 K, δ): 7.41 – 7.35 (m, 2H), 7.33 – 7.27 (m, 3H), 3.62 (t, *J* = 6.3 Hz, 2H), 2.52 (t, *J* = 6.9 Hz, 2H), 2.14 (s, 6H), 1.87 (p, *J* = 6.5 Hz, 2H).

^{13}C NMR (126 MHz, $CDCl_3$, 298 K, δ): 131.7, 128.4, 127.8, 124.5 (q, *J* = 273.6 Hz), 123.9, 89.1, 81.3, 66.3 (d, *J* = 3.0 Hz), 65.4, 50.7 (d, *J* = 1.5 Hz), 31.1 (q, *J* = 39.6 Hz), 28.7, 16.2.

^{19}F NMR (471 MHz, $CDCl_3$, 298 K, δ): –70.09 (s).

HRMS-APPI (m/z) calc'd for $C_{17}H_{18}OF_3^+$ [M+H]$^+$, 295.1304; found, 295.1304; deviation: +0.0 ppm.

Bicyclo[1.1.1]pentylether 7

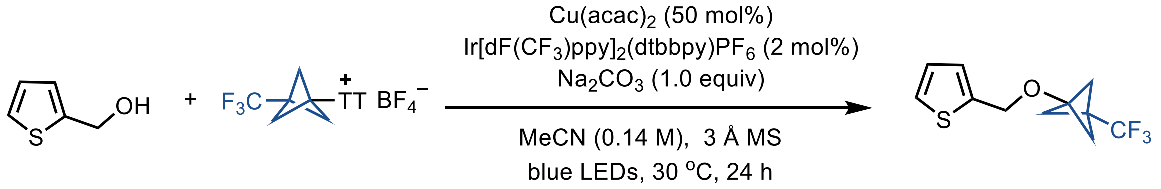

1.0 equiv. 1.5 equiv. **70%** **7**

Under nitrogen atmosphere, to a 4 mL borosilicate vial equipped with a magnetic stir bar were added 2-thiophenmethanol (11.4 mg, 0.100 mmol, 1.00 equiv.), CF_3BCP-TT$^+$ BF_4^- (65.7 mg, 0.150 mmol, 1.50 equiv.), Ir[dF(CF_3)ppy]$_2$(dtbbpy)PF_6 (2 mg, 2 µmoL, 2 mol%), Cu(acac)$_2$ (13 mg, 50 µmoL, 50 mol%), Na_2CO_3 (10.6 mg, 0.100 mmol, 1.00 equiv.), 3Å molecular sieves (120 mg), and anhydrous MeCN (0.70 mL, *c* = 0.14 M). The vial was sealed with a septum-cap. Then, the mixture was stirred for 1 h at 25 °C, and placed 5 cm away from two blue LEDs (Kessil A160WE Tuna Blue (460 nm), LED lighting, 40 W). The mixture was irradiated for 24 h while maintaining the temperature at approximately 30 °C through cooling with a fan. After irradiation, the mixture was concentrated under reduced pressure. The residue was purified by flash column chromatography on silica gel eluting with EtOAc/pentane (0:100–1:50 (v/v)) to afford the title compound **7** as a colorless oil (17.4 mg, 70.0 µmol, 70%).

R_f = 0.46 (pentane/EtOAc, 20:1 (v/v)).

NMR Spectroscopy:

^{1}H NMR (500 MHz, $CDCl_3$, 298 K, δ): 7.30 (dd, *J* = 5.0, 1.2 Hz, 1H), 7.01 (d, *J* = 3.4 Hz, 1H), 6.97 (dd, *J* = 5.0, 3.5 Hz, 1H), 4.71 (s, 2H), 2.14 (s, 6H).

^{13}C NMR (126 MHz, $CDCl_3$, 298 K, δ): 140.3, 126.9, 126.7, 126.3, 124.3 (q, *J* = 273.6 Hz), 66.4 (d, *J* = 3.0 Hz), 64.2, 51.1 (d, *J* = 1.4 Hz), 31.3 (q, *J* = 39.8 Hz).

^{19}F NMR (471 MHz, $CDCl_3$, 298 K, δ): –70.07 (s).

HRMS-CI (m/z) calc'd for $C_{11}H_{12}F_3OS^+$ $[M+H]^+$, 249.0555; found, 249.0557; deviation: –0.8 ppm.

Bicyclo[1.1.1]pentylether 8

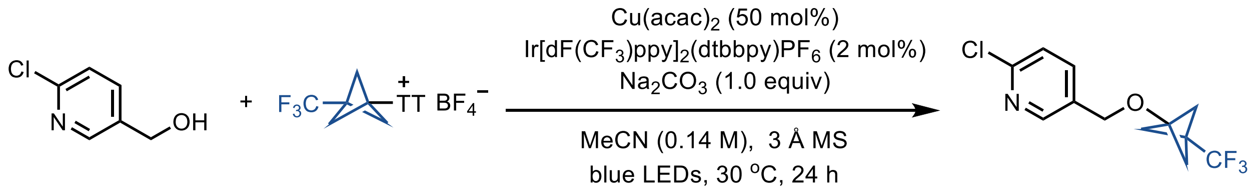

1.0 equiv. 1.5 equiv. **69%** **8**

Under nitrogen atmosphere, to a 4 mL borosilicate vial equipped with a magnetic stir bar were added 2-chloro-5-hydroxymethylpyridine (14.3 mg, 0.100 mmol, 1.00 equiv.), CF_3BCP-TT^+ BF_4^- (65.7 mg, 0.150 mmol, 1.50 equiv.), Ir[dF(CF_3)ppy]$_2$(dtbbpy)PF_6 (2 mg, 2 µmoL, 2 mol%), Cu(acac)$_2$ (13 mg, 50 µmoL, 50 mol%), Na_2CO_3 (10.6 mg, 0.100 mmol, 1.00 equiv.), 3Å molecular sieves (120 mg), and anhydrous MeCN (0.70 mL, *c* = 0.14 M). The vial was sealed with a septum-cap. Then, the mixture was stirred for 1 h at 25 °C, and placed 5 cm away from two blue LEDs (Kessil A160WE Tuna Blue (460 nm), LED lighting, 40 W). The mixture was irradiated for 24 h while maintaining the temperature at approximately 30 °C through cooling with a fan. After irradiation, the mixture was concentrated under reduced pressure. The residue was purified by flash column chromatography on silica gel eluting with EtOAc/pentane (0:100–1:15 (v/v)) to afford the title compound **8** as a colorless oil (19.2 mg, 69.0 µmol, 69%).

R_f = 0.30 (pentane/EtOAc, 10:1 (v/v)).

NMR Spectroscopy:

^{1}H NMR (500 MHz, $CDCl_3$, 298 K, δ): 8.34 (d, *J* = 2.6 Hz, 1H), 7.65 (dd, *J* = 8.2, 2.4 Hz, 1H), 7.32 (d, *J* = 8.2 Hz, 1H), 4.53 (s, 2H), 2.14 (s, 6H).

^{13}C NMR (126 MHz, $CDCl_3$, 298 K, δ): 151.2, 148.7, 138.1, 132.1, 124.3, 124.2 (d, *J* = 273.0 Hz), 66.6 (d, *J* = 3.0 Hz), 65.9, 50.9 (d, *J* = 1.5 Hz), 31.2 (q, *J* = 37.8 Hz).

^{19}F NMR (565 MHz, $CDCl_3$, 298 K, δ): –70.10 (s).

HRMS-ESI (m/z) calc'd for $C_{12}H_{12}NOF_3Cl^+$ $[M+H]^+$, 278.0554; found, 278.0555; deviation: –0.5 ppm.

Bicyclo[1.1.1]pentylether 9

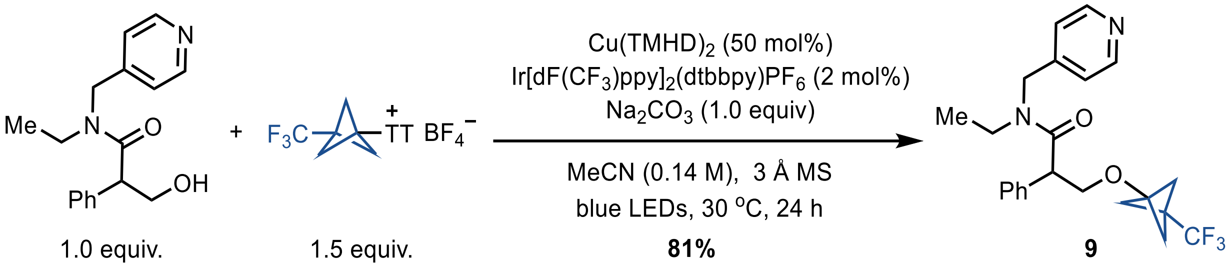

Under nitrogen atmosphere, to a 4 mL borosilicate vial equipped with a magnetic stir bar were added tropicamide (28.4 mg, 0.100 mmol, 1.00 equiv.), $CF_3BCP\text{-}TT^+ BF_4^-$ (65.7 mg, 0.150 mmol, 1.50 equiv.), Ir[dF(CF_3)ppy]$_2$(dtbbpy)PF_6 (2 mg, 2 µmoL, 2 mol%), Cu(TMHD)$_2$ (22 mg, 50 µmoL, 50 mol%), Na_2CO_3 (10.6 mg, 0.100 mmol, 1.00 equiv.), 3Å molecular sieves (120 mg), and anhydrous MeCN (0.70 mL, *c* = 0.14 M). The vial was sealed with a septum-cap. Then, the mixture was stirred for 1 h at 25 °C, and placed 5 cm away from two blue LEDs (Kessil A160WE Tuna Blue (460 nm), LED lighting, 40 W). The mixture was irradiated for 24 h while maintaining the temperature at approximately 30 °C through cooling with a fan. After irradiation, the mixture was concentrated under reduced pressure. The residue was purified by flash column chromatography on silica gel eluting with EtOAc/pentane (0:100–1:2 (v/v)) to afford the title compound **9** as a colorless oil (33.9 mg, 81.0 µmol, 81%, mixture of rotamers).

R_f = 0.21 (pentane/EtOAc, 1:1 (v/v)).

NMR Spectroscopy:

^{1}H NMR (600 MHz, CD_3CN, 298 K, δ): 8.53 (s, 1.9H), 7.60 (s, 0.5H), 7.46 – 7.35 (m, 2.7H), 7.34 – 7.24 (m, 2.2H), 7.11 (s, 1.9H), 4.67 – 4.54 (m, 1.0H), 4.55 – 4.38 (m, 1.1H), 4.22 (dd, *J* = 8.8, 5.5 Hz, 0.7H), 4.18 – 4.06 (m, 1.0H), 3.96 – 3.86 (m, 0.4H), 3.63 (dd, *J* = 9.1, 5.5 Hz, 0.7H), 3.59 – 3.51 (m, 0.6H), 3.45 – 3.37 (m, 0.7H), 3.35 – 3.25 (m, 0.7H), 3.20 (d, *J* = 7.1 Hz, 0.4H), 2.12 (s, 4.1H), 2.05 (s, 1.9H), 1.02 (t, *J* = 7.1 Hz, 3H).

^{13}C NMR (151 MHz, CD_3CN, 298 K, δ): 172.2, 171.9, 150.7, 150.5, 148.6, 148.0, 137.7, 137.4, 129.8, 129.4, 129.2, 128.6, 128.6, 127.2 – 124.5 (m), 123.5, 123.1, 123.0, 79.2, 70.5, 70.4, 66.9 (q, *J* = 2.5 Hz), 66.8 (q, *J* = 2.5 Hz), 51.2 (q, *J* = 1.6 Hz), 51.2 (d, *J* = 1.9 Hz), 50.1, 49.5, 49.1, 48.5, 43.5, 42.2, 32.3 – 31.1 (m), 14.6, 12.8.

^{19}F NMR (565 MHz, $CDCl_3$, 298 K, δ): –70.63 (s), –70.66 (s).

HRMS-ESI (m/z) calc'd for $C_{23}H_{26}N_2O_2F_3^+$ [M+H]$^+$, 419.1941; found, 419.1940; deviation: +0.2 ppm.

Bicyclo[1.1.1]pentylether 10

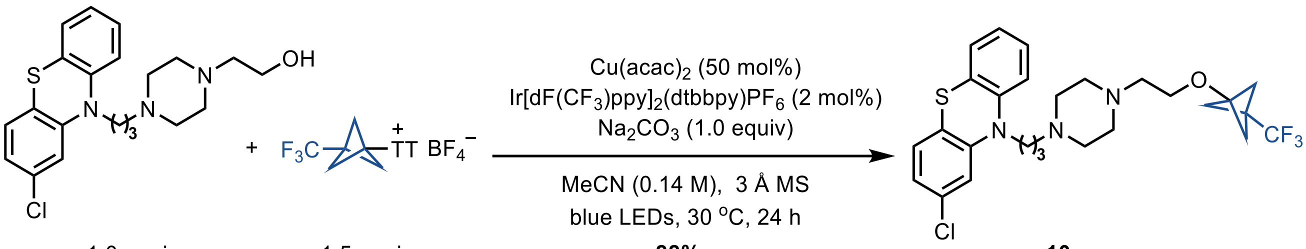

1.0 equiv. 1.5 equiv. **88%** **10**

Under nitrogen atmosphere, to a 4 mL borosilicate vial equipped with a magnetic stir bar were added perphenazin (40.3 mg, 0.100 mmol, 1.00 equiv.), CF_3BCP-TT$^+$ BF_4^- (65.7 mg, 0.150 mmol, 1.50 equiv.), Ir[dF(CF_3)ppy]$_2$(dtbbpy)PF_6 (2 mg, 2 µmoL, 2 mol%), Cu(acac)$_2$ (13 mg, 50 µmoL, 50 mol%), Na_2CO_3 (10.6 mg, 0.100 mmol, 1.00 equiv.), 3Å molecular sieves (120 mg), and anhydrous MeCN (0.70 mL, *c* = 0.14 M). The vial was sealed with a septum-cap. Then, the mixture was stirred for 1 h at 25 °C, and placed 5 cm away from two blue LEDs (Kessil A160WE Tuna Blue (460 nm), LED lighting, 40 W). The mixture was irradiated for 24 h while maintaining the temperature at approximately 30 °C through cooling with a fan. After irradiation, the mixture was concentrated under reduced pressure. The residue was purified by flash column chromatography on silica gel eluting with DCM/MeOH (1:0–200:1 (v/v), with 1% Et_3N) to afford the title compound **10** as a colorless oil (47.4 mg, 88.0 µmol, 88%).

R_f = 0.35 (DCM/MeOH, 10:1 (v/v)).

NMR Spectroscopy:

^{1}H NMR (500 MHz, $CDCl_3$, 298 K, δ): 7.19 – 7.12 (m, 1H), 7.11 (dd, *J* = 7.6, 1.5 Hz, 1H), 7.01 (d, *J* = 8.1 Hz, 1H), 6.92 (t, *J* = 6.9 Hz, 1H), 6.91 – 6.85 (m, 2H), 6.84 (d, *J* = 2.1 Hz, 1H), 3.89 (t, *J* = 6.9 Hz, 2H), 3.56 (t, *J* = 6.0 Hz, 2H), 2.57 (t, *J* = 6.0 Hz, 2H), 2.47 (t, *J* = 7.1 Hz, 10H), 2.11 (s, 6H), 2.02 – 1.88 (m, 2H).

^{13}C NMR (151 MHz, $CDCl_3$, 298 K, δ): 146.6, 144.6, 133.4, 128.0, 127.7, 127.6, 125.0, 124.4 (q, *J* = 273.7 Hz), 123.7, 123.1, 122.4, 116.0, 116.0, 66.4 (d, *J* = 2.5 Hz), 64.5, 57.6, 55.6, 53.6, 53.2, 50.7 (q, *J* = 1.9 Hz), 45.5, 31.1 (q, *J* = 39.7 Hz), 24.3.

^{19}F NMR (471 MHz, $CDCl_3$, 298 K, δ): –70.09 (s).

HRMS-ESI (m/z) calc'd for $C_{27}H_{32}N_3OClSF_3^+$ [M+H]$^+$, 538.1901; found, 538.1900; deviation: +0.2 ppm.

Bicyclo[1.1.1]pentylether 11

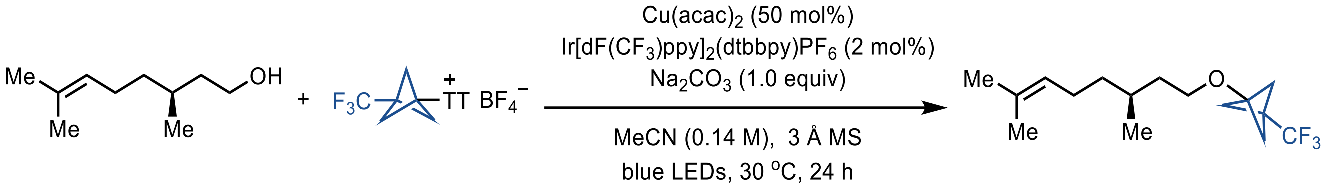

1.0 equiv. 1.5 equiv. **89%** **11**

Under nitrogen atmosphere, to a 4 mL borosilicate vial equipped with a magnetic stir bar were added (-)-*β*-citronellol (15.6 mg, 0.100 mmol, 1.00 equiv.), CF_3BCP-TT^+ BF_4^- (65.7 mg, 0.150 mmol, 1.50 equiv.), Ir[dF(CF_3)ppy]$_2$(dtbbpy)PF_6 (2 mg, 2 µmoL, 2 mol%), Cu(acac)$_2$ (13 mg, 50 µmoL, 50 mol%), Na_2CO_3 (10.6 mg, 0.100 mmol, 1.00 equiv.), 3Å molecular sieves (120 mg), and anhydrous MeCN (0.70 mL, *c* = 0.14 M). The vial was sealed with a septum-cap. Then, the mixture was stirred for 1 h at 25 °C, and placed 5 cm away from two blue LEDs (Kessil A160WE Tuna Blue (460 nm), LED lighting, 40 W). The mixture was irradiated for 24 h while maintaining the temperature at approximately 30 °C through cooling with a fan. After irradiation, the mixture was concentrated under reduced pressure. The residue was purified by flash column chromatography on silica gel eluting with EtOAc/pentane (0:100–1:40 (v/v)) to afford the title compound **11** as a colorless oil (25.8 mg, 89.0 µmol, 89%).

R*f* = 0.46 (pentane/EtOAc, 20:1 (v/v)).

NMR Spectroscopy:

^{1}H NMR (500 MHz, $CDCl_3$, 298 K, δ): 5.17 – 5.03 (m, 1H), 3.70 – 3.32 (m, 2H), 2.11 (s, 6H), 1.97 (td, *J* = 15.7, 6.9 Hz, 2H), 1.68 (s, 3H), 1.60 (s, 4H), 1.59 – 1.49 (m, 1H), 1.44 – 1.36 (m, 1H), 1.36 – 1.28 (m, 1H), 1.22 – 1.10 (m, 1H), 0.89 (d, *J* = 6.7 Hz, 3H).

^{13}C NMR (126 MHz, $CDCl_3$, 298 K, δ): 131.5, 124.8, 124.5 (d, *J* = 273.6 Hz), 66.3 (d, *J* = 7.7 Hz), 65.3, 50.7 (d, *J* = 1.5 Hz), 37.3, 36.6, 31.2 (q, *J* = 39.9 Hz), 29.6, 25.9, 25.6, 19.6, 17.8.

^{19}F NMR (471 MHz, $CDCl_3$, 298 K, δ): –70.15 (s).

HRMS-APPI (m/z) calc'd for $C_{16}H_{26}OF_3^+$ $[M+H]^+$, 291.1930; found, 291.1928; deviation: +0.7 ppm.

Bicyclo[1.1.1]pentylether 12

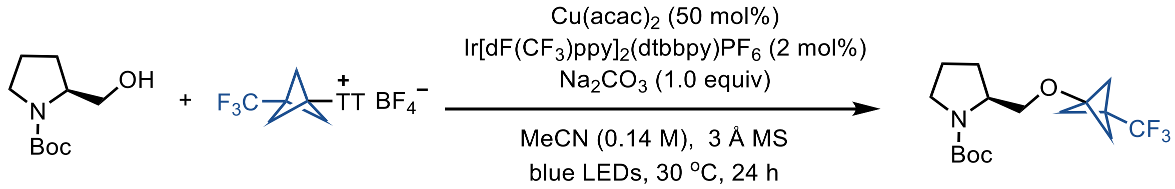

1.0 equiv. 1.5 equiv. **84%** **12**

Under nitrogen atmosphere, to a 4 mL borosilicate vial equipped with a magnetic stir bar were added *N*-Boc-*L*-prolinol (20.1 mg, 0.100 mmol, 1.00 equiv.), CF_3BCP-TT^+ BF_4^- (65.7 mg, 0.150 mmol, 1.50 equiv.),

Ir[dF(CF_3)ppy]$_2$(dtbbpy)PF_6 (2 mg, 2 µmoL, 2 mol%), Cu(acac)$_2$ (13 mg, 50 µmoL, 50 mol%), Na_2CO_3 (10.6 mg, 0.100 mmol, 1.00 equiv.), 3Å molecular sieves (120 mg), and anhydrous MeCN (0.70 mL, *c* = 0.14 M). The vial was sealed with a septum-cap. Then, the mixture was stirred for 1 h at 25 °C, and placed 5 cm away from two blue LEDs (Kessil A160WE Tuna Blue (460 nm), LED lighting, 40 W). The mixture was irradiated for 24 h while maintaining the temperature at approximately 30 °C through cooling with a fan. After irradiation, the mixture was concentrated under reduced pressure. The residue was purified by flash column chromatography on silica gel eluting with EtOAc/pentane (0:100–1:12 (v/v)) to afford the title compound **12** as a colorless oil (28.2 mg, 84.0 µmol, 84%).

R_f = 0.30 (pentane/EtOAc, 10:1 (v/v)).

NMR Spectroscopy:

^{1}H NMR (500 MHz, $CDCl_3$, 298 K, δ): 3.87 (d, *J* = 33.0 Hz, 1H), 3.59 (d, *J* = 19.5 Hz, 1H), 3.50 – 3.20 (m, 3H), 2.09 (s, 6H), 1.96 – 1.73 (m, 4H), 1.46 (s, 9H).

^{13}C NMR (151 MHz, $CDCl_3$, 298 K, δ): 154.7 (d, *J* = 25.3 Hz), 124.5 (q, *J* = 277.5 Hz), 79.5 (d, *J* = 31.2 Hz), 67.4 (d, *J* = 68.7 Hz), 66.4, 56.5, 50.8, 46.9 (d, *J* = 67.8 Hz), 31.0 (q, *J* = 40.3 Hz), 28.7, 28.4 (d, *J* = 120.1 Hz), 23.5 (d, *J* = 138.8 Hz).

^{19}F NMR (565 MHz, $CDCl_3$, 298 K, δ): –70.09 (d, *J* = 26.9 Hz).

HRMS-ESI (m/z) calc'd for $C_{16}H_{24}NO_3F_3Na^+$ [M+Na]$^+$, 358.1600; found, 358.1600; deviation: –0.0 ppm.

Bicyclo[1.1.1]pentylether 13

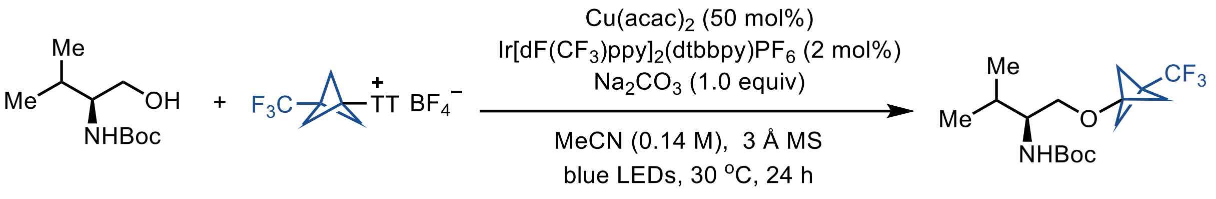

1.0 equiv. 1.5 equiv. **90%** **13**

Under nitrogen atmosphere, to a 4 mL borosilicate vial equipped with a magnetic stir bar were added *N*-Boc-*L*-valinol (20.3 mg, 0.100 mmol, 1.00 equiv.), CF_3BCP-TT$^+$ BF_4^- (65.7 mg, 0.150 mmol, 1.50 equiv.), Ir[dF(CF_3)ppy]$_2$(dtbbpy)PF_6 (2 mg, 2 µmoL, 2 mol%), Cu(acac)$_2$ (13 mg, 50 µmoL, 50 mol%), Na_2CO_3 (10.6 mg, 0.100 mmol, 1.00 equiv.), 3Å molecular sieves (120 mg), and anhydrous MeCN (0.70 mL, *c* = 0.14 M). The vial was sealed with a septum-cap. Then, the mixture was stirred for 1 h at 25 °C, and placed 5 cm away from two blue LEDs (Kessil A160WE Tuna Blue (460 nm), LED lighting, 40 W). The mixture was irradiated for 24 h while maintaining the temperature at approximately 30 °C through cooling with a fan. After irradiation, the mixture was concentrated under reduced pressure. The residue was purified by flash column chromatography on silica gel eluting with EtOAc/pentane (0:100–1:20 (v/v)) to afford the title compound **13** as a colorless oil (30.4 mg, 90.0 µmol, 90%).

R$_f$ = 0.33 (pentane/EtOAc, 10:1 (v/v)).

NMR Spectroscopy:

^{1}H NMR (500 MHz, $CDCl_3$, 298 K, δ): 4.63 (s, 1H), 3.63 – 3.34 (m, 3H), 2.09 (s, 6H), 1.83 (q, J = 6.9 Hz, 1H), 1.44 (s, 9H), 0.91 (dd, J = 6.9, 5.0 Hz, 6H).

^{13}C NMR (151 MHz, $CDCl_3$, 298 K, δ): 155.9, 124.4 (q, J = 273.4 Hz), 79.3, 67.0, 66.4 (q, J = 2.5 Hz), 55.3, 50.6 (q, J = 1.9 Hz), 31.1 (q, J = 39.9 Hz), 29.4, 28.5, 19.7, 18.7.

^{19}F NMR (565 MHz, $CDCl_3$, 298 K, δ): –70.15 (s).

HRMS-ESI (m/z) calc'd for $C_{16}H_{26}NO_3F_3Na^+$ $[M+Na]^+$, 360.1757; found, 360.1761; deviation: –1.1 ppm.

Bicyclo[1.1.1]pentylether 14

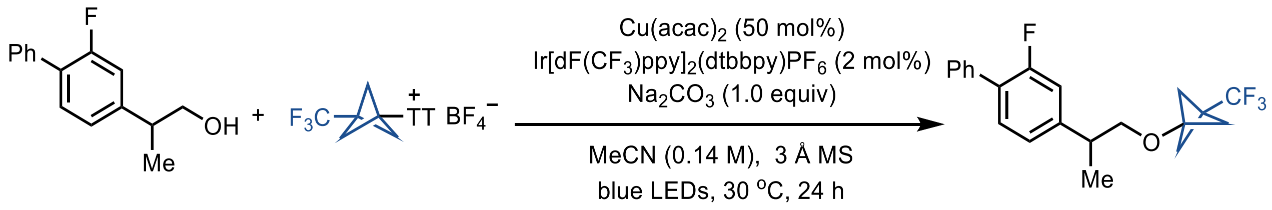

1.0 equiv. 1.5 equiv. **78%** **14**

Under nitrogen atmosphere, to a 4 mL borosilicate vial equipped with a magnetic stir bar were added flurbiprofen derivative (23.0 mg, 0.100 mmol, 1.00 equiv.), CF_3BCP-TT^+ BF_4^- (65.7 mg, 0.150 mmol, 1.50 equiv.), Ir[dF(CF_3)ppy]$_2$(dtbbpy)PF_6 (2 mg, 2 µmoL, 2 mol%), Cu(acac)$_2$ (13 mg, 50 µmoL, 50 mol%), Na_2CO_3 (10.6 mg, 0.100 mmol, 1.00 equiv.), 3Å molecular sieves (120 mg), and anhydrous MeCN (0.70 mL, c = 0.14 M). The vial was sealed with a septum-cap. Then, the mixture was stirred for 1 h at 25 °C, and placed 5 cm away from two blue LEDs (Kessil A160WE Tuna Blue (460 nm), LED lighting, 40 W). The mixture was irradiated for 24 h while maintaining the temperature at approximately 30 °C through cooling with a fan. After irradiation, the mixture was concentrated under reduced pressure. The residue was purified by flash column chromatography on silica gel eluting with EtOAc/pentane (0:100–1:50 (v/v)) to afford the title compound **14** as a colorless oil (28.4 mg, 78.0 µmol, 78%).

R$_f$ = 0.53 (pentane/EtOAc, 20:1 (v/v)).

NMR Spectroscopy:

^{1}H NMR (500 MHz, $CDCl_3$, 298 K, δ): 7.55 (d, J = 7.8 Hz, 2H), 7.45 (t, J = 7.7 Hz, 2H), 7.41 – 7.32 (m, 2H), 7.08 (d, J = 7.9 Hz, 1H), 7.03 (d, J = 11.9 Hz, 1H), 3.68 – 3.57 (m, 1H), 3.51 (t, J = 8.2 Hz, 1H), 3.04 (q, J = 6.9 Hz, 1H), 2.11 (s, 6H), 1.33 (d, J = 7.0 Hz, 3H).

^{13}C NMR (126 MHz, $CDCl_3$, 298 K, δ): 159.9 (d, J = 247.8 Hz), 145.6 (d, J = 7.3 Hz), 124.5 (d, J = 273.5 Hz), 135.8, 130.8 (d, J = 3.9 Hz), 129.1 (d, J = 2.9 Hz), 128.6, 127.7, 127.3 (d, J = 13.6 Hz), 123.5 (d, J = 3.2 Hz), 115.0 (d, J = 22.9 Hz), 72.2, 66.4 (d, J = 3.0 Hz), 50.7 (d, J = 1.5 Hz), 39.6, 31.2 (q, J = 39.6

Hz), 18.3.

19F NMR (471 MHz, $CDCl_3$, 298 K, δ): –70.08 (s, 3H), –118.13 (dd, *J* = 11.7, 8.2 Hz, 1H).

HRMS-ESI (m/z) calc'd for $C_{21}H_{20}OF_4Na^+$ $[M+Na]^+$, 387.1342; found, 387.1344; deviation: –0.3 ppm.

Bicyclo[1.1.1]pentylether 15

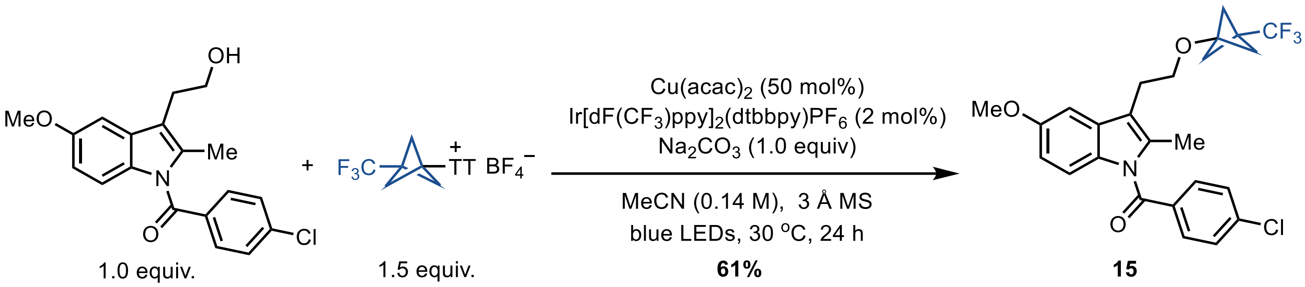

Under nitrogen atmosphere, to a 4 mL borosilicate vial equipped with a magnetic stir bar were added indomethacin derivative (34.3 mg, 0.100 mmol, 1.00 equiv.), CF_3BCP-TT$^+$ BF_4^- (65.7 mg, 0.150 mmol, 1.50 equiv.), Ir[dF(CF_3)ppy]$_2$(dtbbpy)PF_6 (2 mg, 2 µmoL, 2 mol%), Cu(acac)$_2$ (13 mg, 50 µmoL, 50 mol%), Na_2CO_3 (10.6 mg, 0.100 mmol, 1.00 equiv.), 3Å molecular sieves (120 mg), and anhydrous MeCN (0.70 mL, *c* = 0.14 M). The vial was sealed with a septum-cap. Then, the mixture was stirred for 1 h at 25 °C, and placed 5 cm away from two blue LEDs (Kessil A160WE Tuna Blue (460 nm), LED lighting, 40 W). The mixture was irradiated for 24 h while maintaining the temperature at approximately 30 °C through cooling with a fan. After irradiation, the mixture was concentrated under reduced pressure. The residue was purified by flash column chromatography on silica gel eluting with EtOAc/pentane (0:100–1:20 (v/v)) to afford the title compound **15** as a colorless oil (29.2 mg, 61.0 µmol, 61%).

R_f = 0.3 (pentane/EtOAc, 10:1 (v/v)).

NMR Spectroscopy:

1H NMR (600 MHz, $CDCl_3$, 298 K, δ): 7.65 (d, *J* = 8.8 Hz, 2H), 7.47 (d, *J* = 8.7 Hz, 2H), 6.93 (dd, *J* = 2.6, 0.4 Hz, 1H), 6.86 (dd, *J* = 9.0, 0.5 Hz, 1H), 6.67 (dd, *J* = 9.0, 2.6 Hz, 1H), 3.84 (s, 3H), 3.67 – 3.51 (m, 2H), 2.95 (t, *J* = 7.5 Hz, 2H), 2.36 (s, 3H), 2.10 (s, 6H).

13C NMR (151 MHz, $CDCl_3$, 298 K, δ): 168.4, 156.1, 139.3, 135.5, 134.2, 131.3, 131.1(3), 131.1(0), 129.3, 124.4 (q, *J* = 273.7 Hz), 115.8, 115.2, 111.3, 101.5, 66.3 (q, *J* = 2.5 Hz), 66.2, 55.9, 50.7 (d, *J* = 1.9 Hz), 31.1 (q, *J* = 39.7 Hz), 25.0, 13.5.

19F NMR (565 MHz, $CDCl_3$, 298 K, δ): –70.10 (s).

HRMS-ESI (m/z) calc'd for $C_{25}H_{23}NO_3F_3ClNa^+$ $[M+Na]^+$, 500.1211; found, 500.1214; deviation: –0.7 ppm.

Bicyclo[1.1.1]pentylether 16

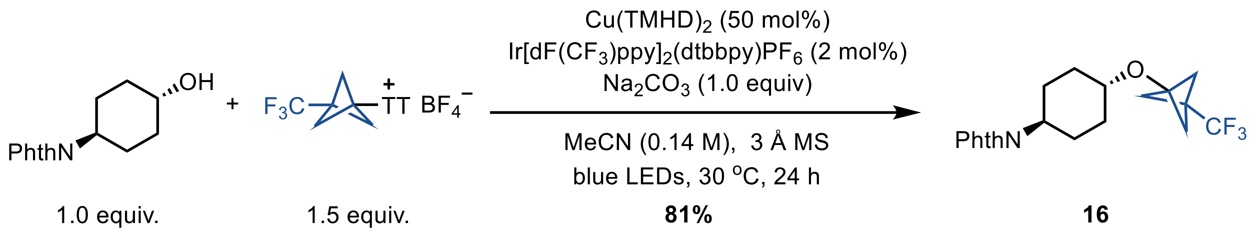

16

Under nitrogen atmosphere, to a 4 mL borosilicate vial equipped with a magnetic stir bar were added trans-2-(4-hydroxycyclohexyl)-1H-isoindole-1,3(2H)-dione (24.5 mg, 0.100 mmol, 1.00 equiv.), CF_3BCP-TT$^+$ BF_4^- (65.7 mg, 0.150 mmol, 1.50 equiv.), Ir[dF(CF_3)ppy]$_2$(dtbbpy)PF_6 (2 mg, 2 µmoL, 2 mol%), Cu(TMHD)$_2$ (22 mg, 50 µmoL, 50 mol%), Na_2CO_3 (10.6 mg, 0.100 mmol, 1.00 equiv.), 3Å molecular sieves (120 mg), and anhydrous MeCN (0.70 mL, *c* = 0.14 M). The vial was sealed with a septum-cap. Then, the mixture was stirred for 1 h at 25 °C, and placed 5 cm away from two blue LEDs (Kessil A160WE Tuna Blue (460 nm), LED lighting, 40 W). The mixture was irradiated for 24 h while maintaining the temperature at approximately 30 °C through cooling with a fan. After irradiation, the mixture was concentrated under reduced pressure. The residue was purified by flash column chromatography on silica gel eluting with EtOAc/pentane (0:100–1:12 (v/v)) to afford the title compound **16** as a colorless solid (30.7 mg, 81.0 µmol, 81%).

$\mathbf{R}_f$ = 0.20 (pentane/EtOAc, 10:1 (v/v)).

NMR Spectroscopy:

^{1}**H NMR** (500 MHz, $CDCl_3$, 298 K, δ): 7.81 (dd, *J* = 5.5, 3.1 Hz, 2H), 7.70 (dd, *J* = 5.5, 3.1 Hz, 2H), 4.22 – 3.99 (m, 1H), 3.62 – 3.52 (m, 1H), 2.32 (qd, *J* = 14.1, 3.6 Hz, 2H), 2.15 (s, 6H), 2.06 (d, *J* = 13.3 Hz, 2H), 1.77 (d, *J* = 13.0 Hz, 2H), 1.51 – 1.39 (m, 2H).

^{13}**C NMR** (126 MHz, $CDCl_3$, 298 K, δ): 168.4, 134.1, 132.1, 124.4 (q, *J* = 273.5 Hz), 123.3, 74.6, 65.6, 65.5 (d, *J* = 2.4 Hz), 51.8 (d, *J* = 1.4 Hz), 49.6, 32.5, 31.5 (q, *J* = 39.3 Hz), 27.7.

^{19}**F NMR** (471 MHz, $CDCl_3$, 298 K, δ): –70.05 (s).

HRMS-ESI (m/z) calc'd for $C_{20}H_{20}NO_3F_3Na^+$ [M+Na]$^+$, 402.1287; found, 402.1289; deviation: –0.4 ppm.

Bicyclo[1.1.1]pentylether 17

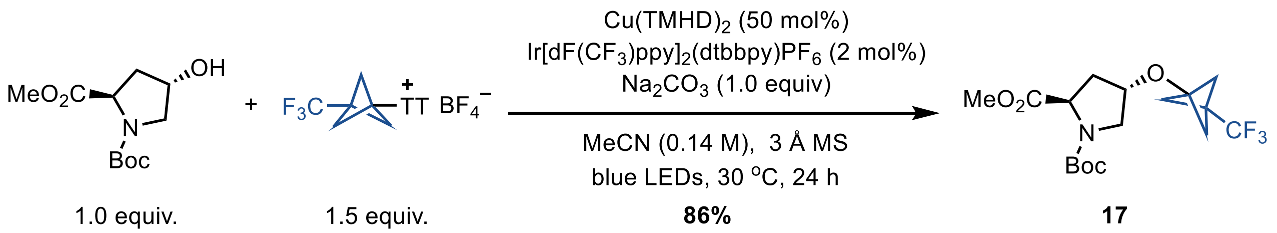

17

Under nitrogen atmosphere, to a 4 mL borosilicate vial equipped with a magnetic stir bar were added *N*-Boc-trans-4-hydroxy-L-prolin-methylester (24.5 mg, 0.100 mmol, 1.00 equiv.), CF_3BCP-TT$^+$ BF_4^- (65.7 mg, 0.150

mmol, 1.50 equiv.), Ir[dF(CF_3)ppy]$_2$(dtbbpy)PF_6 (2 mg, 2 µmoL, 2 mol%), Cu(TMHD)$_2$ (22 mg, 50 µmoL, 50 mol%), Na_2CO_3 (10.6 mg, 0.100 mmol, 1.00 equiv.), 3Å molecular sieves (120 mg), and anhydrous MeCN (0.70 mL, *c* = 0.14 M). The vial was sealed with a septum-cap. Then, the mixture was stirred for 1 h at 25 °C, and placed 5 cm away from two blue LEDs (Kessil A160WE Tuna Blue (460 nm), LED lighting, 40 W). The mixture was irradiated for 24 h while maintaining the temperature at approximately 30 °C through cooling with a fan. After irradiation, the mixture was concentrated under reduced pressure. The residue was purified by flash column chromatography on silica gel eluting with EtOAc/pentane (0:100–1:8 (v/v)) to afford the title compound **17** as a colorless oil (32.7 mg, 86.0 µmol, 86%).

R_f = 0.33 (pentane/EtOAc, 5:1 (v/v)).

NMR Spectroscopy:

^{1}H NMR (500 MHz, $CDCl_3$, 298 K, δ): 4.53 – 4.29 (m, 1H), 4.27 – 4.18 (m, 1H), 3.72 (d, *J* = 5.3 Hz, 3H), 3.65 (p, *J* = 5.9 Hz, 1H), 3.46 (dd, *J* = 41.0, 12.4 Hz, 1H), 2.34 – 2.20 (m, 1H), 2.16 – 2.04 (m, 7H), 1.42 (d, *J* = 25.2 Hz, 9H).

^{13}C NMR (126 MHz, $CDCl_3$, 298 K, δ):173.5, 173.3, 154.4, 153.7, 124.2 (q, *J* = 273.3 Hz), 80.5, 74.6, 73.8, 65.7, 57.9, 57.5, 52.4 (d, *J* = 12.5 Hz), 52.2 (d, *J* = 9.5 Hz), 51.4, 37.2, 36.2, 31.6 (q, *J* = 39.9 Hz), 28.5, 28.3.

^{19}F NMR (471 MHz, $CDCl_3$, 298 K, δ): –70.12 (d, *J* = 5.2 Hz).

HRMS-ESI (m/z) calc'd for $C_{17}H_{24}NO_5F_3Na^+$ [M+Na]$^+$, 402.1499; found, 402.1298; deviation: +0.2 ppm.

Bicyclo[1.1.1]pentylether 18

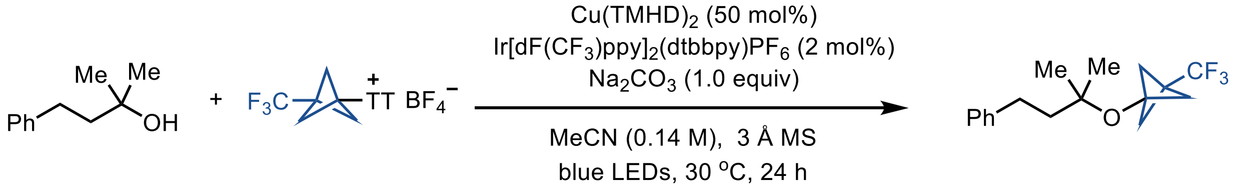

1.0 equiv. 1.5 equiv. **62%** **18**

Under nitrogen atmosphere, to a 4 mL borosilicate vial equipped with a magnetic stir bar were added CF_3BCP-TT$^+$ BF_4^- (65.7 mg, 0.150 mmol, 1.50 equiv.), Ir[dF(CF_3)ppy]$_2$(dtbbpy)PF_6 (2 mg, 2 µmoL, 2 mol%), Cu(TMHD)$_2$ (22 mg, 50 µmoL, 50 mol%), Na_2CO_3 (10.6 mg, 0.100 mmol, 1.00 equiv.), 3Å molecular sieves (120 mg), and anhydrous MeCN (0.70 mL, *c* = 0.14 M). The vial was sealed with a septum-cap, and transferred out of the glove box. Then, 2-phenethyl-2-propanol (16.9 µL, 16.4 mg, 0.100 mmol, 1.00 equiv.) was added to the vial. The mixture was stirred for 1 h at 25 °C, and placed 5 cm away from two blue LEDs (Kessil A160WE Tuna Blue (460 nm), LED lighting, 40 W). The mixture was irradiated for 24 h while maintaining the temperature at approximately 30 °C through cooling with a fan. After irradiation, the mixture was concentrated under reduced pressure. The residue was purified by flash column chromatography on

silica gel eluting with EtOAc/pentane (0:100–1:100 (v/v)), and subsequently by preparative TLC eluting with EtOAc/pentane (1:30 (v/v)) to afford the title compound **18** as a colorless oil (18.5 mg, 62.0 µmol, 62%).

R*f* = 0.45 (pentane/EtOAc, 20:1 (v/v)).

NMR Spectroscopy:

^{1}H NMR (500 MHz, $CDCl_3$, 298 K, δ): 7.33 – 7.27 (m, 2H), 7.22 – 7.16 (m, 3H), 2.71 – 2.63 (m, 2H), 2.23 (s, 6H), 1.85 – 1.78 (m, 2H), 1.31 (s, 6H).

^{13}C NMR (151 MHz, $CDCl_3$, 298 K, δ): 142.5, 128.6, 128.4, 126.0, 124.2 (q, *J* = 273.5 Hz), 78.1, 63.7 (d, *J* = 2.3 Hz), 54.1 (d, *J* = 1.5 Hz), 43.7, 32.6 (q, *J* = 39.3 Hz), 30.5, 27.4.

^{19}F NMR (471 MHz, $CDCl_3$, 298 K, δ): –70.11 (s).

HRMS-ESI (m/z) calc'd for $C_{17}H_{21}OF_3Na^+$ $[M+Na]^+$, 321.1437; found, 321.1436; deviation: +0.3 ppm.

Bicyclo[1.1.1]pentylether 19

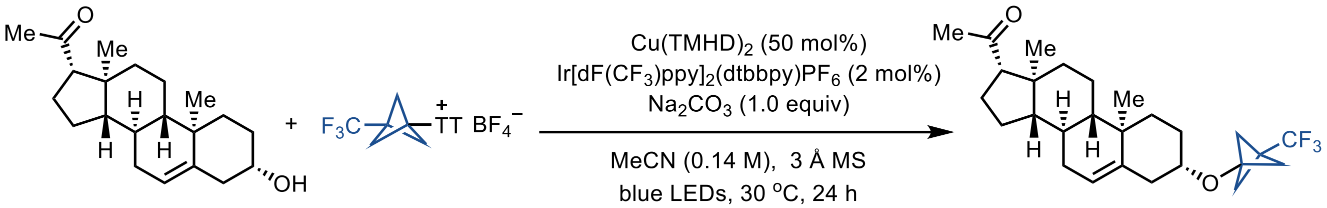

1.0 equiv. 1.5 equiv. **90%** **19**

Under nitrogen atmosphere, to a 4 mL borosilicate vial equipped with a magnetic stir bar were added pregnenolone (31.6 mg, 0.100 mmol, 1.00 equiv.), CF_3BCP-TT$^+$ BF_4^- (65.7 mg, 0.150 mmol, 1.50 equiv.), Ir[dF(CF_3)ppy]$_2$(dtbbpy)PF_6 (2 mg, 2 µmoL, 2 mol%), Cu(TMHD)$_2$ (22 mg, 50 µmoL, 50 mol%), Na_2CO_3 (10.6 mg, 0.100 mmol, 1.00 equiv.), 3Å molecular sieves (120 mg), and anhydrous MeCN (0.70 mL, *c* = 0.14 M). The vial was sealed with a septum-cap. Then, the mixture was stirred for 1 h at 25 °C, and placed 5 cm away from two blue LEDs (Kessil A160WE Tuna Blue (460 nm), LED lighting, 40 W). The mixture was irradiated for 24 h while maintaining the temperature at approximately 30 °C through cooling with a fan. After irradiation, the mixture was concentrated under reduced pressure. The residue was purified by flash column chromatography on silica gel eluting with EtOAc/pentane (0:100–1:30 (v/v)) to afford the title compound **19** as a colorless solid (40.6 mg, 90.0 µmol, 90%).

R*f* = 0.21 (pentane/EtOAc, 20:1 (v/v)).

NMR Spectroscopy:

^{1}H NMR (600 MHz, $CDCl_3$, 298 K, δ): 5.35 (d, *J* = 6.9 Hz, 1H), 3.59 – 3.28 (m, 1H), 2.53 (t, *J* = 9.0 Hz, 1H), 2.27 (d, *J* = 7.4 Hz, 2H), 2.21 – 2.16 (m, 1H), 2.13 (d, *J* = 7.3 Hz, 9H), 2.07 – 1.96 (m, 2H), 1.91 – 1.79 (m, 2H), 1.73 – 1.61 (m, 3H), 1.57 – 1.41 (m, 5H), 1.27 – 1.19 (m, 1H), 1.18 – 1.11 (m, 1H), 1.07 (td, *J* = 13.6, 3.7 Hz, 1H), 1.04 – 0.92 (m, 4H), 0.63 (s, 3H).

^{13}C NMR (151 MHz, $CDCl_3$, 298 K, δ): 209.7, 140.6, 124.4 (q, J = 273.5 Hz), 121.9, 76.7, 65.4 (q, J = 2.4 Hz), 63.8, 57.1, 51.8 (d, J = 1.6 Hz), 50.2, 44.1, 40.0, 39.0, 37.3, 36.8, 32.0, 31.9, 31.7, 31.6 (q, J = 39.7 Hz), 29.3, 24.6, 23.0, 21.2, 19.4, 13.4.

^{19}F NMR (565 MHz, $CDCl_3$, 298 K, δ): –70.08 (s).

HRMS-ESI (m/z) calc'd for $C_{27}H_{38}O_2F_3^+$ $[M+H]^+$, 451.2818; found, 451.2824; deviation: –1.3 ppm.

Bicyclo[1.1.1]pentylether 20

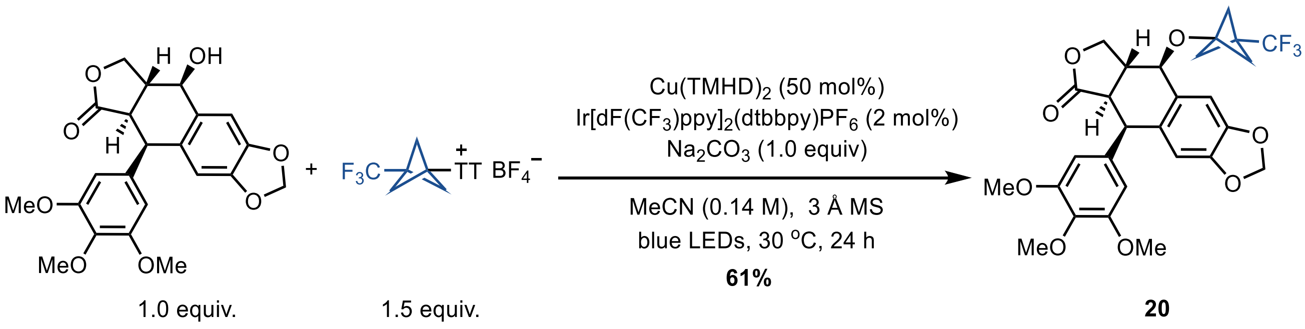

Under nitrogen atmosphere, to a 4 mL borosilicate vial equipped with a magnetic stir bar were added podophyllotoxin (41.4 mg, 0.100 mmol, 1.00 equiv.), $CF_3BCP\text{-}TT^+$ BF_4^- (65.7 mg, 0.150 mmol, 1.50 equiv.), Ir[dF(CF_3)ppy]$_2$(dtbbpy)PF_6 (2 mg, 2 µmoL, 2 mol%), Cu(TMHD)$_2$ (22 mg, 50 µmoL, 50 mol%), Na_2CO_3 (10.6 mg, 0.100 mmol, 1.00 equiv.), 3Å molecular sieves (120 mg), and anhydrous MeCN (0.70 mL, c = 0.14 M). The vial was sealed with a septum-cap. Then, the mixture was stirred for 1 h at 25 °C, and placed 5 cm away from two blue LEDs (Kessil A160WE Tuna Blue (460 nm), LED lighting, 40 W). The mixture was irradiated for 24 h while maintaining the temperature at approximately 30 °C through cooling with a fan. After irradiation, the mixture was concentrated under reduced pressure. The residue was purified by flash column chromatography on silica gel eluting with EtOAc/pentane (0:100–1:4 (v/v)) to afford the title compound **20** as a colorless oil (33.4 mg, 61.0 µmol, 61%).

R_f = 0.45 (pentane/EtOAc, 2:1 (v/v)).

NMR Spectroscopy:

^{1}H NMR (500 MHz, $CDCl_3$, 298 K, δ): 6.83 (s, 1H), 6.50 (s, 1H), 6.35 (s, 2H), 5.97 (dd, J = 5.2, 1.5 Hz, 2H), 4.63 (d, J = 9.3 Hz, 1H), 4.57 (d, J = 4.7 Hz, 1H), 4.50 (dd, J = 8.5, 6.9 Hz, 1H), 4.00 (dd, J = 10.3, 8.5 Hz, 1H), 3.80 (s, 3H), 3.74 (s, 6H), 2.93 – 2.77 (m, 2H), 2.25 (qd, J = 9.5, 1.5 Hz, 6H).

^{13}C NMR (126 MHz, $CDCl_3$, 298 K, δ): 174.0, 152.8, 148.1, 147.7, 137.3, 135.2, 131.7, 130.7, 124.1 (d, J = 273.0 Hz), 109.9, 108.3, 106.8, 101.6, 77.3, 71.0, 67.0 (d, J = 2.4 Hz), 60.9, 56.3, 51.8 (d, J = 1.3 Hz), 45.5, 44.0, 38.7, 31.3 (d, J = 40.0 Hz).

^{19}F NMR (471 MHz, $CDCl_3$, 298 K, δ): –69.95 (s).

HRMS-ESI (m/z) calc'd for $C_{28}H_{27}O_8F_3Na^+$ $[M+Na]^+$, 571.1550; found, 571.1553; deviation: –0.5 ppm.

Bicyclo[1.1.1]pentylether 21

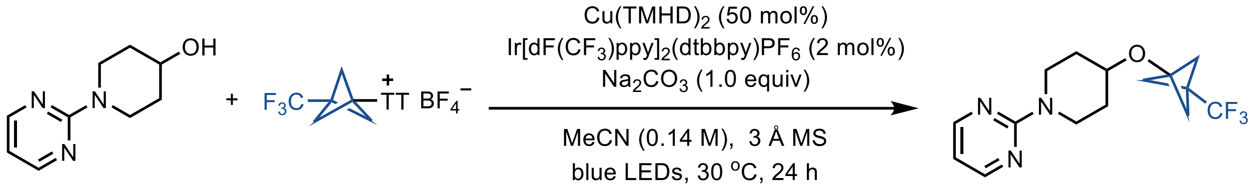

1.0 equiv. 1.5 equiv. **96%** **21**

Under nitrogen atmosphere, to a 4 mL borosilicate vial equipped with a magnetic stir bar were added 1-(pyrimidin-2-yl)-piperidin-4-ol (17.9 mg, 0.100 mmol, 1.00 equiv.), CF_3BCP-TT$^+$ BF_4^- (65.7 mg, 0.150 mmol, 1.50 equiv.), Ir[dF(CF_3)ppy]$_2$(dtbbpy)PF_6 (2 mg, 2 µmoL, 2 mol%), Cu(TMHD)$_2$ (22 mg, 50 µmoL, 50 mol%), Na_2CO_3 (10.6 mg, 0.100 mmol, 1.00 equiv.), 3Å molecular sieves (120 mg), and anhydrous MeCN (0.70 mL, *c* = 0.14 M). The vial was sealed with a septum-cap. Then, the mixture was stirred for 1 h at 25 °C, and placed 5 cm away from two blue LEDs (Kessil A160WE Tuna Blue (460 nm), LED lighting, 40 W). The mixture was irradiated for 24 h while maintaining the temperature at approximately 30 °C through cooling with a fan. After irradiation, the mixture was concentrated under reduced pressure. The residue was purified by flash column chromatography on silica gel eluting with EtOAc/pentane (0:100–1:10 (v/v)) to afford the title compound **21** as a colorless oil (30.0 mg, 96.0 µmol, 96%).

R_f = 0.38 (pentane/EtOAc, 5:1 (v/v)).

NMR Spectroscopy:

^{1}H NMR (500 MHz, $CDCl_3$, 298 K, δ): 8.29 (d, *J* = 4.7 Hz, 2H), 6.45 (t, *J* = 4.7 Hz, 1H), 4.25 (dd, *J* = 9.1, 5.9 Hz, 2H), 3.78 (p, *J* = 4.1 Hz, 1H), 3.44 (ddd, *J* = 13.1, 9.2, 3.6 Hz, 2H), 2.16 (s, 6H), 1.87 (dt, *J* = 6.1, 3.4 Hz, 2H), 1.60 (dt, *J* = 8.7, 4.2 Hz, 2H).

^{13}C NMR (151 MHz, $CDCl_3$, 298 K, δ): 161.7, 157.9, 124.4 (q, *J* = 273.4 Hz), 109.8, 73.0, 65.6 (d, *J* = 2.5 Hz), 51.8 (q, *J* = 1.7 Hz), 41.3, 31.9, 31.7 (q, *J* = 39.7 Hz).

^{19}F NMR (565 MHz, $CDCl_3$, 298 K, δ): –70.10 (s).

HRMS-ESI (m/z) calc'd for $C_{15}H_{19}N_3OF_3^+$ $[M+H]^+$, 314.1474; found, 314.1473; deviation: +0.4 ppm.

Bicyclo[1.1.1]pentylether 22

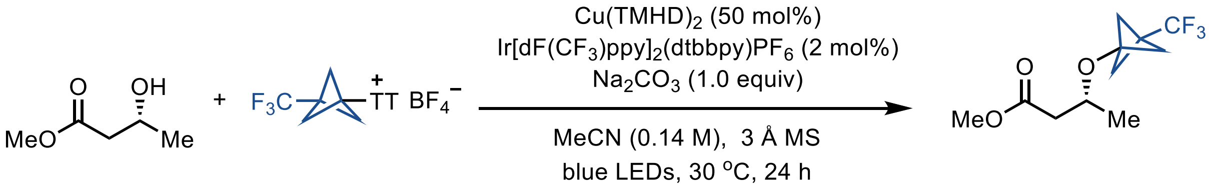

1.0 equiv. 1.5 equiv. **90%** **22**

Under nitrogen atmosphere, to a 4 mL borosilicate vial equipped with a magnetic stir bar were added methyl (R)-(-)-3-hydroxybutyrate (11.8 mg, 0.100 mmol, 1.00 equiv.), CF_3BCP-TT$^+$ BF_4^- (65.7 mg, 0.150 mmol,

1.50 equiv.), Ir[dF(CF_3)ppy]$_2$(dtbbpy)PF_6 (2 mg, 2 µmoL, 2 mol%), Cu(TMHD)$_2$ (22 mg, 50 µmoL, 50 mol%), Na_2CO_3 (10.6 mg, 0.100 mmol, 1.00 equiv.), 3Å molecular sieves (120 mg), and anhydrous MeCN (0.70 mL, *c* = 0.14 M). The vial was sealed with a septum-cap. Then, the mixture was stirred for 1 h at 25 °C, and placed 5 cm away from two blue LEDs (Kessil A160WE Tuna Blue (460 nm), LED lighting, 40 W). The mixture was irradiated for 24 h while maintaining the temperature at approximately 30 °C through cooling with a fan. After irradiation, the mixture was concentrated under reduced pressure. The residue was purified by flash column chromatography on silica gel eluting with EtOAc/pentane (0:100–1:20 (v/v)) to afford the title compound **22** as a colorless oil (22.7 mg, 90.0 µmol, 90%).

R*f* = 0.36 (pentane/EtOAc, 10:1 (v/v)).

NMR Spectroscopy:

^{1}H NMR (500 MHz, $CDCl_3$, 298 K, δ): 4.14 – 4.04 (m, 1H), 3.68 (s, 3H), 2.55 (dd, *J* = 15.5, 7.9 Hz, 1H), 2.40 (dd, *J* = 15.5, 5.4 Hz, 1H), 2.19 – 2.08 (m, 6H), 1.22 (d, *J* = 6.3 Hz, 3H).

^{13}C NMR (126 MHz, $CDCl_3$, 298 K, δ): 171.5, 124.4 (q, *J* = 273.5 Hz), 69.9, 65.6 (d, *J* = 2.4 Hz), 51.8, 51.6 (d, *J* = 1.3 Hz), 42.2, 31.4 (q, *J* = 39.6 Hz), 21.2.

^{19}F NMR (471 MHz, $CDCl_3$, 298 K, δ): –70.09 (s).

HRMS-ESI (m/z) calc'd for $C_{11}H_{15}O_3F_3Na^+$ $[M+Na]^+$, 275.0866; found, 275.0865; deviation: +0.1 ppm.

Bicyclo[1.1.1]pentylether 23

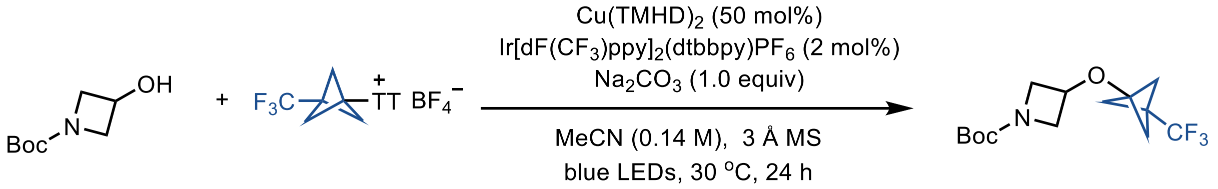

1.0 equiv. 1.5 equiv. **80%** **23**

Under nitrogen atmosphere, to a 4 mL borosilicate vial equipped with a magnetic stir bar were added N-Boc-3-hydroxyazetidine (17.3 mg, 0.100 mmol, 1.00 equiv.), CF_3BCP-TT^+ BF_4^- (65.7 mg, 0.150 mmol, 1.50 equiv.), Ir[dF(CF_3)ppy]$_2$(dtbbpy)PF_6 (2 mg, 2 µmoL, 2 mol%), Cu(TMHD)$_2$ (22 mg, 50 µmoL, 50 mol%), Na_2CO_3 (10.6 mg, 0.100 mmol, 1.00 equiv.), 3Å molecular sieves (120 mg), and anhydrous MeCN (0.70 mL, *c* = 0.14 M). The vial was sealed with a septum-cap. Then, the mixture was stirred for 1 h at 25 °C, and placed 5 cm away from two blue LEDs (Kessil A160WE Tuna Blue (460 nm), LED lighting, 40 W). The mixture was irradiated for 24 h while maintaining the temperature at approximately 30 °C through cooling with a fan. After irradiation, the mixture was concentrated under reduced pressure. The residue was purified by flash column chromatography on silica gel eluting with EtOAc/pentane (1:100–1:12 (v/v)) to afford the title compound **23** as a colorless solid (24.6 mg, 80.0 µmol, 80%).

R*f* = 0.26 (pentane/EtOAc, 10:1 (v/v)).

NMR Spectroscopy:

^{1}**H NMR** (500 MHz, $CDCl_3$, 298 K, δ): 4.33 (td, *J* = 6.6, 3.2 Hz, 1H), 4.09 (dd, *J* = 8.9, 7.1 Hz, 2H), 3.83 (dd, *J* = 10.0, 4.8 Hz, 2H), 2.10 (s, 6H), 1.43 (s, 9H).

^{13}**C NMR** (126 MHz, $CDCl_3$, 298 K, δ): 156.2, 123.9 (q, *J* = 273.6 Hz), 79.8, 65.0 (q, *J* = 2.4 Hz), 64.8, 56.7, 51.0 (d, *J* = 1.5 Hz), 31.5 (q, *J* = 39.9 Hz), 28.3.

^{19}**F NMR** (471 MHz, $CDCl_3$, 298 K, δ): –70.16 (s).

HRMS-ESI (m/z) calc'd for $C_{14}H_{20}NO_3F_3Na^+$ $[M+Na]^+$, 330.1287; found, 330.1286; deviation: +0.5 ppm.

Bicyclo[1.1.1]pentylether 24

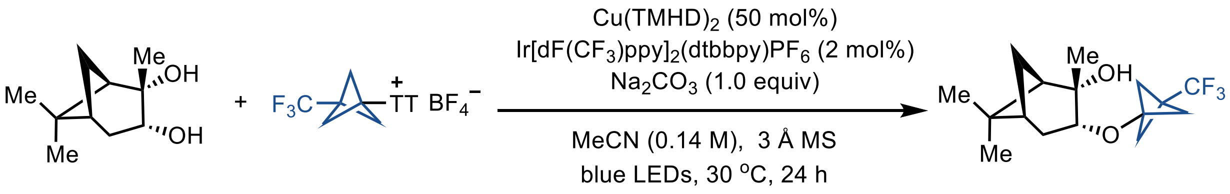

1.0 equiv. 1.5 equiv. **75%** **24**

Under nitrogen atmosphere, to a 4 mL borosilicate vial equipped with a magnetic stir bar were added (1R,2S,3S,5R)-2,6,6-trimethylbicyclo[3.1.1]heptane-2,3-diol (17.0 mg, 0.100 mmol, 1.00 equiv.), $CF_3BCP\text{-}TT^+$ BF_4^- (65.7 mg, 0.150 mmol, 1.50 equiv.), Ir[dF(CF_3)ppy]$_2$(dtbbpy)PF_6 (2 mg, 2 µmoL, 2 mol%), Cu(TMHD)$_2$ (22 mg, 50 µmoL, 50 mol%), Na_2CO_3 (10.6 mg, 0.100 mmol, 1.00 equiv.), 3Å molecular sieves (120 mg), and anhydrous MeCN (0.70 mL, *c* = 0.14 M). The vial was sealed with a septum-cap. Then, the mixture was stirred for 1 h at 25 °C, and placed 5 cm away from two blue LEDs (Kessil A160WE Tuna Blue (460 nm), LED lighting, 40 W). The mixture was irradiated for 24 h while maintaining the temperature at approximately 30 °C through cooling with a fan. After irradiation, the mixture was concentrated under reduced pressure. The residue was purified by flash column chromatography on silica gel eluting with EtOAc/pentane (0:100–1:20 (v/v)) to afford the title compound **24** as a colorless oil (22.7 mg, 75.0 µmol, 75%).

R_f = 0.33 (pentane/EtOAc, 10:1 (v/v)).

NMR Spectroscopy:

^{1}**H NMR** (500 MHz, $CDCl_3$, 298 K, δ): 3.82 (dd, *J* = 9.4, 5.3 Hz, 1H), 3.29 (s, 1H), 2.33 (dddd, *J* = 13.3, 9.3, 3.7, 2.4 Hz, 1H), 2.20 (dd, *J* = 9.5, 1.5 Hz, 4H), 2.11 (dd, *J* = 9.3, 1.7 Hz, 3H), 1.98 (t, *J* = 5.8 Hz, 1H), 1.94 – 1.87 (m, 1H), 1.71 (ddd, *J* = 13.7, 5.3, 2.6 Hz, 1H), 1.48 (d, *J* = 10.4 Hz, 1H), 1.26 (d, *J* = 14.5 Hz, 6H), 0.94 (s, 3H).

^{13}**C NMR** (126 MHz, $CDCl_3$, 298 K, δ): 124.3 (q, *J* = 273.5 Hz), 74.8, 73.2, 66.1 (d, *J* = 2.4 Hz), 53.6, 51.7 (d, *J* = 1.2 Hz), 40.5, 38.5, 36.5, 31.5 (q, *J* = 40.5 Hz), 30.4, 28.5, 28.0, 24.5.

^{19}**F NMR** (471 MHz, $CDCl_3$, 298 K, δ): –70.01 (s).

HRMS-ESI (m/z) calc'd for $C_{16}H_{23}O_2F_3Na^+$ [M+Na]$^+$, 327.1542; found, 327.1542; deviation: +0.2 ppm.

Bicyclo[1.1.1]pentylether 25

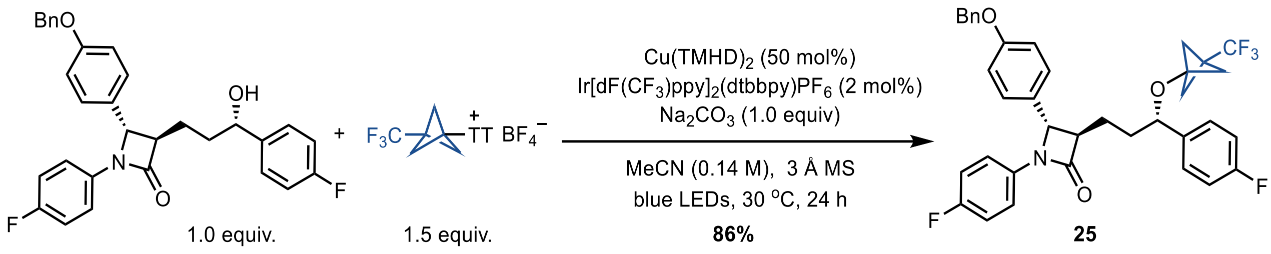

Under nitrogen atmosphere, to a 4 mL borosilicate vial equipped with a magnetic stir bar were added O-Bn-ezetimibe (25.0 mg, 50.0 µmol, 1.00 equiv.), CF_3BCP-TT$^+$ BF_4^- (32.8 mg, 75.0 µmol, 1.50 equiv.), $Ir[dF(CF_3)ppy]_2(dtbbpy)PF_6$ (1 mg, 1 µmoL, 2 mol%), $Cu(TMHD)_2$ (11 mg, 25 µmoL, 50 mol%), Na_2CO_3 (5.3 mg, 0.10 mmol, 1.0 equiv.), 3Å molecular sieves (60 mg), and anhydrous MeCN (0.35 mL, *c* = 0.14 M). The vial was sealed with a septum-cap. Then, the mixture was stirred for 1 h at 25 °C, and placed 5 cm away from two blue LEDs (Kessil A160WE Tuna Blue (460 nm), LED lighting, 40 W). The mixture was irradiated for 24 h while maintaining the temperature at approximately 30 °C through cooling with a fan. After irradiation, the mixture was concentrated under reduced pressure. The residue was purified by flash column chromatography on silica gel eluting with EtOAc/pentane (0:100–1:10 (v/v)) to afford the title compound **25** as a colorless oil (27.3 mg, 43.0 µmol, 86%).

$\mathbf{R}_f$ = 0.38 (pentane/EtOAc, 5:1 (v/v)).

NMR Spectroscopy:

^{1}H NMR (500 MHz, $CDCl_3$, 298 K, δ): 7.45 – 7.30 (m, 5H), 7.22 (td, *J* = 9.5, 4.4 Hz, 6H), 7.05 – 6.88 (m, 6H), 5.05 (s, 2H), 4.57 (d, *J* = 2.4 Hz, 1H), 4.42 – 4.36 (m, 1H), 3.02 (dq, *J* = 6.4, 3.1 Hz, 1H), 2.11 – 1.70 (m, 10H).

^{13}C NMR (126 MHz, $CDCl_3$, 298 K, δ): 167.4, 162.5 (d, *J* = 246.2 Hz), 159.2, 159.1 (d, *J* = 243.5 Hz), 138.2 (d, *J* = 3.5 Hz), 136.8, 134.1 (d, *J* = 3.0 Hz), 129.8, 128.8, 128.3, 127.9 (d, *J* = 8.1 Hz), 127.6, 127.3, 124.2 (q, *J* = 273.0 Hz), 118.5 (d, *J* = 7.8 Hz), 116.0 (d, *J* = 22.6 Hz), 115.7, 115.5, 78.7, 70.3, 65.9 (d, *J* = 3.0 Hz), 61.0, 60.4, 51.5 (d, *J* = 1.4 Hz), 36.0, 32.0 (q, *J* = 39.9 Hz), 25.2.

^{19}F NMR (471 MHz, $CDCl_3$, 298 K, δ): –70.12 (s, 3F), –114.24 (td, *J* = 8.7, 4.3 Hz, 1F), –118.11 (tt, *J* = 8.7, 5.2 Hz, 1F).

HRMS-ESI (m/z) calc'd for $C_{37}H_{32}NO_3F_5Na^+$ [M+Na]$^+$, 656.2195; found, 656.2198; deviation: –0.5 ppm.

Bicyclo[1.1.1]pentylether 26

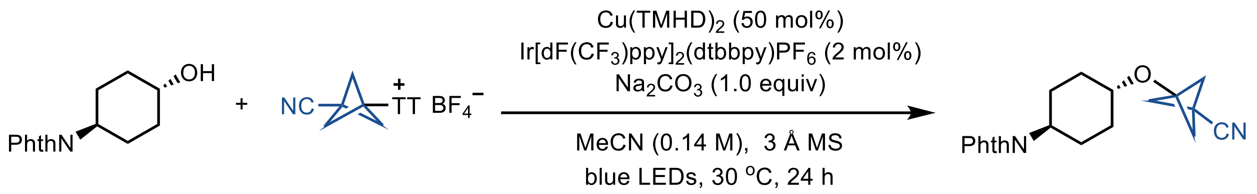

1.0 equiv. 1.5 equiv. **62%** **26**

Under nitrogen atmosphere, to a 4 mL borosilicate vial equipped with a magnetic stir bar were added trans-2-(4-hydroxycyclohexyl)-1H-isoindole-1,3(2H)-dione (24.5 mg, 0.100 mmol, 1.00 equiv.), CNBCP-TT$^+$ BF_4^- (59.3 mg, 0.150 mmol, 1.50 equiv.), Ir[dF(CF_3)ppy]$_2$(dtbbpy)PF_6 (2 mg, 2 µmoL, 2 mol%), Cu(TMHD)$_2$ (22 mg, 50 µmoL, 50 mol%), Na_2CO_3 (10.6 mg, 0.100 mmol, 1.00 equiv.), 3Å molecular sieves (120 mg), and anhydrous MeCN (0.70 mL, *c* = 0.14 M). The vial was sealed with a septum-cap. Then, the mixture was stirred for 1 h at 25 °C, and placed 5 cm away from two blue LEDs (Kessil A160WE Tuna Blue (460 nm), LED lighting, 40 W). The mixture was irradiated for 24 h while maintaining the temperature at approximately 30 °C through cooling with a fan. After irradiation, the mixture was concentrated under reduced pressure. The residue was purified by flash column chromatography on silica gel eluting with EtOAc/pentane (0:100–1:10 (v/v)) to afford the title compound **26** as a colorless solid (20.8 mg, 62.0 µmol, 62%).

R_f = 0.17 (pentane/EtOAc, 5:1 (v/v)).

NMR Spectroscopy:

^{1}H NMR (500 MHz, $CDCl_3$, 298 K, δ): 7.82 (dd, *J* = 5.3, 3.1 Hz, 2H), 7.71 (dd, *J* = 5.4, 3.0 Hz, 2H), 4.12 (ddd, *J* = 12.2, 8.3, 4.0 Hz, 1H), 3.67 – 3.45 (m, 1H), 2.41 (s, 6H), 2.32 (qd, *J* = 13.4, 3.7 Hz, 2H), 2.02 (d, *J* = 13.4 Hz, 2H), 1.77 (d, *J* = 13.4 Hz, 2H), 1.49 – 1.37 (m, 2H).

^{13}C NMR (126 MHz, $CDCl_3$, 298 K, δ): 168.5, 134.1, 132.1, 123.3, 117.7, 74.9, 67.1, 57.2, 49.5, 32.4, 27.6, 18.0.

HRMS-ESI (m/z) calc'd for $C_{20}H_{20}O_3N_2Na^+$ $[M+Na]^+$, 359.1366; found, 359.1365; deviation: +0.3 ppm.

Bicyclo[1.1.1]pentylether 27

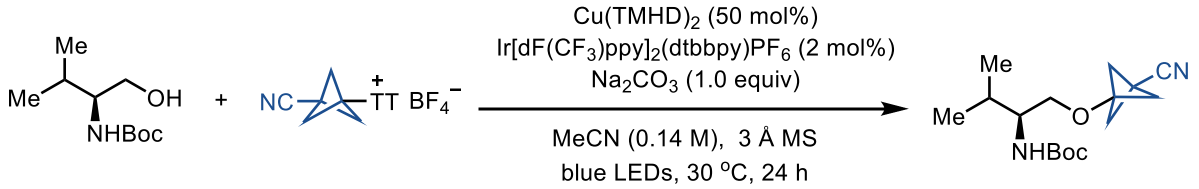

1.0 equiv. 1.5 equiv. **91%** **27**

Under nitrogen atmosphere, to a 4 mL borosilicate vial equipped with a magnetic stir bar were added *N*-Boc-*L*-valinol (20.3 mg, 0.100 mmol, 1.00 equiv.), CNBCP-TT$^+$ BF_4^- (59.3 mg, 0.150 mmol, 1.50 equiv.), Ir[dF(CF_3)ppy]$_2$(dtbbpy)PF_6 (2 mg, 2 µmoL, 2 mol%), Cu(TMHD)$_2$ (22 mg, 50 µmoL, 50 mol%), Na_2CO_3 (10.6

mg, 0.100 mmol, 1.00 equiv.), 3Å molecular sieves (120 mg), and anhydrous MeCN (0.70 mL, *c* = 0.14 M). The vial was sealed with a septum-cap. Then, the mixture was stirred for 1 h at 25 °C, and placed 5 cm away from two blue LEDs (Kessil A160WE Tuna Blue (460 nm), LED lighting, 40 W). The mixture was irradiated for 24 h while maintaining the temperature at approximately 30 °C through cooling with a fan. After irradiation, the mixture was concentrated under reduced pressure. The residue was purified by flash column chromatography on silica gel eluting with EtOAc/pentane (0:100–1:10 (v/v)) to afford the title compound **27** as a colorless oil (26.8 mg, 91.0 µmol, 91%).

R*f* = 0.31 (pentane/EtOAc, 5:1 (v/v)).

NMR Spectroscopy:

^{1}H NMR (300 MHz, $CDCl_3$, 298 K, δ): 4.56 (s, 1H), 3.54 – 3.35 (m, 3H), 2.35 (s, 6H), 1.87 – 1.71 (m, 1H), 1.44 (s, 9H), 0.90 (dd, *J* = 6.8, 2.7 Hz, 6H).

^{13}C NMR (126 MHz, $CDCl_3$, 298 K, δ): 155.9, 117.6, 79.5, 67.9, 67.2, 56.1, 55.2, 29.3, 28.5, 19.7, 18.6, 17.6.

HRMS-ESI (m/z) calc'd for $C_{16}H_{26}O_3N_2Na^+$ $[M+Na]^+$, 317.1836; found, 317.1832; deviation: +0.9 ppm.

Bicyclo[1.1.1]pentylether 28

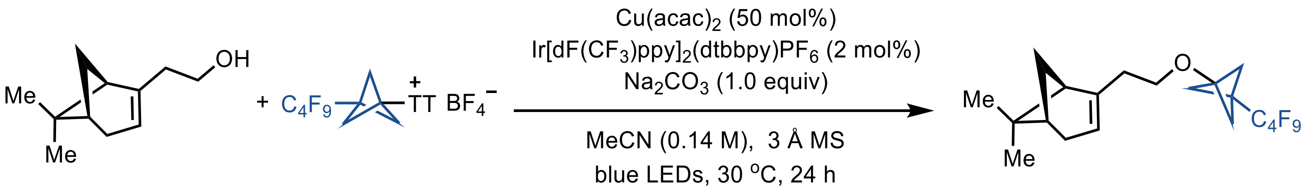

1.0 equiv. 1.5 equiv. **73%** **28**

Under nitrogen atmosphere, to a 4 mL borosilicate vial equipped with a magnetic stir bar were added (-)-nopol (16.6 mg, 0.100 mmol, 1.00 equiv.), C_4F_9BCP-TT$^+$ BF_4^- (88.2 mg, 0.150 mmol, 1.50 equiv.), Ir[dF(CF_3)ppy]$_2$(dtbbpy)PF_6 (2 mg, 2 µmoL, 2 mol%), Cu(acac)$_2$ (13 mg, 50 µmoL, 50 mol%), Na_2CO_3 (10.6 mg, 0.100 mmol, 1.00 equiv.), 3Å molecular sieves (120 mg), and anhydrous MeCN (0.70 mL, *c* = 0.14 M). The vial was sealed with a septum-cap. Then, the mixture was stirred for 1 h at 25 °C, and placed 5 cm away from two blue LEDs (Kessil A160WE Tuna Blue (460 nm), LED lighting, 40 W). The mixture was irradiated for 24 h while maintaining the temperature at approximately 30 °C through cooling with a fan. After irradiation, the mixture was concentrated under reduced pressure. The residue was purified by flash column chromatography on silica gel eluting with EtOAc/pentane (0:100–1:50 (v/v)) to afford the title compound **28** as a colorless oil (32.8 mg, 73.0 µmol, 73%).

R*f* = 0.52 (pentane/EtOAc, 20:1 (v/v)).

NMR Spectroscopy:

^{1}H NMR (500 MHz, $CDCl_3$, 298 K, δ): 5.27 (s, 1H), 3.45 (t, *J* = 7.1 Hz, 2H), 2.40 – 2.32 (m, 1H), 2.28 –

2.19 (m, 4H), 2.16 (s, 6H), 2.09 (t, J = 3.7 Hz, 1H), 2.05 – 1.95 (m, 1H), 1.27 (s, 3H), 1.15 (d, J = 8.5 Hz, 1H), 0.82 (s, 3H).

^{13}C {1H, 19F} NMR (126 MHz, $CDCl_3$, 298 K, δ): 144.7, 118.5, 117.6, 115.5, 111.3, 108.8, 66.4, 65.3, 51.5, 45.9, 40.9, 38.2, 37.1, 31.8, 31.5, 31.0, 26.5, 21.4.

^{19}F NMR (471 MHz, $CDCl_3$, 298 K, δ): –69.16 – –89.60 (m, 3F), –113.72 (t, J = 14.3 Hz, 2F), –121.96 (d, J = 9.5 Hz, 2F), –122.82 – –131.33 (m, 2F).

HRMS-APPI (m/z) calc'd for $C_{20}H_{24}OF_9^+$ $[M+H]^+$, 451.1678; found, 451.1677; deviation: +0.3 ppm.

Bicyclo[1.1.1]pentylether 29

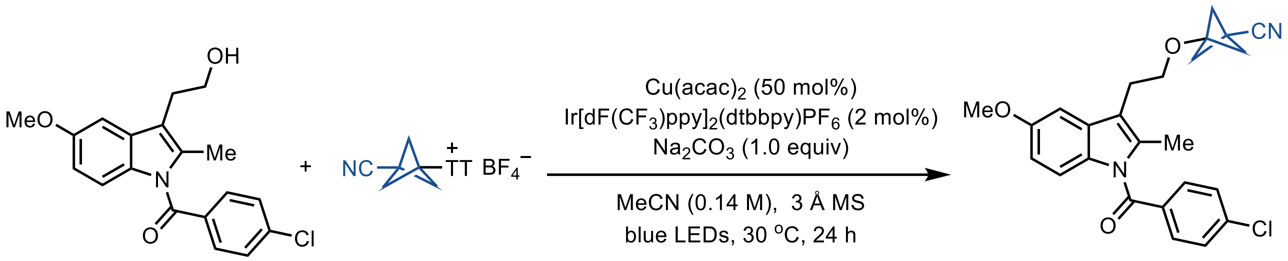

1.0 equiv. 1.5 equiv. **74%** **29**

Under nitrogen atmosphere, to a 4 mL borosilicate vial equipped with a magnetic stir bar were added indomethacin derivative (34.3 mg, 0.100 mmol, 1.00 equiv.), CNBCP-TT$^+$ BF_4^- (59.3 mg, 0.150 mmol, 1.50 equiv.), Ir[dF(CF_3)ppy]$_2$(dtbbpy)PF_6 (2 mg, 2 µmoL, 2 mol%), Cu(acac)$_2$ (13 mg, 50 µmoL, 50 mol%), Na_2CO_3 (10.6 mg, 0.100 mmol, 1.00 equiv.), 3Å molecular sieves (120 mg), and anhydrous MeCN (0.70 mL, c = 0.14 M). The vial was sealed with a septum-cap. Then, the mixture was stirred for 1 h at 25 °C, and placed 5 cm away from two blue LEDs (Kessil A160WE Tuna Blue (460 nm), LED lighting, 40 W). The mixture was irradiated for 24 h while maintaining the temperature at approximately 30 °C through cooling with a fan. After irradiation, the mixture was concentrated under reduced pressure. The residue was purified by flash column chromatography on silica gel eluting with EtOAc/pentane (0:100–1:10 (v/v)) to afford the title compound **29** as a colorless oil (32.2 mg, 74.0 µmol, 74%).

R_f = 0.24 (pentane/EtOAc, 5:1 (v/v)).

NMR Spectroscopy:

^{1}H NMR (600 MHz, $CDCl_3$, 298 K, δ): 7.65 (d, J = 8.8 Hz, 2H), 7.47 (d, J = 8.7 Hz, 2H), 6.90 (dd, J = 2.5, 0.5 Hz, 1H), 6.85 (dd, J = 9.0, 0.5 Hz, 1H), 6.66 (dd, J = 9.0, 2.6 Hz, 1H), 3.84 (s, 3H), 3.68 – 3.53 (m, 2H), 2.92 (t, J = 7.3 Hz, 2H), 2.35 (s, 3H), 2.33 (s, 6H).

^{13}C NMR (151 MHz, $CDCl_3$, 298 K, δ): 168.4, 156.1, 139.4, 135.6, 134.1, 131.3, 131.0(9), 131.0(6), 129.3, 117.6, 115.7, 115.2, 111.2, 101.5, 67.9, 66.4, 56.2, 55.9, 24.9, 17.6, 13.5.

HRMS-ESI (m/z) calc'd for $C_{25}H_{24}N_2O_3Cl^+$ $[M+H]^+$, 435.1470; found, 435.1467; deviation: +0.8 ppm.

Bicyclo[1.1.1]pentylether 30

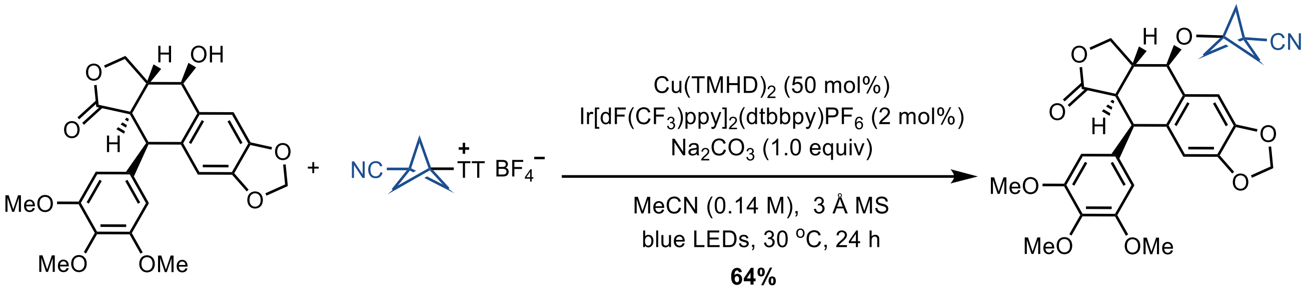

1.0 equiv. 1.5 equiv. **30**

Under nitrogen atmosphere, to a 4 mL borosilicate vial equipped with a magnetic stir bar were added podophyllotoxin (41.4 mg, 0.100 mmol, 1.00 equiv.), CNBCP-TT^+ BF_4^- (59.3 mg, 0.150 mmol, 1.50 equiv.), $Ir[dF(CF_3)ppy]_2(dtbbpy)PF_6$ (2 mg, 2 µmoL, 2 mol%), $Cu(TMHD)_2$ (22 mg, 50 µmoL, 50 mol%), Na_2CO_3 (10.6 mg, 0.100 mmol, 1.00 equiv.), 3Å molecular sieves (120 mg), and anhydrous MeCN (0.70 mL, *c* = 0.14 M). The vial was sealed with a septum-cap. Then, the mixture was stirred for 1 h at 25 °C, and placed 5 cm away from two blue LEDs (Kessil A160WE Tuna Blue (460 nm), LED lighting, 40 W). The mixture was irradiated for 24 h while maintaining the temperature at approximately 30 °C through cooling with a fan. After irradiation, the mixture was concentrated under reduced pressure. The residue was purified by flash column chromatography on silica gel eluting with EtOAc/pentane (0:100–1:3 (v/v)) to afford the title compound **30** as a colorless oil (32.2 mg, 64.0 µmol, 64%).

R_f = 0.54 (pentane/EtOAc, 1:1 (v/v)).

NMR Spectroscopy:

^{1}H NMR (300 MHz, $CDCl_3$, 298 K, δ): 6.76 (d, *J* = 0.7 Hz, 1H), 6.49 (s, 1H), 6.32 (s, 2H), 5.97 (q, *J* = 1.3 Hz, 2H), 4.69 – 4.53 (m, 2H), 4.47 (dd, *J* = 8.5, 6.5 Hz, 1H), 4.12 – 3.89 (m, 1H), 3.79 (s, 3H), 3.72 (s, 6H), 2.96 – 2.75 (m, 2H), 2.56 – 2.40 (m, 6H).

^{13}C NMR (126 MHz, $CDCl_3$, 298 K, δ): 173.8, 152.7, 148.1, 147.7, 137.3, 135.1, 131.8, 130.2, 116.9, 109.9, 108.2, 106.7, 101.7, 70.8, 68.4, 60.9, 57.0, 56.3, 45.4, 43.9, 38.6, 17.8.

HRMS-ESI (m/z) calc'd for $C_{28}H_{27}NO_8Na^+$ $[M+Na]^+$, 528.1629; found, 528.1625; deviation: +0.8 ppm.

Synthesis of BCP pharmaceutical analogs

Synthesis of BCP-fluoxetine hydrochloride (36)

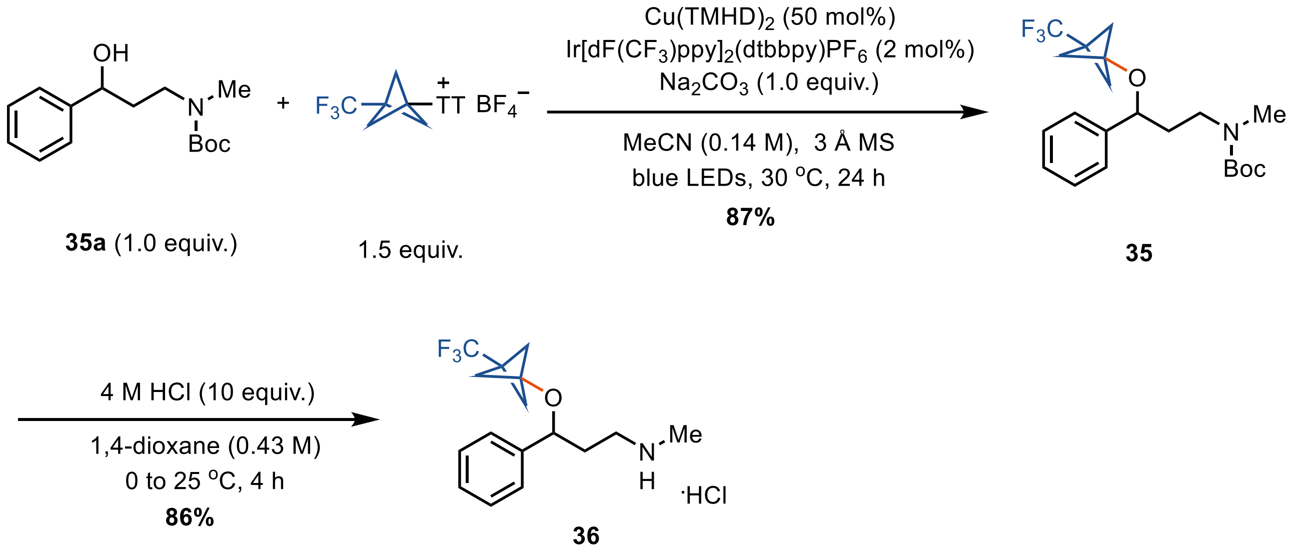

Under nitrogen atmosphere, to a 4 mL borosilicate vial equipped with a magnetic stir bar were added **35a** (26.5 mg, 0.100 mmol, 1.00 equiv.), CNBCP-TT^+ BF_4^- (59.3 mg, 0.150 mmol, 1.50 equiv.), Ir[dF(CF_3)ppy]$_2$(dtbbpy)PF_6 (2 mg, 2 µmoL, 2 mol%), Cu(TMHD)$_2$ (22 mg, 50 µmoL, 50 mol%), Na_2CO_3 (10.6 mg, 0.100 mmol, 1.00 equiv.), 3Å molecular sieves (120 mg), and anhydrous MeCN (0.70 mL, *c* = 0.14 M). The vial was sealed with a septum-cap. Then, the mixture was stirred for 1 h at 25 °C, and placed 5 cm away from two blue LEDs (Kessil A160WE Tuna Blue (460 nm), LED lighting, 40 W). The mixture was irradiated for 24 h while maintaining the temperature at approximately 30 °C through cooling with a fan. After irradiation, the mixture was concentrated under reduced pressure. The residue was purified by flash column chromatography on silica gel eluting with EtOAc/pentane (1:100–1:12 (v/v)) to afford the title compound **35** as a colorless oil (34.8 mg, 87.0 µmol, 87%).

$\mathbf{R_f}$ = 0.56 (pentane/EtOAc, 5:1 (v/v)).

NMR Spectroscopy:

^{1}H NMR (500 MHz, $CDCl_3$, 298 K, δ): 7.40 – 7.30 (m, 2H), 7.30 – 7.24 (m, 3H), 4.54 – 4.30 (m, 1H), 3.47 – 3.10 (m, 2H), 2.83 (s, 3H), 2.25 – 1.92 (m, 4H), 1.92 – 1.79 (m, 4H), 1.43 (s, 9H).

^{13}C NMR (151 MHz, $CDCl_3$, 298 K, δ): 155.9, 142.6 (d, *J* = 40.7 Hz), 128.7, 128.0, 126.3, 124.3 (q, *J* = 273.7 Hz), 79.5, 77.8, 65.9 (q, *J* = 2.2 Hz), 51.6 (d, *J* = 1.9 Hz), 45.9 (d, *J* = 34.7 Hz), 36.8 (d, *J* = 72.8 Hz), 34.7 (d, *J* = 35.3 Hz), 31.6 (q, *J* = 39.6 Hz), 28.6.

^{19}F NMR (565 MHz, $CDCl_3$, 298 K, δ): –70.16 (s).

HRMS-ESI (m/z) calc'd for $C_{21}H_{28}NO_3F_3Na^+$ $[M+Na]^+$, 422.1913; found, 422.1917; deviation: –0.8 ppm.

Under argon atmosphere, to a 4 mL borosilicate vial equipped with a magnetic stir bar were added **31** (104 mg, 0.260 mmol, 1.00 equiv.) and anhydrous 1,4-dioxane (0.60 mL, *c* = 0.43 M). The mixture was cooled to 0 °C, and 4 M HCl (in 1,4-dioxane, 0.66 mL, 2.6 mmol, 10 equiv.) was added slowly. Then, the mixture was stirred for 4 h at 25 °C. After that, the mixture was concentrated to dryness under reduced pressure. Diethyl ether (2 mL) was added to the residue, and the suspension was stirred vigorously at room temperature for 30 min. The product was collected by filtration, washed with Et_2O (3 × 2 mL), and dried under vacuum to afford **36** as a colorless solid (75 mg, 0.22 mmol, 86%).

$\mathbf{R_f}$ = 0.30 (DCM/MeOH, 10:1 (v/v)).

NMR Spectroscopy:

^{1}H NMR (500 MHz, $CDCl_3$, 298 K, δ): 9.59 (s, 1H), 7.35 – 7.25 (m, 5H), 4.66 (dd, *J* = 8.6, 4.5 Hz, 1H), 3.00 (s, 2H), 2.62 (d, *J* = 5.5 Hz, 3H), 2.31 – 2.09 (m, 2H), 1.98 (d, *J* = 9.3 Hz, 3H), 1.84 (s, 3H).

^{13}C NMR (151 MHz, $CDCl_3$, 298 K, δ): 140.9, 128.9, 128.5, 126.3, 124.1 (q, *J* = 273.5 Hz), 76.9, 65.9 (d, *J* = 2.2 Hz), 51.6 (q, *J* = 1.6 Hz), 46.3, 34.2, 33.0, 31.6 (q, *J* = 39.7 Hz).

^{19}F NMR (565 MHz, $CDCl_3$, 298 K, δ): –70.07 (s).

HRMS-ESI (m/z) calc'd for $C_{16}H_{21}NOF_3^+$ $[M-Cl]^+$, 300.1570; found, 300.1571; deviation: –0.4 ppm.

Synthesis of BCP-butoxycaine (38)

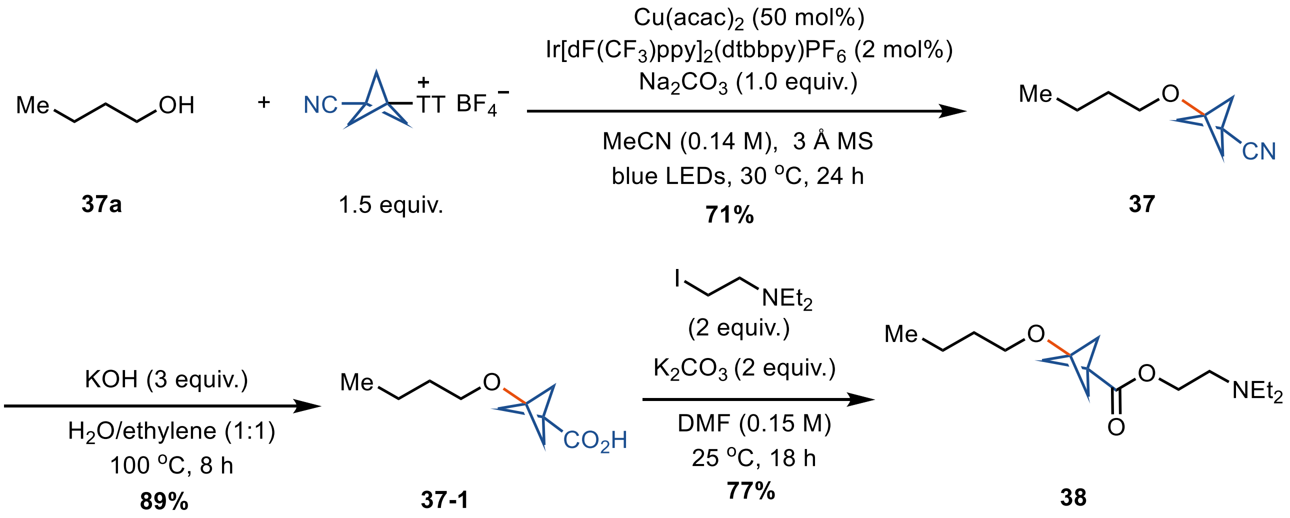

Under nitrogen atmosphere, to a 4 mL borosilicate vial equipped with a magnetic stir bar were added CNBCP-TT^+ BF_4^- (59.3 mg, 0.150 mmol, 1.50 equiv.), $Ir[dF(CF_3)ppy]_2(dtbbpy)PF_6$ (2 mg, 2 µmoL, 2 mol%), $Cu(acac)_2$ (13 mg, 50 µmoL, 50 mol%), Na_2CO_3 (10.6 mg, 0.100 mmol, 1.00 equiv.), 3Å molecular sieves (120 mg), and anhydrous MeCN (0.70 mL, *c* = 0.14 M). The vial was sealed with a septum-cap, and transferred out of the glove box. Then, **37a** (7.4 mg, 0.10 mmol, 1.0 equiv.) was added to the vial. The mixture was stirred for 1 h at 25 °C, and placed 5 cm away from two blue LEDs (Kessil A160WE Tuna Blue

(460 nm), LED lighting, 40 W). The mixture was irradiated for 24 h while maintaining the temperature at approximately 30 °C through cooling with a fan. After irradiation, the mixture was concentrated under reduced pressure. The residue was purified by flash column chromatography on silica gel eluting with EtOAc/pentane (0:100–1:4 (v/v)) to afford the title compound **37** as a colorless oil (11.7 mg, 71.0 µmol, 71%).

R*f* = 0.35 (pentane/EtOAc, 20:1 (v/v)).

NMR Spectroscopy:

^{1}H NMR (500 MHz, $CDCl_3$, 298 K, δ): 3.41 (t, *J* = 6.6 Hz, 2H), 2.35 (s, 6H), 1.57 – 1.48 (m, 2H), 1.40 – 1.29 (m, 2H), 0.90 (t, *J* = 7.4 Hz, 3H).

^{13}C NMR (126 MHz, $CDCl_3$, 298 K, δ): 117.8, 67.8, 67.0, 56.1, 31.6, 19.2, 17.6, 13.9.

HRMS-ESI (m/z) calc'd for $C_{10}H_{15}ONNa^+$ $[M+Na]^+$, 188.1046; found, 188.1048; deviation: –0.9 ppm.

Under argon atmosphere, to a 5 mL round-bottom flask equipped with a magnetic stir bar were added **37** (70.0 mg, 0.420 mmol, 1.00 equiv.), KOH (71.2 mg, 1.26 mmol, 3.00 equiv.), and 1:1 H_2O-ethylene (4.2 mL, *c* = 0.10 M). Then, the mixture was stirred at 100 °C for 12 h. After cooling to 25 °C, H_2O (10 mL) was added to the mixture, and the mixture was then acidified to PH = 1–2 with 1M HCl. The aqueous phase was extracted with EtOAc (3 × 10 mL). The combined organic phase was washed with brine (1 × 5 mL), dried over Na_2SO_4, filtered, and the solvent was removed under reduced pressure to afford the title compound **37-1** as a as a pale yellow solid (68.9 mg, 0.374 mmol, 89%).

R*f* = 0.50 (pentane/EtOAc, 2:1 (v/v)).

NMR Spectroscopy:

^{1}H NMR (300 MHz, $CDCl_3$, 298 K, δ): 3.44 (t, *J* = 6.6 Hz, 2H), 2.22 (s, 6H), 1.63 – 1.48 (m, 2H), 1.46 – 1.22 (m, 2H), 0.92 (t, *J* = 7.3 Hz, 3H).

^{13}C NMR (75 MHz, $CDCl_3$, 298 K, δ): 175.8, 66.7, 66.3, 53.6, 31.8, 31.1, 19.3, 14.0.

HRMS-ESI (m/z) calc'd for $C_{10}H_{15}O_3^-$ $[M-H]^-$, 183.1027; found, 183.1028; deviation: –0.6 ppm.

Under argon atmosphere, to a 4 mL borosilicate vial equipped with a magnetic stir bar were added **37-1** (18.4 mg, 0.100 mmol, 1.00 equiv.), *N*,*N*-diethyl-2-iodoethan-1-amine (45.4 mg, 0.200 mmol, 2.00 equiv.), K_2CO_3 (27.6 mg, 0.200 mmol, 2.00 equiv.), and anhydrous DMF (0.67 mL, *c* = 0.15 M). The vial was sealed with a septum-cap. Then, the mixture was stirred for 18 h at 25 °C. EtOAc (6 mL) was added to the mixture, and washed with brine (2 × 3 mL). The organic phase was dried over Na_2SO_4, filtered, and the solvent was removed under reduced pressure. The residue was purified by flash column chromatography on silica gel eluting with DCM/MeOH (1:0–150:1 with 1% Et_3N (v/v/v)) to afford the title compound **38** as a colorless oil (21.8 mg, 77.0 µmol, 77%).

R*f* = 0.35 (DCM/MeOH, 10:1 (v/v)).

NMR Spectroscopy:

^{1}**H NMR** (300 MHz, $CDCl_3$, 298 K, δ): 4.22 (t, *J* = 6.1 Hz, 2H), 3.43 (t, *J* = 6.6 Hz, 2H), 2.80 (t, *J* = 6.1 Hz, 2H), 2.66 (q, *J* = 7.2 Hz, 4H), 2.18 (s, 6H), 1.63 – 1.46 (m, 2H), 1.46 – 1.22 (m, 2H), 1.09 (t, *J* = 7.2 Hz, 6H), 0.91 (t, *J* = 7.3 Hz, 3H).

^{13}**C NMR** (75 MHz, $CDCl_3$, 298 K, δ): 170.3, 66.6, 66.3, 62.7, 53.4, 50.7, 48.0, 31.8, 31.3, 19.4, 14.0, 11.8.

HRMS-ESI (m/z) calc'd for $C_{16}H_{30}O_3N^+$ [M+H]$^+$, 284.2220; found, 284.2221; deviation: –0.4 ppm.

Synthesis of BCP-safinamide (40)

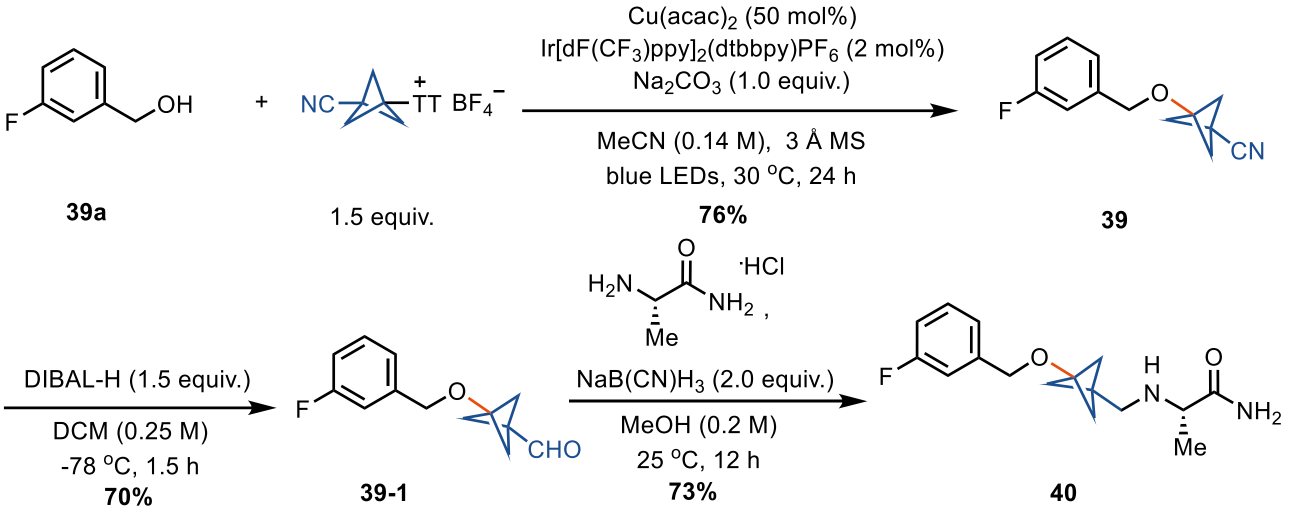

Under nitrogen atmosphere, to a 4 mL borosilicate vial equipped with a magnetic stir bar were added 3-fluorobenzyl alcohol (12.6 mg, 0.100 mmol, 1.00 equiv.), CNBCP-TT$^+$ BF_4^- (59.3 mg, 0.150 mmol, 1.50 equiv.), Ir[dF(CF_3)ppy]$_2$(dtbbpy)PF_6 (2 mg, 2 µmoL, 2 mol%), Cu(acac)$_2$ (13 mg, 50 µmoL, 50 mol%), Na_2CO_3 (10.6 mg, 0.100 mmol, 1.00 equiv.), 3Å molecular sieves (120 mg), and anhydrous MeCN (0.70 mL, *c* = 0.14 M). The vial was sealed with a septum-cap. Then, the mixture was stirred for 1 h at 25 °C, and placed 5 cm away from two blue LEDs (Kessil A160WE Tuna Blue (460 nm), LED lighting, 40 W). The mixture was irradiated for 24 h while maintaining the temperature at approximately 30 °C through cooling with a fan. After irradiation, the mixture was concentrated under reduced pressure. The residue was purified by flash column chromatography on silica gel eluting with EtOAc/pentane (0:100–1:30 (v/v)) to afford the title compound **39** as a colorless oil (16.5 mg, 76.0 µmol, 76%).

R_f = 0.20 (pentane/EtOAc, 20:1 (v/v)).

NMR Spectroscopy:

^{1}**H NMR** (300 MHz, $CDCl_3$, 298 K, δ): 7.42 – 7.27 (m, 1H), 7.12 – 6.93 (m, 3H), 4.52 (s, 2H), 2.38 (s, 6H).

^{13}C NMR (75 MHz, $CDCl_3$, 298 K, δ): 163.1 (d, *J* = 246.4 Hz), 139.9 (d, *J* = 7.2 Hz), 130.2 (d, *J* = 8.1 Hz), 122.8 (d, *J* = 3.3 Hz), 117.4, 115.0 (d, *J* = 21.1 Hz), 114.3 (d, *J* = 22.1 Hz), 68.7 (d, *J* = 2.0 Hz), 68.0, 56.4, 17.7.

^{19}F NMR (282 MHz, $CDCl_3$, 298 K, δ): –112.75 (s).

HRMS-ESI (m/z) calc'd for $C_{13}H_{12}ONFNa^+$ $[M+Na]^+$, 240.0795; found, 240.0798; deviation: –1.0 ppm.

Under argon atmosphere, to a 5 mL round-bottom flask equipped with a magnetic stir bar were added **39** (30.3 mg, 0.139 mmol, 1.00 equiv.) and anhydrous DCM (0.56 mL, *c* = 0.25 M). The mixture was cooled to -78 °C for 10 min. Then, 1 M DIBAL-H (in toluene, 209 µL, 0.209 mmol, 1.50 equiv.) was added to the mixture slowly. After 1.5 h, 1 M HCl (0.5 mL) was added. Then, the mixture was stirred at 25 °C for 30 min, and 1 M HCl (1.0 mL) was added. The aqueous phase was extracted with DCM (3 × 10 mL). The combined organic phase was washed with brine (1 × 5 mL), dried over Na_2SO_4, filtered, and the solvent was removed under reduced pressure. The residue was purified by flash column chromatography on silica gel eluting with EtOAc/pentane (0:20–1:8 (v/v)) to afford the title compound **39-1** as a colorless oil (21.4 mg, 97.3 µmol, 70%).

R_f = 0.56 (pentane/EtOAc, 3:1 (v/v)).

NMR Spectroscopy:

^{1}H NMR (500 MHz, $CDCl_3$, 298 K, δ): 9.72 (s, 1H), 7.30 (td, *J* = 7.9, 5.8 Hz, 1H), 7.10 (d, *J* = 7.6 Hz, 1H), 7.06 (d, *J* = 9.6 Hz, 1H), 6.98 (td, *J* = 8.5, 2.9 Hz, 1H), 4.54 (s, 2H), 2.19 (s, 6H).

^{13}C NMR (126 MHz, $CDCl_3$, 298 K, δ): 197.8, 163.1 (d, *J* = 246.2 Hz), 140.5, 130.1 (d, *J* = 8.3 Hz), 122.9 (d, *J* = 3.0 Hz), 114.8 (d, *J* = 20.9 Hz), 114.4 (d, *J* = 22.1 Hz), 68.3 (d, *J* = 2.4 Hz), 67.6, 52.6, 37.5.

^{19}F NMR (565 MHz, $CDCl_3$, 298 K, δ): –113.05 (td, *J* = 8.7, 5.2 Hz).

HRMS-ESI (m/z) calc'd for $C_{13}H_{13}O_2FNa^+$ $[M+Na]^+$, 243.0792; found, 243.0791; deviation: +0.5 ppm.

Under argon atmosphere, to a 4 mL borosilicate vial equipped with a magnetic stir bar were added **39-1** (21.3 mg, 97.0 µmol, 1.00 equiv.), *L*-alaninamid-hydrochlorid (24.0 mg, 0.194 mmol, 2.00 equiv.), and anhydrous MeOH (0.49 mL, *c* = 0.20 M). The vial was sealed with a septum-cap. Then, the mixture was stirred for 2 h at 25 °C, and $NaB(CN)H_3$ (12.2 mg, 0.194 mmol, 2.00 equiv.) was added. The mixture was stirred for 12 h at 25 °C. The mixture was concentrated under reduced pressure, and the residue was purified by flash column chromatography on silica gel eluting with DCM/MeOH (1:0–20:1 with 1% Et_3N (v/v/v)) to afford the title compound **40** as a colorless oil (20.7 mg, 70.8 µmol, 73%).

R_f = 0.32 (DCM/MeOH, 10:1 (v/v)).

NMR Spectroscopy:

^{1}H NMR (300 MHz, $CDCl_3$, 298 K, δ): 7.39 – 7.21 (m, 1H), 7.19 – 7.01 (m, 3H), 7.01 – 6.89 (m, 1H), 5.80 (s, 1H), 4.51 (s, 2H), 3.20 (d, *J* = 7.0 Hz, 1H), 2.80 (q, *J* = 12.6 Hz, 2H), 1.89 – 1.76 (m, 7H), 1.32 (d, *J* =

7.0 Hz, 3H).

^{13}C NMR (75 MHz, $CDCl_3$, 298 K, δ): 178.0, 163.0 (d, *J* = 245.8 Hz), 141.1 (d, *J* = 7.5 Hz), 130.0 (d, *J* = 8.1 Hz), 122.9 (d, *J* = 2.9 Hz), 114.6 (d, *J* = 15.6 Hz), 114.3 (d, *J* = 16.3 Hz), 68.2 (d, *J* = 2.0 Hz), 67.1, 58.2, 51.1, 47.7, 31.5, 19.6.

^{19}F NMR (282 MHz, $CDCl_3$, 298 K, δ): –113.31 (s).

HRMS-ESI (m/z) calc'd for $C_{16}H_{22}O_2N_2F^+$ [M+H]$^+$, 293.1660; found, 293.1663; deviation: –1.0 ppm.

Synthesis of BCP-pranlukast (42)

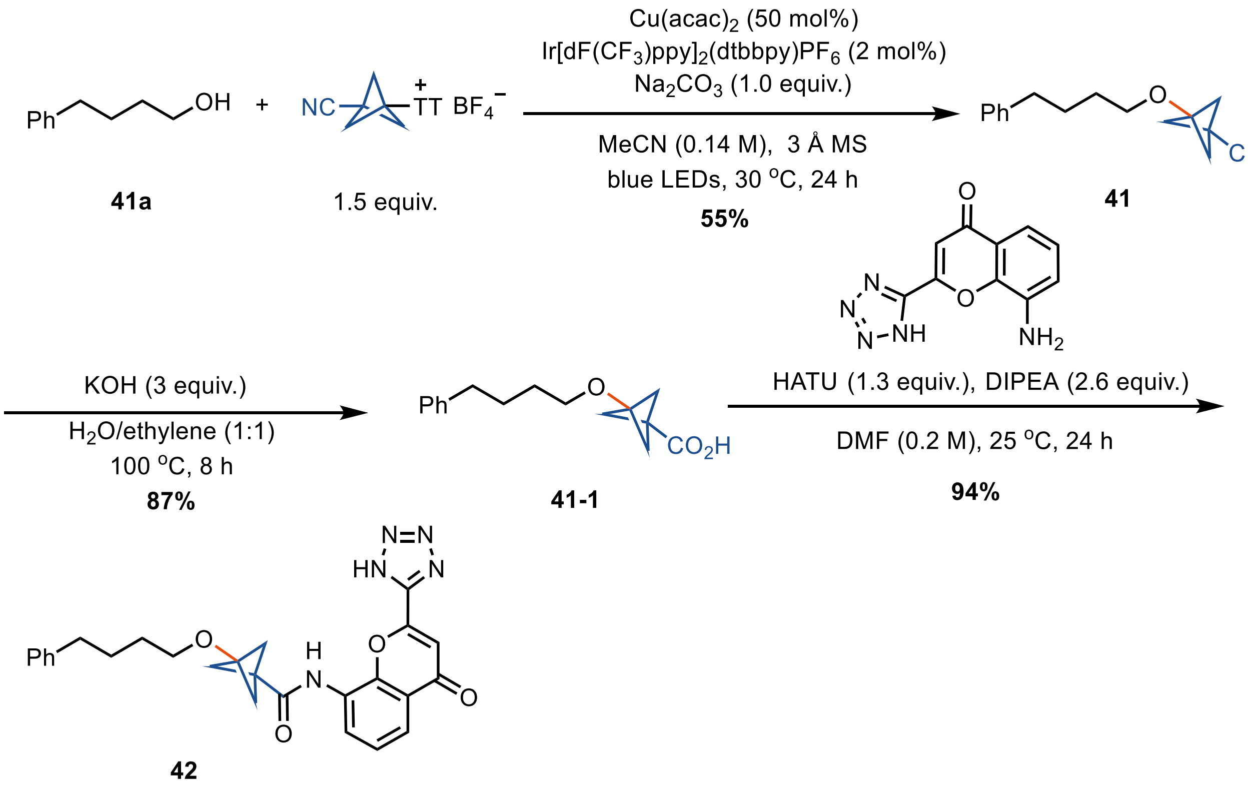

Under nitrogen atmosphere, to a 4 mL borosilicate vial equipped with a magnetic stir bar were added 4-phenyl-1-butanol (15.0 mg, 0.100 mmol, 1.00 equiv.), CNBCP-TT$^+$ BF_4^- (59.3 mg, 0.150 mmol, 1.50 equiv.), Ir[dF(CF_3)ppy]$_2$(dtbbpy)PF_6 (2 mg, 2 µmoL, 2 mol%), Cu(acac)$_2$ (13 mg, 50 µmoL, 50 mol%), Na_2CO_3 (10.6 mg, 0.100 mmol, 1.00 equiv.), 3Å molecular sieves (120 mg), and anhydrous MeCN (0.70 mL, *c* = 0.14 M). The vial was sealed with a septum-cap. Then, the mixture was stirred for 1 h at 25 °C, and placed 5 cm away from two blue LEDs (Kessil A160WE Tuna Blue (460 nm), LED lighting, 40 W). The mixture was irradiated for 24 h while maintaining the temperature at approximately 30 °C through cooling with a fan. After irradiation, the mixture was concentrated under reduced pressure. The residue was purified by flash column chromatography on silica gel eluting with EtOAc/pentane (0:100–1:3 (v/v)) to afford the title compound **41** as a colorless oil (13.3 mg, 55.0 µmol, 55%).

R*f* = 0.22 (pentane/EtOAc, 20:1 (v/v)).

NMR Spectroscopy:

^{1}H NMR (300 MHz, $CDCl_3$, 298 K, δ): 7.38 – 7.26 (m, 2H), 7.23 – 7.06 (m, 3H), 3.43 (t, *J* = 6.3 Hz, 2H), 2.62 (t, *J* = 7.4 Hz, 2H), 2.34 (s, 6H), 1.76 – 1.51 (m, 4H).

^{13}C NMR (75 MHz, $CDCl_3$, 298 K, δ): 142.2, 128.5, 128.5, 126.0, 117.7, 67.8, 67.1, 56.1, 35.7, 29.2, 27.9, 17.6.

HRMS-ESI (m/z) calc'd for $C_{16}H_{19}ONNa^+$ $[M+Na]^+$, 264.1359; found, 264.1360; deviation: –0.3 ppm.

Under argon atmosphere, to a 5 mL round-bottom flask equipped with a magnetic stir bar were added **41** (72.4 mg, 0.300 mmol, 1.00 equiv.), KOH (51.0 mg, 0.900 mmol, 3.00 equiv.), and 1:1 H_2O-ethylene (3.0 mL, *c* = 0.10 M). Then, the mixture was stirred at 100 °C for 12 h. After cooling to 25 °C, H_2O (10 mL) was added to the mixture, and the mixture was then acidified to PH = 1–2 with 1M HCl solution. The aqueous phase was extracted with EtOAc (3 × 10 mL). The combined organic phase was washed with brine (1 × 5 mL), dried over Na_2SO_4, filtered, and the solvent was removed under reduced pressure to afford the title compound **41-1** as a as a pale yellow oil (68.2 mg, 0.262 mmol, 87%).

R*f* = 0.33 (pentane/EtOAc, 2:1 (v/v)).

NMR Spectroscopy:

^{1}H NMR (300 MHz, $CDCl_3$, 298 K, δ): 7.35 – 7.24 (m, 2H), 7.21 – 7.09 (m, 3H), 3.45 (t, *J* = 6.3 Hz, 2H), 2.68 – 2.58 (m, 2H), 2.20 (s, 6H), 1.77 – 1.53 (m, 4H).

^{13}C NMR (75 MHz, $CDCl_3$, 298 K, δ): 176.1, 142.4, 128.6, 128.4, 125.9, 66.7, 66.2, 53.5, 35.8, 31.1, 29.4, 28.0.

HRMS-ESI (m/z) calc'd for $C_{16}H_{19}O_3^-$ $[M-H]^-$, 259.1340; found, 259.1341; deviation: –0.6 ppm.

Under argon atmosphere, to a 4 mL borosilicate vial equipped with a magnetic stir bar were added **41-1** (40.9 mg, 0.157 mmol, 1.00 equiv.), HATU (77.6 mg, 0.204 mmol, 1.30 equiv.), and anhydrous DMF (0.78 mL, *c* = 0.20 M). The vial was sealed with a septum-cap. Then, the mixture was stirred for 15 min at 25 °C, and DIPEA (52.8 mg, 71 µL, 0.408 mmol, 2.60 equiv.) and 8-Amino-2-(1H-tetrazol-5-yl)-4H-chromen-4-one (46.8 mg, 0.204 mmol, 1.30 equiv.) were added. The mixture was then stirred for 24 h at 25 °C. H_2O (5 mL) was added to the mixture, and the mixture was then acidified to PH = ~5 with 1M HCl solution. The aqueous phase was extracted with EtOAc (4 × 10 mL). The combined organic phase was washed with brine (1 × 5 mL), dried over Na_2SO_4, filtered, and the solvent was removed under reduced pressure. The residue was purified by flash column chromatography on silica gel eluting with DCM/MeOH (1:0–10:1 (v/v)) to afford the title compound **42** as a colorless solid (69.8 mg, 0.148 mmol, 94%).

R*f* = 0.55 (DCM/MeOH, 5:1 (v/v)).

NMR Spectroscopy:

^{1}H NMR (300 MHz, MeOH-d4, 298 K, δ): 8.34 (dd, *J* = 7.9, 1.6 Hz, 1H), 7.95 (dd, *J* = 8.0, 1.6 Hz, 1H), 7.48 (t, *J* = 8.0 Hz, 1H), 7.33 – 7.08 (m, 6H), 3.54 (t, *J* = 6.3 Hz, 2H), 2.65 (t, *J* = 7.4 Hz, 2H), 2.36 (s, 6H), 1.80 – 1.54 (m, 4H).

^{13}C NMR (151 MHz, MeOH-d4, 298 K, δ): 179.9, 171.6, 158.1, 149.3, 143.6, 129.7, 129.4, 129.3, 128.7, 126.8, 126.2, 125.4, 121.9, 109.5, 67.5, 67.0, 54.1, 36.6, 34.5, 30.4, 29.1.

HRMS-ESI (m/z) calc'd for $C_{26}H_{24}N_5O_4^-$ [M-H]$^-$, 470.1834; found, 470.1837; deviation: –0.6 ppm.

Bicyclo[1.1.1]pentylether 43

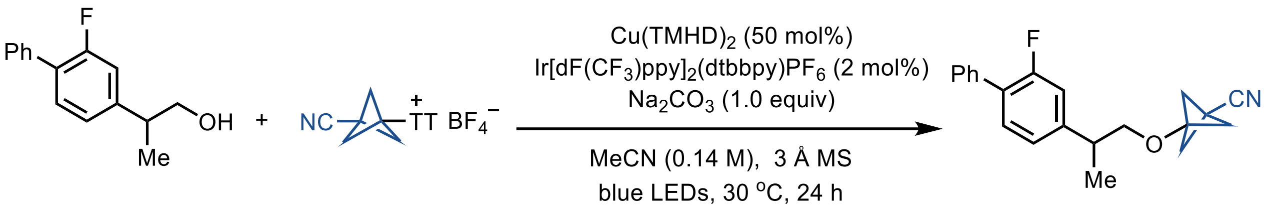

1.0 equiv. 1.5 equiv. **88 %** **43**

Under nitrogen atmosphere, to a 4 mL borosilicate vial equipped with a magnetic stir bar were added flurbiprofen derivative (23.0 mg, 0.100 mmol, 1.00 equiv.), CNBCP-TT$^+$ BF_4^- (59.3 mg, 0.150 mmol, 1.50 equiv.), Ir[dF(CF_3)ppy]$_2$(dtbbpy)PF_6 (2 mg, 2 µmoL, 2 mol%), Cu(TMHD)$_2$ (22 mg, 50 µmoL, 50 mol%), Na_2CO_3 (10.6 mg, 0.100 mmol, 1.00 equiv.), 3Å molecular sieves (120 mg), and anhydrous MeCN (0.70 mL, *c* = 0.14 M). The vial was sealed with a septum-cap. Then, the mixture was stirred for 1 h at 25 °C, and placed 5 cm away from two blue LEDs (Kessil A160WE Tuna Blue (460 nm), LED lighting, 40 W). The mixture was irradiated for 24 h while maintaining the temperature at approximately 30 °C through cooling with a fan. After irradiation, the mixture was concentrated under reduced pressure. The residue was purified by flash column chromatography on silica gel eluting with EtOAc/pentane (0:100–1:20 (v/v)) to afford the title compound **43** as a colorless oil (28.3 mg, 88.0 µmol, 88%).

R_f = 0.29 (pentane/EtOAc, 10:1 (v/v)).

NMR Spectroscopy:

^{1}H NMR (500 MHz, $CDCl_3$, 298 K, δ): 7.54 (d, *J* = 8.1 Hz, 2H), 7.44 (t, *J* = 7.6 Hz, 2H), 7.37 (q, *J* = 7.8 Hz, 2H), 7.05 (dd, *J* = 7.9, 1.5 Hz, 1H), 7.00 (dd, *J* = 11.8, 1.5 Hz, 1H), 3.57 (dd, *J* = 9.3, 6.6 Hz, 1H), 3.48 (dd, *J* = 9.2, 7.1 Hz, 1H), 3.01 (q, *J* = 6.9 Hz, 1H), 2.34 (s, 6H), 1.30 (d, *J* = 7.0 Hz, 3H).

^{13}C NMR (126 MHz, $CDCl_3$, 298 K, δ): 159.9 (d, *J* = 248.0 Hz), 145.3 (d, *J* = 7.3 Hz), 135.8, 130.8 (d, *J* = 4.0 Hz), 129.1 (d, *J* = 2.9 Hz), 128.6, 127.7, 127.4 (d, *J* = 13.6 Hz), 123.4 (d, *J* = 3.1 Hz), 117.6, 115.0 (d, *J* = 23.0 Hz), 72.4, 67.9, 56.1, 39.5, 18.1, 17.6.

^{19}F NMR (282 MHz, $CDCl_3$, 298 K, δ): –118.01 – –118.05 (m).

HRMS-EI (m/z) calc'd for $C_{21}H_{20}NOF^+$ [M]$^+$, 321.1523; found, 321.1525; deviation: –0.3 ppm.

Bicyclo[1.1.1]pentylester 44

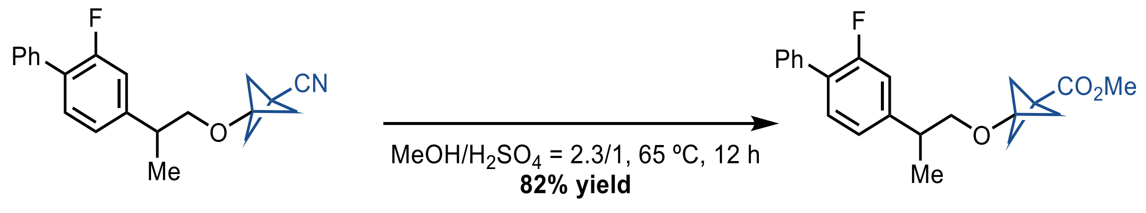

43 **44**

A 4 mL borosilicate vial equipped with a magnetic stir bar was charged with **43** (32.1 mg, 0.100 mmol, 1.00 equiv) and MeOH (800 µL, *c* = 0.125 M). The mixture was cooled to 0 °C, and concentrated H_2SO_4 (0.35 mL) was then added over 1 min. The mixture was stirred at 65 °C for 12 h. Cold water (5 mL) was added to the mixture, which was then extracted with EtOAc (3 × 15 mL). The organic phase was dried over Na_2SO_4, filtered, and the solvent was removed under reduced pressure. The residue was purified by chromatography on silica gel eluting with EtOAc/pentane (1:100–1:20 (v/v)) to afford the title compound **44** as a colorless oil (29.1 mg, 82.0 µmol, 82%).

R*f* = 0.29 (pentane/EtOAc, 10:1 (v/v)).

NMR Spectroscopy:

^{1}H NMR (500 MHz, $CDCl_3$, 298 K, δ): 7.54 (d, *J* = 7.4 Hz, 2H), 7.44 (t, *J* = 7.4 Hz, 2H), 7.37 (q, *J* = 8.3 Hz, 2H), 7.07 (d, *J* = 7.9 Hz, 1H), 7.02 (d, *J* = 11.9 Hz, 1H), 3.69 (s, 3H), 3.63 – 3.55 (m, 1H), 3.49 (t, *J* = 8.3 Hz, 1H), 3.03 (q, *J* = 6.8 Hz, 1H), 2.17 (s, 6H), 1.32 (d, *J* = 6.9 Hz, 3H).

^{13}C NMR (126 MHz, $CDCl_3$, 298 K, δ): 170.7, 159.9 (d, *J* = 247.8 Hz), 145.8 (d, *J* = 7.3 Hz), 135.9, 130.7 (d, *J* = 3.9 Hz), 129.1 (d, *J* = 2.9 Hz), 128.6, 127.7, 127.2 (d, *J* = 13.6 Hz), 123.5 (d, *J* = 3.2 Hz), 115.0 (d, *J* = 23.0 Hz), 72.1, 66.4, 53.5, 52.0, 39.6, 31.2, 18.3.

^{19}F NMR (282 MHz, $CDCl_3$, 298 K, δ): –118.18 – –118.23 (m).

HRMS-ESI (m/z) calculated for $C_{22}H_{23}FNaO_3^+$ $[M+Na]^+$, 377.1523; found, 377.1525; deviation: –0.5 ppm.

Bicyclo[1.1.1]pentylalkylamine 45

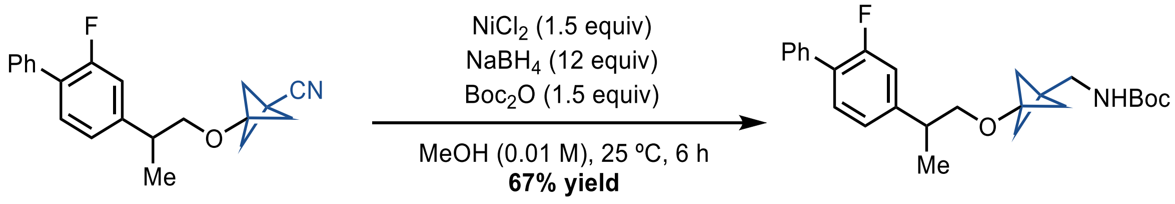

43 **45**

To a 25 mL round-bottom flask equipped with a magnetic stir bar were added **43** (32.1 mg, 0.100 mmol, 1.00 equiv), $NiCl_2$ (19.5 mg, 0.150 mmol, 1.50 equiv), Boc_2O (65.4 mg, 0.300 mmol, 3.00 equiv), and MeOH (10

mL, *c* = 10 mM). The mixture was cooled to 0 °C, and $NaBH_4$ (45.4 mg, 1.20 mmol, 12.0 equiv) was then added over 1 min. The mixture was stirred at 25 °C for 6 h. Then, saturated NH_4Cl solution (5 mL) was added to the mixture, which was then extracted with EtOAc (3 × 25 mL). The organic phase was dried over Na_2SO_4, filtered, and the solvent was removed under reduced pressure. The residue was purified by chromatography on silica gel eluting with EtOAc/pentane (1:50–1:10 (v/v)) to afford the title compound **45** as a colorless oil (28.5 mg, 67.0 µmol, 67%).

$\mathbf{R}_f$ = 0.39 (pentane/EtOAc, 5:1 (v/v)).

NMR Spectroscopy:

^{1}H NMR (500 MHz, $CDCl_3$, 298 K, δ): 7.54 (d, *J* = 7.6 Hz, 2H), 7.43 (t, *J* = 7.6 Hz, 2H), 7.36 (q, *J* = 7.6 Hz, 2H), 7.07 (d, *J* = 8.0 Hz, 1H), 7.02 (d, *J* = 12.0 Hz, 1H), 4.46 (s, 1H), 3.58 (dd, *J* = 9.3, 6.5 Hz, 1H), 3.53 – 3.41 (m, 1H), 3.33 (s, 2H), 3.03 (q, *J* = 6.9 Hz, 1H), 1.78 (s, 6H), 1.44 (s, 9H), 1.31 (d, *J* = 6.9 Hz, 3H).

^{13}C NMR (126 MHz, $CDCl_3$, 298 K, δ): 159.7 (d, *J* = 247.4 Hz), 155.9, 145.9 (d, *J* = 7.4 Hz), 135.8, 130.5 (d, *J* = 4.2 Hz), 129.0 (d, *J* = 3.0 Hz), 128.4, 127.5, 126.9 (d, *J* = 13.9 Hz), 123.4 (d, *J* = 3.0 Hz), 114.9 (d, *J* = 23.2 Hz), 79.3, 71.8, 66.7, 50.6, 40.2, 39.5, 31.2, 28.4, 18.3.

^{19}F NMR (282 MHz, $CDCl_3$, 298 K, δ): –118.32 – –118.36 (m).

HRMS-ESI (m/z) calculated for $C_{26}H_{32}FNNaO_3^+$ [M+Na]$^+$, 448.2258; found, 448.2257; deviation: +0.4 ppm.

Bicyclo[1.1.1]pentylamide 46

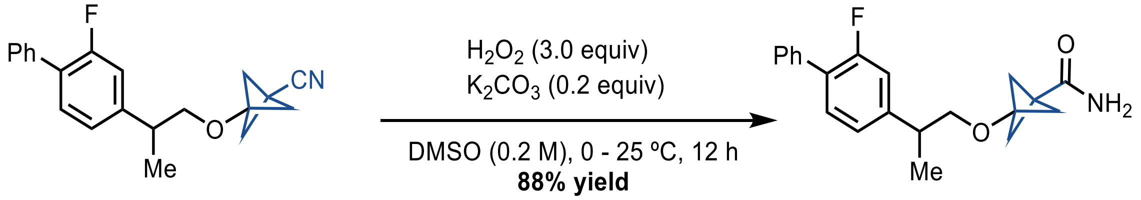

43 **46**

A 4 mL borosilicate vial equipped with a magnetic stir bar was charged with **43** (32.1 mg, 0.100 mmol, 1.00 equiv), K_2CO_3 (2.8 mg, 0.020 mmol, 0.20 equiv), and DMSO (500 µL, *c* = 0.2 M). The mixture was cooled to 0 °C, and concentrated 30% H_2O_2 (34 µL, 0.30 mmol, 3.0 equiv) was then added over 1 min. The mixture was stirred at 25 °C for 12 h. Cold water (5 mL) was added to the mixture, which was then extracted with EtOAc (3 × 15 mL). The organic phase was dried over Na_2SO_4, filtered, and the solvent was removed under reduced pressure. The residue was purified by chromatography on silica gel eluting with EtOAc/pentane (1:100–1:0 (v/v)) to afford the title compound **46** as a colorless oil (29.9 mg, 88.0 µmol, 88%).

$\mathbf{R}_f$ = 0.33 (EtOAc, 10:1 (v/v)).

NMR Spectroscopy:

^{1}H NMR (500 MHz, $CDCl_3$, 298 K, δ): 7.54 (d, *J* = 7.4 Hz, 2H), 7.44 (t, *J* = 7.4 Hz, 2H), 7.37 (q, *J* = 8.3 Hz, 2H), 7.07 (d, *J* = 7.8 Hz, 1H), 7.02 (d, *J* = 11.9 Hz, 1H), 5.57 (d, *J* = 61.9 Hz, 2H), 3.72 – 3.55 (m, 1H), 3.49 (t, *J* = 8.3 Hz, 1H), 3.03 (q, *J* = 6.8 Hz, 1H), 2.16 (s, 6H), 1.32 (d, *J* = 6.9 Hz, 3H).

^{13}C NMR (126 MHz, $CDCl_3$, 298 K, δ): 172.0, 159.0 (d, *J* = 247.9 Hz), 145.7 (d, *J* = 7.4 Hz), 135.9, 130.7 (d, *J* = 3.9 Hz), 129.1 (d, *J* = 2.9 Hz), 128.6, 127.7, 127.2 (d, *J* = 13.7 Hz), 123.5 (d, *J* = 3.0 Hz), 115.0 (d, *J* = 23.0 Hz), 72.1, 65.9, 53.0, 39.6, 32.3, 18.3.

^{19}F NMR (282 MHz, $CDCl_3$, 298 K, δ): –118.17 – –118.21 (m).

HRMS-ESI (m/z) calculated for $C_{21}H_{22}FNNaO_2^+$ $[M+Na]^+$, 362.1527; found, 362.1526; deviation: +0.2 ppm.

Bicyclo[1.1.1]pentylamine 47

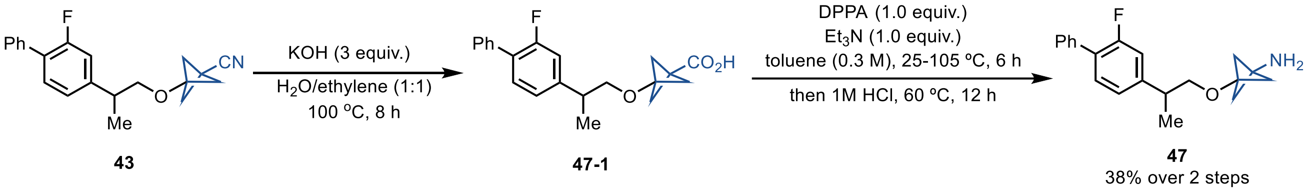

Under argon atmosphere, to a 5 mL round-bottom flask equipped with a magnetic stir bar were added **43** (32.1 mg, 0.100 mmol, 1.00 equiv.), KOH (17.0 mg, 0.300 mmol, 3.00 equiv.), and 1:1 H_2O-ethylene (1.0 mL, *c* = 0.10 M). Then, the mixture was stirred at 100 °C for 12 h. After cooling to 25 °C, H_2O (3 mL) was added to the mixture, and the mixture was then acidified to PH = 1–2 with 1M HCl solution. The aqueous phase was extracted with EtOAc (3 × 5 mL). The combined organic phase was washed with brine (1 × 5 mL), dried over Na_2SO_4, filtered, and the solvent was removed under reduced pressure to afford the crude compound **47-1**, which was transferred to a 5 mL round-bottom flask equipped with a magnetic stir bar. Then, anhydrous toluene (0.33 mL, *c* = 0.30 M), Et_3N (14 µL, 10 mg, 0.10 mmol, 1.0 equiv.) and diphenylphosphorylazide (DPPA) (22 µL, 28 mg, 0.10 mmol, 1.0 equiv.) were added at 25 °C under argon. The resulting solution was stirred at 25 °C for 3 h and then at 110 °C for 3 h, to form the corresponding isocyanate intermediate. The reaction mixture was then cooled to 60 °C, and 1M HCl (0.17 mL) was added. After 12 h, the reaction mixture was cooled to 25 °C and diluted with EtOAc (5 mL). The organic layer was separated and extracted with 1M HCl (3 × 5 mL). The combined aqueous extracts were basified to pH = 12–13 by adding 1M NaOH and extracted with DCM (3 × 10 mL). The combined organic phase was dried over Na_2SO_4, filtered, and the solvent was removed under reduced pressure. The residue was purified by fast chromatography on silica gel eluting with DCM/MeOH/Et_3N (1/0/0.01–100/1/0.5 (v/v)) to afford the title compound **47** as a yellow oil (11.8 mg, 38.0 µmol, 38%).

R_f = 0.43 (DCM/MeOH, 10:1 (v/v)).

NMR Spectroscopy:

^{1}H NMR (500 MHz, CD_2Cl_2, 298 K, δ): 7.54 (d, *J* = 7.5 Hz, 2H), 7.43 (t, *J* = 7.6 Hz, 2H), 7.36 (q, *J* = 7.4

Hz, 2H), 7.06 (d, J = 7.9 Hz, 1H), 7.01 (d, J = 11.9 Hz, 1H), 3.56 (dd, J = 9.1, 6.6 Hz, 1H), 3.44 (t, J = 8.4 Hz, 1H), 3.03 (q, J = 6.9 Hz, 1H), 2.58 (s, 2H), 1.97 (s, 6H), 1.30 (d, J = 6.9 Hz, 3H).

^{13}C NMR (126 MHz, CD_2Cl_2, 298 K, δ): 159.8 (d, J = 247.7 Hz), 146.0 (d, J = 7.7 Hz), 135.9, 130.7 (d, J = 3.9 Hz), 129.1 (d, J = 2.9 Hz), 128.5, 127.6, 127.1 (d, J = 13.6 Hz), 123.5 (d, J = 3.2 Hz), 115.0 (d, J = 23.0 Hz), 72.8, 65.3, 54.1, 39.6, 18.4.

^{19}F NMR (282 MHz, $CDCl_3$, 298 K, δ): –118.30 – –118.34 (m).

HRMS-ESI (m/z) calculated for $C_{20}H_{23}NFO^+$ $[M+H]^+$, 312.1758; found, 312.1759; deviation: –0.3 ppm.

Unsuccessful substrates

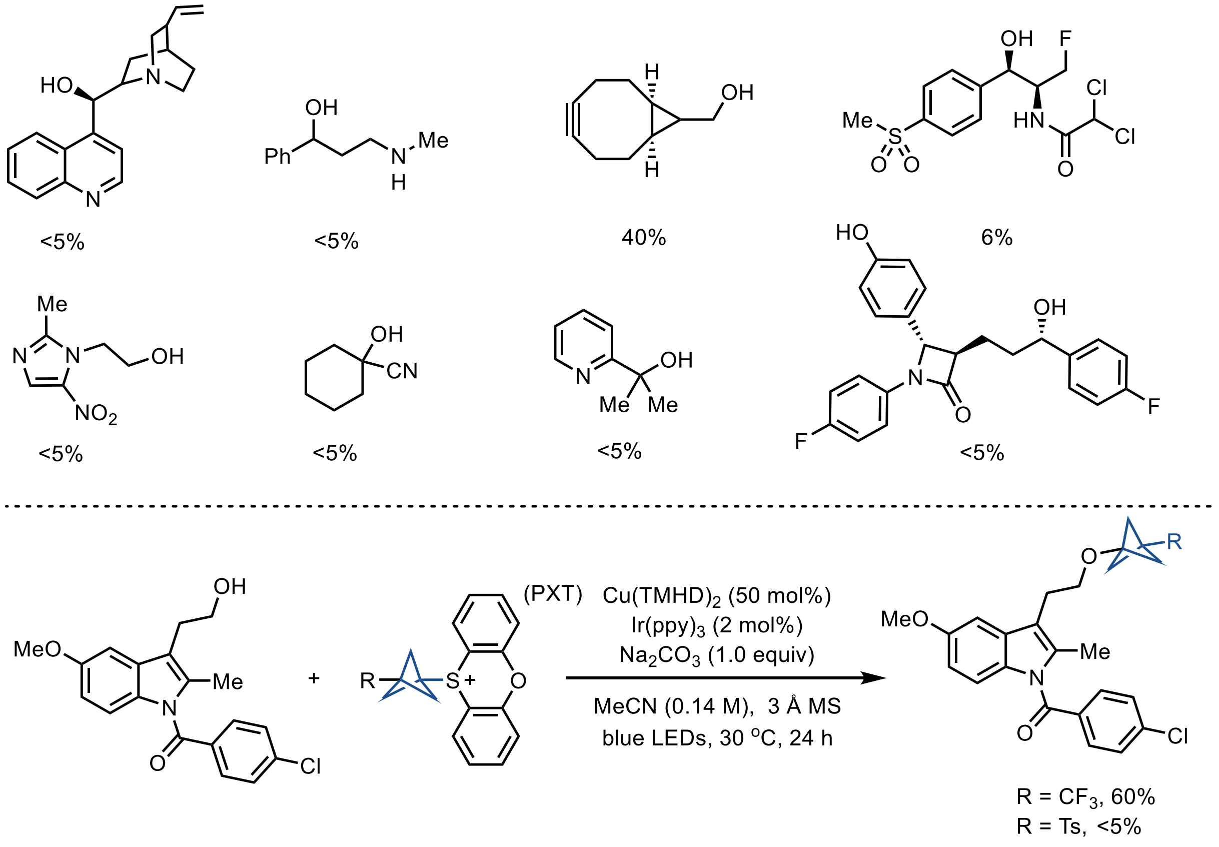

Figure S1. Unsuccessful substrates. ^{19}F NMR yield using $PhCF_3$ as an internal standard.

Mechanistic investigations

Radical trapping experiment

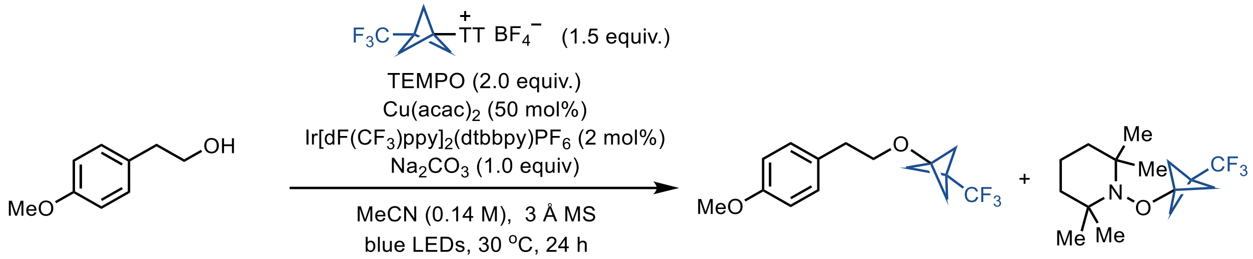

1a, 1.0 equiv. **1**, <3% detected by HRMS

Under nitrogen atmosphere, to a 4 mL borosilicate vial equipped with a magnetic stir bar were added 4-methoxyphenethyl acohol (15.2 mg, 0.100 mmol, 1.00 equiv.), CF_3BCP-TT^+ BF_4^- (65.7 mg, 0.150 mmol, 1.50 equiv.), Ir[dF(CF_3)ppy]$_2$(dtbbpy)PF_6 (2 mg, 2 µmoL, 2 mol%), Cu(acac)$_2$ (13 mg, 50 µmoL, 50 mol%), Na_2CO_3 (10.6 mg, 0.100 mmol, 1.00 equiv.), 3Å molecular sieves (120 mg), TEMPO (31.3 mg, 0.200 mmol, 2.00 equiv.), and anhydrous MeCN (0.70 mL, *c* = 0.14 M). The vial was sealed with a septum-cap. Then, the mixture was stirred for 1 h at 25 °C, and placed 5 cm away from two blue LEDs (Kessil A160WE Tuna Blue (460 nm), LED lighting, 40 W). The mixture was irradiated for 24 h while maintaining the temperature at approximately 30 °C through cooling with a fan. After irradiation, the mixture was analyzed by HRMS. The TEMPO-BCP adduct was observed.

HRMS-ESI (m/z) calc'd for $C_{15}H_{25}NOF_3^+$ $[M+H]^+$, 292.1883; found, 292.1880; deviation: +0.8 ppm.

Stern-Volmer luminiscence quenching studies

Visible light luminescence intensities were recorded using an Edinburgh Instruments FS5 spectrofluorometer. All luminescence measurements were recorded using a screw-top quartz cuvette (Hellma fluorescence quartz cuvette, 10 x 10 mm, 3.5 mL). All solutions of Ir(dF(CF_3)ppy)$_2$(dtbbpy)PF_6, **1b**, thianthrene, and Cu(acac)$_2$ were prepared in MeCN in a nitrogen-filled glovebox. The solutions were transferred to the screw-top cuvette inside the glovebox, the cuvette was sealed, and then, brought out of the glovebox for visible light luminescence measurements.

In a typical procedure, **1b** (132 mg, 0.300 mmol) was dissolved and diluted to a final volume of 10 mL (*c* = 0.040 M) with a stock solution of Ir(dF(CF_3)ppy)$_2$(dtbbpy)PF_6 in MeCN (*c* = 16 µM). Serial dilution of this 0.030 M **1b** solution was carried out by dilution of 7 mL of the 0.030 M **1b** solution to 10 mL (0.021 M) with the 16 µM stock solution of Ir(dF(CF_3)ppy)$_2$(dtbbpy)PF_6. All subsequent solutions were prepared by dilution of 7 mL of the preceding solution to a final volume of 10 mL. All solutions were excited at 450 nm and the emission was measured from 455 to 600 nm.

Quenching was analyzed by plotting I_0/I according to the Stern-Volmer relationship:

$I_o/I = k_q\tau_o[Q]+1$

where I_o represents the integral of the luminescence over the range of 455 to 600 nm in the absence of a quencher, I is the integral of luminiscense over the range of 455 to 600 nm in the presence of a quencher, k_q represents the quenching rate constant, [Q] is the concentration of a given quencher, and τ_o is the excited state lifetime of the emissive photocatalyst in the absence of quencher. The excited state lifetime of $Ir(dF(CF_3)ppy)_2(dtbbpy)PF_6$ in MeCN is 2300 ns.[4]

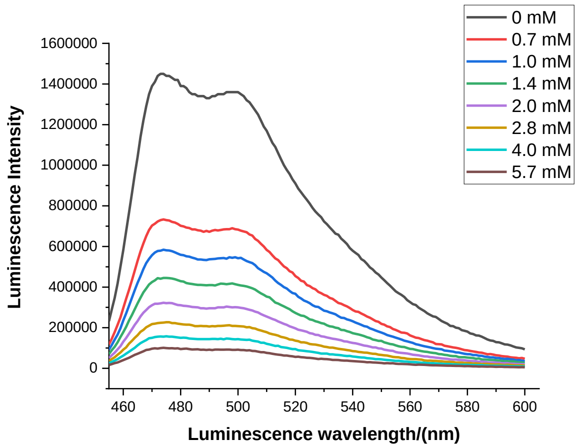

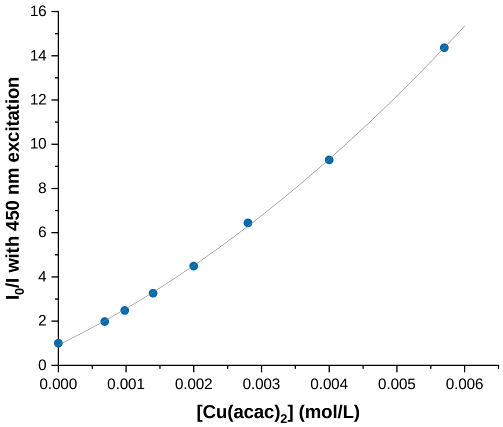

Figure S2. Emission spectra for $Ir(dF(CF_3)ppy)_2(dtbbpy)PF_6$ luminescence quenching by $Cu(acac)_2$ (left) and Stern-Volmer plot (right). A non-linear relationship was obsearved due to the inner filter effect.

Excitation inner filter effect correction factors, η, were calculated using the equation:[5]

$$\eta = \frac{A_{xo}(1-10^{-A_{xi}})}{A_{xi}(1-10^{-A_{xo}})}$$

Where A_{xo} is the fluorophore absorbances (0.008 a.u.) at the excitation wavelength (450 nm), and A_{xi} is total absorbances of the fluorophore and the quencher at a given concentration.

Table S4: Calculated correction factors and corrected I_0/I values for $Cu(acac)_2$.

$[Cu^{II}]$/(mM)	uncorrected I_0/I	A_{xi}	η	corrected I_0/I
0	1	0.008	1	1
0.69	1.98	0.019	0.99	1.96
0.98	2.48	0.027	0.98	2.43
1.4	3.26	0.033	0.97	3.16
2.0	4.49	0.045	0.96	4.31
2.8	6.44	0.065	0.94	6.05

4.0	9.29	0.093	0.91	8.45
5.7	14.36	0.138	0.86	12.35

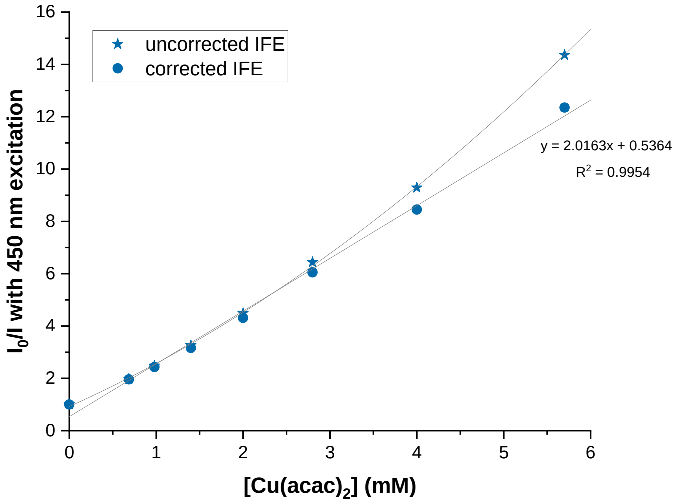

Figure S3. Stern-Volmer plot of $Cu(acac)_2$ with corrections. IFE, inner filter effect.

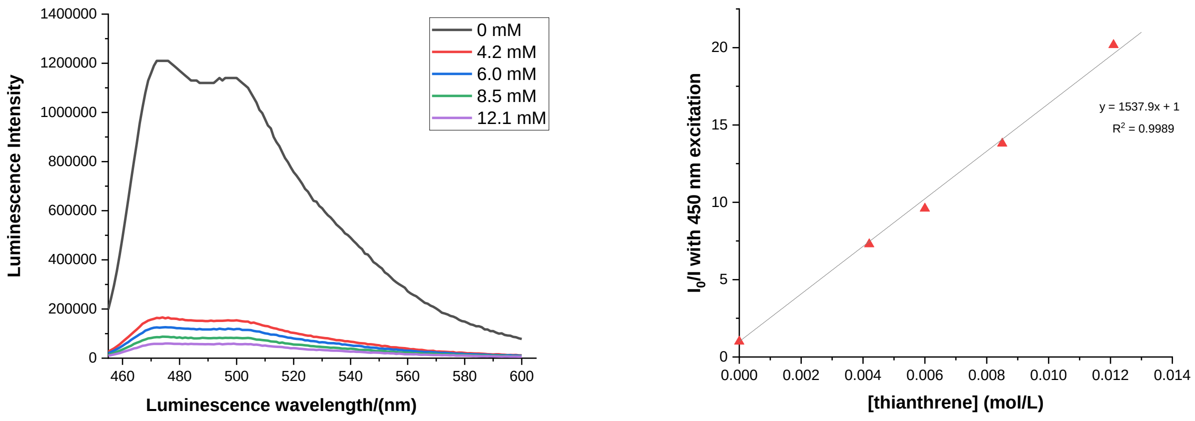

Figure S4. Emission spectra for $Ir(dF(CF_3)ppy)_2(dtbbpy)PF_6$ luminescence quenching by thianthrene (left) and Stern-Volmer plot (right).

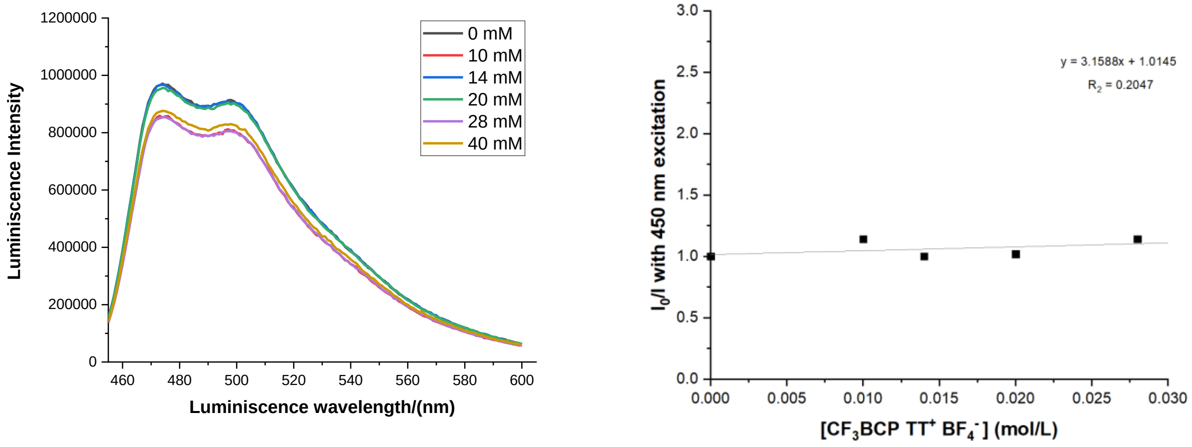

Figure S5. Emission spectra for Ir(dF(CF$_3$)ppy)$_2$(dtbbpy)PF$_6$ luminescence quenching by CF$_3$BCP-TT BF$_4$ (**1b**) (left) and Stern-Volmer plot (right).

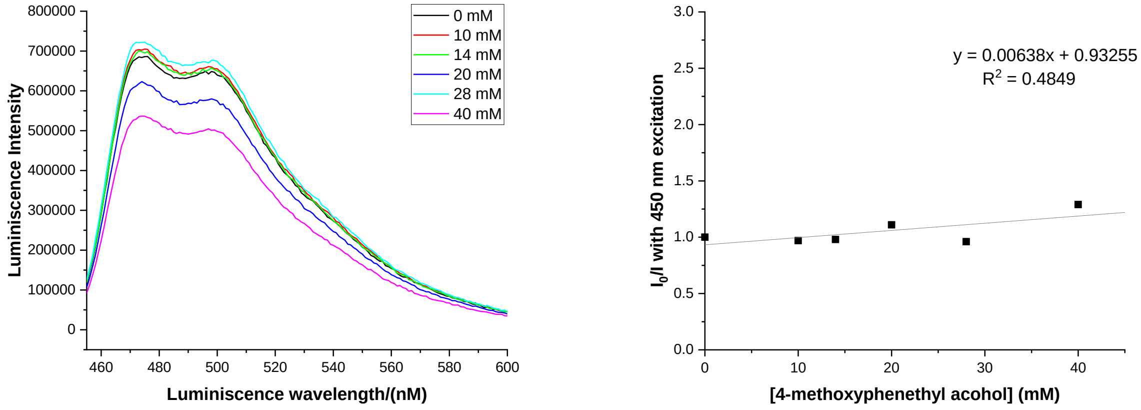

Figure S6. Emission spectra for Ir(dF(CF$_3$)ppy)$_2$(dtbbpy)PF$_6$ luminescence quenching by 4-methoxyphenethyl acohol (left) and Stern-Volmer plot (right).

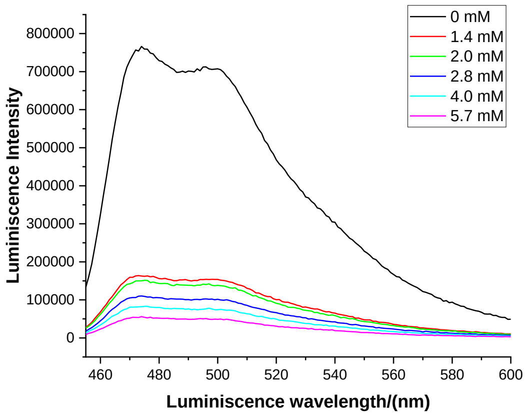

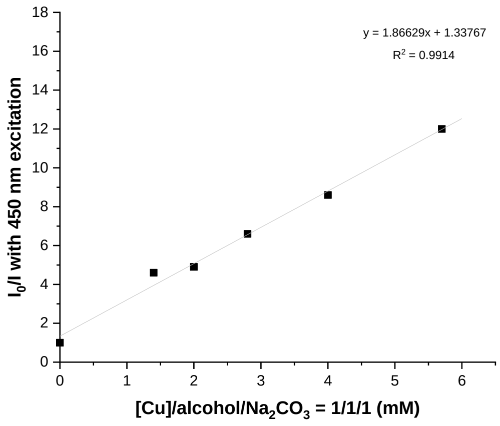

Figure S7. Emission spectra for Ir(dF(CF$_3$)ppy)$_2$(dtbbpy)PF$_6$ luminescence quenching by Cu(acac)$_2$/4-methoxyphenethyl acohol/Na$_2$CO$_3$ = 1/1/1 (left) and Stern-Volmer plot (right) with corrections.

Table S5. Quencher rate coefficients:

Quencher	$K_q/(M^{-1}S^{-1})$
Cu(acac)$_2$	8.8*10^8
thianthrene	6.7*10^8
CF$_3$BCP-TT BF$_4$	1.3*10^5
4-methoxyphenethyl acohol	2.8*10^6
Cu(acac)$_2$/4-methoxyphenethyl acohol/Na$_2$CO$_3$	8.1*10^8

EPR analysis

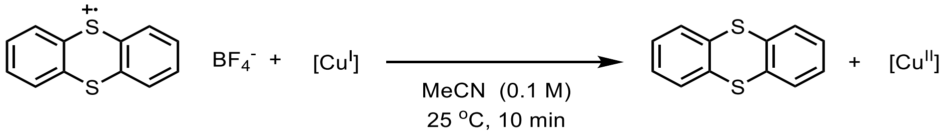

Under nitrogen atmosphere, to a 4 mL borosilicate vial equipped with a magnetic stir bar were added thianthrenium tetrafluoroborate (TT$^{\bullet+}$ BF$_4^-$) (15 mg, 0.050 mmol, 1.0 equiv.), Cu^I cayalyst (0.050 mmol, 1.0 equiv.), and anhydrous MeCN (0.50 mL, *c* = 0.10 M). After 10 min, the reaction mixture was transferred to an oven-dried EPR tube. Then, outside the glovebox, the sample was frozen to 77 K with liquid nitrogen and introduced into the EPR instrument. Intense signals consistent with a Cu(II) catalyst were detected, while the signal of TT radical cation was not observed.[6]

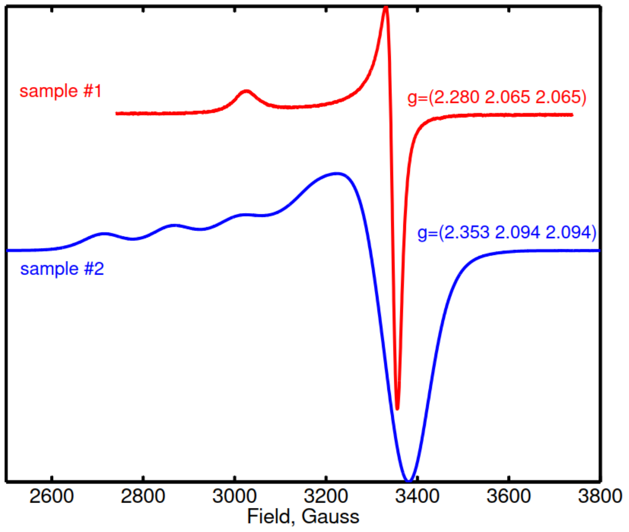

Figure S8. EPR spectra. **Sample 1** with CuCN; **Sample 2** with $Cu(MeCN)_4BF_4$

Analysis of pre-stirring the catalyst and alcohol

Under nitrogen atmosphere, to a 4 mL borosilicate vial equipped with a magnetic stir bar were added 4-methoxyphenethyl acohol (15.2 mg, 0.100 mmol, 1.00 equiv.), $Cu(acac)_2$ (26.0 mg, 0.100 mmol, 1.00 equiv.), Na_2CO_3 (10.6 mg, 0.100 mmol, 1.00 equiv.), 3Å molecular sieves (120 mg), and anhydrous *d*3-MeCN (0.70 mL, *c* = 0.14 M). The vial was sealed with a septum-cap. Then, the mixture was stirred for 1 h at 25 °C, and transferred into the NMR tube by a syringe with a syringe filter for ^{1}H NMR analysis. No significant Cu-alkoxide was observed.

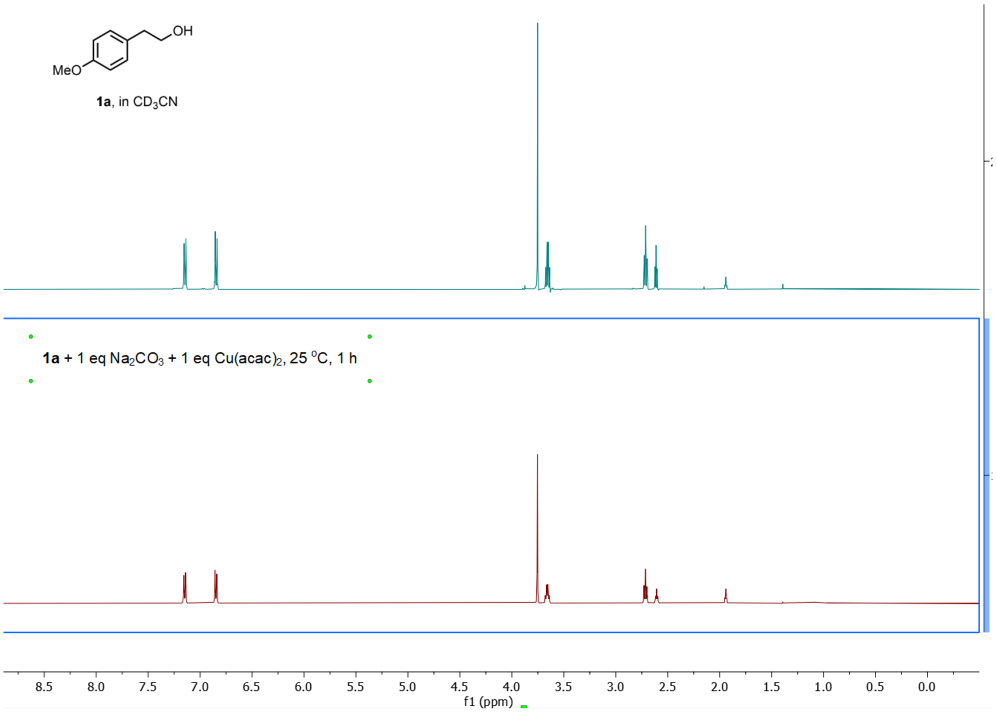

Figure S9. NMR spectra of pre-stirring mixture of [Cu], alcohol, and base.

Alternative mechanism without TT involved

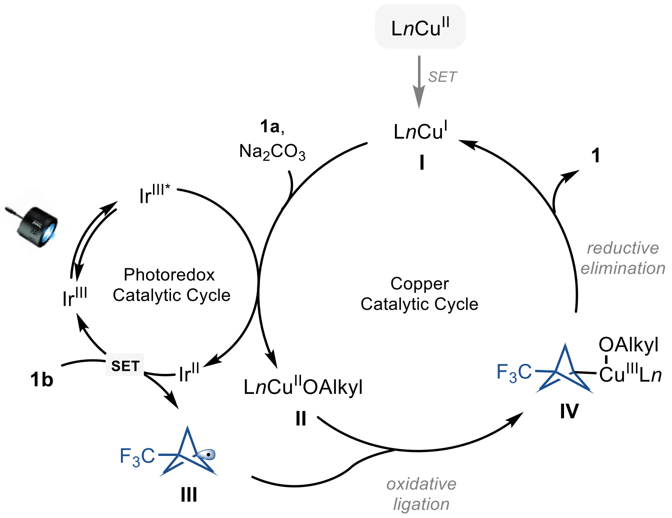

Figure S10. Alternative mechanism.

Unsuccessful attempts to other substituted BCP thianthrenium reagents

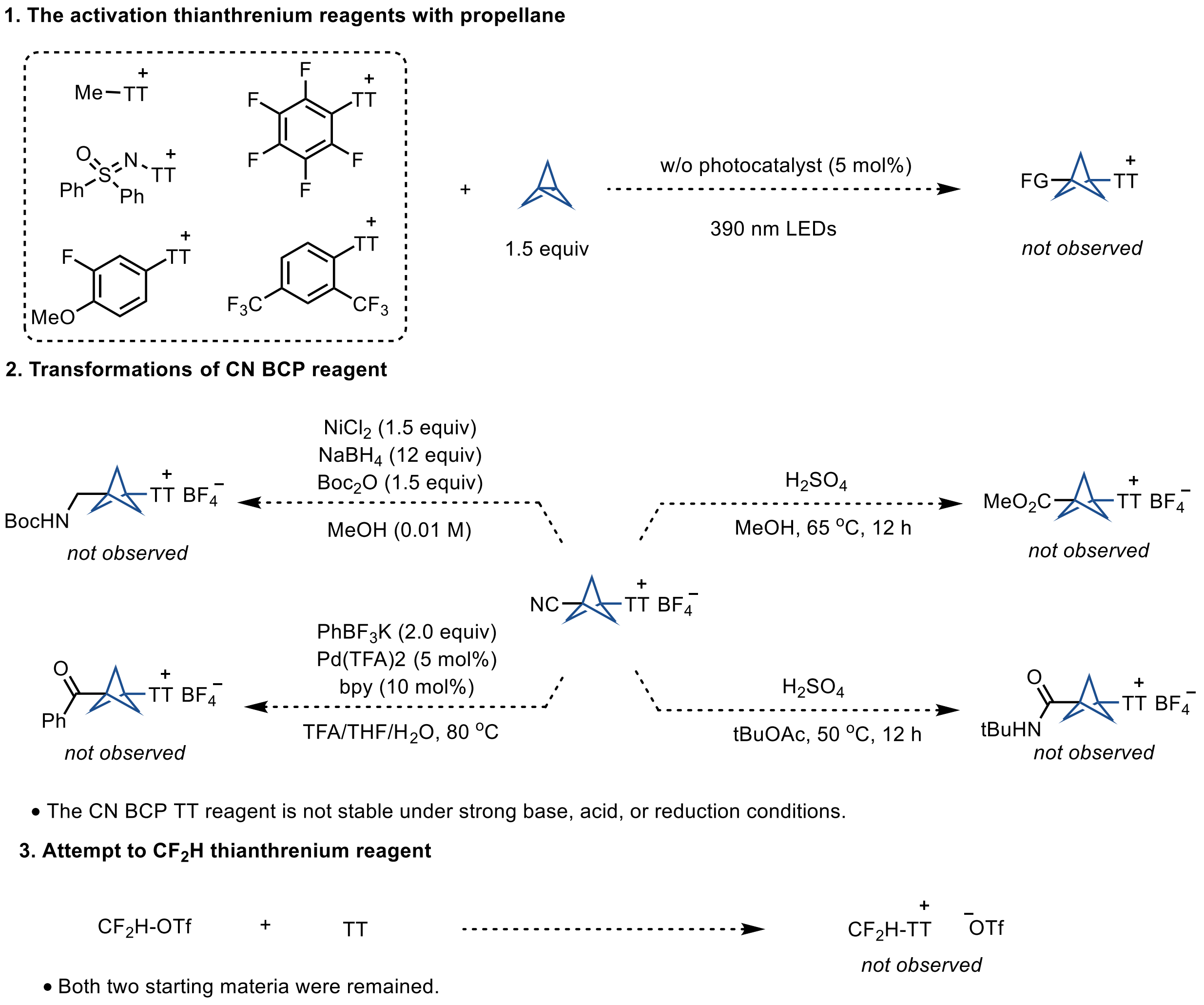

Figure S11. Attempts to prepare other substituted BCP reagents were unsuccessful.

DFT calculations

Methods

Density Functional Theory (DFT) calculations were performed on the Max-Planck-Institut für Kohlenforschung computer cluster using the ORCA 5.0 program package (Version 5.0 Stable).[7] Structural optimizations and frequency calculations were performed with the B3LYP functional[8] with D3 dispersion correction[9] and Becke-Johnson damping (BJ),[10] utilizing the def2/J auxiliary basis set[11] and the def2-SVP basis set[12] on all atoms. Tight SCF convergence and geometry optimization criterions were chosen. Frequency calculations at the same level had been performed to confirm each stationary point to be either a minimum or a transition structure. Transition states were found by performing relaxed surface scans to find the saddlepoint and an eigenvector-following saddlepoint optimization. Solvent effects of acetonitrile were taken into account using the conductor-

like polarized continuum model (CPCM).[13] To obtain more accurate energies, single-point energy calculations were performed on all optimized structures applying the def2/J auxiliary basis set and the def2-TZVPP basis set[12] for all atoms. Input files were created using Avogadro 1.2[14] and images were generated using Chemcraft 1.8.[15] BDE = bond dissociation energy.

Summary of calculation results

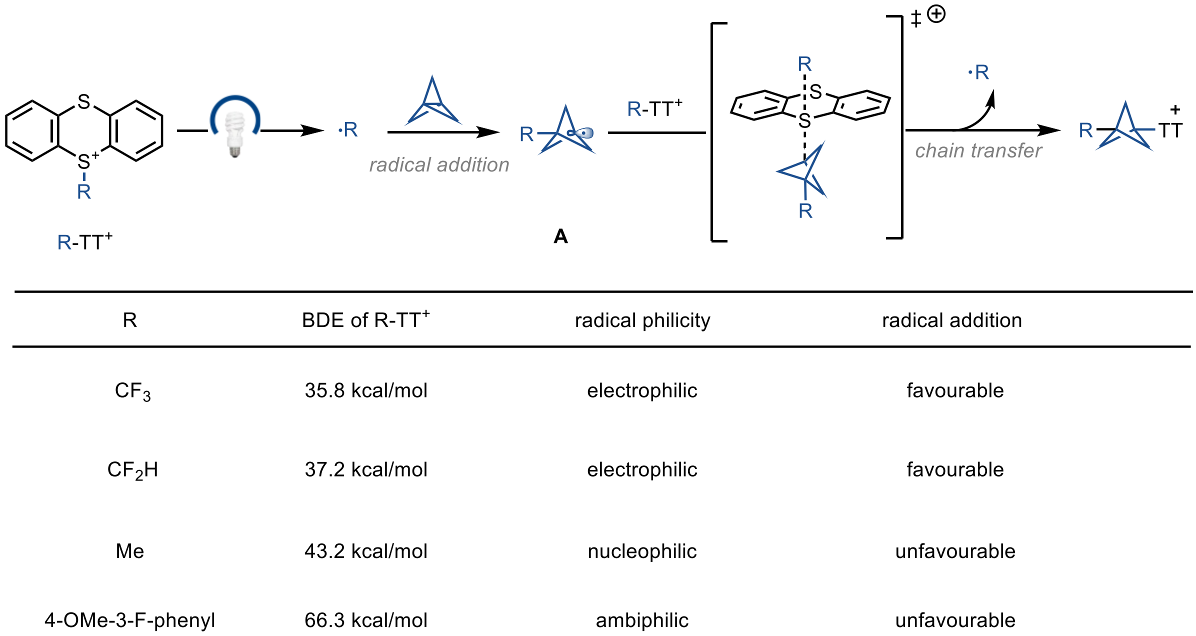

R-TT+ A

R	BDE of R-TT+	radical philicity	radical addition
CF_3	35.8 kcal/mol	electrophilic	favourable
CF_2H	37.2 kcal/mol	electrophilic	favourable
Me	43.2 kcal/mol	nucleophilic	unfavourable
4-OMe-3-F-phenyl	66.3 kcal/mol	ambiphilic	unfavourable

Figure S12. Summary of calculation results

Table S6. Thermodynamic quantities of C1-C3.

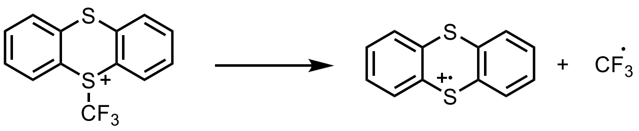

C1 **C2** **C3**

	**Enthalpy H (Ha)**
**CF_3-TT C1**	-1594.36024521
**TT-radical$^+$ C2**	-1257.14139361
**CF_3-radical C3**	-337.16180323

Table S7. Thermodynamic quantities of C4-C5.

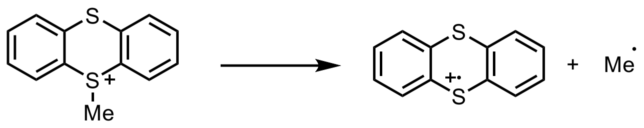

C4 C2 C5

	Enthalpy H (Ha)
**Me-TT C4**	-1296.95535718
**TT-radical⁺ C2**	-1257.14139361
**Me-radical C5**	-39.74510269

Table S8. Thermodynamic quantities of C6-C7.

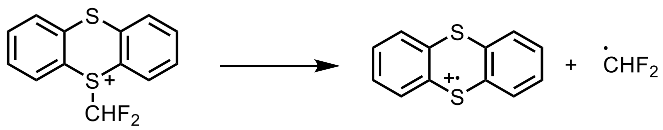

C6 C2 C7

	Enthalpy H (Ha)
**CF_2H-TT C6**	-1495.21574791
**TT-radical⁺ C2**	-1257.14139361
**CF_2H-radical C7**	-238.01499345

Table S9. Thermodynamic quantities of C8-C9.

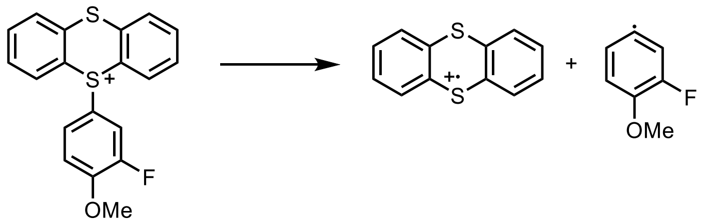

C8 C2 C9

	Enthalpy H (Ha)
**Ar-TT C8**	-1701.89693576
**TT-radical⁺ C2**	-1257.14139361

**Ar-radical C9**	-444.64984725

Table S10. Thermodynamic quantities of CF_3-BCP **TS1**.

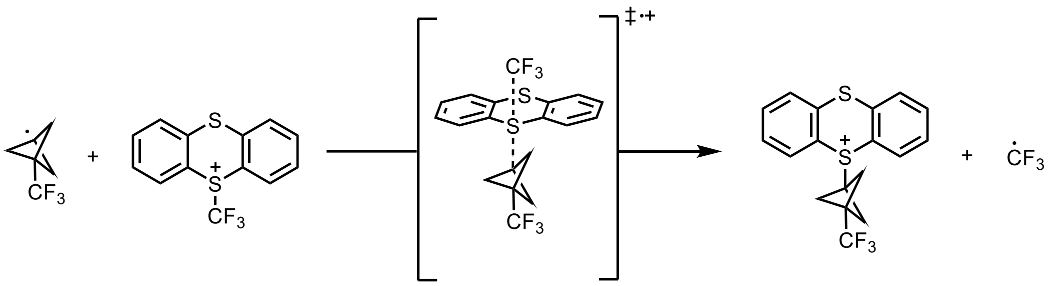

C10 **C1** **TS1**

	Free energy G (Ha)	ΔG‡ (kcal/mol)
**CF_3-BCP radical C10**	-531,622367824147	
**C1**	-1595,81084968894	
**TS1**	-2127,44515976021	6.5

Table S11. Thermodynamic quantities of Me-BCP **TS2**.

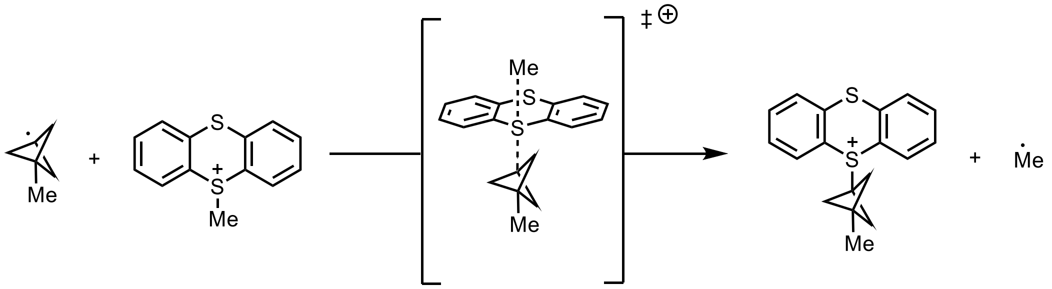

C11 **C4** **TS2**

	Free energy G (Ha)	ΔG‡ (kcal/mol)
**Me-BCP radical C11**	-233,849454100535	
**C4**	-1298,0712482623	
**TS2**	-1531,92250722457	11.1

CF_3-TT C1

S	-0.964008000000	2.538722000000	-1.150680000000
C	-2.361123000000	1.809250000000	-0.357657000000
C	-2.378137000000	0.584108000000	0.335494000000
S	-0.959424000000	-0.458742000000	0.458523000000
C	0.454242000000	0.591529000000	0.341212000000
C	0.433678000000	1.816573000000	-0.352064000000
C	1.638778000000	2.534702000000	-0.437239000000
C	2.803677000000	2.041180000000	0.146937000000
C	2.800059000000	0.830347000000	0.852800000000
C	1.619237000000	0.103899000000	0.958438000000
C	-3.569643000000	2.520998000000	-0.447794000000
C	-4.734342000000	2.021221000000	0.131454000000
C	-4.727286000000	0.810307000000	0.837139000000
C	-3.543064000000	0.090136000000	0.947692000000
H	-3.515571000000	-0.857860000000	1.489520000000
H	-3.584922000000	3.481783000000	-0.967090000000
H	-5.659892000000	2.594710000000	0.045061000000
H	-5.640495000000	0.432418000000	1.299622000000
H	1.651132000000	3.495513000000	-0.956569000000
H	3.726538000000	2.619556000000	0.064342000000
H	3.713272000000	0.457379000000	1.319261000000
H	1.594567000000	-0.844092000000	1.500373000000
C	-0.954628000000	-1.340062000000	-1.252032000000
F	-2.041800000000	-2.080762000000	-1.287446000000
F	-0.953202000000	-0.468715000000	-2.236266000000
F	0.133597000000	-2.079238000000	-1.283011000000

TT-radical^{+} C2

S	-1.013873000000	4.488901000000	1.986554000000
C	-2.411723000000	4.559367000000	0.951733000000
C	-2.417976000000	4.919696000000	-0.421375000000
S	-1.028887000000	5.367830000000	-1.369171000000
C	0.369006000000	5.296707000000	-0.334604000000
C	0.375371000000	4.936371000000	1.038501000000
C	1.585130000000	5.637983000000	-0.966949000000
C	2.773135000000	5.621467000000	-0.255288000000
C	2.779533000000	5.262906000000	1.108159000000
C	1.597656000000	4.924734000000	1.746233000000
C	-3.640230000000	4.931034000000	-1.129253000000
C	-4.822103000000	4.592721000000	-0.491286000000
C	-4.815762000000	4.234193000000	0.872168000000
C	-3.627801000000	4.217840000000	1.583938000000
H	-3.643855000000	5.210682000000	-2.185538000000
H	-5.760527000000	4.604738000000	-1.049321000000
H	-5.749077000000	3.967339000000	1.371715000000
H	-3.621653000000	3.939242000000	2.640429000000
H	1.578887000000	5.916450000000	-2.023487000000
H	3.706482000000	5.888198000000	-0.754847000000
H	3.717988000000	5.250732000000	1.666131000000
H	1.601439000000	4.644949000000	2.802468000000

CF_3-radical C3

F	-1.946576000000	3.187002000000	-0.092973000000
C	-1.651507000000	1.962256000000	0.296037000000
F	-2.548652000000	1.093339000000	-0.126424000000

F	-0.434485000000	1.617883000000	-0.076640000000

Me-TT C4

S	-1.237266000000	3.254607000000	1.416330000000
C	-2.630615000000	2.472540000000	0.615921000000
C	-2.692530000000	1.077293000000	0.478736000000
S	-1.320910000000	0.206623000000	1.254179000000
C	0.084007000000	1.002243000000	0.457881000000
C	0.099987000000	2.398684000000	0.595902000000
C	1.195664000000	3.108940000000	0.092560000000
C	2.243314000000	2.422693000000	-0.526906000000
C	2.197354000000	1.032852000000	-0.675037000000
C	1.108153000000	0.306404000000	-0.185841000000
C	-1.370846000000	-1.452926000000	0.534438000000
H	-0.487499000000	-1.984158000000	0.911780000000
H	-1.379838000000	-1.400707000000	-0.561719000000
H	-2.273780000000	-1.937102000000	0.928130000000
C	-3.762247000000	0.438182000000	-0.149668000000
C	-4.817185000000	1.222679000000	-0.623953000000
C	-4.785328000000	2.612975000000	-0.476347000000
C	-3.693194000000	3.241255000000	0.127679000000
H	-3.653186000000	4.329241000000	0.213913000000
H	-5.668431000000	0.740236000000	-1.108565000000
H	-5.613909000000	3.217851000000	-0.850830000000
H	-3.801292000000	-0.645769000000	-0.263243000000
H	1.216168000000	4.197417000000	0.179558000000
H	3.098335000000	2.981832000000	-0.912899000000
H	3.014190000000	0.505305000000	-1.171756000000

H	1.086293000000	-0.777869000000	-0.300224000000

Me-radical C5

H	-1.966890000000	3.008074000000	-0.012982000000
C	-1.645288000000	1.965425000000	-0.000160000000
H	-2.387306000000	1.165772000000	0.012513000000
H	-0.581736000000	1.721209000000	0.000629000000

CF_2H-TT C6

S	-0.968951000000	2.528661000000	-1.148944000000
C	-2.370301000000	1.802929000000	-0.360054000000
C	-2.394448000000	0.572138000000	0.323067000000
S	-0.981111000000	-0.484438000000	0.430287000000
C	0.437236000000	0.567216000000	0.337132000000
C	0.424309000000	1.795704000000	-0.348959000000
C	1.630775000000	2.512060000000	-0.423377000000
C	2.792330000000	2.011221000000	0.162055000000
C	2.781628000000	0.796016000000	0.858889000000
C	1.597493000000	0.072260000000	0.954668000000
C	-3.571812000000	2.528622000000	-0.435901000000
C	-4.737326000000	2.040198000000	0.151131000000
C	-4.736105000000	0.828291000000	0.854515000000
C	-3.558229000000	0.094529000000	0.950230000000
H	-3.535448000000	-0.849908000000	1.499083000000
H	-3.579634000000	3.493073000000	-0.948525000000
H	-5.656846000000	2.624881000000	0.075976000000
H	-5.647415000000	0.461211000000	1.329406000000
H	1.647442000000	3.476577000000	-0.935602000000

H	3.717174000000	2.587449000000	0.087113000000
H	3.691723000000	0.416011000000	1.325798000000
H	1.568495000000	-0.879361000000	1.489654000000
C	-0.947615000000	-1.335539000000	-1.298462000000
H	-1.766096000000	-2.076788000000	-1.298231000000
F	-1.103078000000	-0.434503000000	-2.253660000000
F	0.247048000000	-1.903647000000	-1.377268000000

CF_2H-radical C7

H	-2.080142000000	3.004705000000	-0.002843000000
C	-1.646083000000	2.021579000000	-0.246594000000
F	-2.435958000000	0.994763000000	0.030432000000
F	-0.419038000000	1.839432000000	0.219005000000

Ar-TT C8

C	-0.615397000000	1.884077000000	0.641790000000
C	-1.742530000000	1.109691000000	0.337007000000
C	-1.549744000000	-0.120208000000	-0.266061000000
C	-0.253776000000	-0.612106000000	-0.564864000000
C	0.849823000000	0.189603000000	-0.234099000000
C	0.674515000000	1.436844000000	0.374179000000
H	1.547435000000	2.045083000000	0.614909000000
H	-2.762614000000	1.441019000000	0.535141000000
H	1.860464000000	-0.153060000000	-0.453175000000
S	-0.854173000000	3.436091000000	1.541283000000
C	-2.327627000000	4.098663000000	0.795700000000
C	-2.327973000000	4.467076000000	-0.560942000000
S	-0.892374000000	4.390807000000	-1.598179000000

C	0.404078000000	4.846147000000	-0.478802000000
C	0.426659000000	4.476922000000	0.877536000000
C	1.479441000000	5.597983000000	-0.974338000000
C	2.527774000000	5.967337000000	-0.129035000000
C	2.516480000000	5.619282000000	1.226952000000
C	1.455786000000	4.872774000000	1.739792000000
C	-3.537130000000	4.896312000000	-1.126728000000
C	-4.694620000000	4.957379000000	-0.347903000000
C	-4.668113000000	4.618031000000	1.009910000000
C	-3.475250000000	4.189682000000	1.592017000000
O	-0.203990000000	-1.809530000000	-1.146467000000
C	1.056001000000	-2.384317000000	-1.465144000000
H	1.605746000000	-1.759598000000	-2.189440000000
H	0.840813000000	-3.360556000000	-1.915093000000
H	1.668028000000	-2.525829000000	-0.558509000000
H	-3.560853000000	5.202100000000	-2.174671000000
H	-5.627029000000	5.295789000000	-0.804986000000
H	-5.574530000000	4.686142000000	1.614373000000
H	-3.435201000000	3.910878000000	2.647332000000
H	1.478345000000	5.910967000000	-2.020549000000
H	3.355563000000	6.555280000000	-0.532158000000
H	3.331039000000	5.929372000000	1.884125000000
H	1.430773000000	4.584699000000	2.793095000000
F	-2.598037000000	-0.869057000000	-0.583397000000

Ar-radical C9

C	-0.644414000000	1.780808000000	0.628043000000
C	-1.657470000000	0.904234000000	0.284144000000

C	-1.263656000000	-0.301749000000	-0.291372000000
C	0.098135000000	-0.610552000000	-0.510823000000
C	1.071043000000	0.326649000000	-0.137214000000
C	0.699395000000	1.554793000000	0.447577000000
H	1.465083000000	2.279112000000	0.737016000000
H	-2.720755000000	1.105065000000	0.438424000000
H	2.128547000000	0.112587000000	-0.297430000000
O	0.345438000000	-1.814745000000	-1.076712000000
F	-2.184125000000	-1.201777000000	-0.651851000000
C	1.682541000000	-2.182516000000	-1.324987000000
H	2.177968000000	-1.481033000000	-2.021169000000
H	1.652149000000	-3.179950000000	-1.782977000000
H	2.273682000000	-2.232465000000	-0.39230800000

CF_3-BCP radical C10

C	-0.207451000000	-1.868619000000	0.213951000000
C	-1.147554000000	-2.988578000000	0.719080000000
C	0.097206000000	-3.625035000000	0.047491000000
H	-2.102654000000	-3.120924000000	0.193641000000
H	-1.234591000000	-3.121411000000	1.805910000000
C	-0.029173000000	-2.619045000000	-1.126361000000
H	-0.916251000000	-2.729791000000	-1.764210000000
H	0.888687000000	-2.421837000000	-1.696582000000
C	1.009028000000	-2.621162000000	0.801058000000
H	1.054420000000	-2.733782000000	1.892803000000
H	1.990488000000	-2.423349000000	0.349640000000
C	0.355925000000	-5.107571000000	-0.091614000000
F	-0.615477000000	-5.704297000000	-0.802580000000

F	0.409915000000	-5.704434000000	1.110306000000
F	1.522453000000	-5.338266000000	-0.717214000000

TS1

F	4.087169000000	0.474444000000	1.520531000000
C	3.835223000000	-0.394630000000	0.536776000000
F	4.765263000000	-0.249055000000	-0.411223000000
F	3.916946000000	-1.634099000000	1.030295000000
C	2.451090000000	-0.148547000000	-0.039423000000
C	1.979440000000	1.175984000000	-0.707372000000
C	1.123232000000	-0.218438000000	0.769289000000
C	0.855322000000	0.135420000000	-0.699556000000
S	-1.210670000000	0.503979000000	-1.497881000000
C	-1.712060000000	-1.084778000000	-0.863704000000
C	-1.473874000000	-2.151814000000	-1.742093000000
C	-1.827483000000	-3.445133000000	-1.369420000000
C	-2.411489000000	-3.661773000000	-0.115183000000
C	-2.637141000000	-2.598768000000	0.754719000000
C	-2.292024000000	-1.281664000000	0.395823000000
S	-2.655294000000	-0.063946000000	1.619390000000
C	-2.026529000000	1.471720000000	1.020677000000
C	-1.446112000000	1.732209000000	-0.227028000000
C	-0.983703000000	3.009766000000	-0.574269000000
C	-1.105436000000	4.058338000000	0.332385000000
C	-1.684955000000	3.818323000000	1.584458000000
C	-2.136069000000	2.546109000000	1.924526000000
C	1.797262000000	-0.967920000000	-1.190586000000
H	1.868476000000	2.038710000000	-0.039768000000

H	2.436441000000	1.419878000000	-1.674085000000
H	0.814544000000	-1.213809000000	1.109582000000
H	0.963172000000	0.572489000000	1.511353000000
H	-1.023619000000	-1.963672000000	-2.719609000000
H	-1.652334000000	-4.276893000000	-2.053949000000
H	-2.695074000000	-4.671101000000	0.190013000000
H	-3.089508000000	-2.780851000000	1.732620000000
H	-0.540198000000	3.180971000000	-1.557993000000
H	-0.753160000000	5.055461000000	0.063246000000
H	-1.786506000000	4.631823000000	2.306090000000
H	-2.584025000000	2.370555000000	2.905649000000
H	2.244620000000	-0.846321000000	-2.184600000000
H	1.524897000000	-2.002265000000	-0.950057000000
C	-3.049501000000	0.881380000000	-2.393724000000
F	-3.191532000000	-0.001765000000	-3.363073000000
F	-2.993770000000	2.107465000000	-2.876501000000
F	-4.025622000000	0.769473000000	-1.513206000000

Me-BCP radical C11

C	-0.205104000000	-1.882564000000	0.212518000000
C	-1.140917000000	-3.000797000000	0.715519000000
C	0.103649000000	-3.659591000000	0.044297000000
H	-2.101630000000	-3.126334000000	0.194998000000
H	-1.234790000000	-3.126836000000	1.804323000000
C	-0.026748000000	-2.632920000000	-1.122683000000
H	-0.913537000000	-2.734940000000	-1.765508000000
H	0.888105000000	-2.426980000000	-1.697971000000
C	1.007212000000	-2.634440000000	0.797417000000

H	1.056589000000	-2.738019000000	1.891678000000
H	1.991239000000	-2.428179000000	0.351217000000
C	0.362943000000	-5.149169000000	-0.095658000000
H	-0.438765000000	-5.629353000000	-0.679570000000
H	0.405901000000	-5.629885000000	0.894571000000
H	1.320834000000	-5.328092000000	-0.609819000000

TS2

C	2.599919000000	-0.189390000000	0.100809000000
C	2.079812000000	1.136502000000	-0.552461000000
C	1.242590000000	-0.299210000000	0.876511000000
C	0.973791000000	0.082178000000	-0.584168000000
S	-0.937830000000	0.423376000000	-1.439645000000
C	-1.519596000000	-1.174556000000	-0.885734000000
C	-1.361482000000	-2.201905000000	-1.825327000000
C	-1.750203000000	-3.500833000000	-1.507407000000
C	-2.301493000000	-3.762439000000	-0.247746000000
C	-2.457000000000	-2.737489000000	0.681188000000
C	-2.061877000000	-1.419513000000	0.380686000000
S	-2.295337000000	-0.262507000000	1.695125000000
C	-1.863468000000	1.331989000000	1.068550000000
C	-1.319790000000	1.638318000000	-0.183722000000
C	-0.991376000000	2.953569000000	-0.536958000000
C	-1.207173000000	3.990890000000	0.366807000000
C	-1.757418000000	3.703788000000	1.621330000000
C	-2.083095000000	2.395027000000	1.966020000000
C	1.948321000000	-0.984706000000	-1.081597000000
H	1.936938000000	1.989872000000	0.123735000000

H	2.533520000000	1.417880000000	-1.512170000000
H	0.939787000000	-1.305382000000	1.193153000000
H	1.047955000000	0.468801000000	1.635671000000
H	-0.930038000000	-1.982574000000	-2.805372000000
H	-1.626122000000	-4.301427000000	-2.238872000000
H	-2.617612000000	-4.774371000000	0.013683000000
H	-2.892359000000	-2.950394000000	1.660692000000
H	-0.562084000000	3.163075000000	-1.520097000000
H	-0.948552000000	5.015144000000	0.092624000000
H	-1.937722000000	4.507034000000	2.338852000000
H	-2.515919000000	2.180526000000	2.946274000000
H	2.393959000000	-0.834168000000	-2.074103000000
H	1.687739000000	-2.031961000000	-0.879299000000
C	3.976711000000	-0.419033000000	0.685291000000
H	4.748091000000	-0.273949000000	-0.085879000000
H	4.060730000000	-1.444069000000	1.076270000000
H	4.165673000000	0.286435000000	1.508159000000
C	-2.710761000000	0.819868000000	-2.515370000000
H	-2.717321000000	0.068605000000	-3.312559000000
H	-2.582706000000	1.845438000000	-2.877882000000
H	-3.514703000000	0.701254000000	-1.780661000000

CF_2H-BCP radical C12

C	0.160291000000	-3.143281000000	0.072654000000
C	-0.901276000000	-4.154289000000	0.566614000000
C	0.274012000000	-4.925567000000	-0.091642000000
H	-1.862467000000	-4.173519000000	0.033251000000
H	-1.015468000000	-4.276384000000	1.652183000000

C	0.269696000000	-3.911017000000	-1.265306000000
H	-0.617398000000	-3.914098000000	-1.914887000000
H	1.208577000000	-3.815383000000	-1.827262000000
C	1.280818000000	-4.020163000000	0.672556000000
H	1.302159000000	-4.136563000000	1.764494000000
H	2.283239000000	-3.932134000000	0.232574000000
C	0.389860000000	-6.427291000000	-0.216877000000
H	-0.443357000000	-6.869593000000	-0.792844000000
F	0.405092000000	-6.991896000000	1.019872000000
F	1.557742000000	-6.747022000000	-0.836911000000

Ar-BCP radical C13

C	0.197749000000	-3.208271000000	0.038341000000
C	-0.848216000000	-4.199788000000	0.587775000000
C	0.304361000000	-5.013584000000	-0.072356000000
H	-1.823565000000	-4.228669000000	0.081223000000
H	-0.939098000000	-4.286310000000	1.680136000000
C	0.269727000000	-4.009569000000	-1.275207000000
H	-0.632514000000	-4.039873000000	-1.902509000000
H	1.192607000000	-3.931777000000	-1.867679000000
C	1.330667000000	-4.071189000000	0.630170000000
H	1.389116000000	-4.152223000000	1.725094000000
H	2.322117000000	-3.985679000000	0.162684000000
C	0.393679000000	-6.508740000000	-0.169889000000
C	-0.764185000000	-7.298002000000	-0.078348000000
C	-0.679783000000	-8.675507000000	-0.197786000000
C	0.551252000000	-9.327806000000	-0.411510000000
C	1.703856000000	-8.536093000000	-0.498681000000

C	1.620722000000	-7.143901000000	-0.378332000000
H	2.533406000000	-6.547809000000	-0.447690000000
H	-1.745013000000	-6.849811000000	0.091489000000
H	2.676343000000	-9.001478000000	-0.659273000000
O	0.508459000000	-10.675793000000	-0.509377000000
C	1.712840000000	-11.374728000000	-0.724340000000
H	2.195520000000	-11.076318000000	-1.673032000000
H	1.451331000000	-12.440094000000	-0.776563000000
H	2.429977000000	-11.219273000000	0.102724000000
F	-1.788098000000	-9.421174000000	-0.107236000000

X-Ray Crystallographic Data (CCDC 2286412):

Experimental

The crystal structure of **36** was obtained by suspending **36** (ca. 10 mg) at 25 °C in $CDCl_3$ (ca. 0.2 mL). The saturated solution was filtered through a syringe filter into a 4 mL vial at 25°C. A few drops of *n*-octane (ca. 50 µL) were added, and the solution was left standing at 25°C over 24 h. The atoms are depicted with 50% probability ellipsoids. The crystallographic data are summarized in the following table.

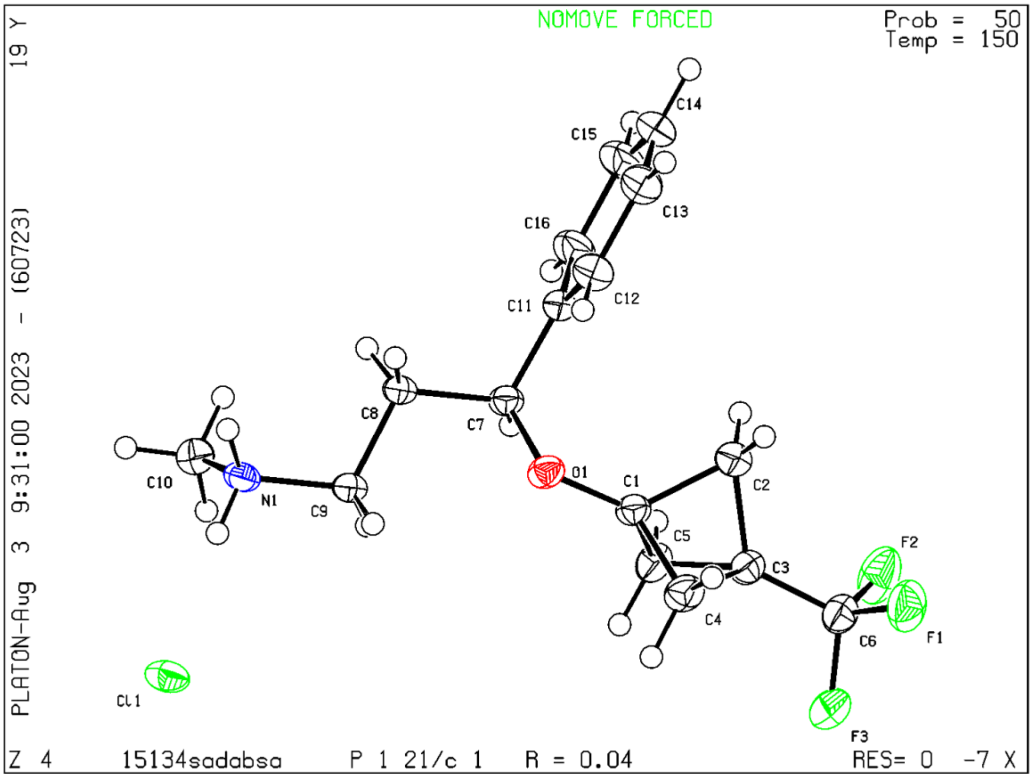

Figure S13. X-ray structure of **36**. Blue = nitrogen, yellow= sulfur, green = fluorine, red = oxygen.

Table S12. Crystal data and structure refinement.

Identification code	15134	
Empirical formula	$C_{16} H_{21} Cl F_3 N O$	
Color	colourless	
Formula weight	335.79 g · mol^{-1}	
Temperature	150(2) K	
Wavelength	0.71073 Å	
Crystal system	MONOCLINIC	
Space group	**$P2_1/c$, (no. 14)**	
Unit cell dimensions	a = 15.6607(5) Å	α = 90°.
	b = 10.1805(3) Å	ß = 94.386(2)°.
	c = 10.8334(4) Å	γ = 90°.
Volume	1722.15(10) Å^3	

Z	4	
Density (calculated)	1.295 Mg · m^{-3}	
Absorption coefficient	0.251 mm^{-1}	
F(000)	704 e	
Crystal size	0.273 x 0.207 x 0.110 mm^3	
θ range for data collection	2.388 to 33.210°.	
Index ranges	-24 ≤ h ≤ 24, -15≤ k ≤ 15, -16 ≤ l ≤ 16	
Reflections collected	554867	
Independent reflections	6602 [R_{int} = 0.0911]	
Reflections with I>2 σ (I)	5690	
Completeness to θ = 25.242°	100.0 %	
Absorption correction	Semi-empirical from equivalents	
Max. and min. transmission	0.98 and 0.94	
Refinement method	Full-matrix least-squares on F^2	
Data / restraints / parameters	6602 / 0 / 208	
Goodness-of-fit on F^2	1.042	
Final R indices [I>2 σ (I)]	R_1 = 0.0351	wR^2 = 0.0929
R indices (all data)	R_1 = 0.0420	wR^2 = 0.0995
Largest diff. peak and hole	0.5 and -0.2 e · $Å^{-3}$	

Table S13. Bond lengths [Å] and angles [°].

F(1)-C(6)	1.3376(11)	F(2)-C(6)	1.3380(12)
F(3)-C(6)	1.3393(12)	O(1)-C(1)	1.3851(10)
O(1)-C(7)	1.4357(9)	N(1)-C(9)	1.4982(11)
N(1)-C(10)	1.4851(12)	N(1)-H(1A)	0.931(14)
N(1)-H(1B)	0.908(15)	C(1)-C(2)	1.5576(12)
C(1)-C(3)	1.8332(11)	C(1)-C(4)	1.5367(11)
C(1)-C(5)	1.5598(12)	C(2)-C(3)	1.5463(12)
C(3)-C(4)	1.5493(12)	C(3)-C(5)	1.5520(12)
C(3)-C(6)	1.4826(12)	C(7)-C(8)	1.5253(11)
C(7)-C(11)	1.5142(11)	C(8)-C(9)	1.5210(11)
C(11)-C(12)	1.3898(13)	C(11)-C(16)	1.3886(12)
C(12)-C(13)	1.3904(14)	C(13)-C(14)	1.3841(18)
C(14)-C(15)	1.3775(19)	C(15)-C(16)	1.3942(14)
C(1)-O(1)-C(7)	113.77(6)	C(9)-N(1)-H(1A)	108.4(8)
C(9)-N(1)-H(1B)	107.8(9)	C(10)-N(1)-C(9)	113.95(7)
C(10)-N(1)-H(1A)	111.9(8)	C(10)-N(1)-H(1B)	107.8(9)
H(1A)-N(1)-H(1B)	106.6(12)	O(1)-C(1)-C(2)	129.27(7)
O(1)-C(1)-C(3)	176.00(7)	O(1)-C(1)-C(4)	122.26(7)
O(1)-C(1)-C(5)	127.25(7)	C(2)-C(1)-C(3)	53.52(5)
C(2)-C(1)-C(5)	88.10(6)	C(4)-C(1)-C(2)	88.56(6)
C(4)-C(1)-C(3)	53.87(5)	C(4)-C(1)-C(5)	88.91(6)
C(5)-C(1)-C(3)	53.71(5)	C(3)-C(2)-C(1)	72.40(6)
C(2)-C(3)-C(1)	54.09(5)	C(2)-C(3)-C(4)	88.52(7)
C(2)-C(3)-C(5)	88.79(6)	C(4)-C(3)-C(1)	53.24(5)
C(4)-C(3)-C(5)	88.75(6)	C(5)-C(3)-C(1)	54.11(5)
C(6)-C(3)-C(1)	178.80(8)	C(6)-C(3)-C(2)	125.69(8)
C(6)-C(3)-C(4)	125.84(7)	C(6)-C(3)-C(5)	127.03(8)
C(1)-C(4)-C(3)	72.89(6)	C(3)-C(5)-C(1)	72.19(6)
F(1)-C(6)-F(2)	106.47(8)	F(1)-C(6)-F(3)	106.20(8)
F(1)-C(6)-C(3)	112.29(8)	F(2)-C(6)-F(3)	106.89(9)
F(2)-C(6)-C(3)	112.42(8)	F(3)-C(6)-C(3)	112.14(8)
O(1)-C(7)-C(8)	106.49(6)	O(1)-C(7)-C(11)	111.64(7)

C(11)-C(7)-C(8)	111.61(6)	C(9)-C(8)-C(7)	111.24(6)
N(1)-C(9)-C(8)	111.52(6)	C(12)-C(11)-C(7)	120.48(8)
C(16)-C(11)-C(7)	120.33(8)	C(16)-C(11)-C(12)	119.18(8)
C(11)-C(12)-C(13)	120.60(10)	C(14)-C(13)-C(12)	119.74(10)
C(15)-C(14)-C(13)	120.12(9)	C(14)-C(15)-C(16)	120.26(10)
C(11)-C(16)-C(15)	120.09(10)		

SPECTROSCOPIC DATA

^{1}H NMR of bicyclo[1.1.1]pentylether 1

CDCl$_3$, 298 K

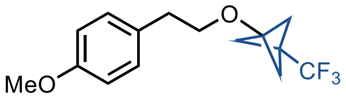

1

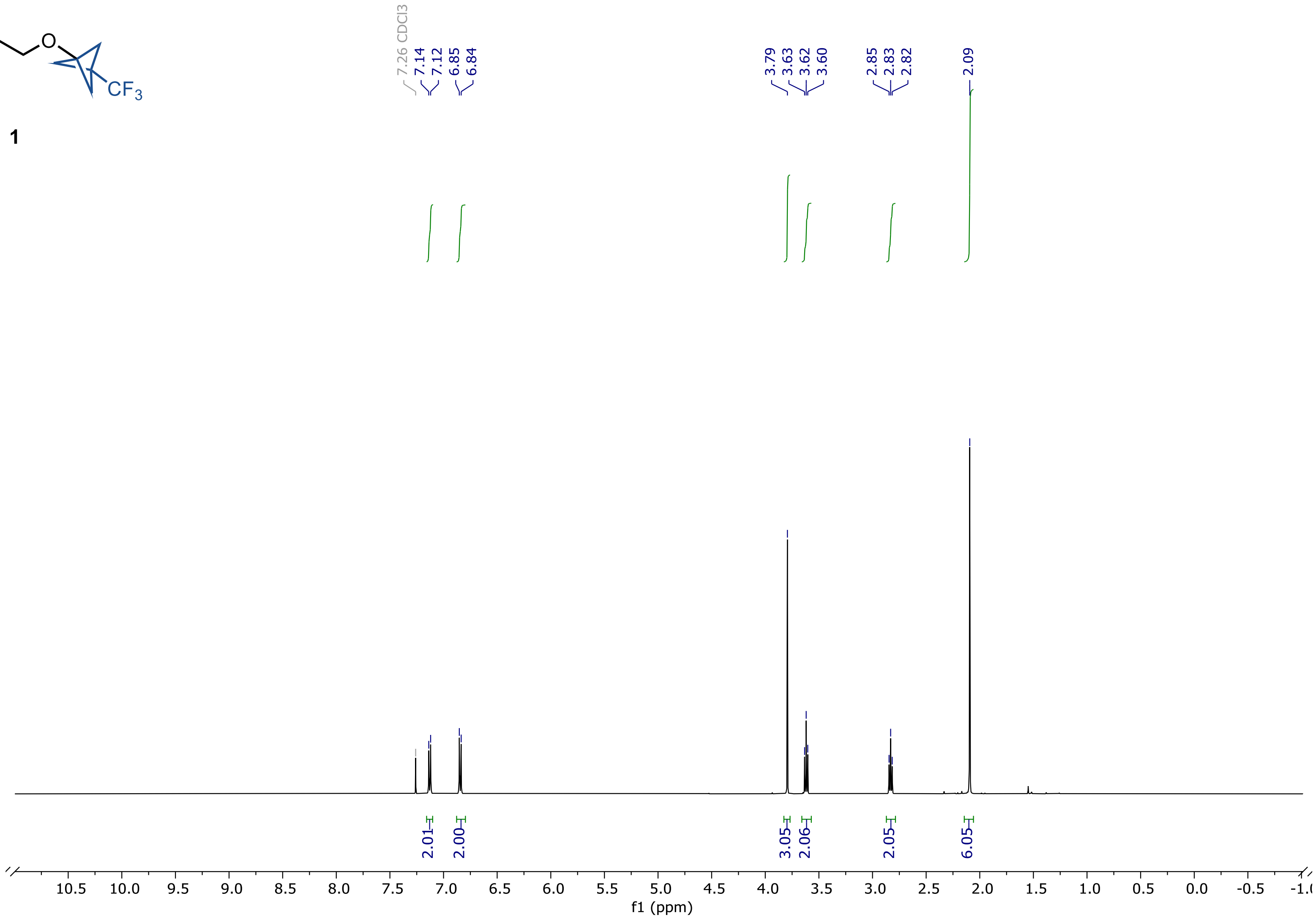

^{13}C NMR of bicyclo[1.1.1]pentylether 1

$CDCl_3$, 298 K

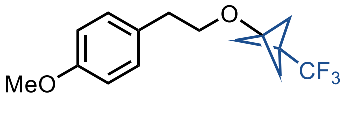

1

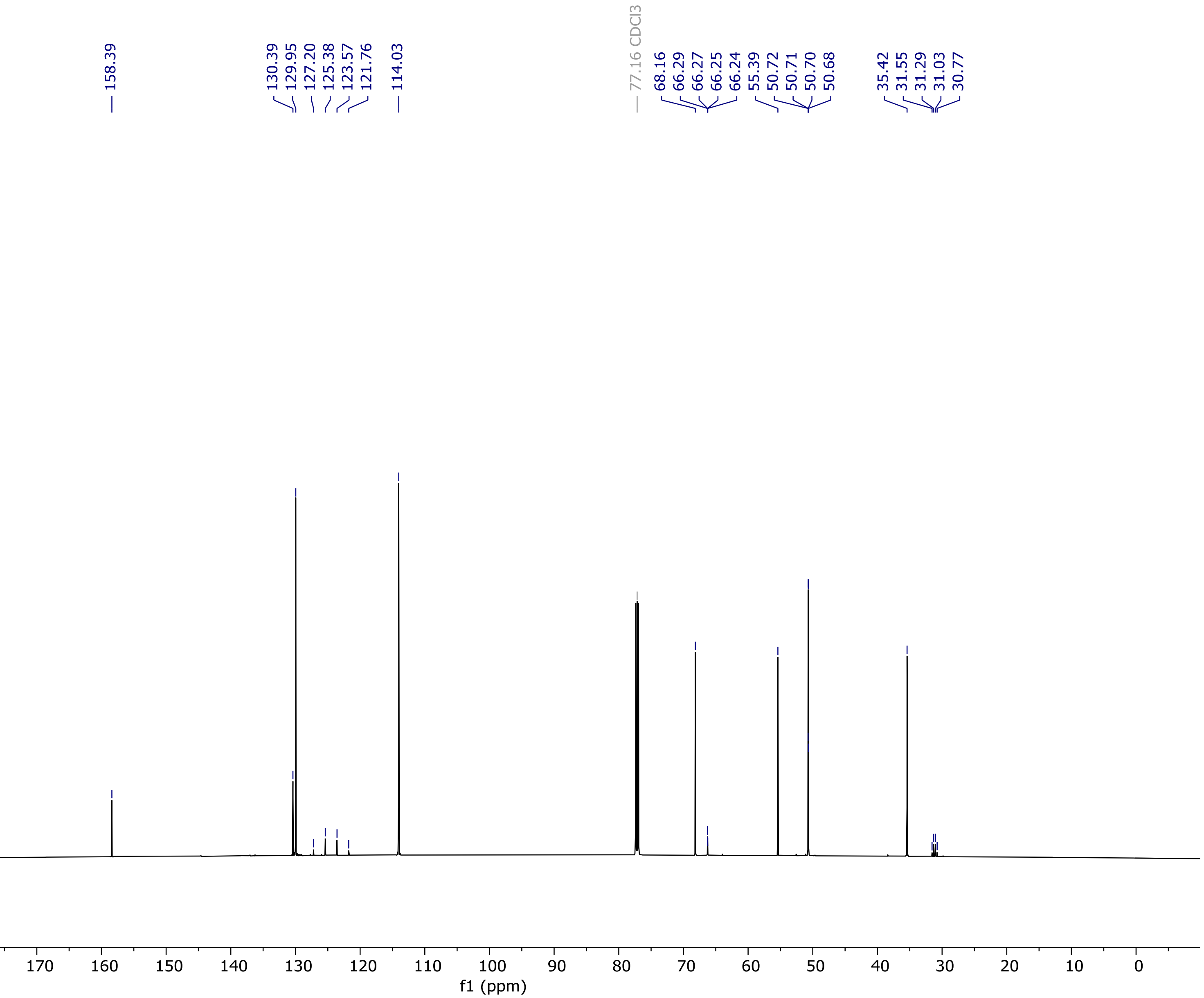

^{19}F NMR of bicyclo[1.1.1]pentylether 1

$CDCl_3$, 298 K

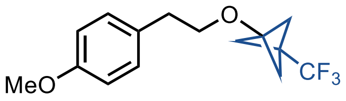

1

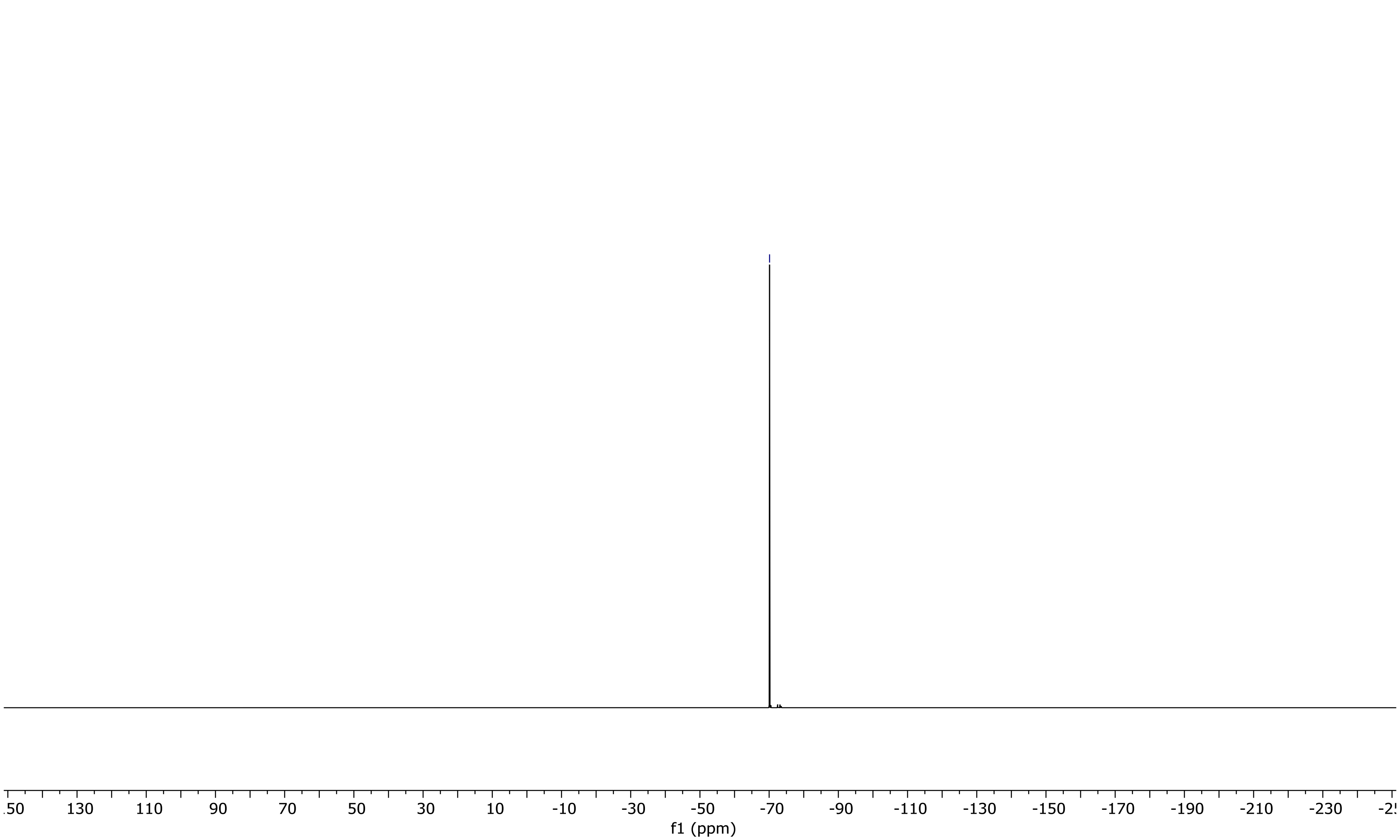

^{1}H NMR of bicyclo[1.1.1]pentylether 2

$CDCl_3$, 298 K

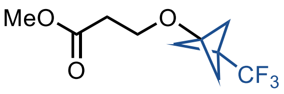

2

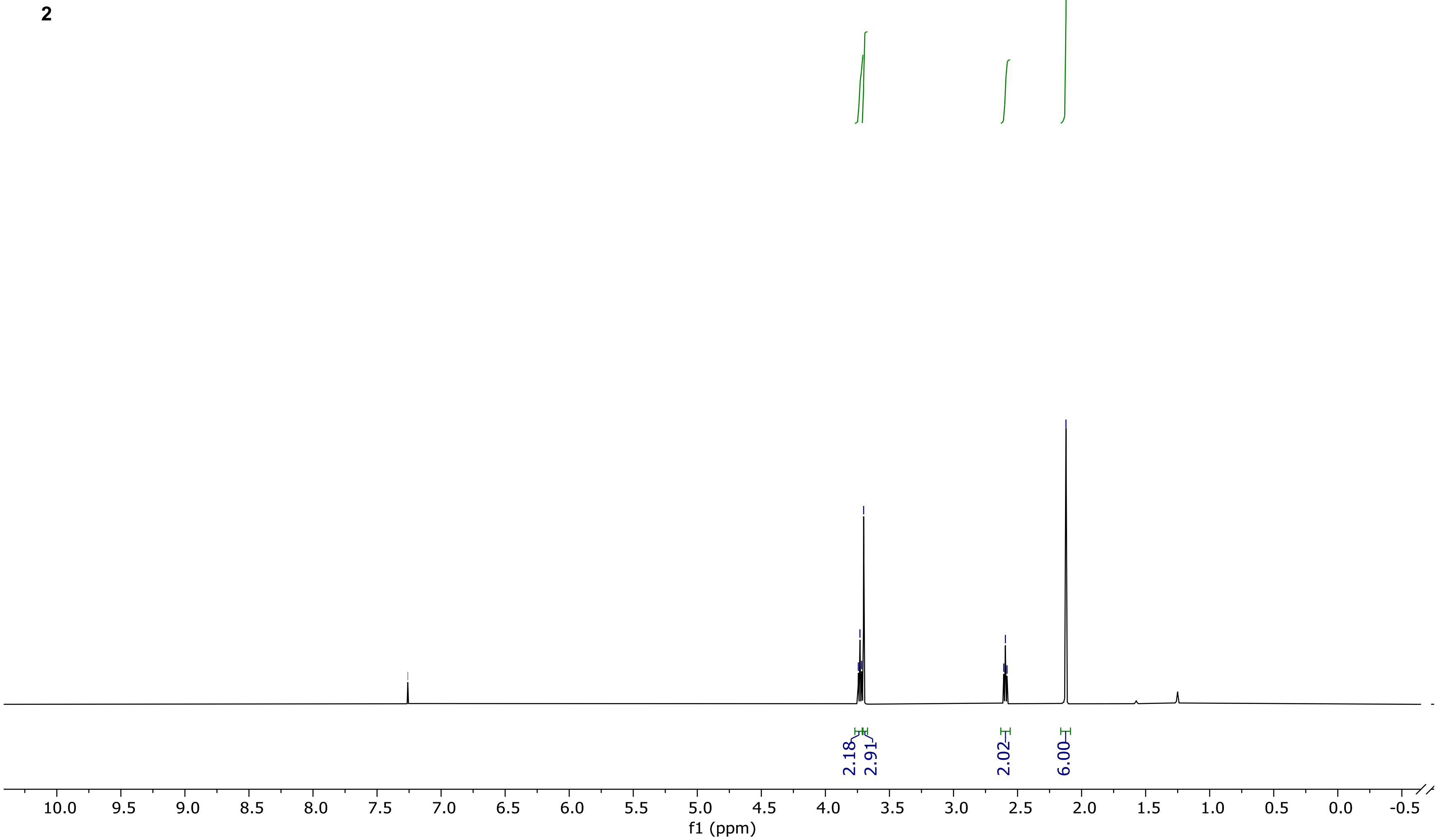

^{13}C NMR of bicyclo[1.1.1]pentylether 2

CDCl$_3$, 298 K

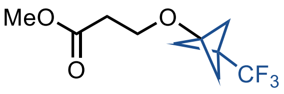

2

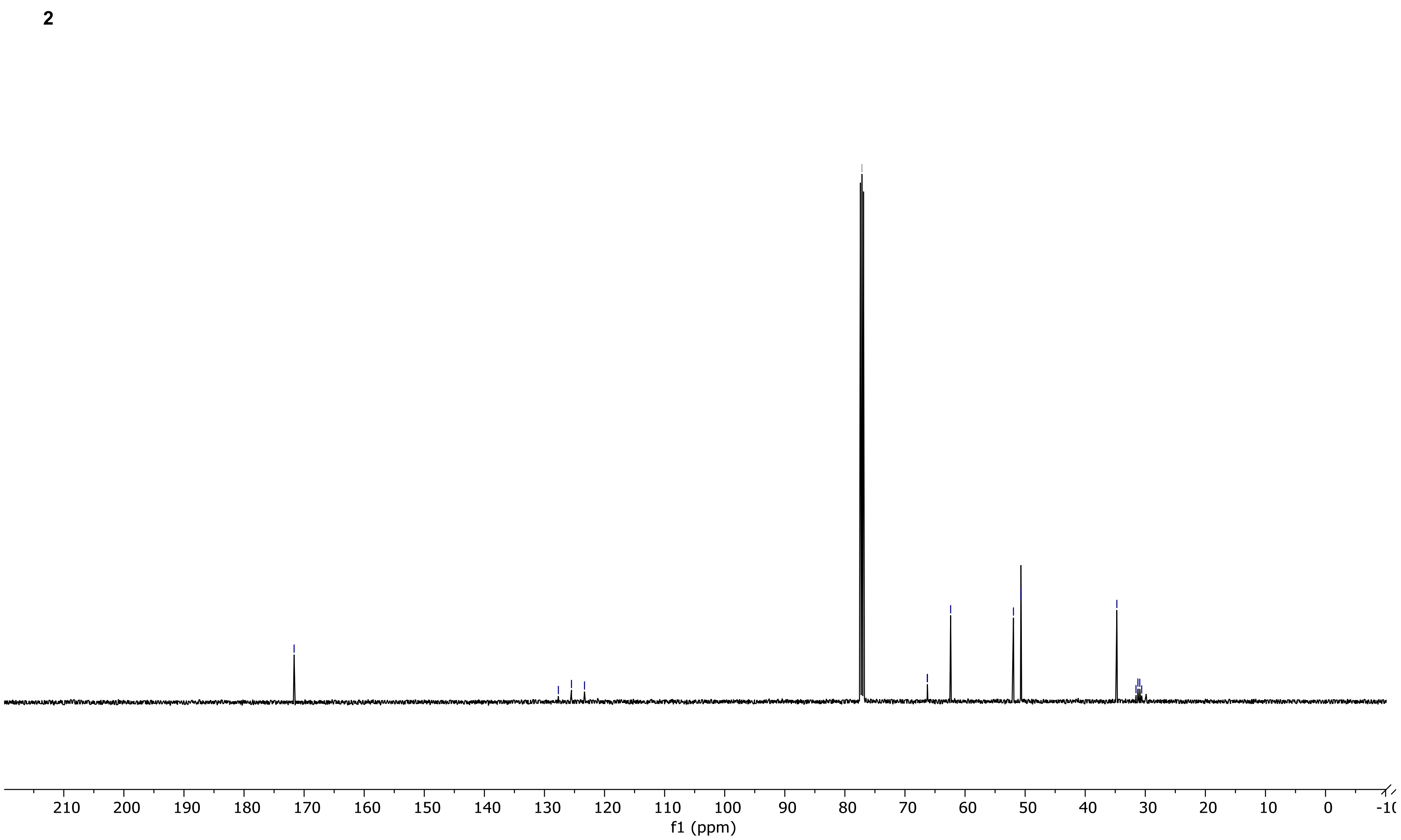

19F NMR of bicyclo[1.1.1]pentylether 2

$CDCl_3$, 298 K

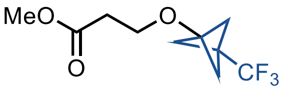

2

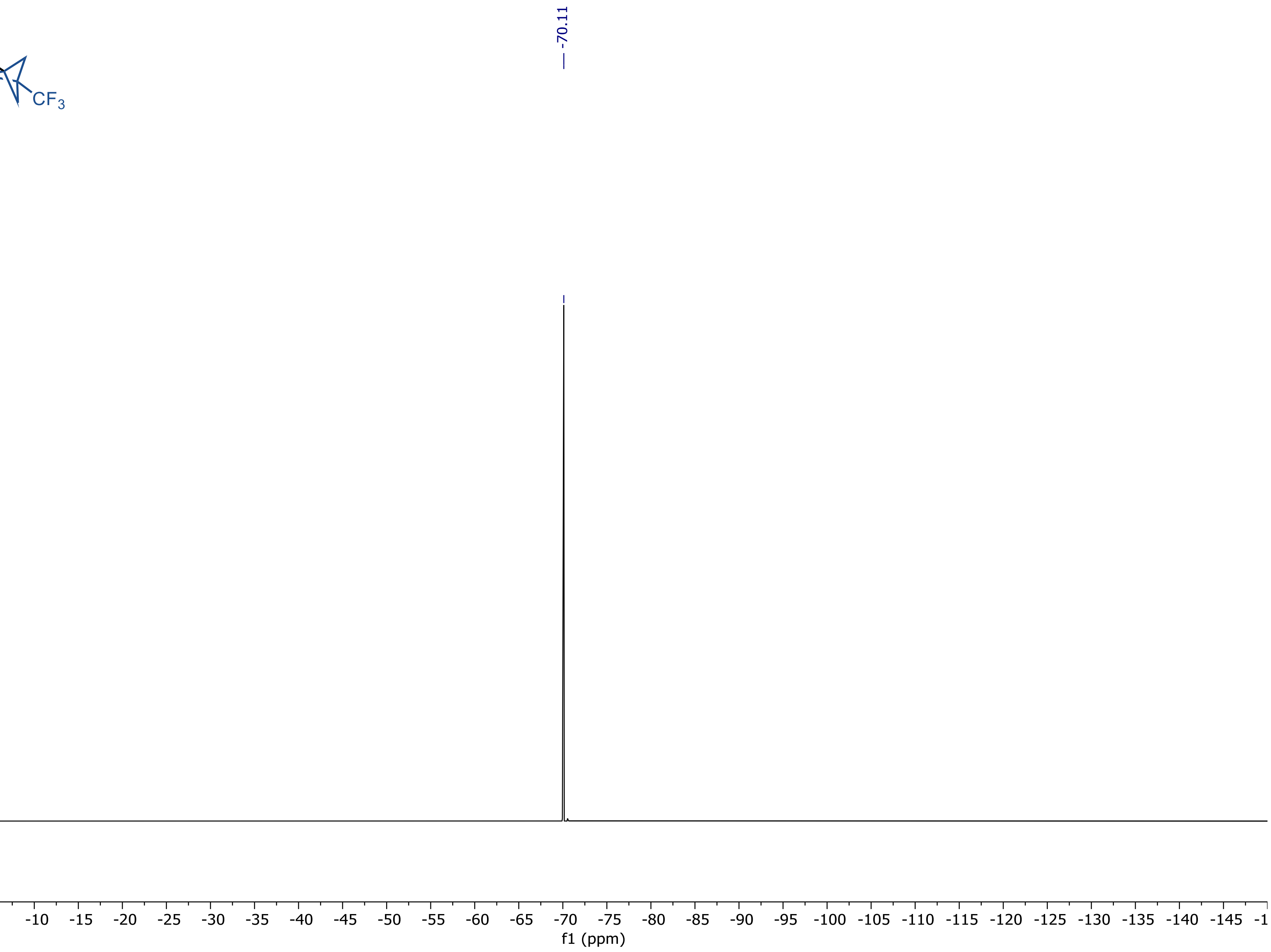

^{1}H NMR of bicyclo[1.1.1]pentylether 3

$CDCl_3$, 298 K

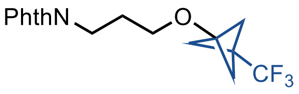

3

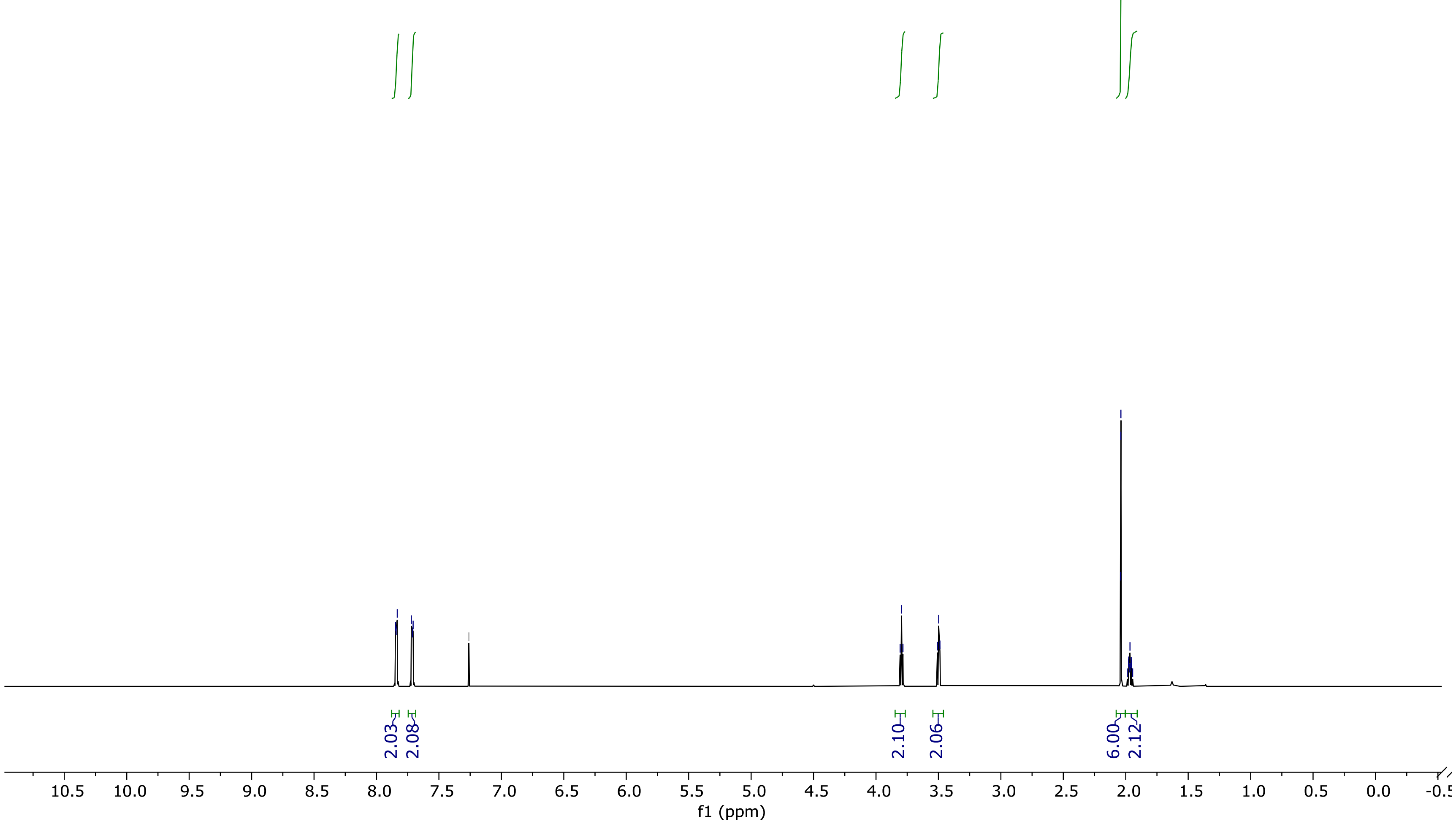

^{13}C NMR of bicyclo[1.1.1]pentylether 3

$CDCl_3$, 298 K

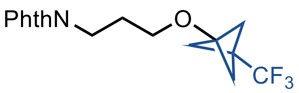

3

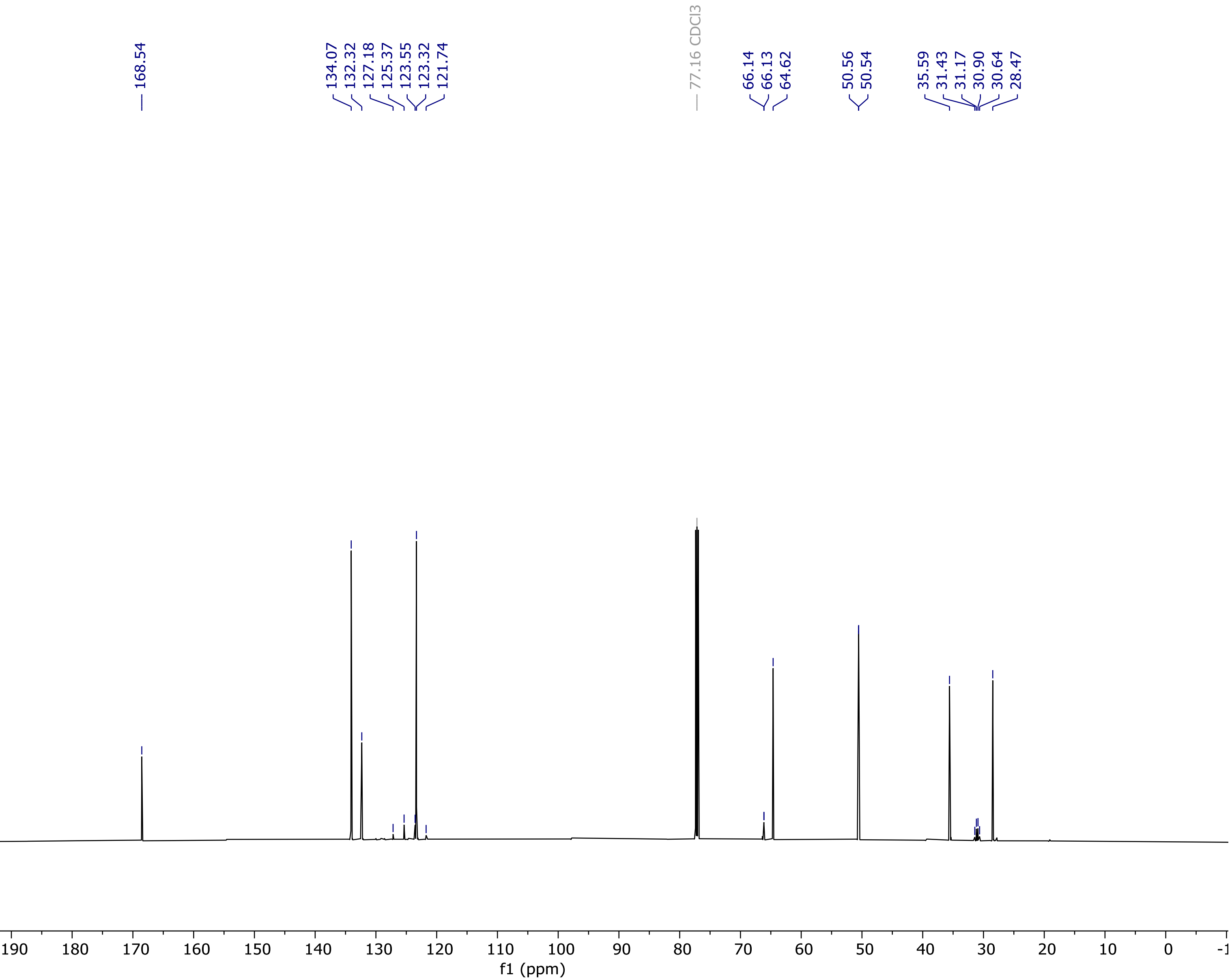

^{19}F NMR of bicyclo[1.1.1]pentylether 3

$CDCl_3$, 298 K

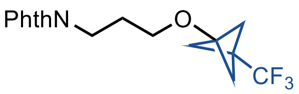

3

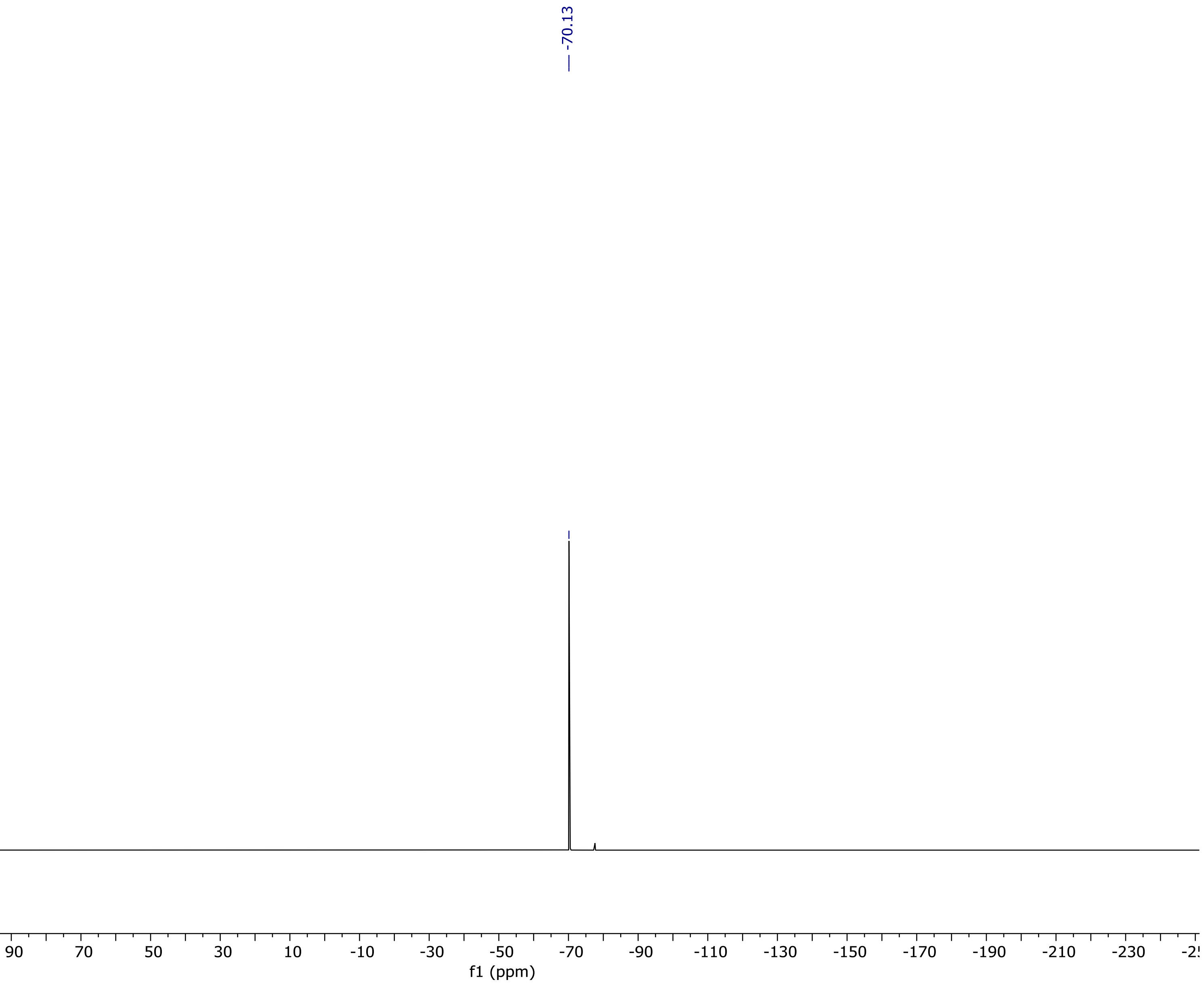

^{1}H NMR of bicyclo[1.1.1]pentylether 4

CDCl$_3$, 298 K

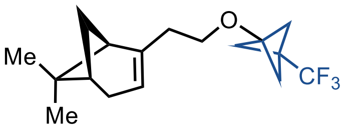

4

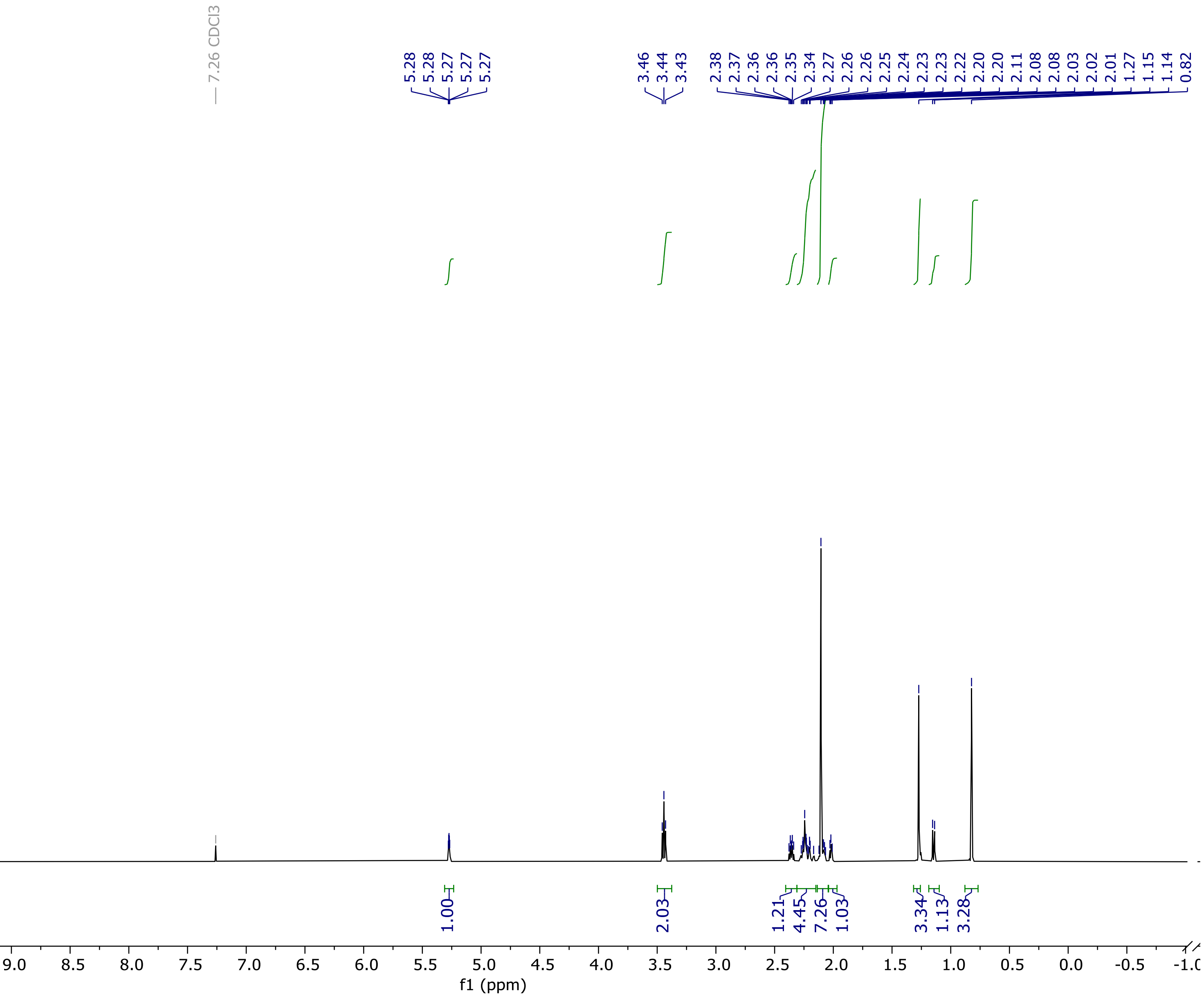

^{13}C NMR of bicyclo[1.1.1]pentylether 4

$CDCl_3$, 298 K

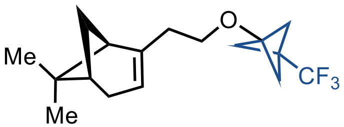

4

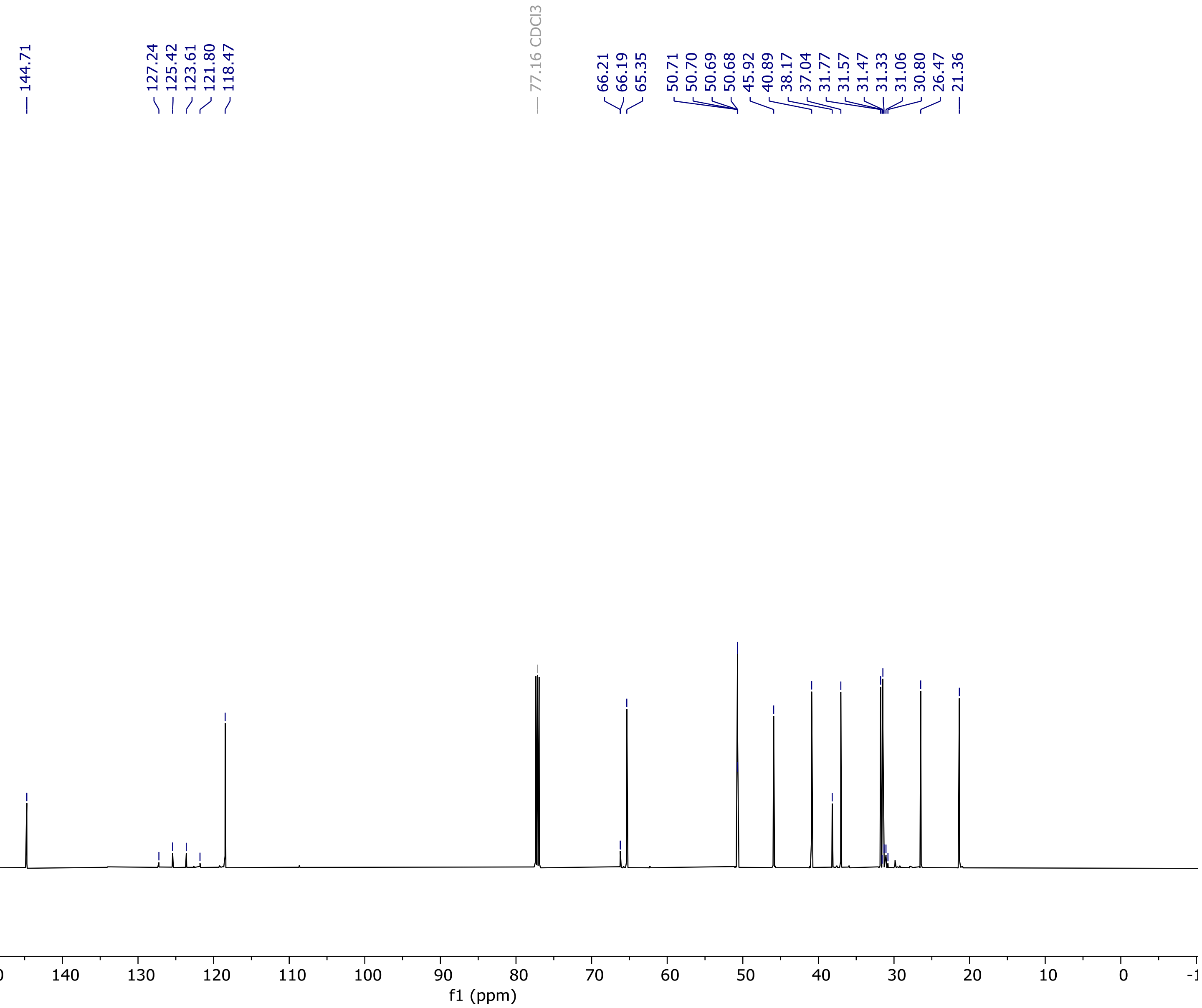

^{19}F NMR of bicyclo[1.1.1]pentylether 4

$CDCl_3$, 298 K

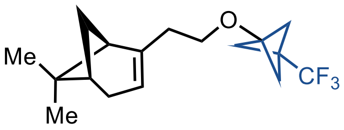

4

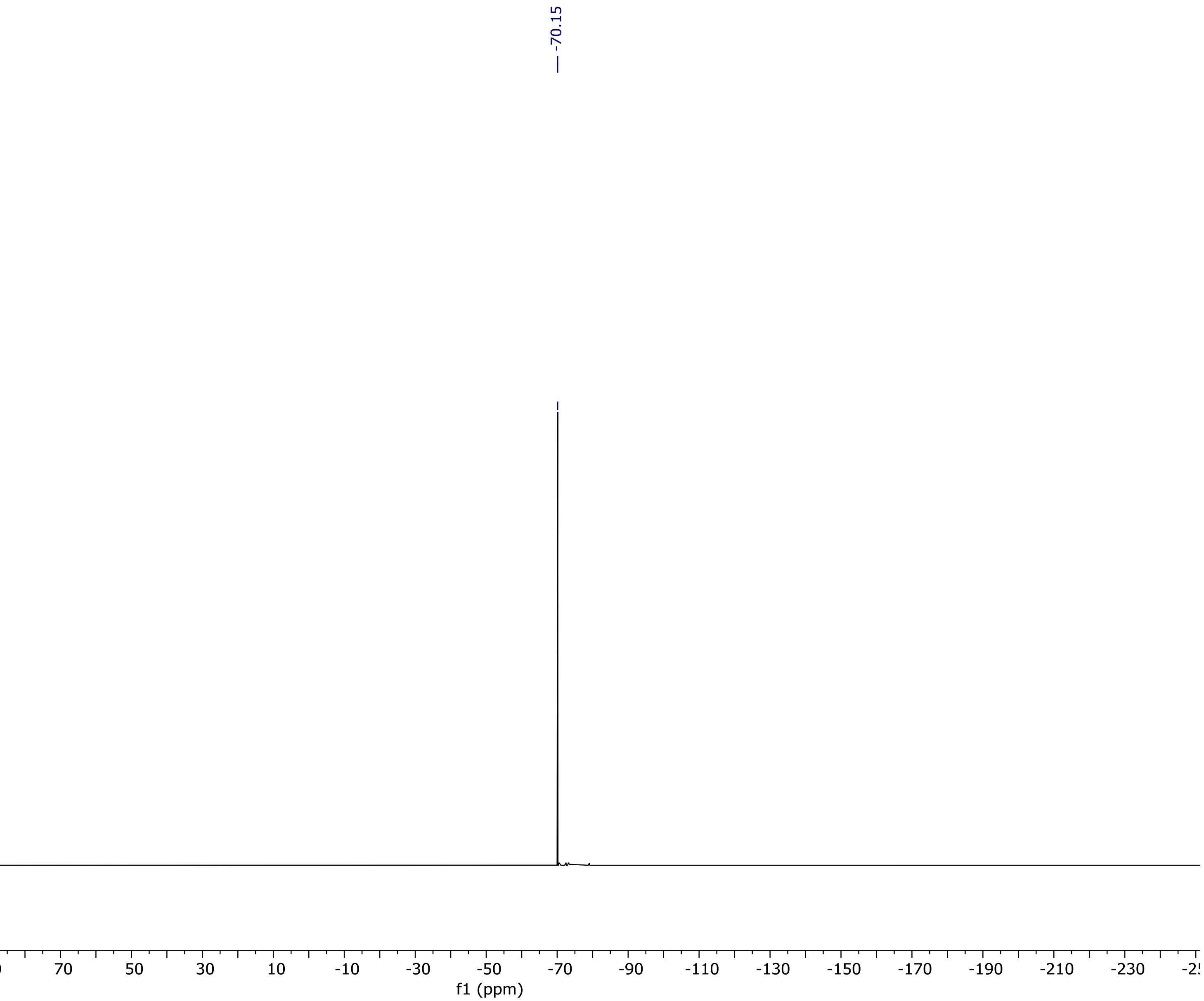

^{1}H NMR of bicyclo[1.1.1]pentylether 5

$CDCl_3$, 298 K

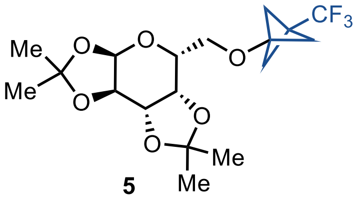

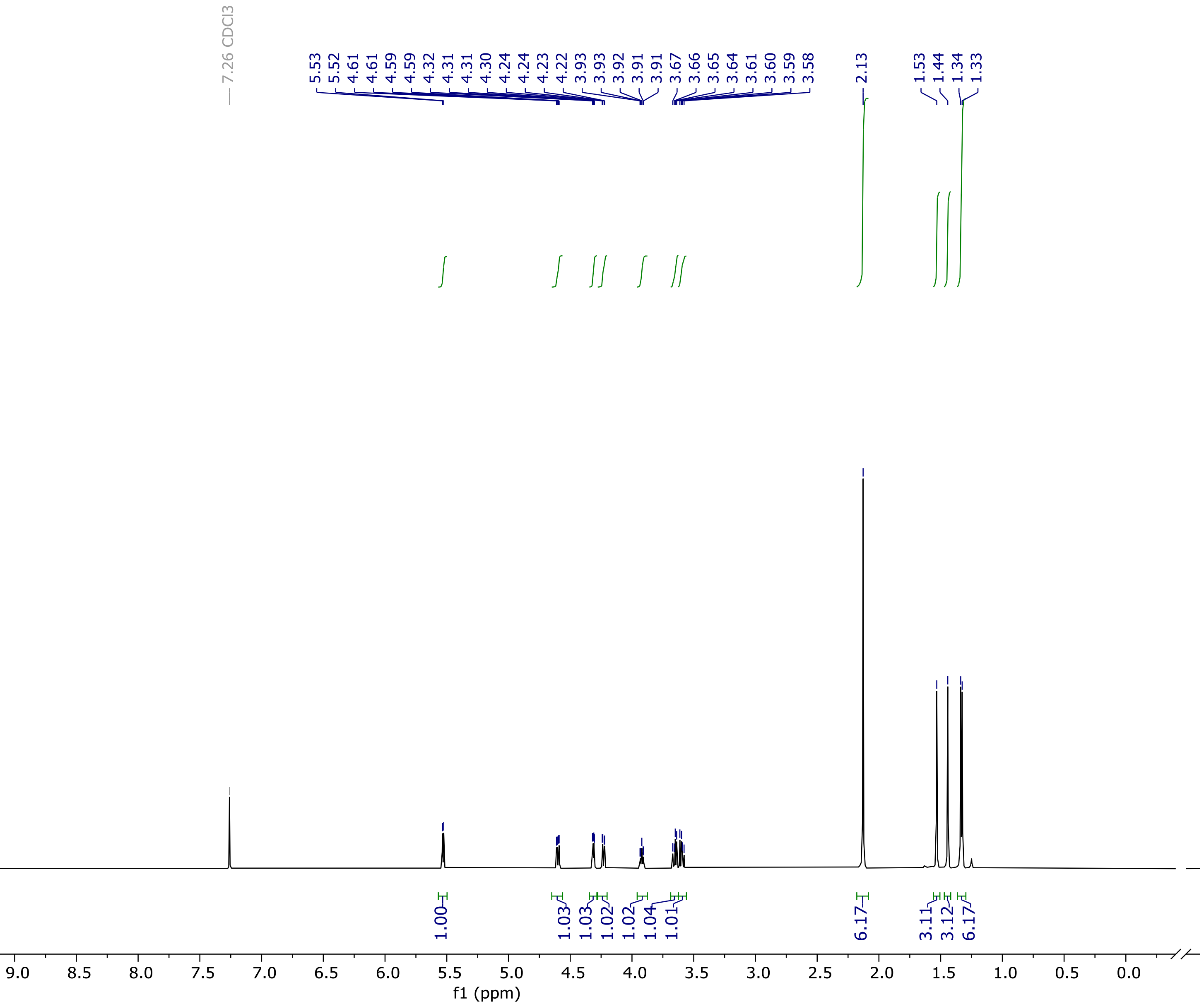

^{13}C NMR of bicyclo[1.1.1]pentylether 5

CDCl$_3$, 298 K

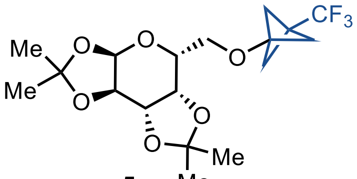

5

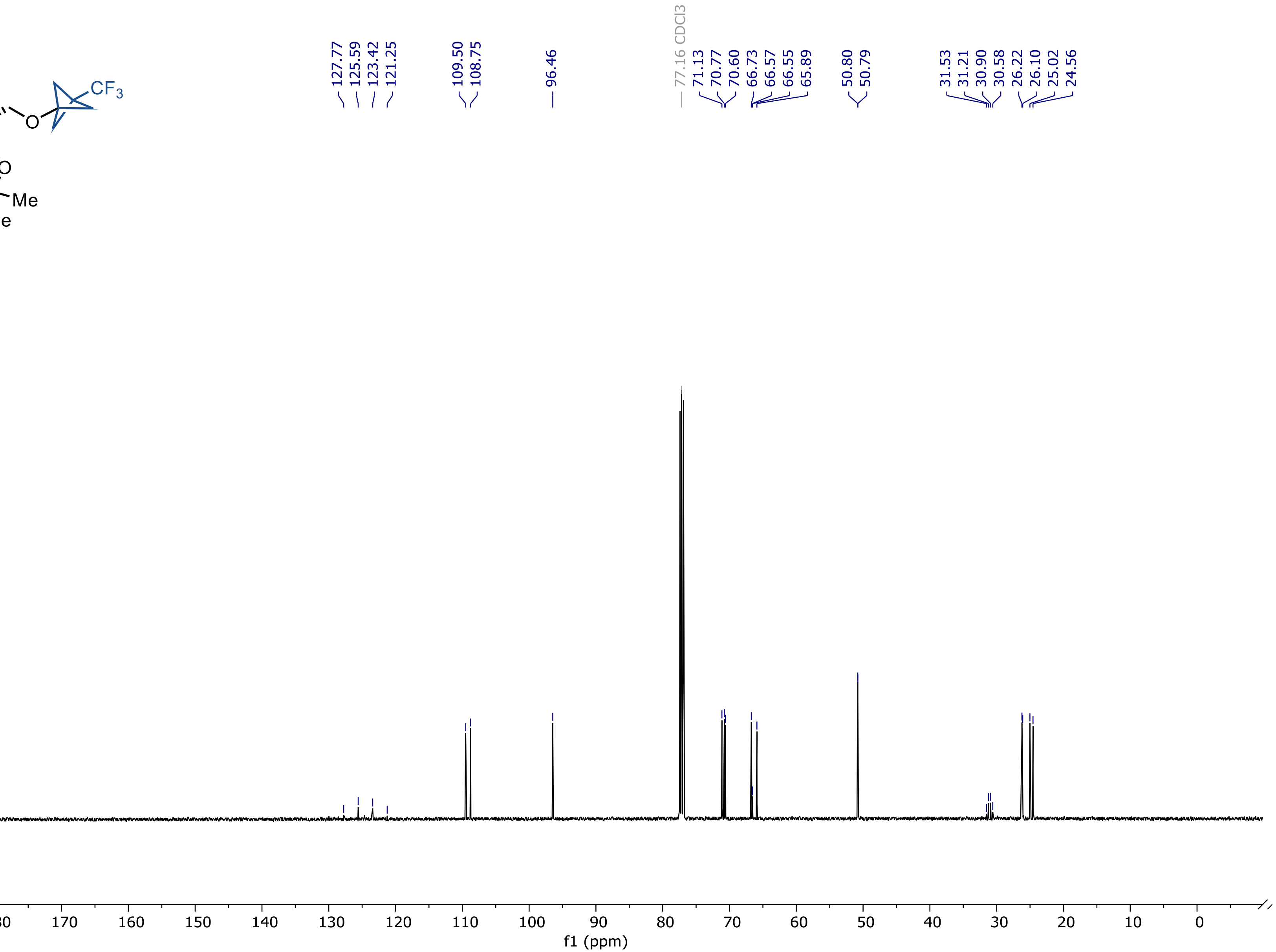

^{19}F NMR of bicyclo[1.1.1]pentylether 5

$CDCl_3$, 298 K

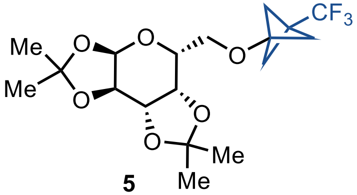

5

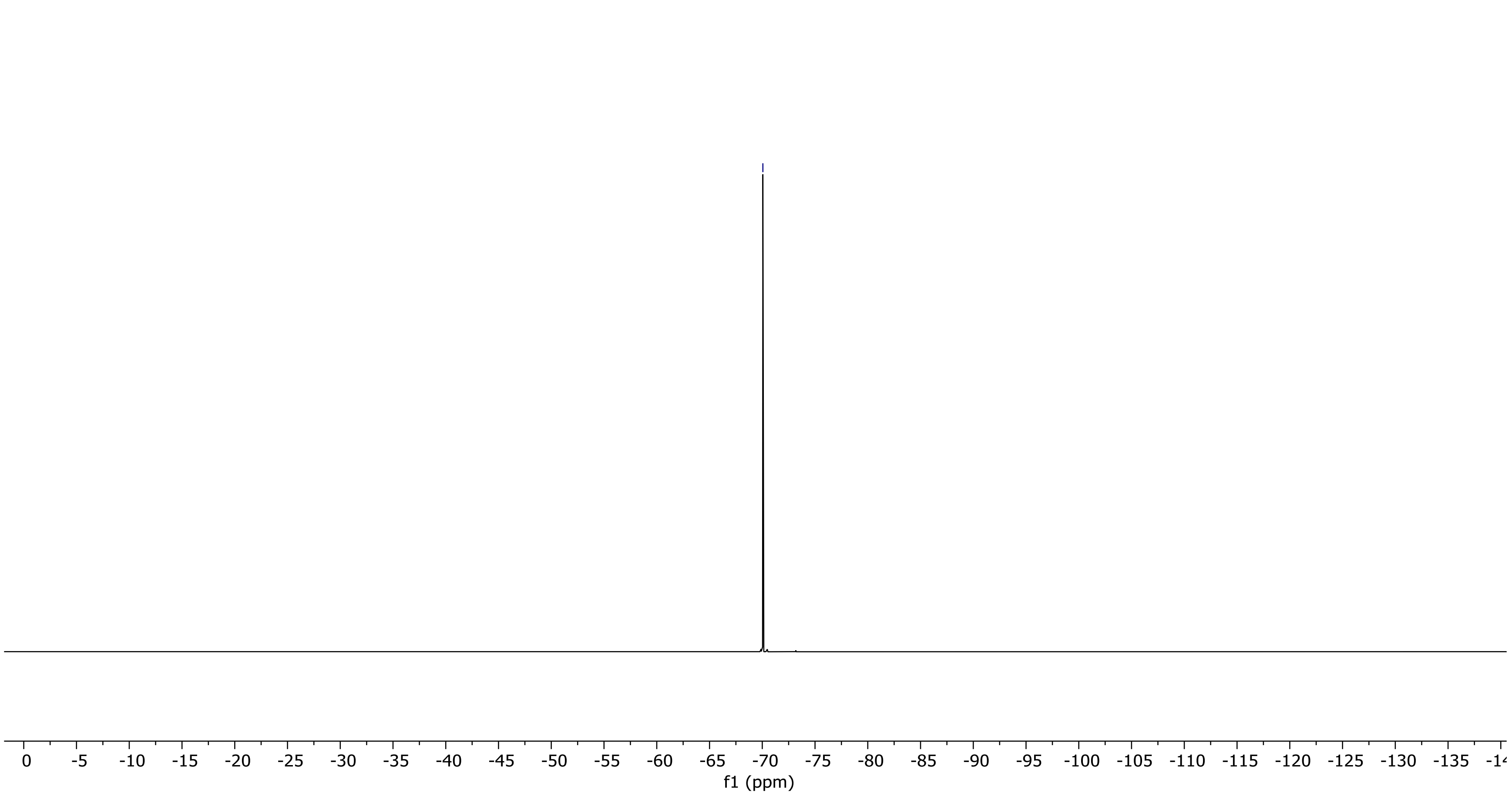

^{1}H NMR of bicyclo[1.1.1]pentylether 6

CDCl3, 298 K

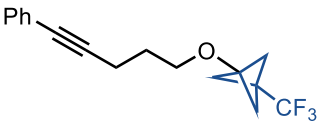

6

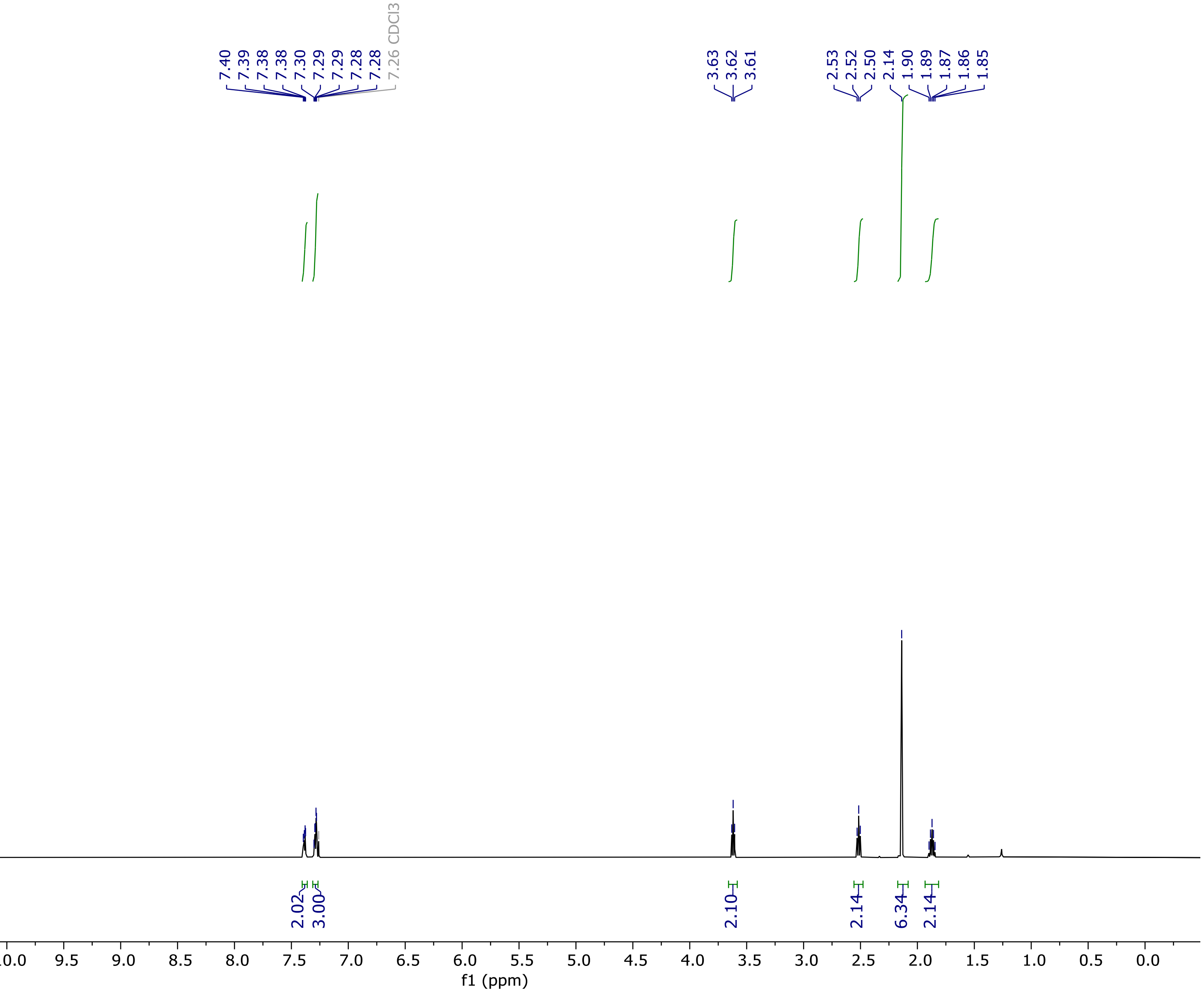

^{13}C NMR of bicyclo[1.1.1]pentylether 6

CDCl$_3$, 298 K

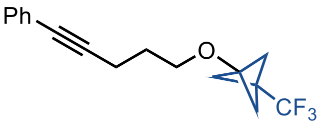

6

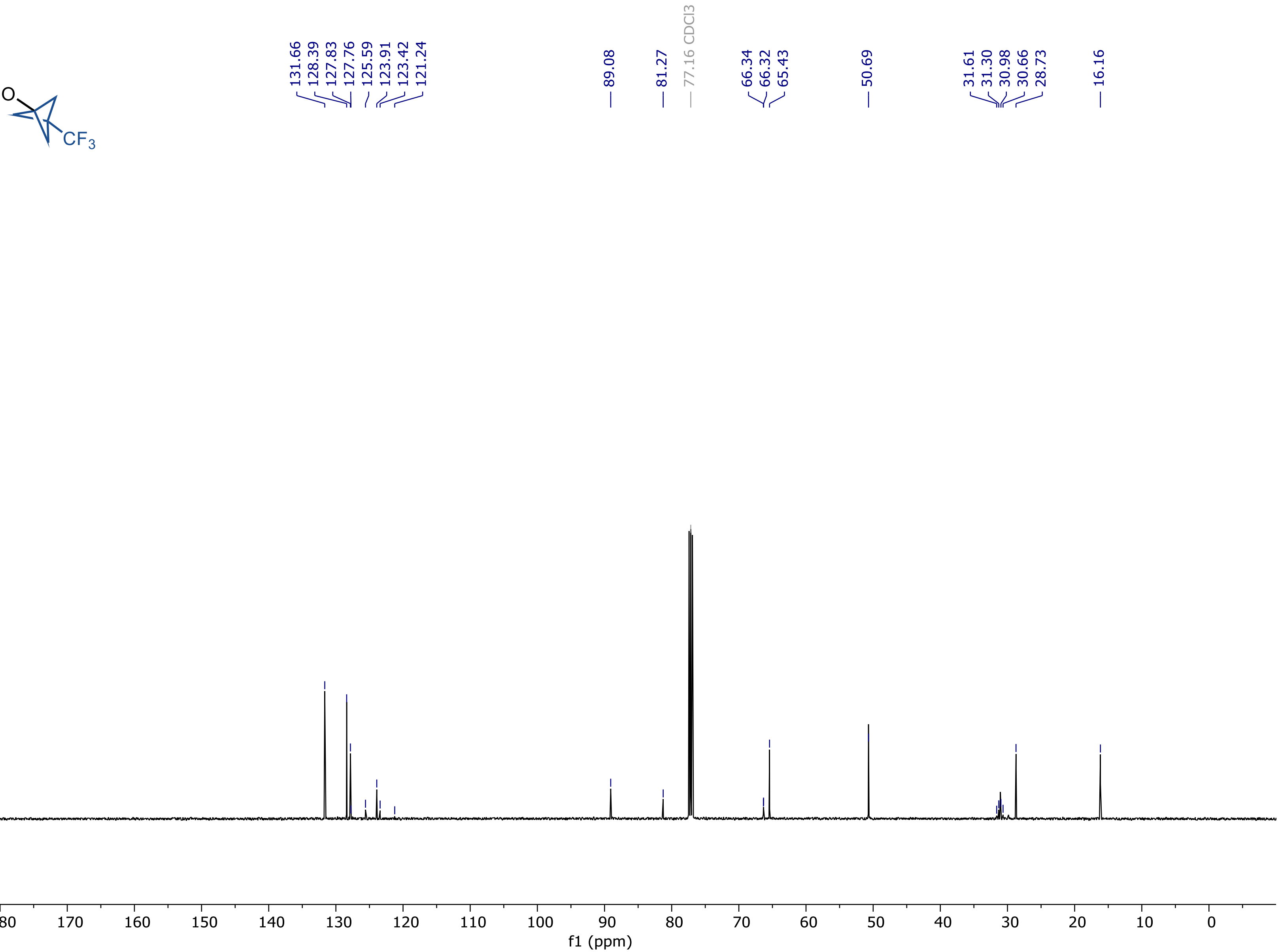

^{19}F NMR of bicyclo[1.1.1]pentylether 6

$CDCl_3$, 298 K

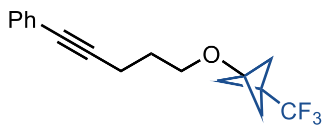

6

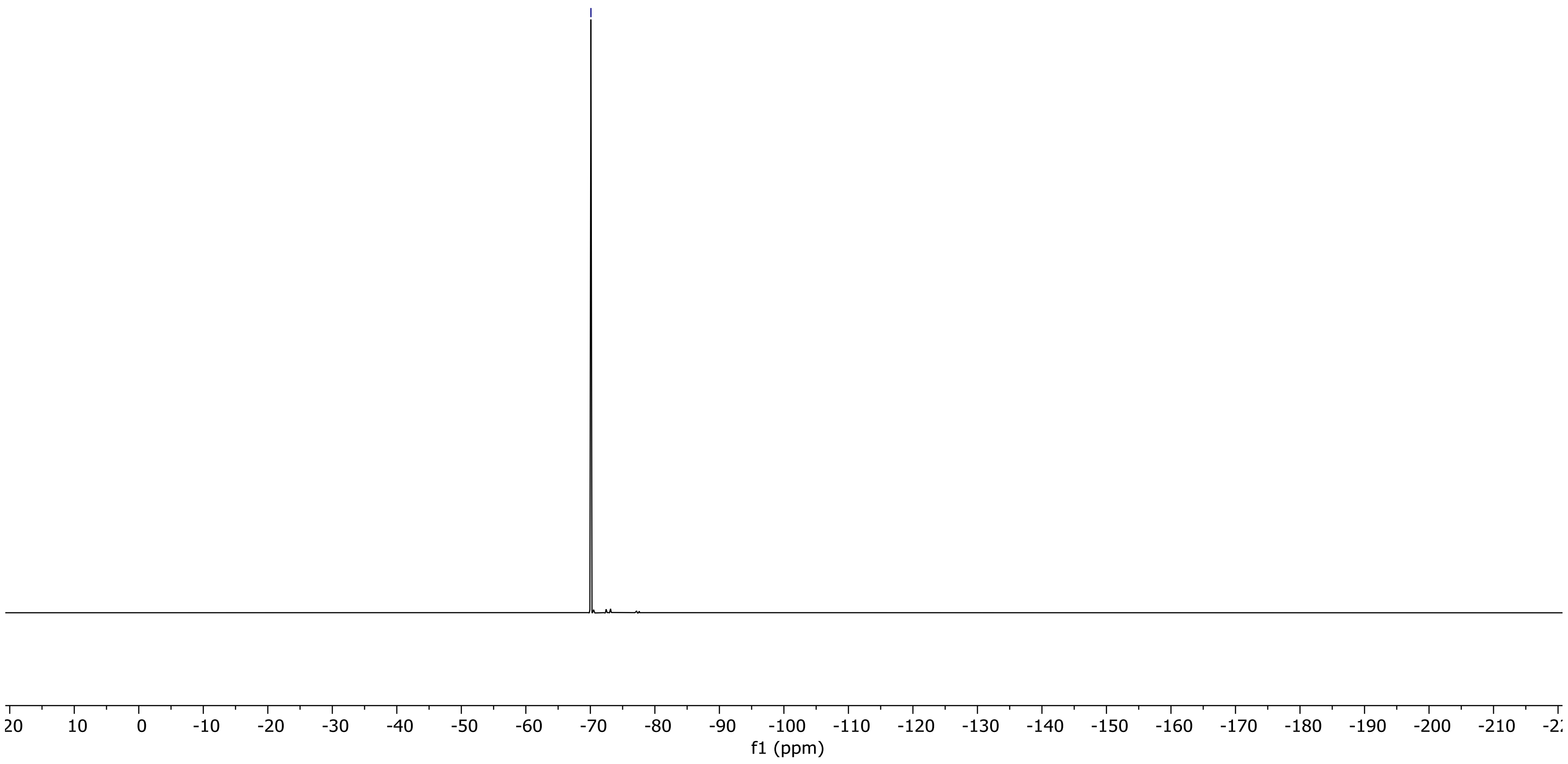

^{1}H NMR of bicyclo[1.1.1]pentylether 7

$CDCl_3$, 298 K

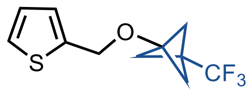

7

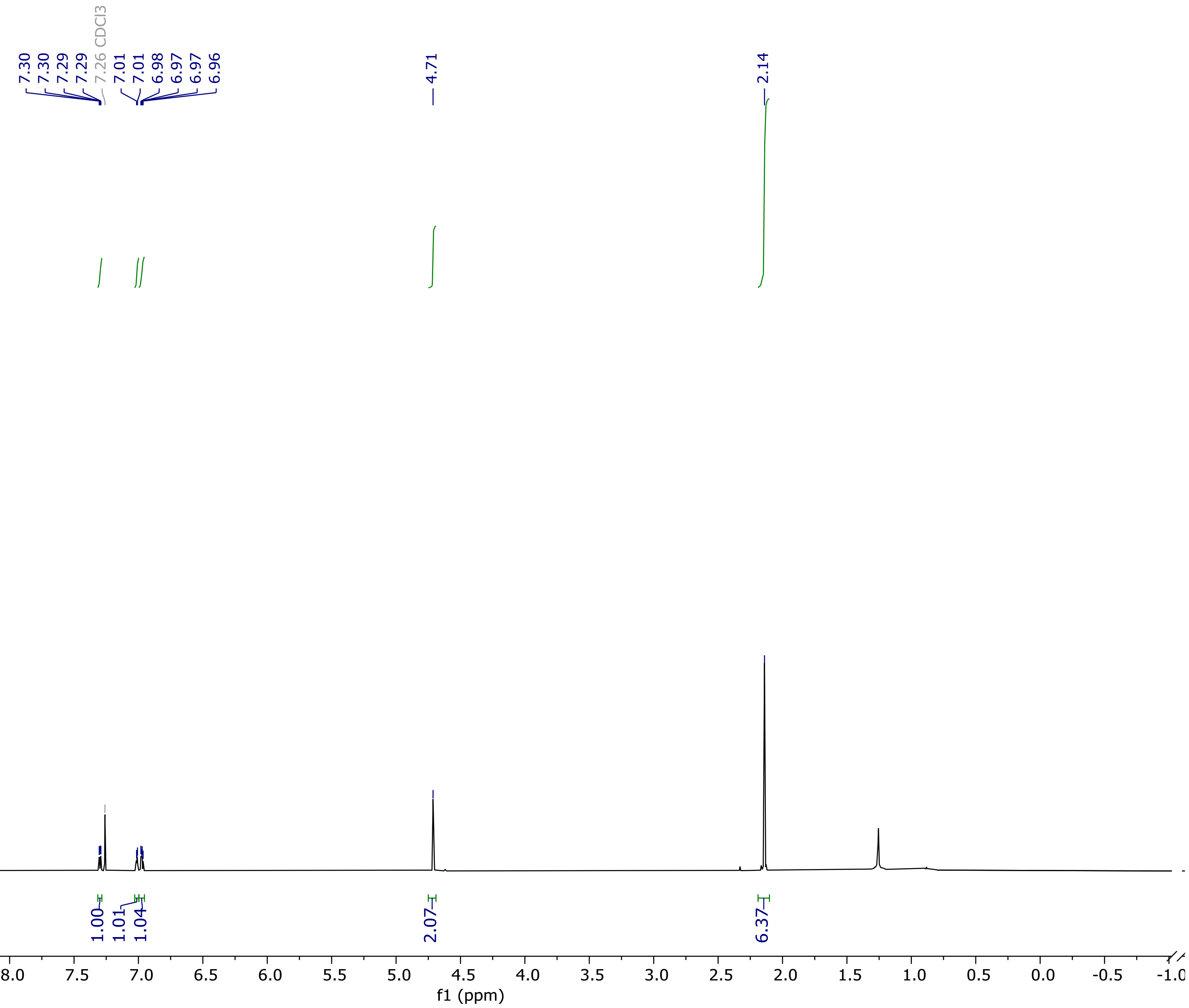

^{13}C NMR of bicyclo[1.1.1]pentylether 7

$CDCl_3$, 298 K

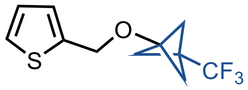

7

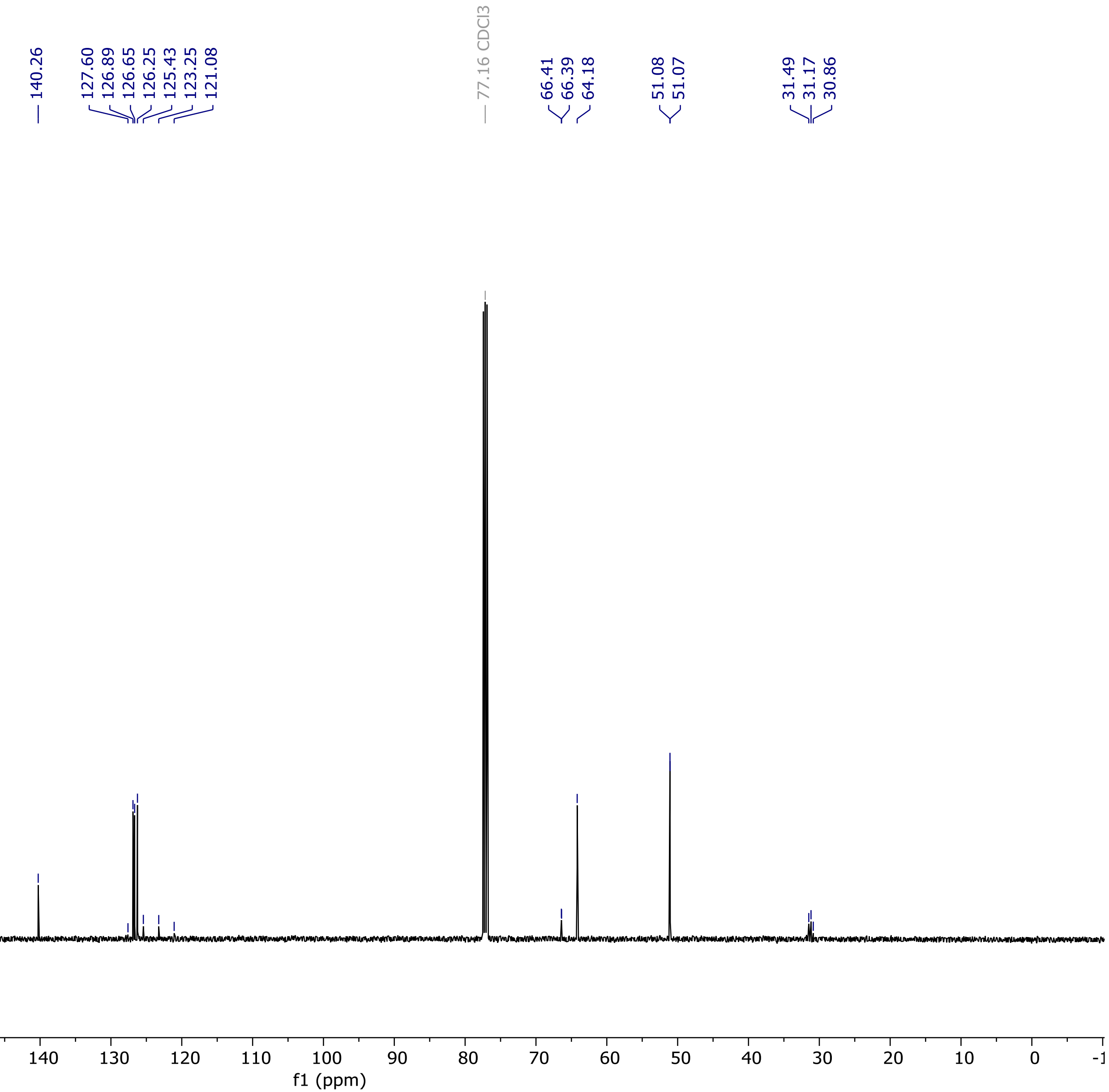

^{19}F NMR of bicyclo[1.1.1]pentylether 7

CDCl$_3$, 298 K

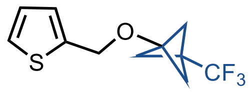

7

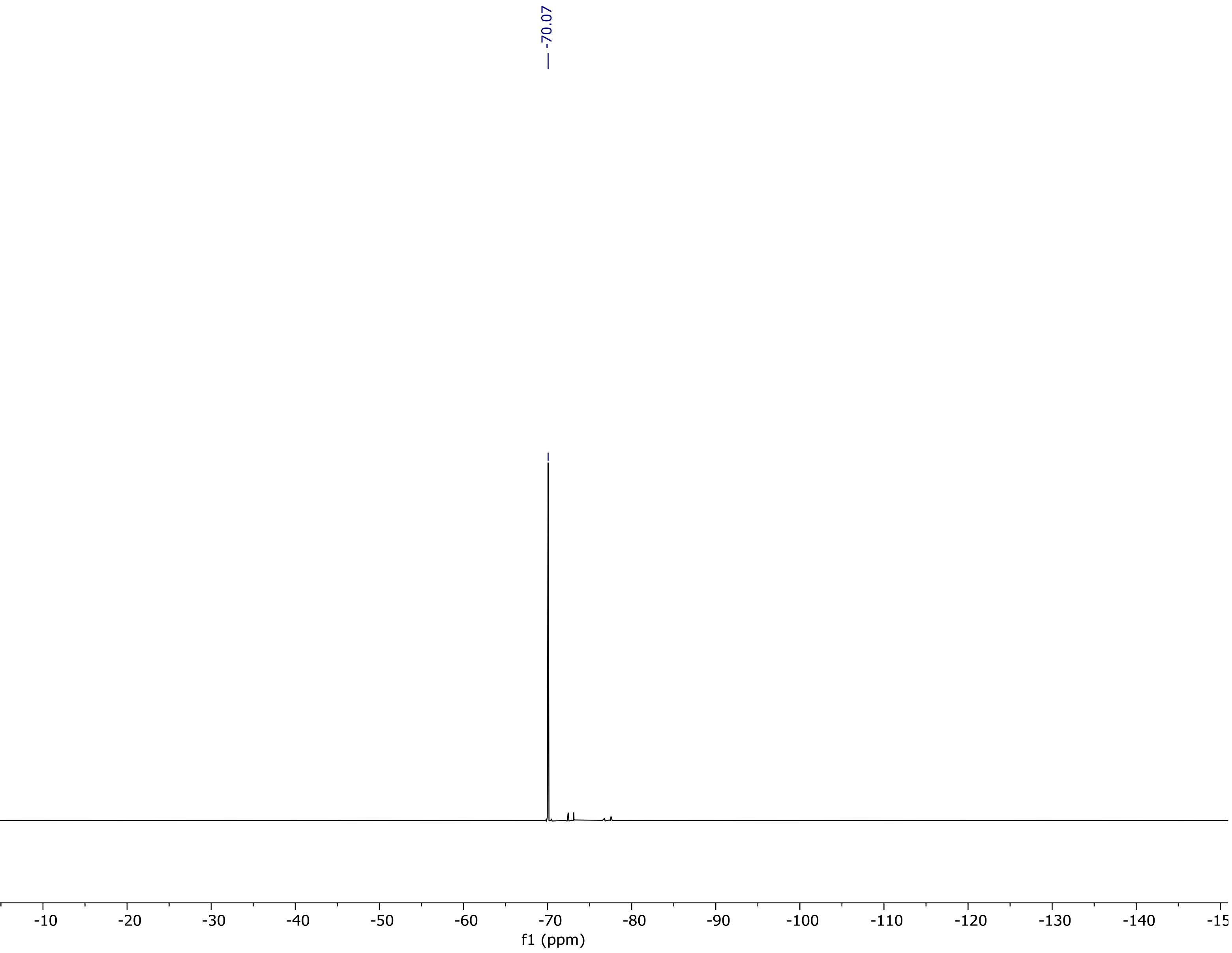

^{1}H NMR of bicyclo[1.1.1]pentylether 8

$CDCl_3$, 298 K

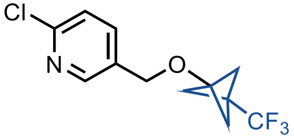

8

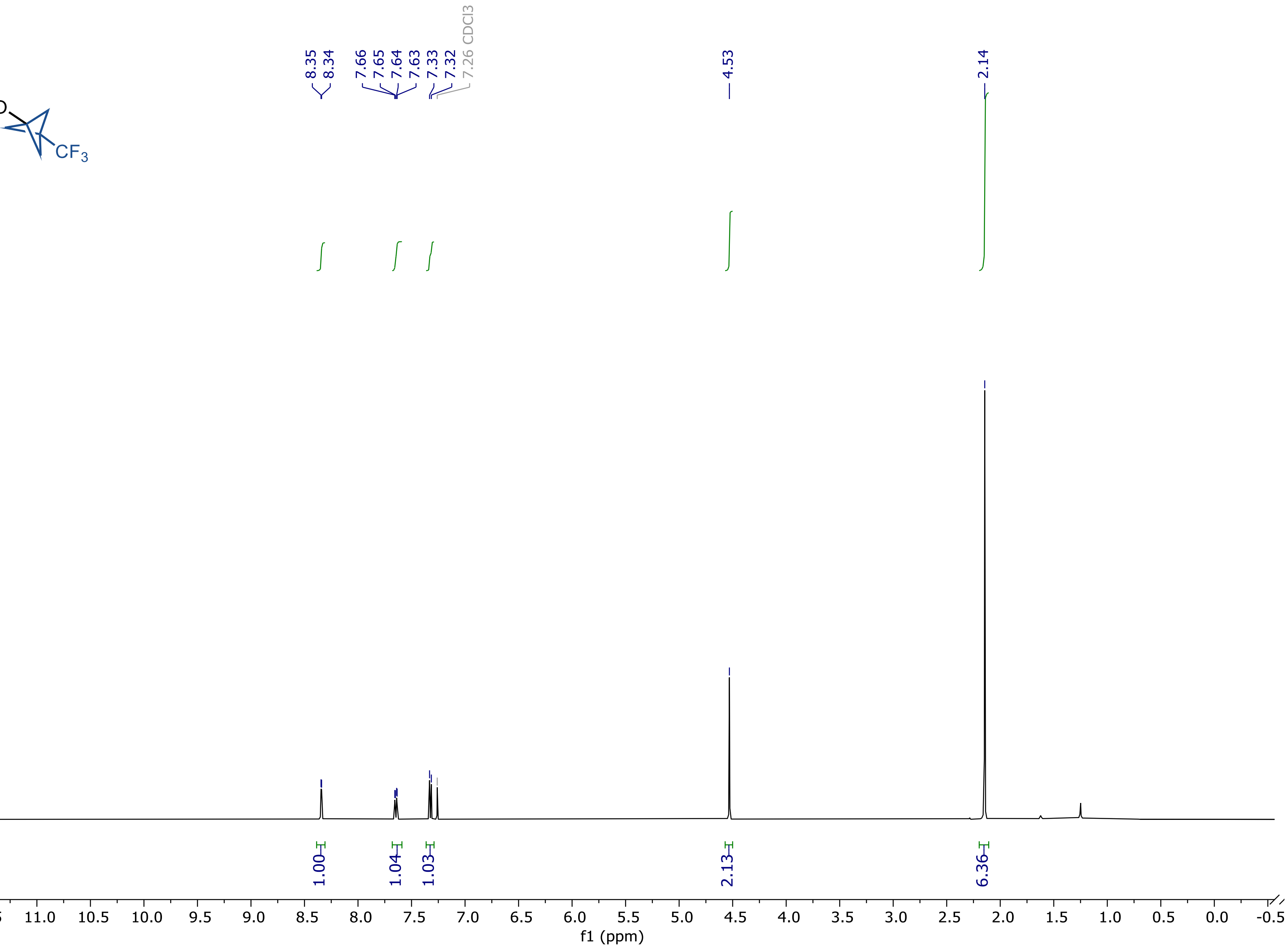

^{13}C NMR of bicyclo[1.1.1]pentylether 8

$CDCl_3$, 298 K

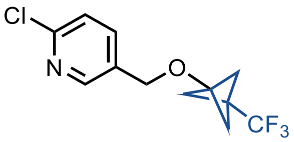

8

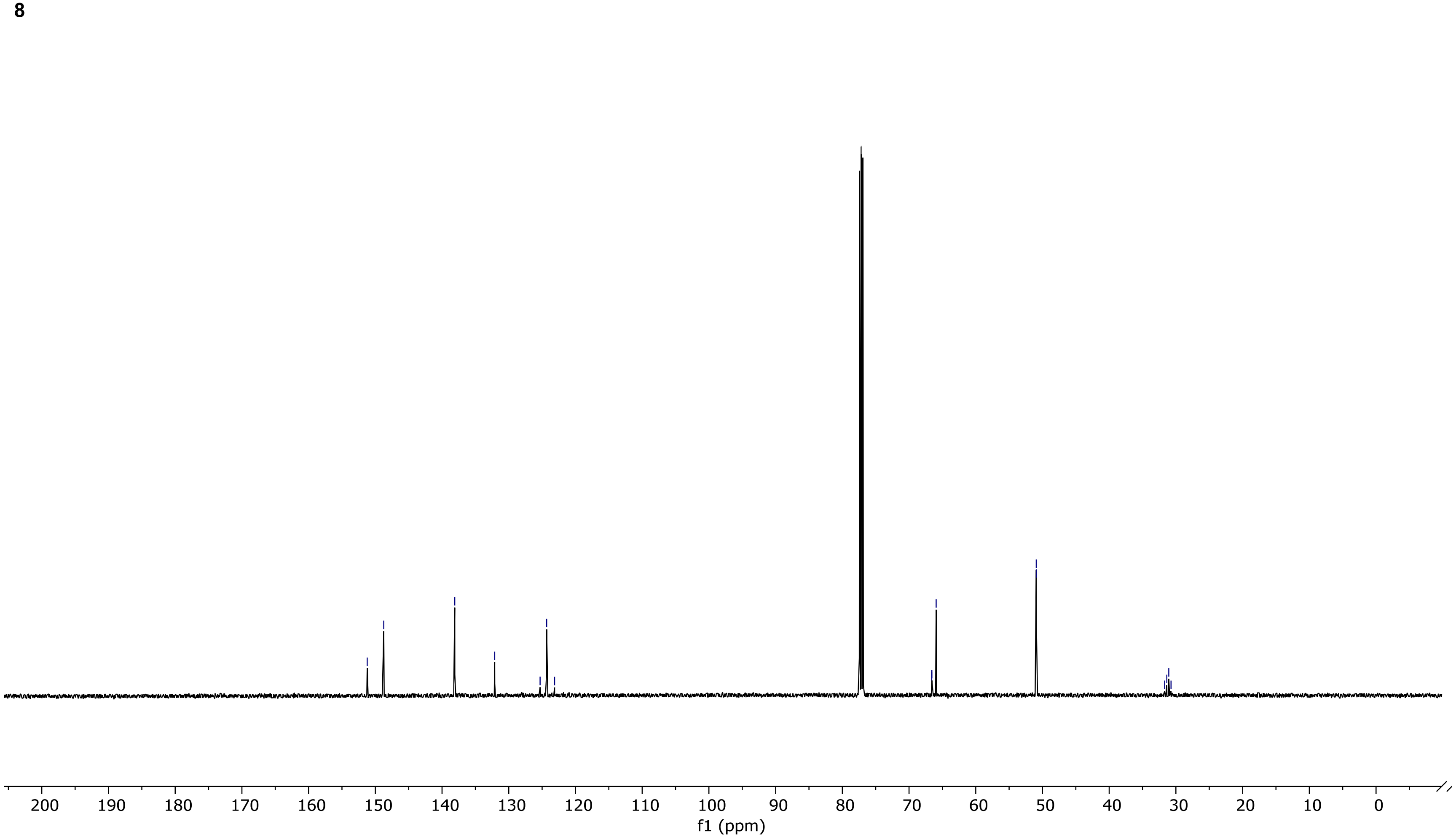

^{19}F NMR of bicyclo[1.1.1]pentylether 8

CDCl$_3$, 298 K

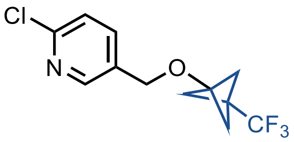

8

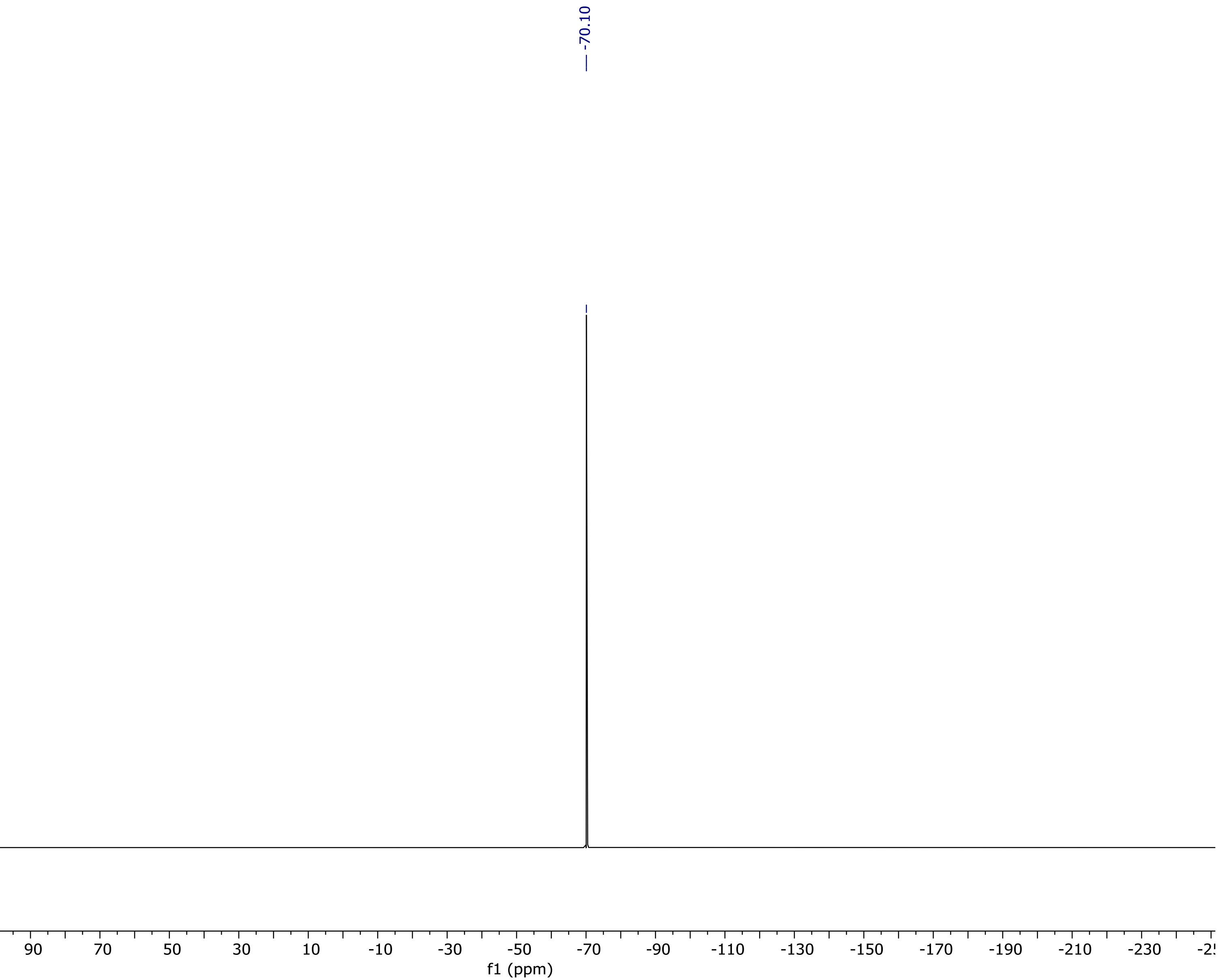

^{1}H NMR of bicyclo[1.1.1]pentylether 9

CDCl₃, 298 K

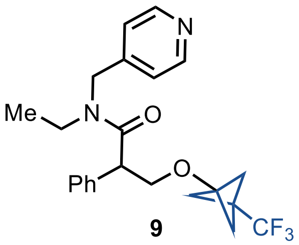

9

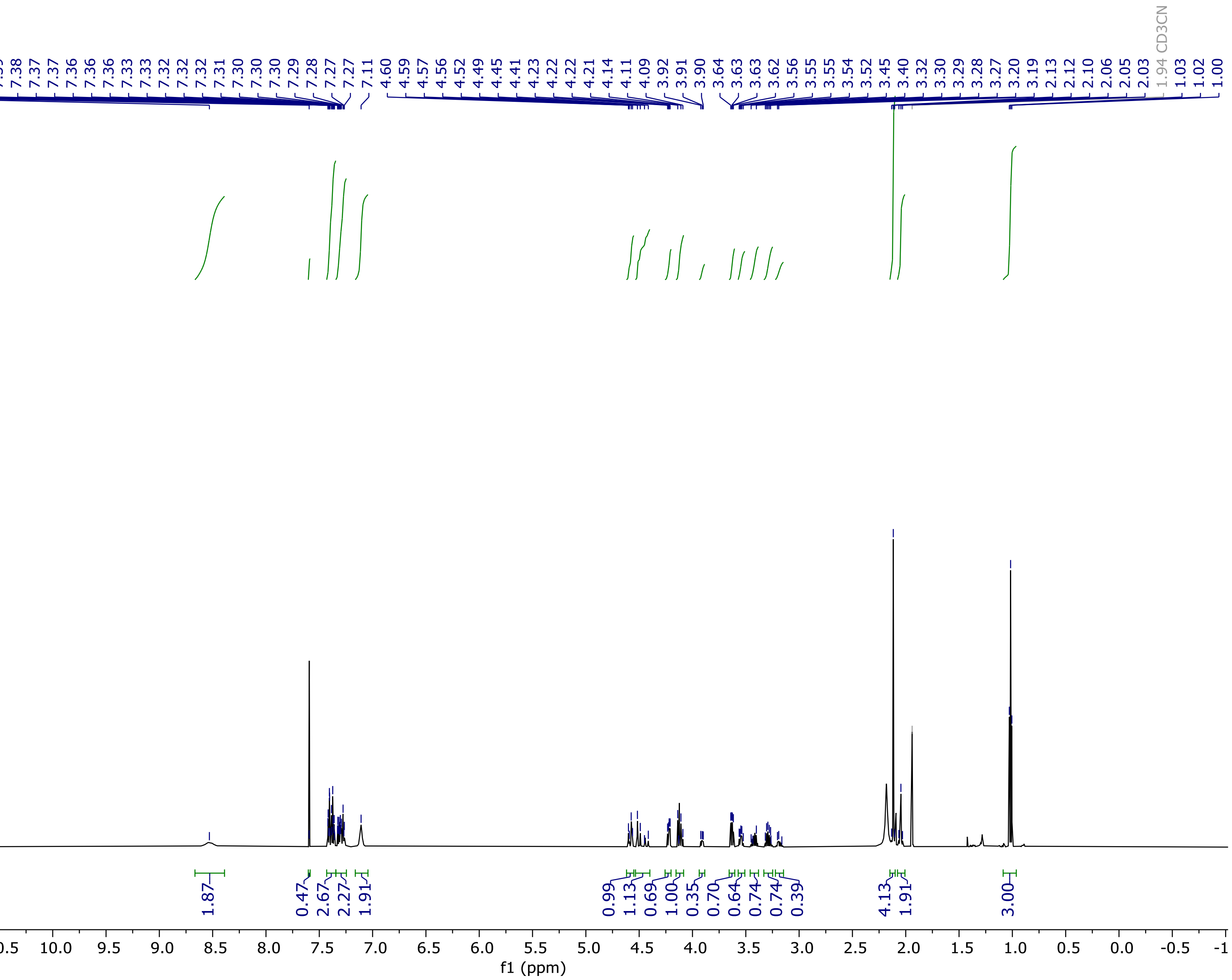

^{13}C NMR of bicyclo[1.1.1]pentylether 9

$CDCl_3$, 298 K

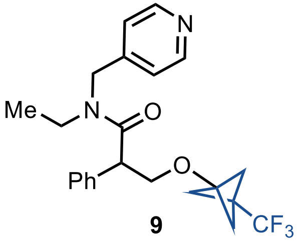

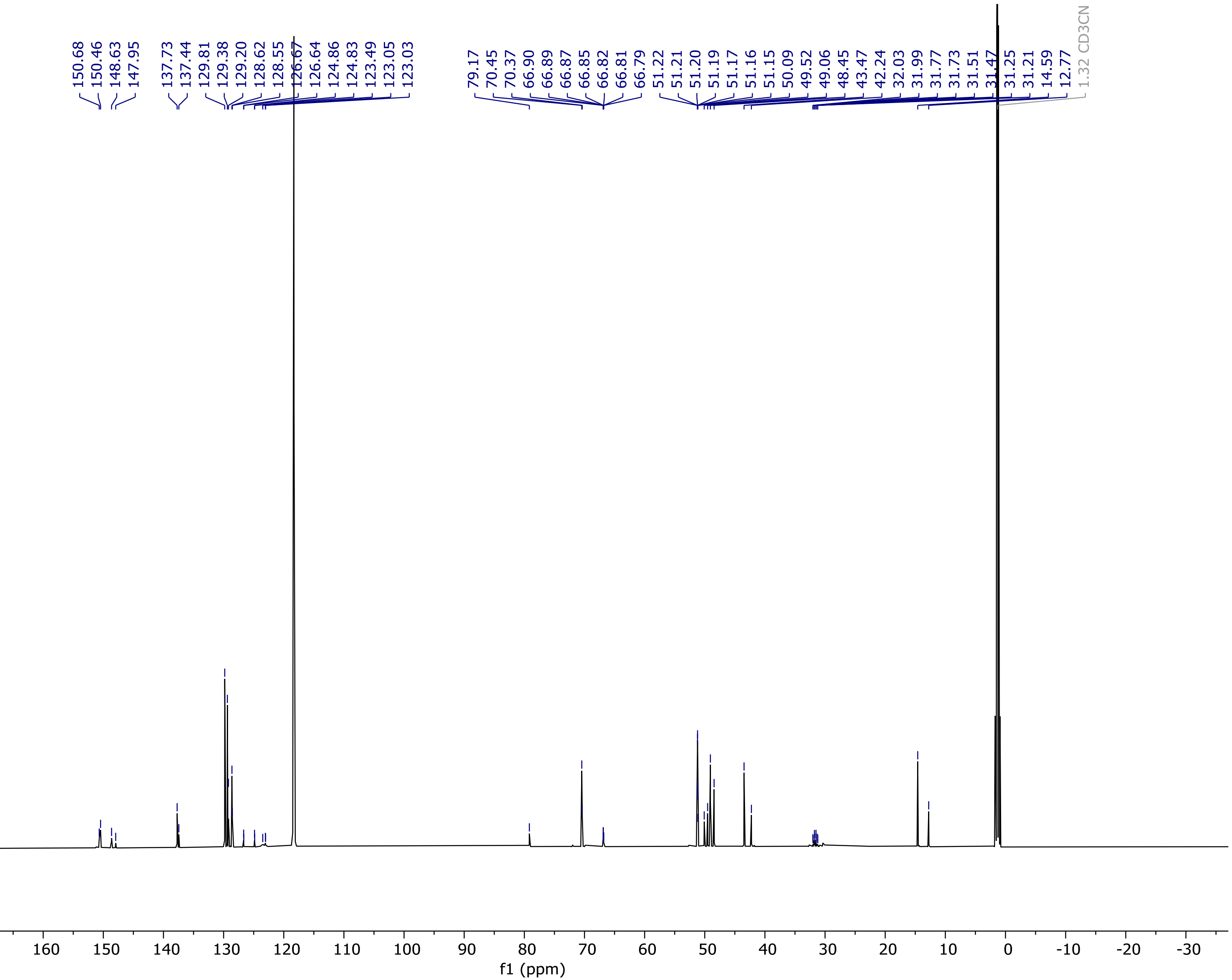

^{19}F NMR of bicyclo[1.1.1]pentylether 9

CDCl$_3$, 298 K

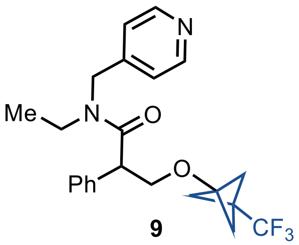

9

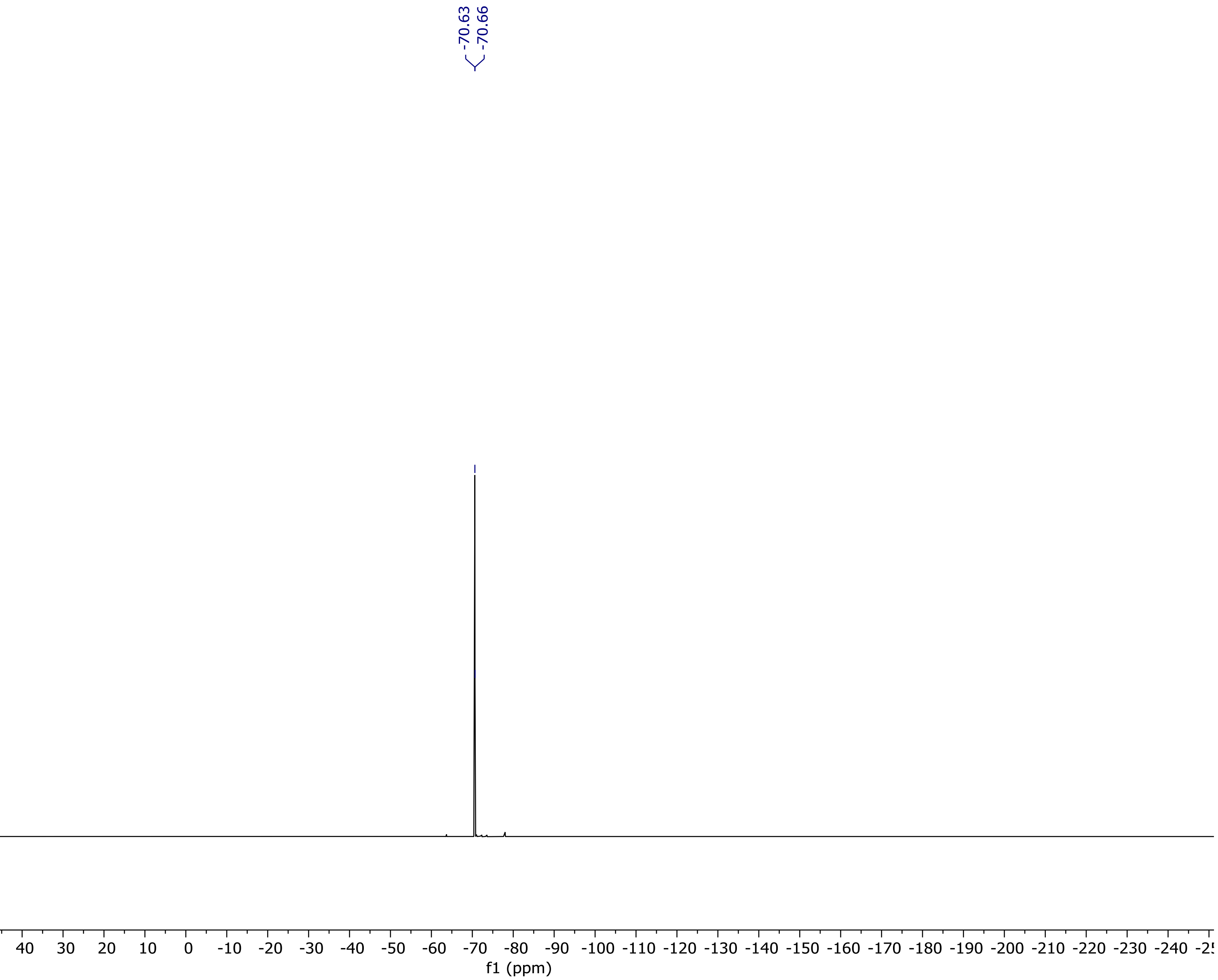

^{1}H NMR of bicyclo[1.1.1]pentylether 10

$CDCl_3$, 298 K

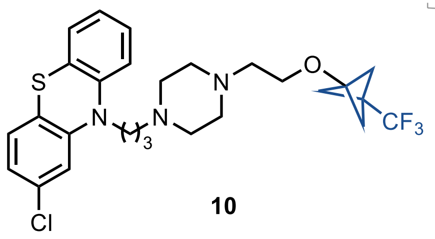

10

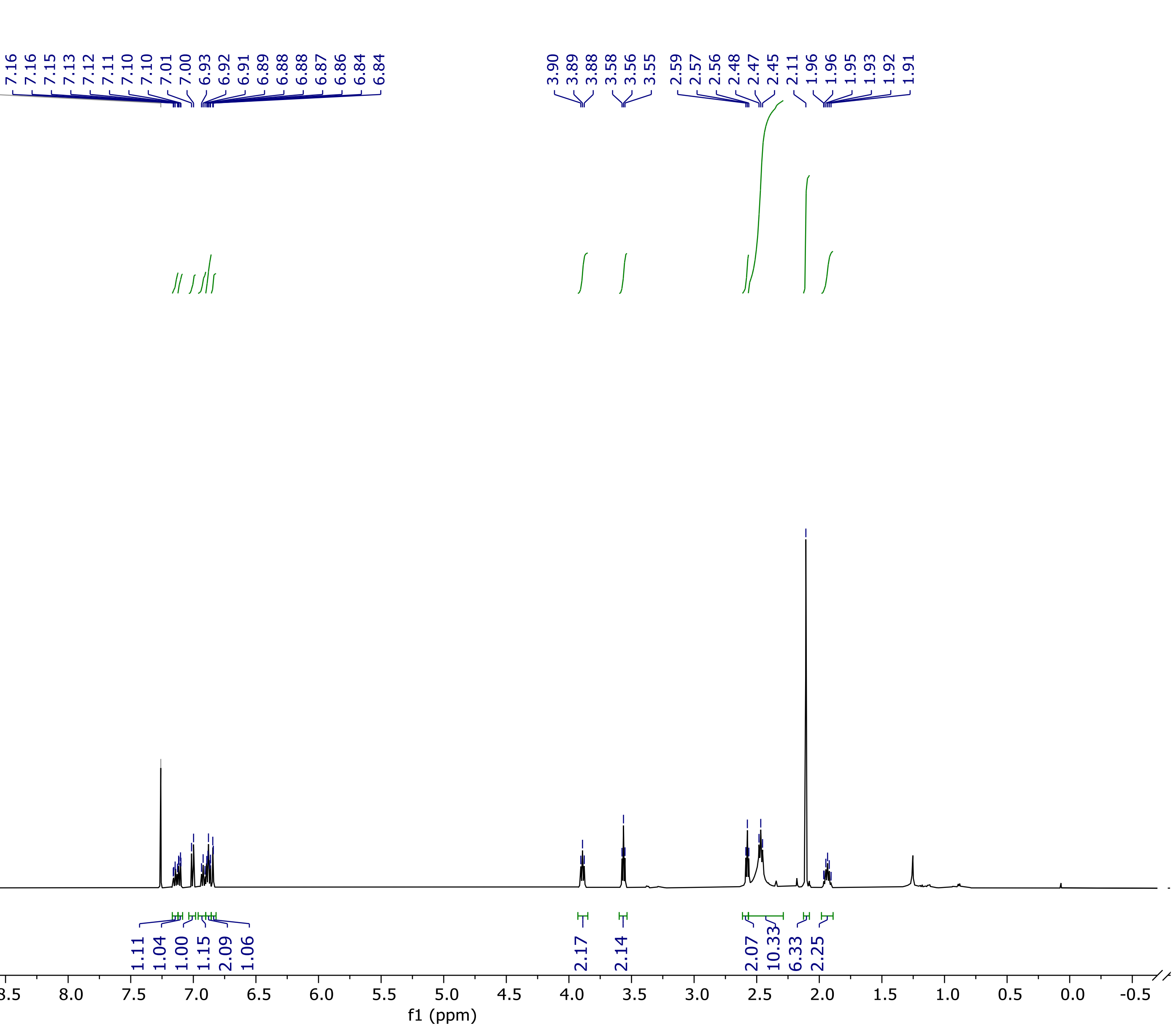

^{13}C NMR of bicyclo[1.1.1]pentylether 10

$CDCl_3$, 298 K

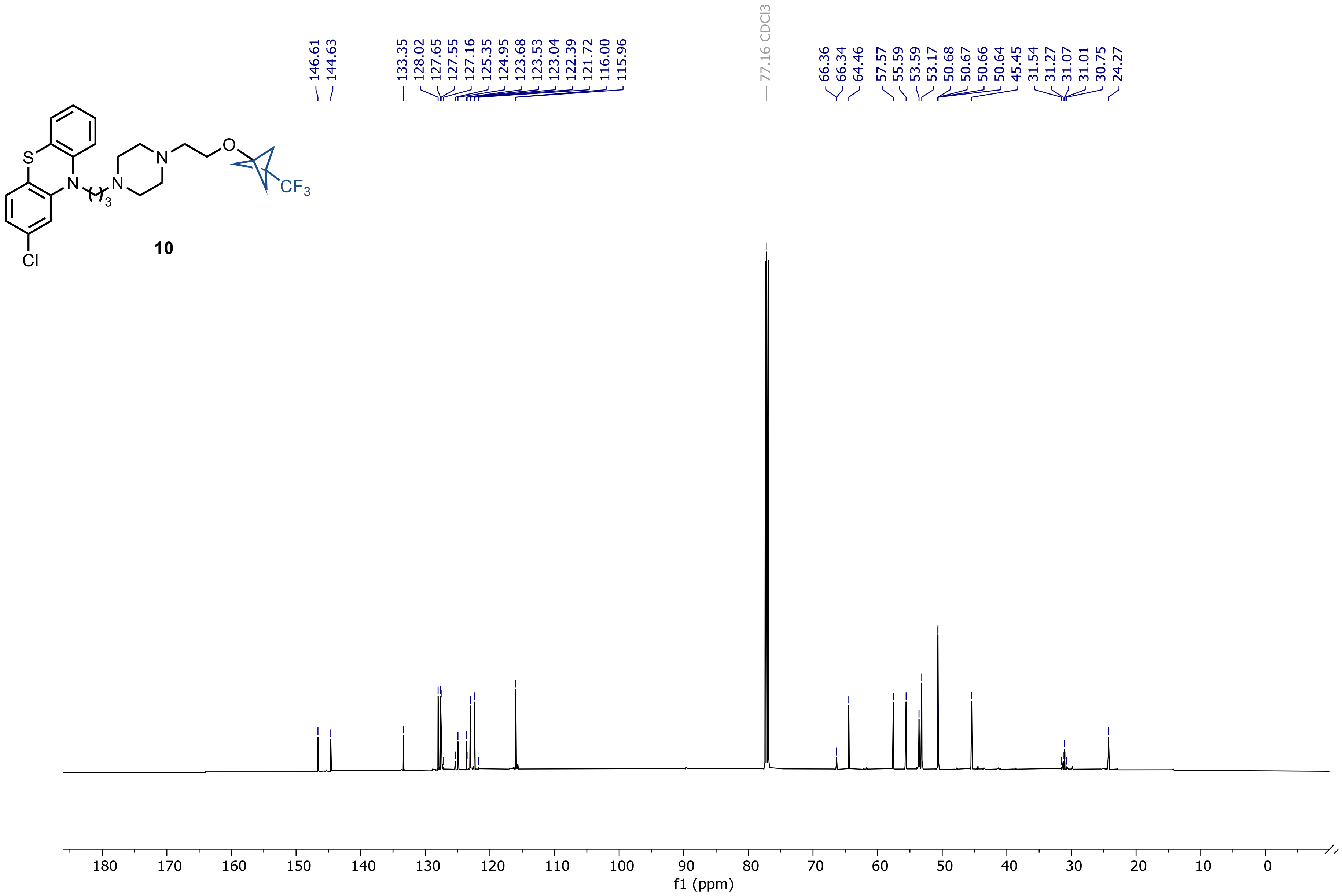

^{19}F NMR of bicyclo[1.1.1]pentylether 10

$CDCl_3$, 298 K

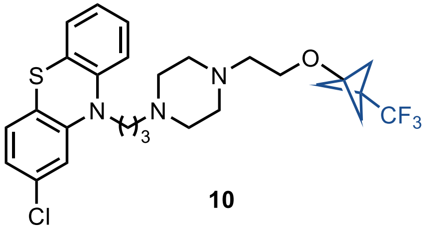

10

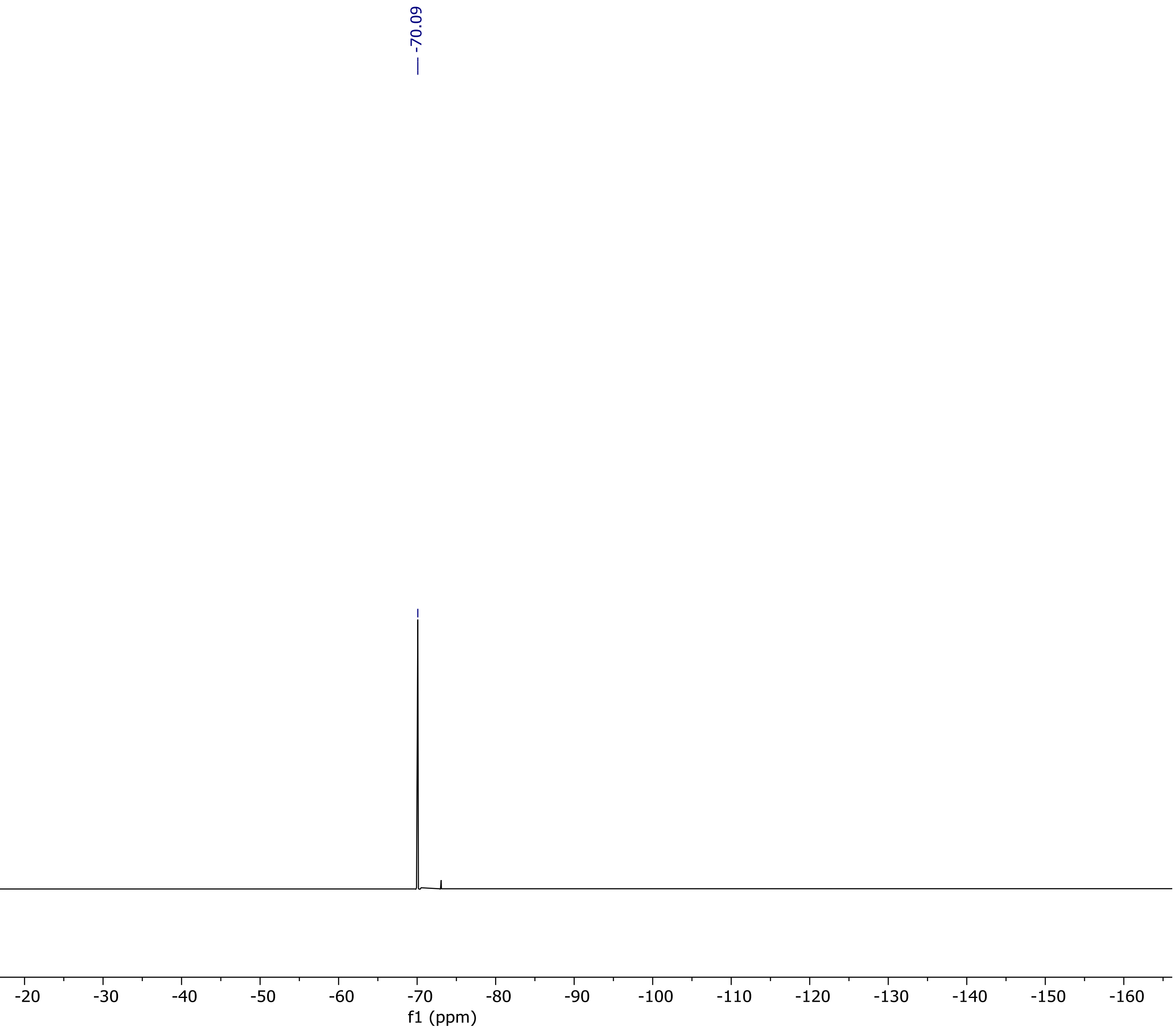

^{1}H NMR of bicyclo[1.1.1]pentylether 11

CDCl$_3$, 298 K

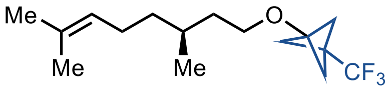

11

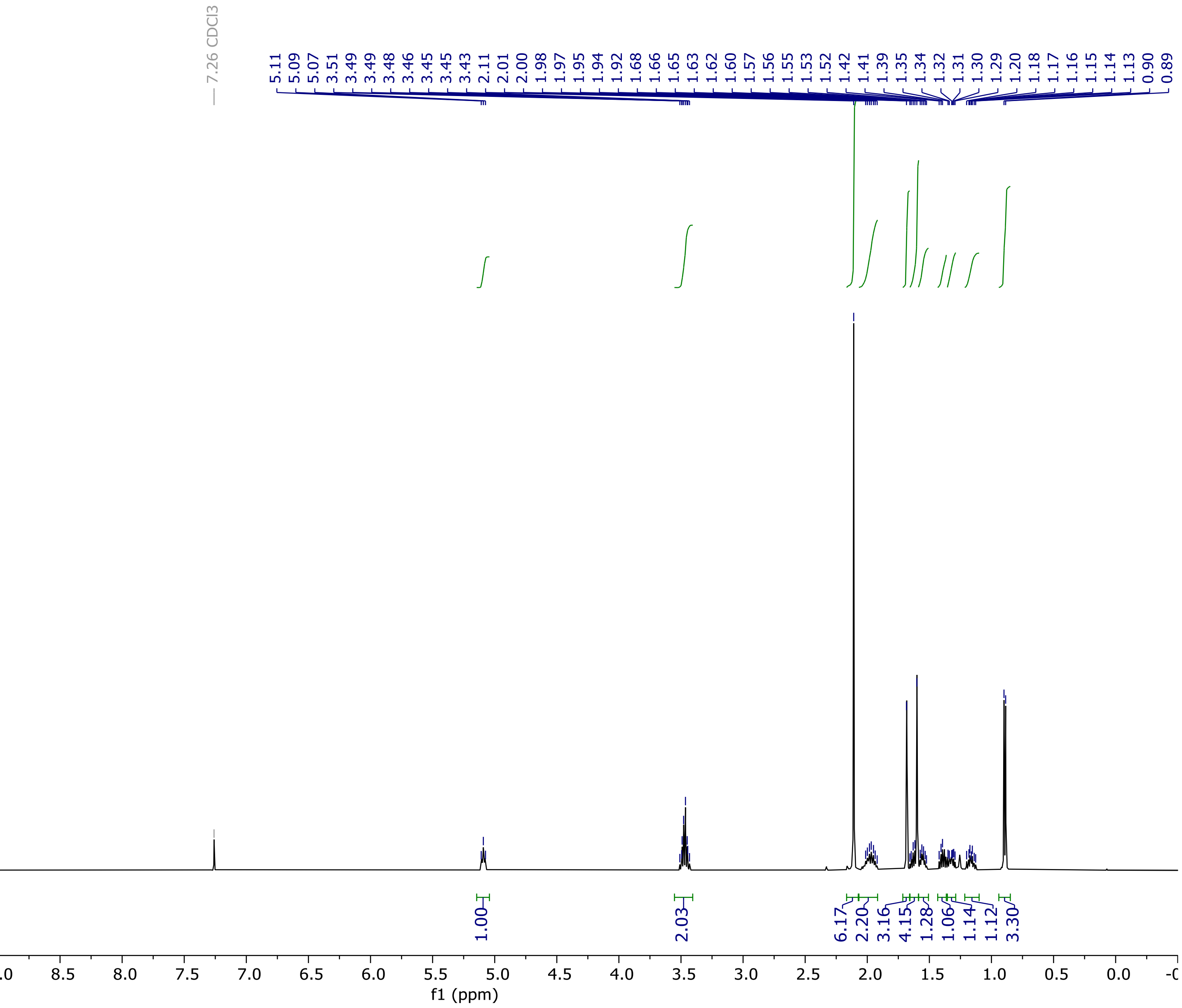

^{13}C NMR of bicyclo[1.1.1]pentylether 11

$CDCl_3$, 298 K

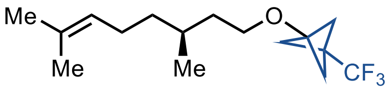

11

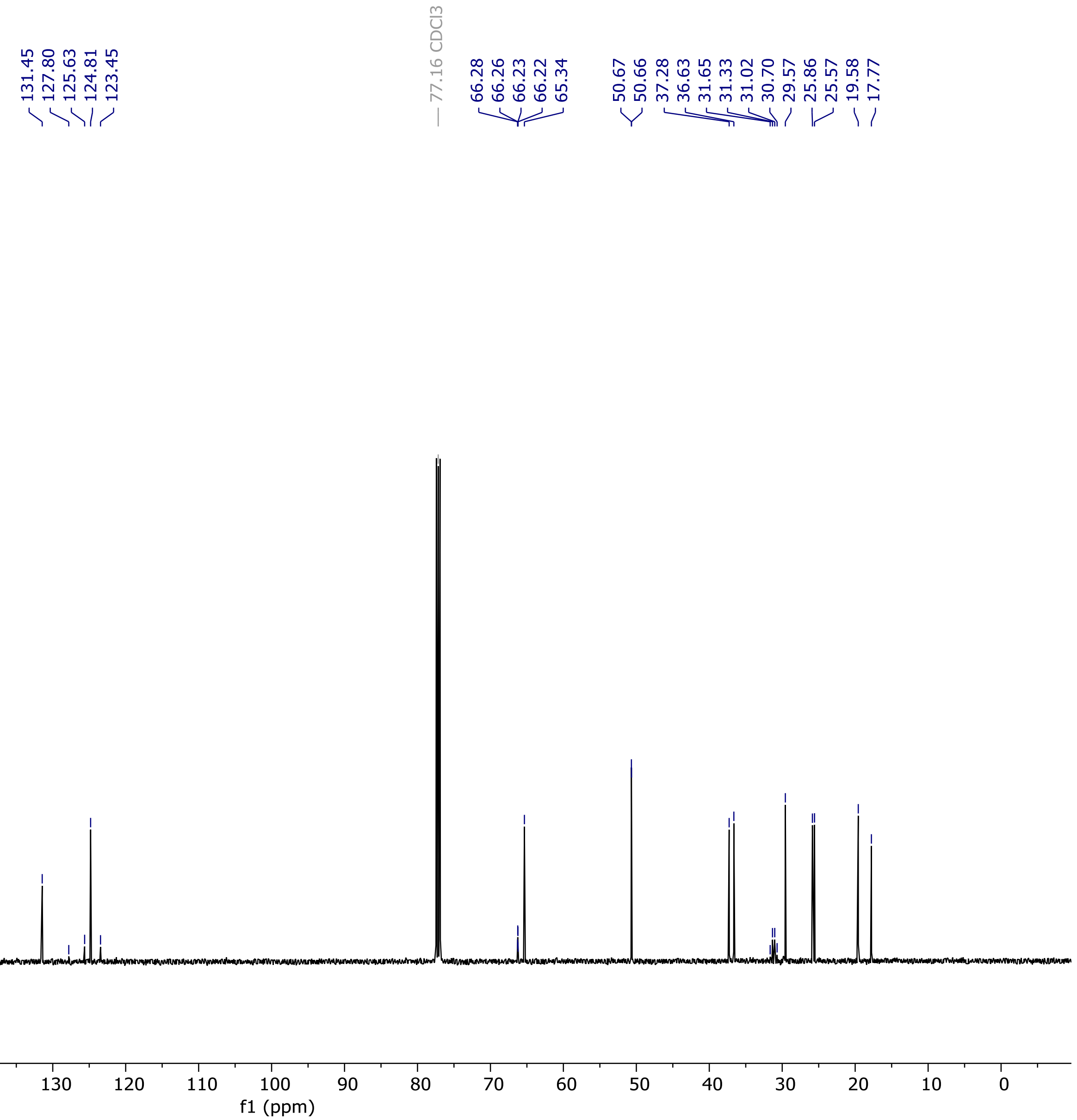

^{19}F NMR of bicyclo[1.1.1]pentylether 11

$CDCl_3$, 298 K

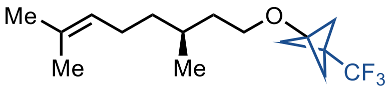

11

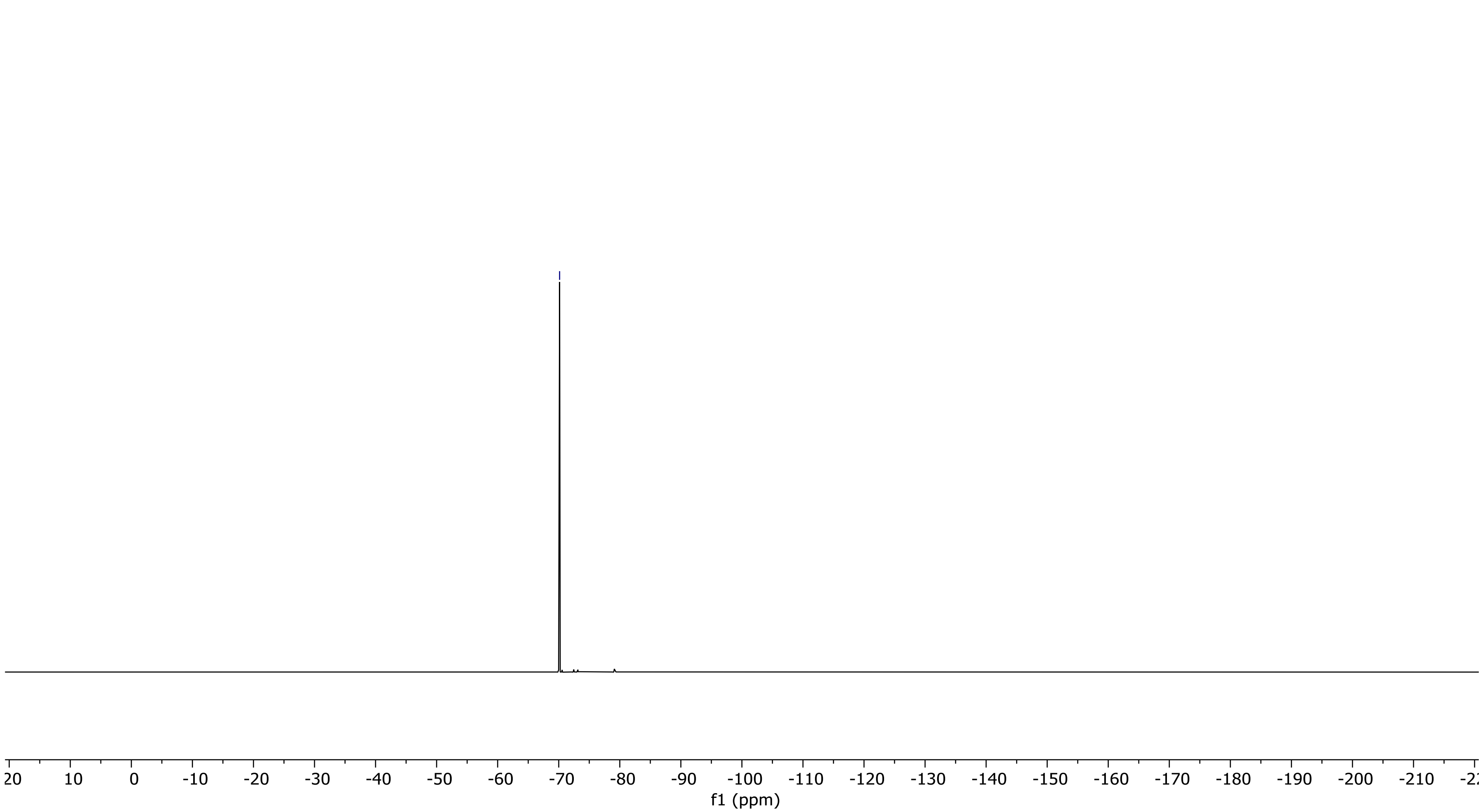

^{1}H NMR of bicyclo[1.1.1]pentylether 12

$CDCl_3$, 298 K

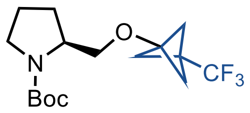

12

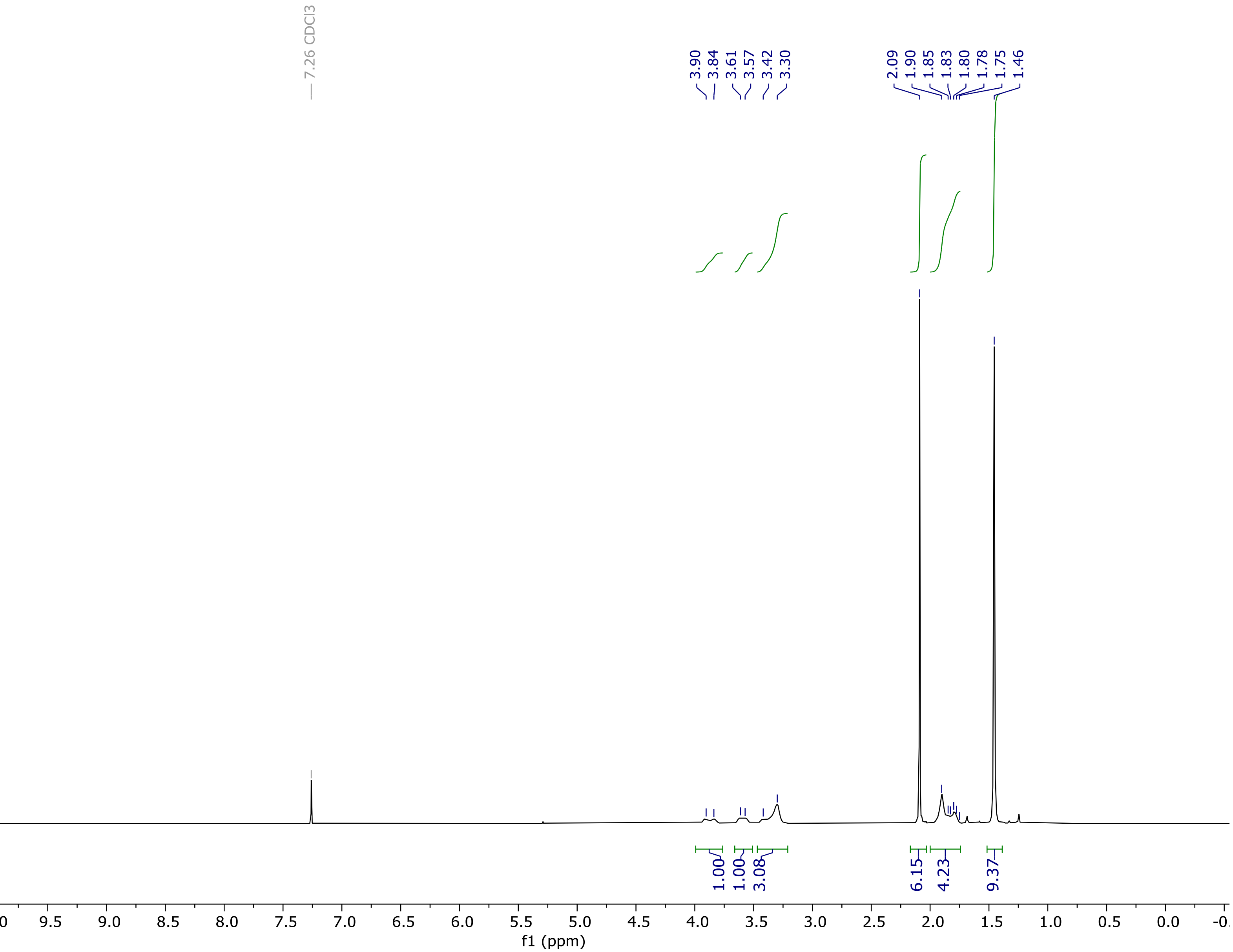

^{13}C NMR of bicyclo[1.1.1]pentylether 12

CDCl$_3$, 298 K

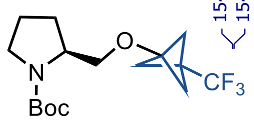

12

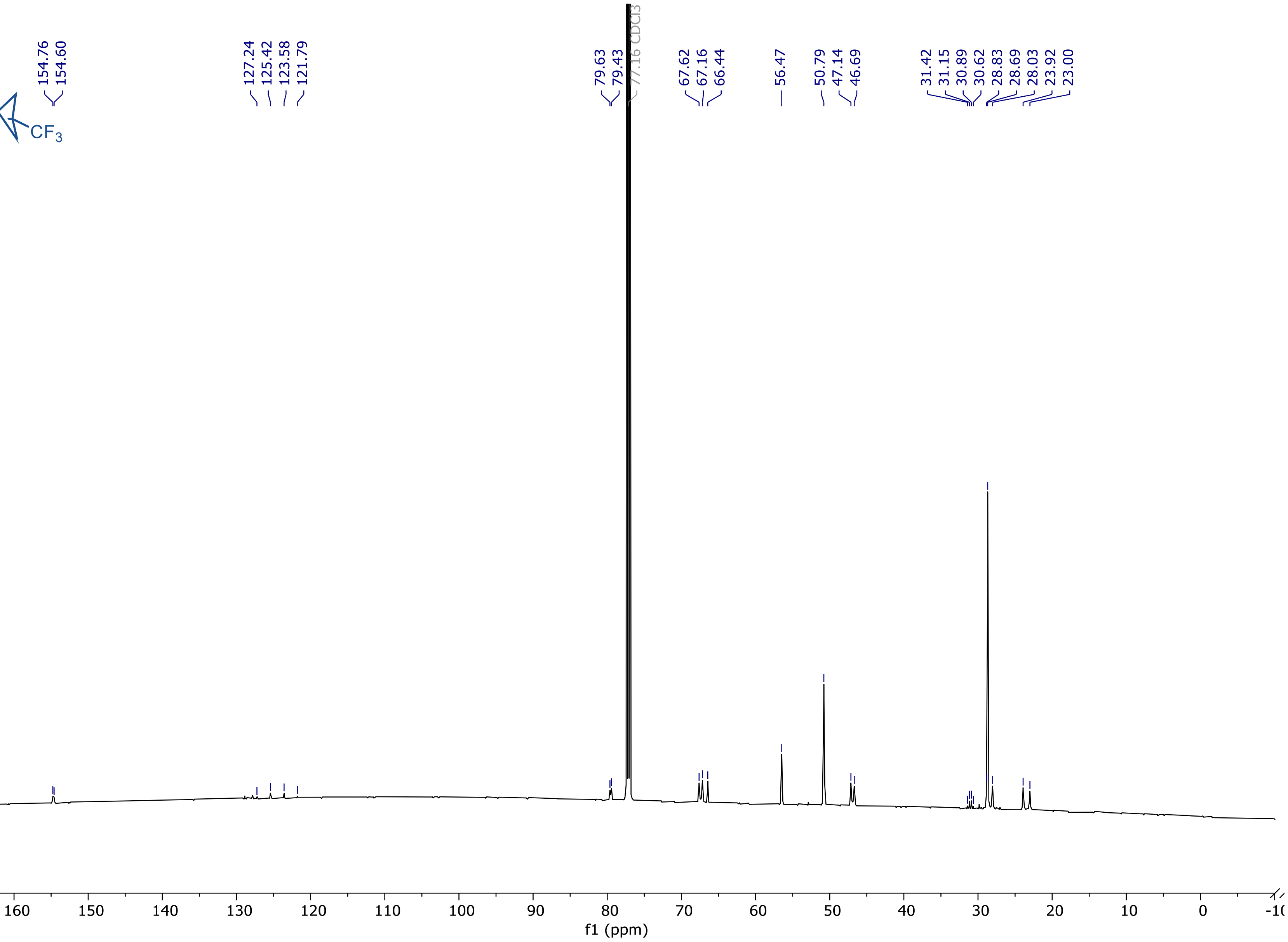

^{19}F NMR of bicyclo[1.1.1]pentylether 12

$CDCl_3$, 298 K

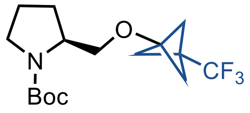

12

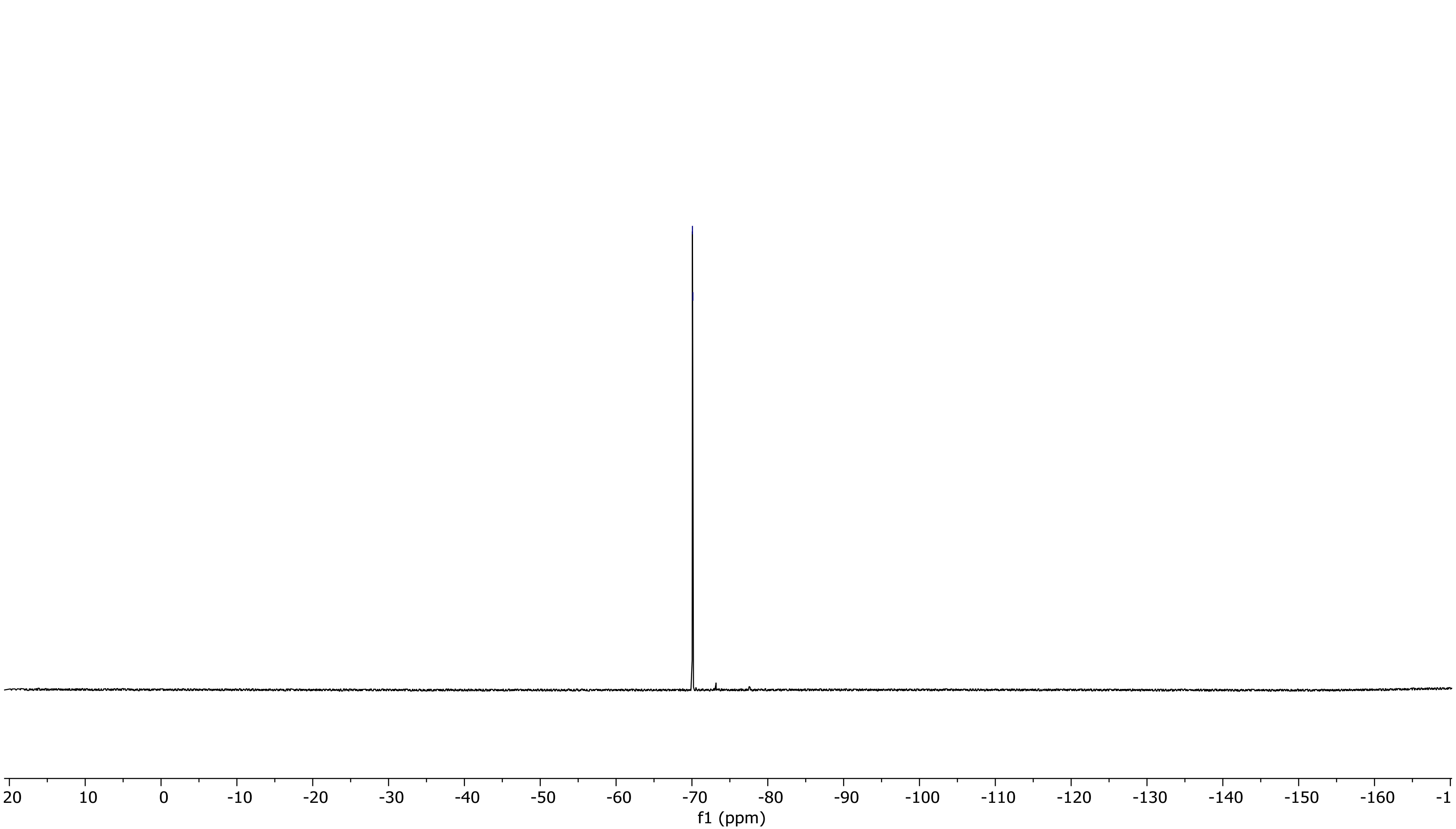

^{1}H NMR of bicyclo[1.1.1]pentylether 13

$CDCl_3$, 298 K

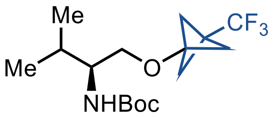

13

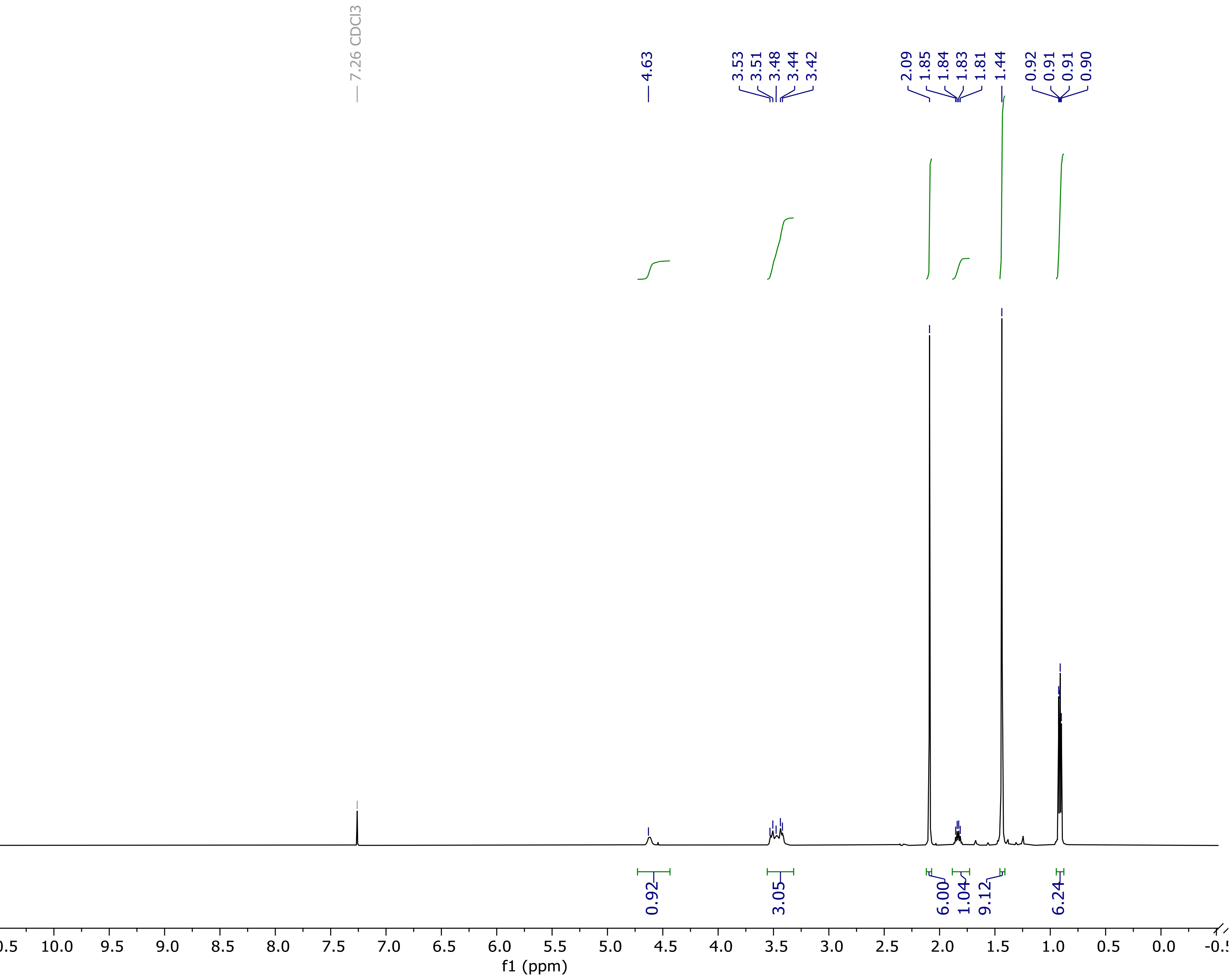

^{13}C NMR of bicyclo[1.1.1]pentylether 13

CDCl$_3$, 298 K

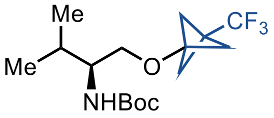

13

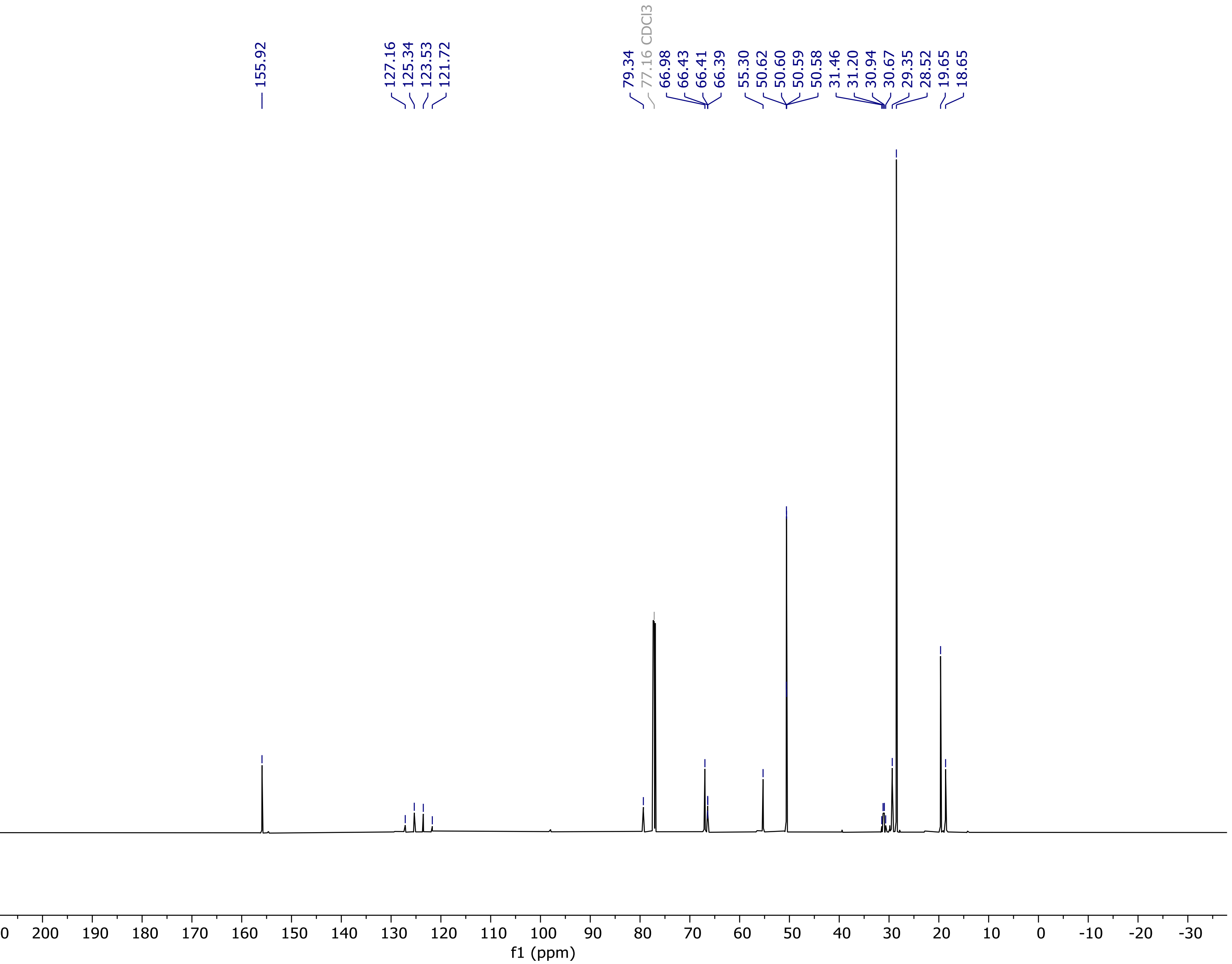

^{19}F NMR of bicyclo[1.1.1]pentylether 13

$CDCl_3$, 298 K

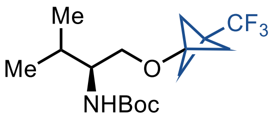

13

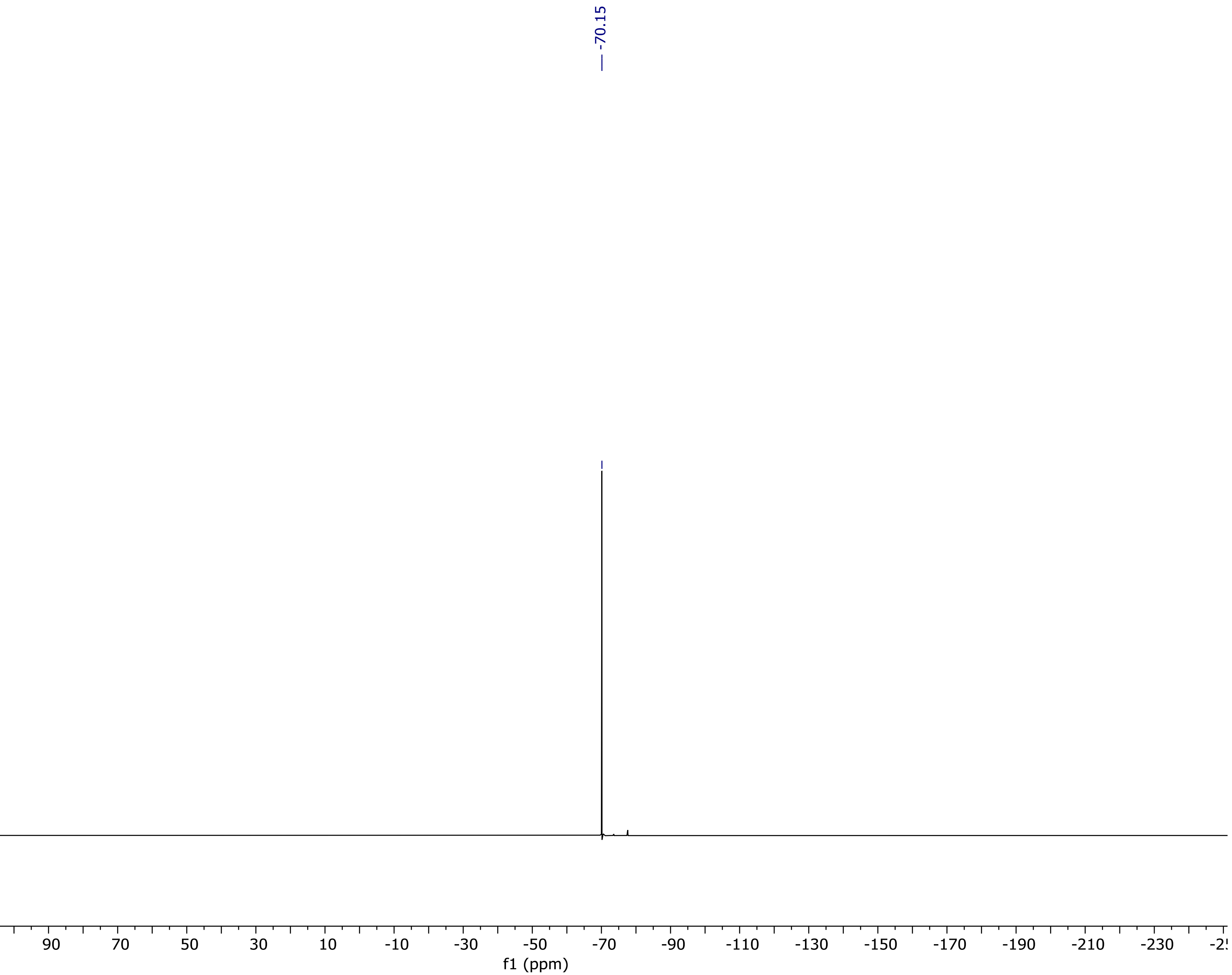

^{1}H NMR of bicyclo[1.1.1]pentylether 14

$CDCl_3$, 298 K

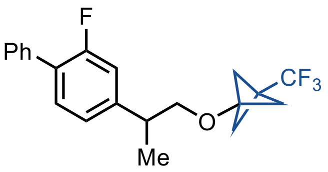

14

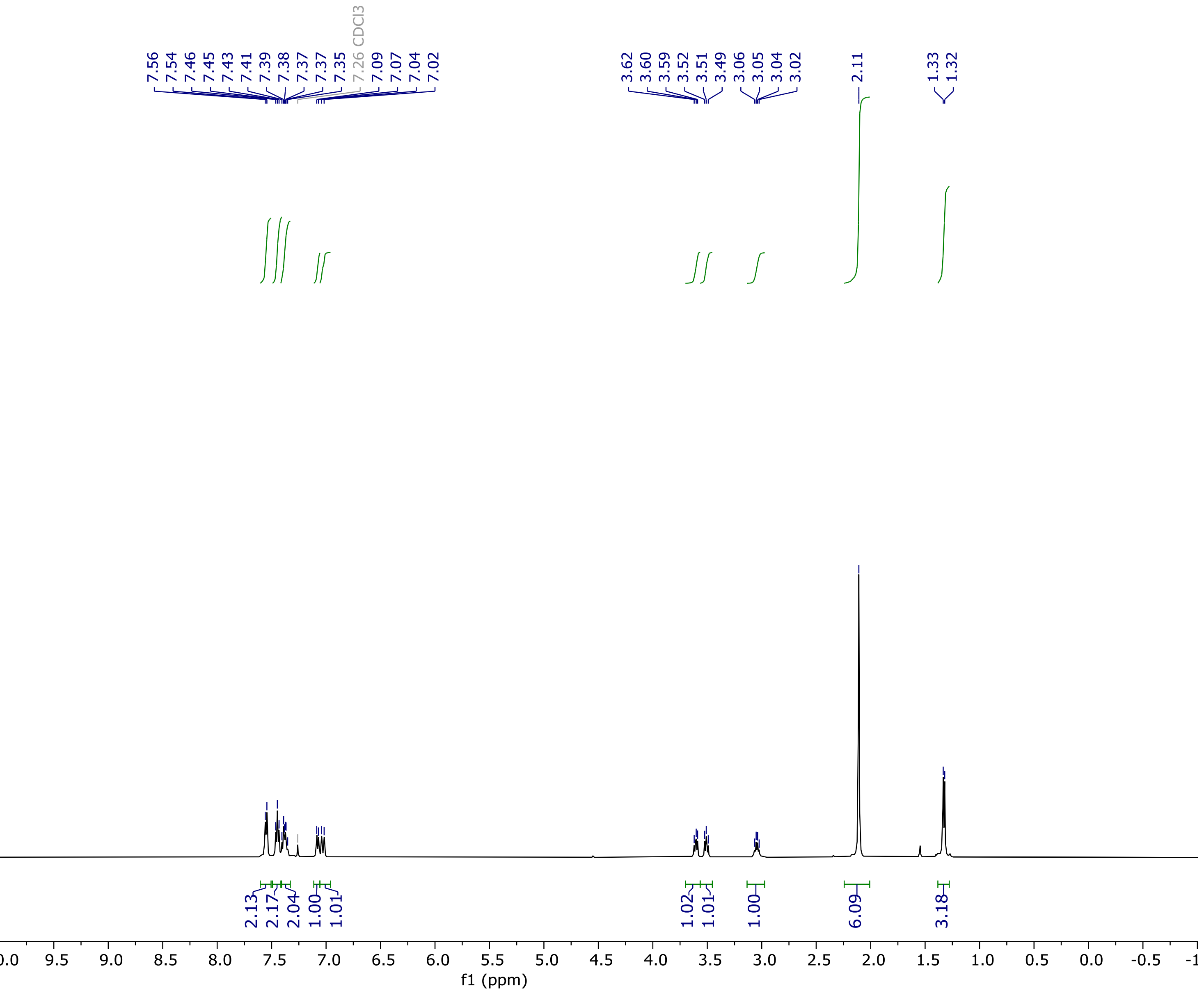

^{13}C NMR of bicyclo[1.1.1]pentylether 14

$CDCl_3$, 298 K

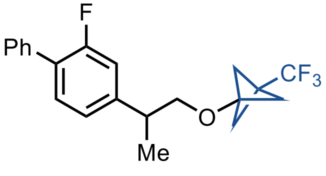

14

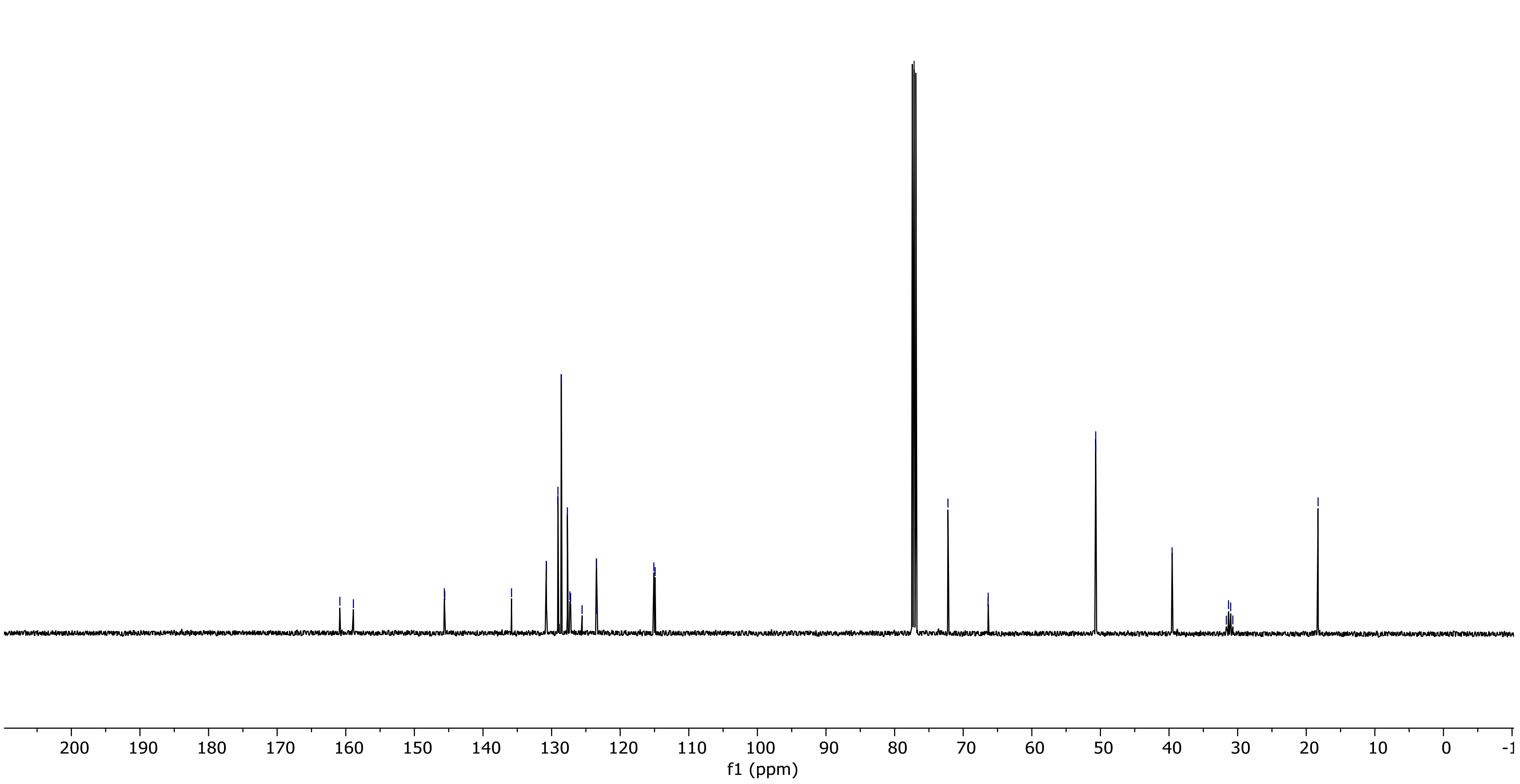

^{19}F NMR of bicyclo[1.1.1]pentylether 14

CDCl$_3$, 298 K

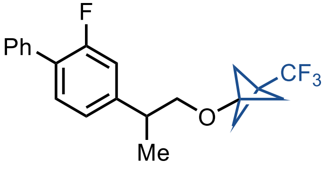

14

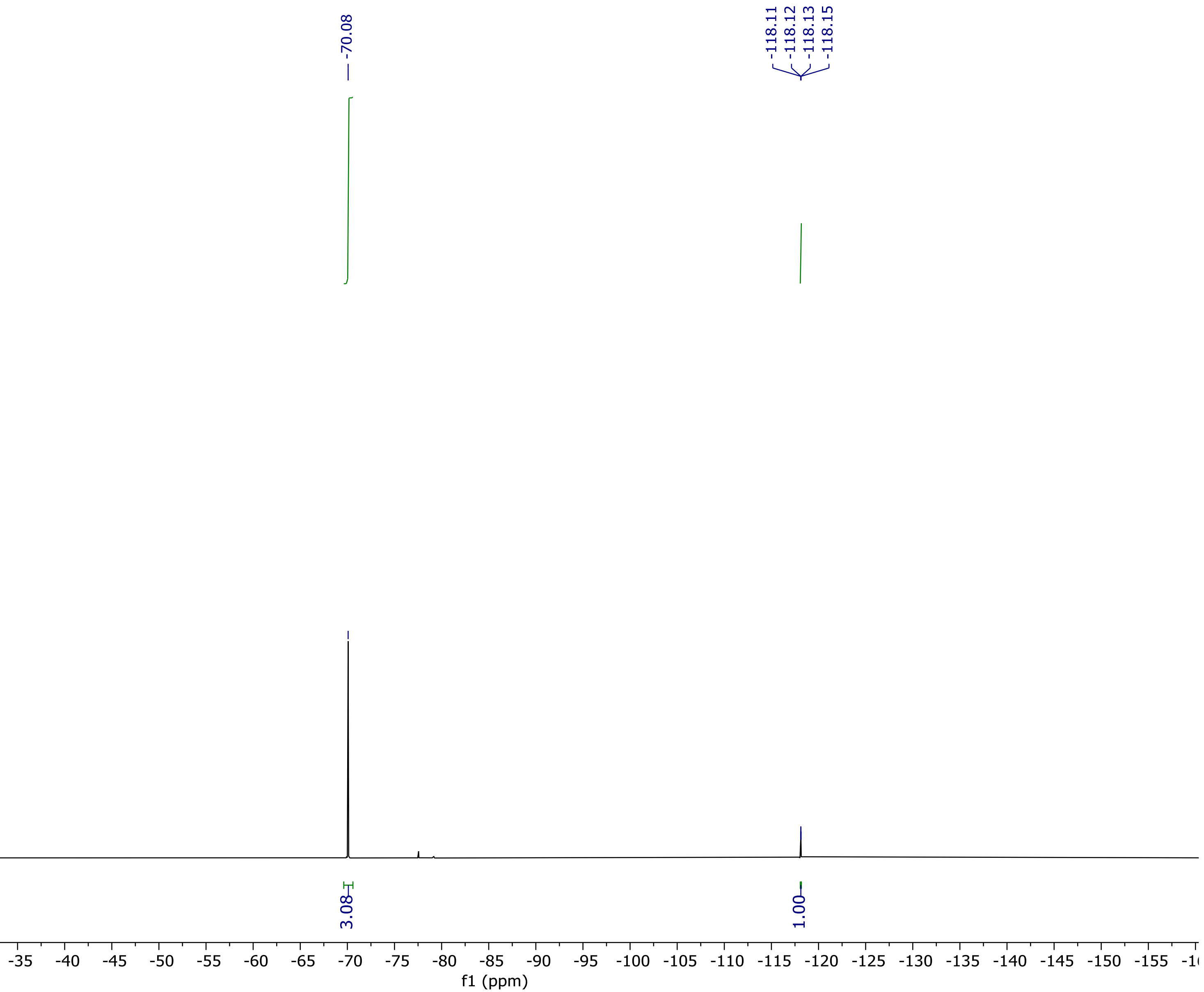

^{1}H NMR of bicyclo[1.1.1]pentylether 15

$CDCl_3$, 298 K

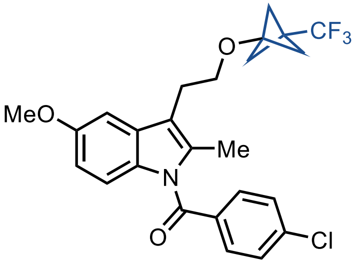

15

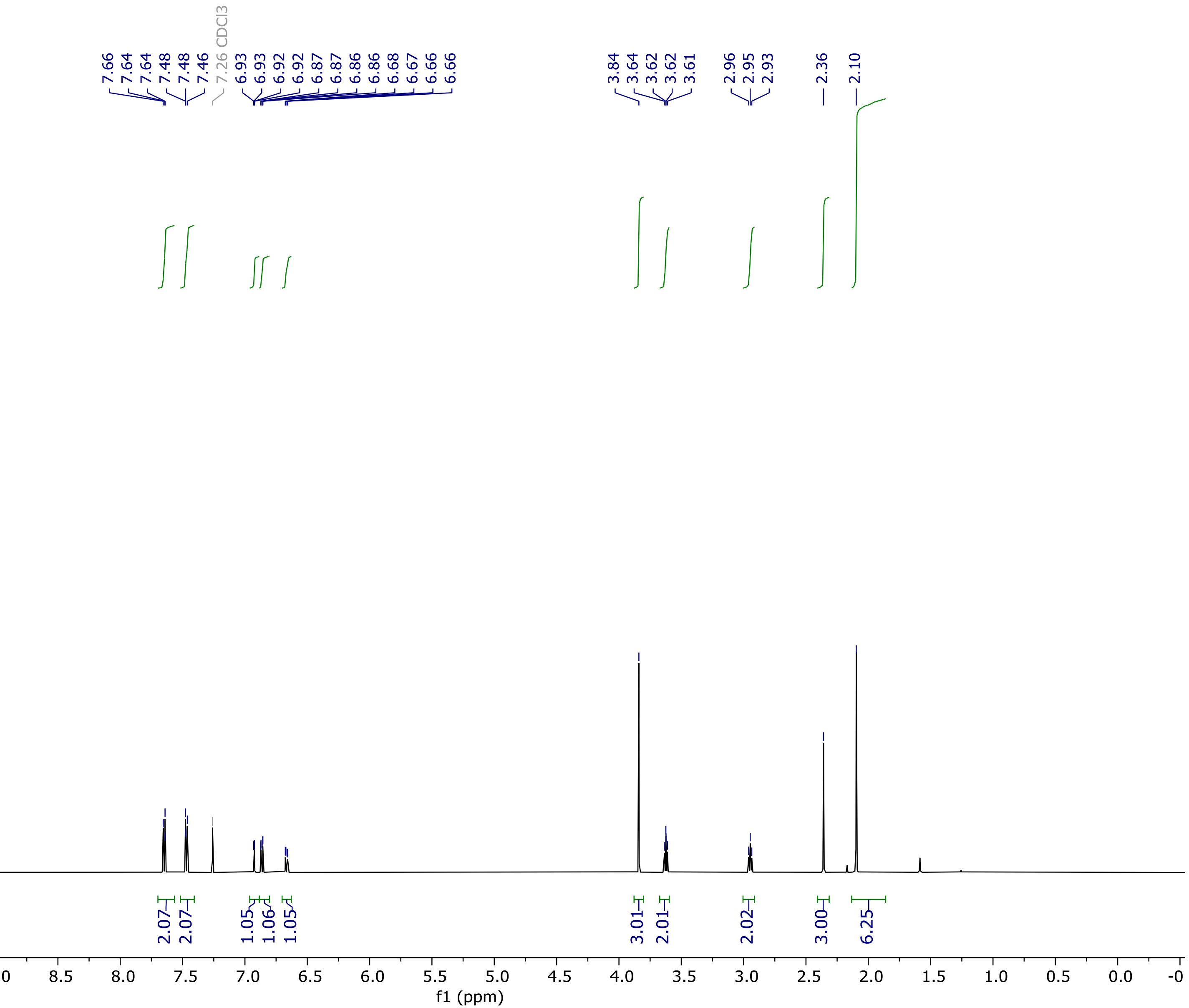

^{13}C NMR of bicyclo[1.1.1]pentylether 15

CDCl$_3$, 298 K

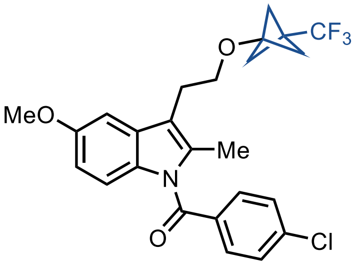

15

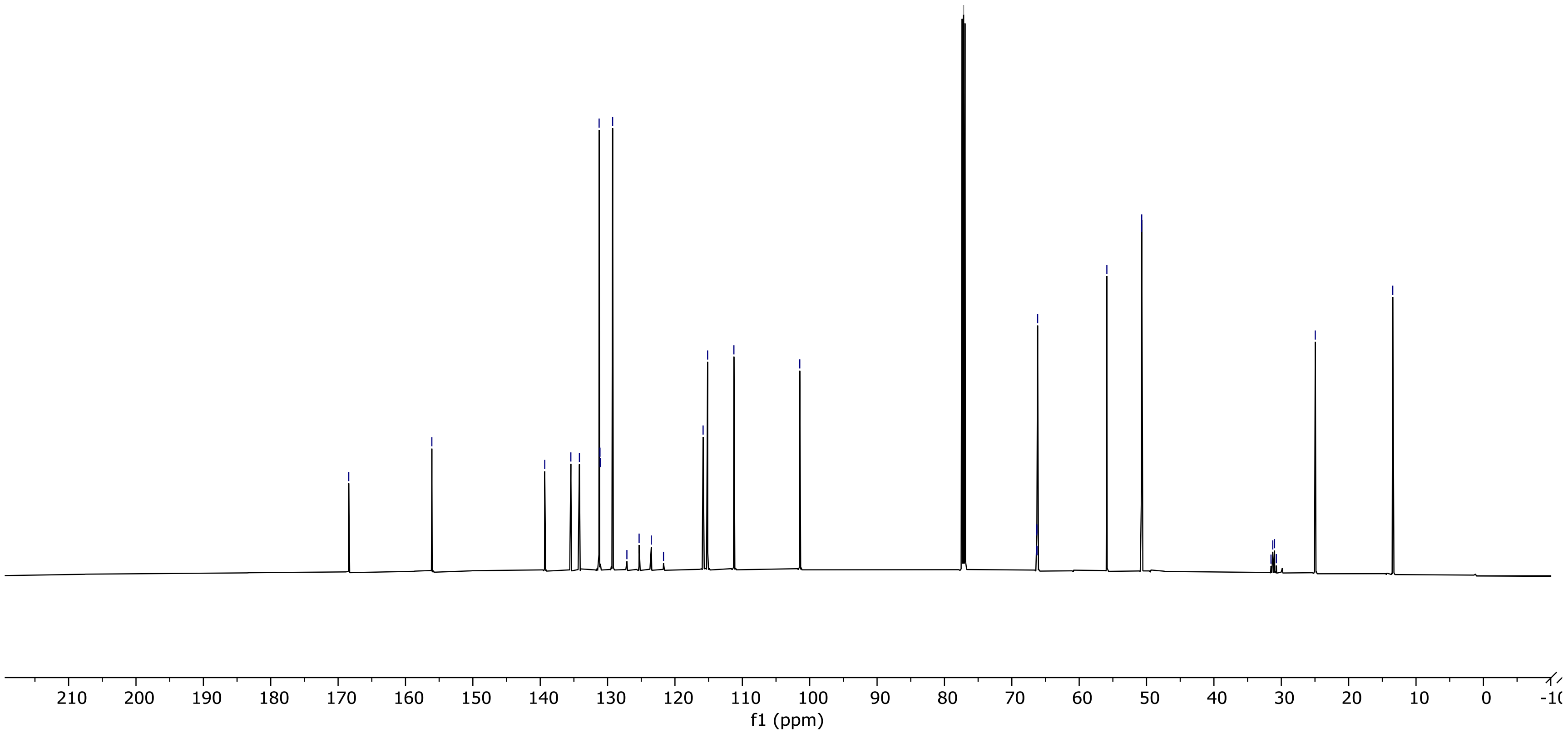

^{19}F NMR of bicyclo[1.1.1]pentylether 15

$CDCl_3$, 298 K

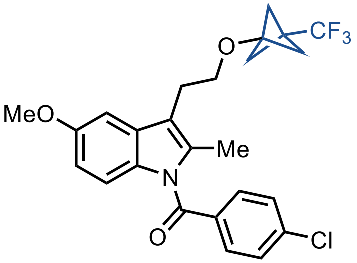

15

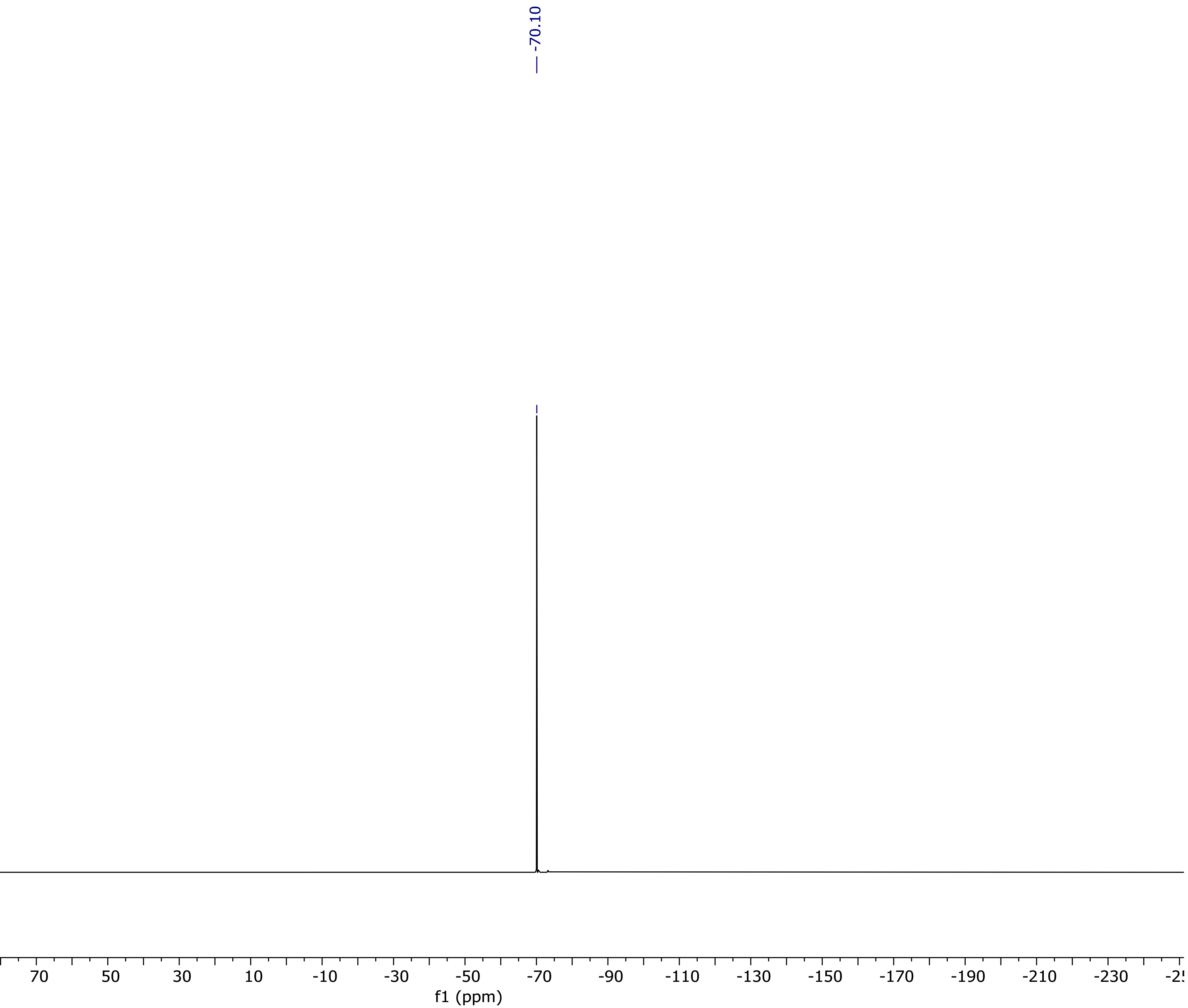

^{1}H NMR of bicyclo[1.1.1]pentylether 16

$CDCl_3$, 298 K

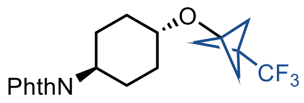

16

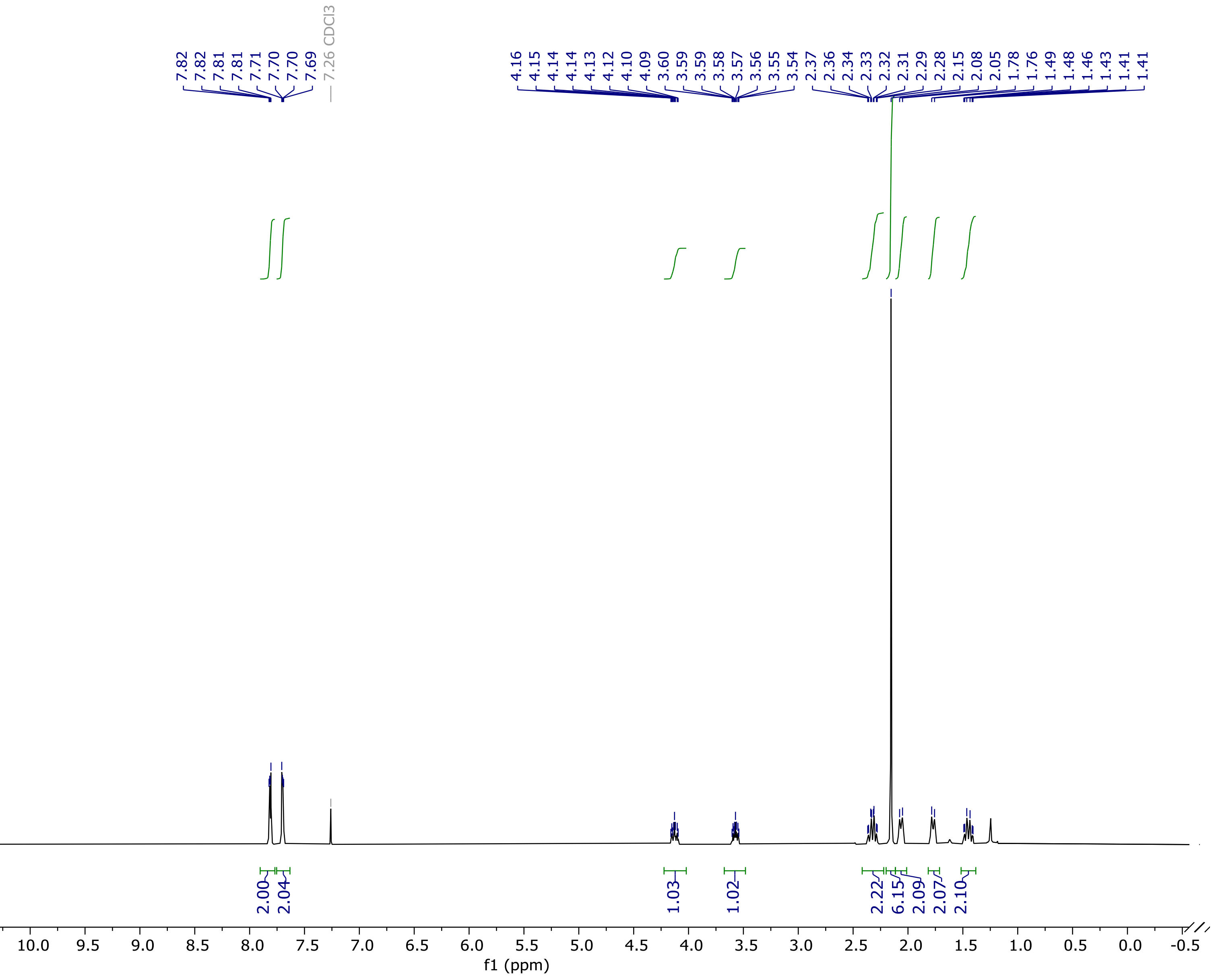

^{13}C NMR of bicyclo[1.1.1]pentylether 16

$CDCl_3$, 298 K

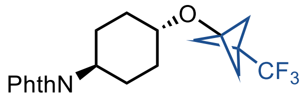

16

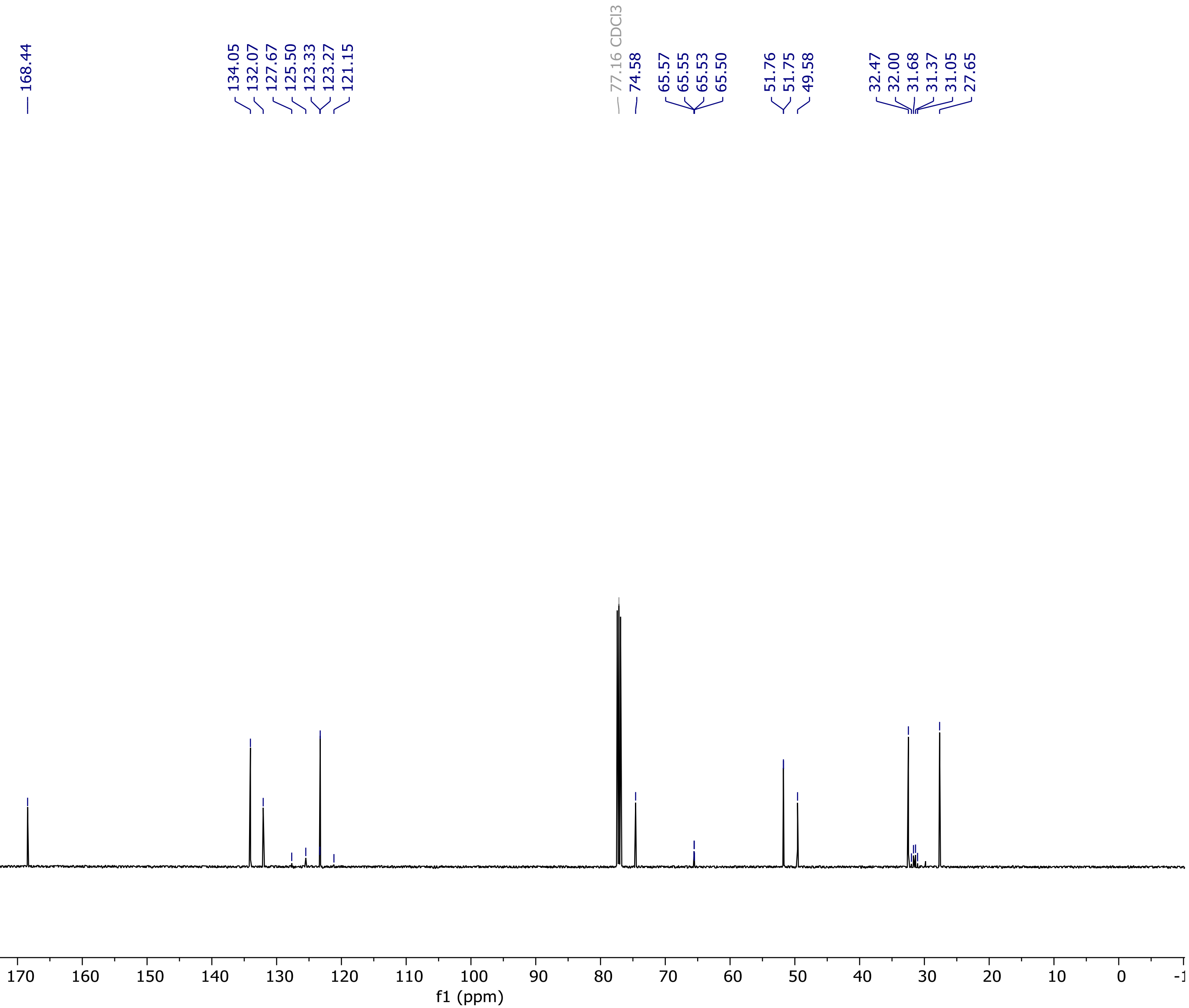

^{19}F NMR of bicyclo[1.1.1]pentylether 16

$CDCl_3$, 298 K

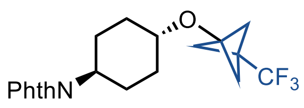

16

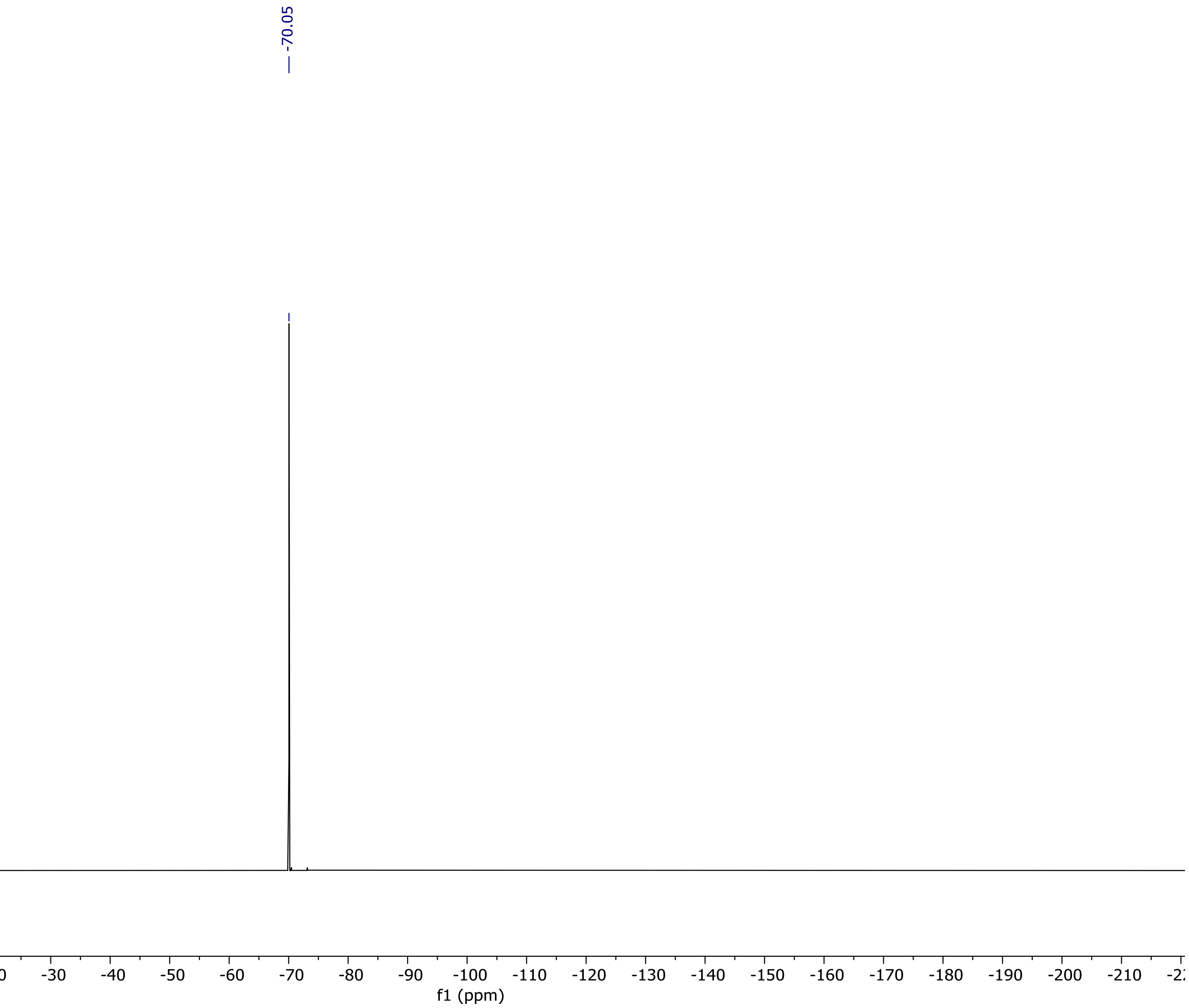

^{1}H NMR of bicyclo[1.1.1]pentylether 17

CDCl3, 298 K

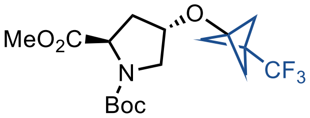

17

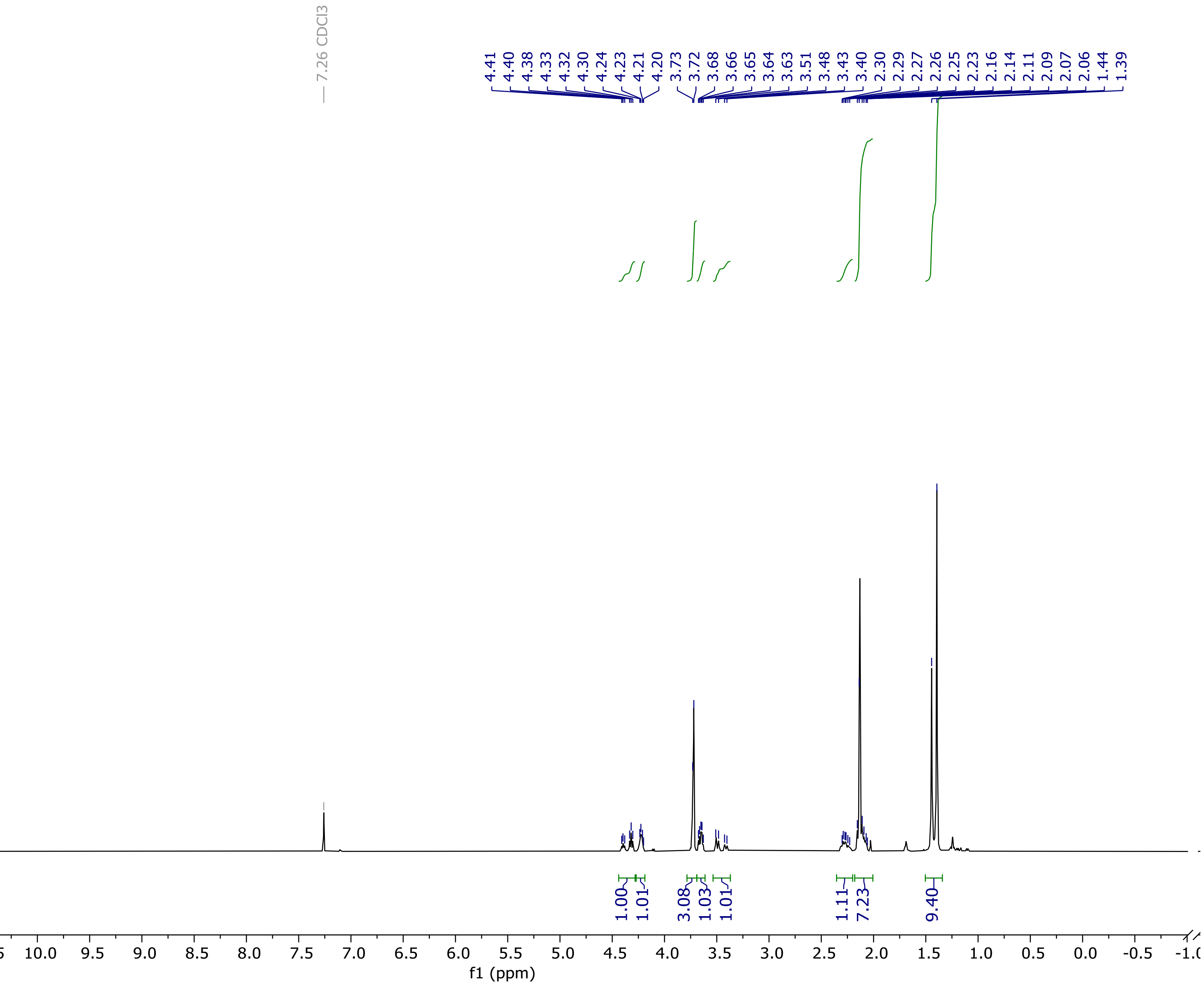

^{13}C NMR of bicyclo[1.1.1]pentylether 17

CDCl₃, 298 K

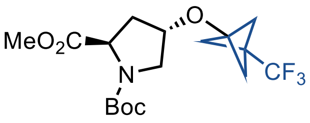

17

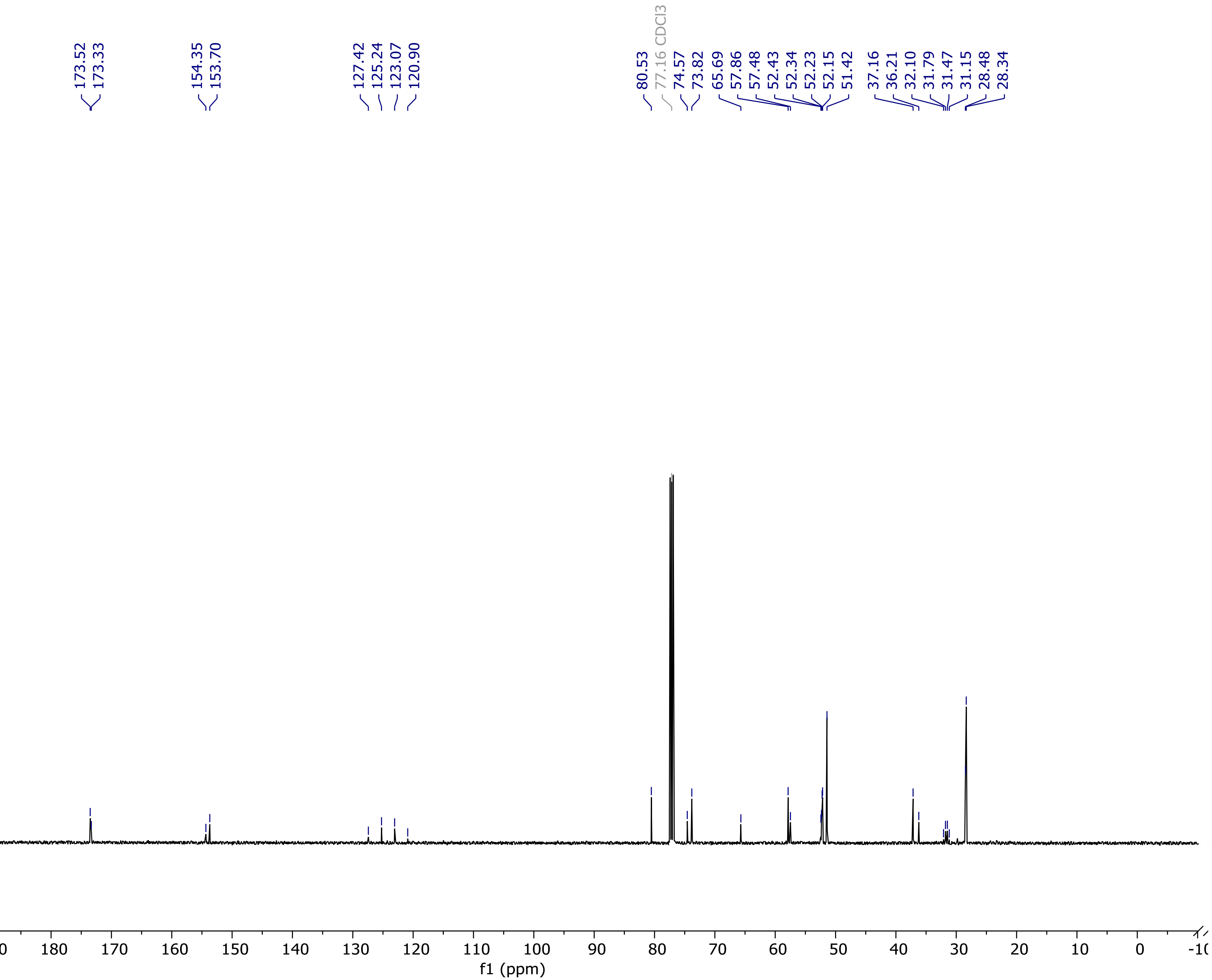

^{19}F NMR of bicyclo[1.1.1]pentylether 17

$CDCl_3$, 298 K

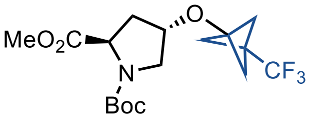

17

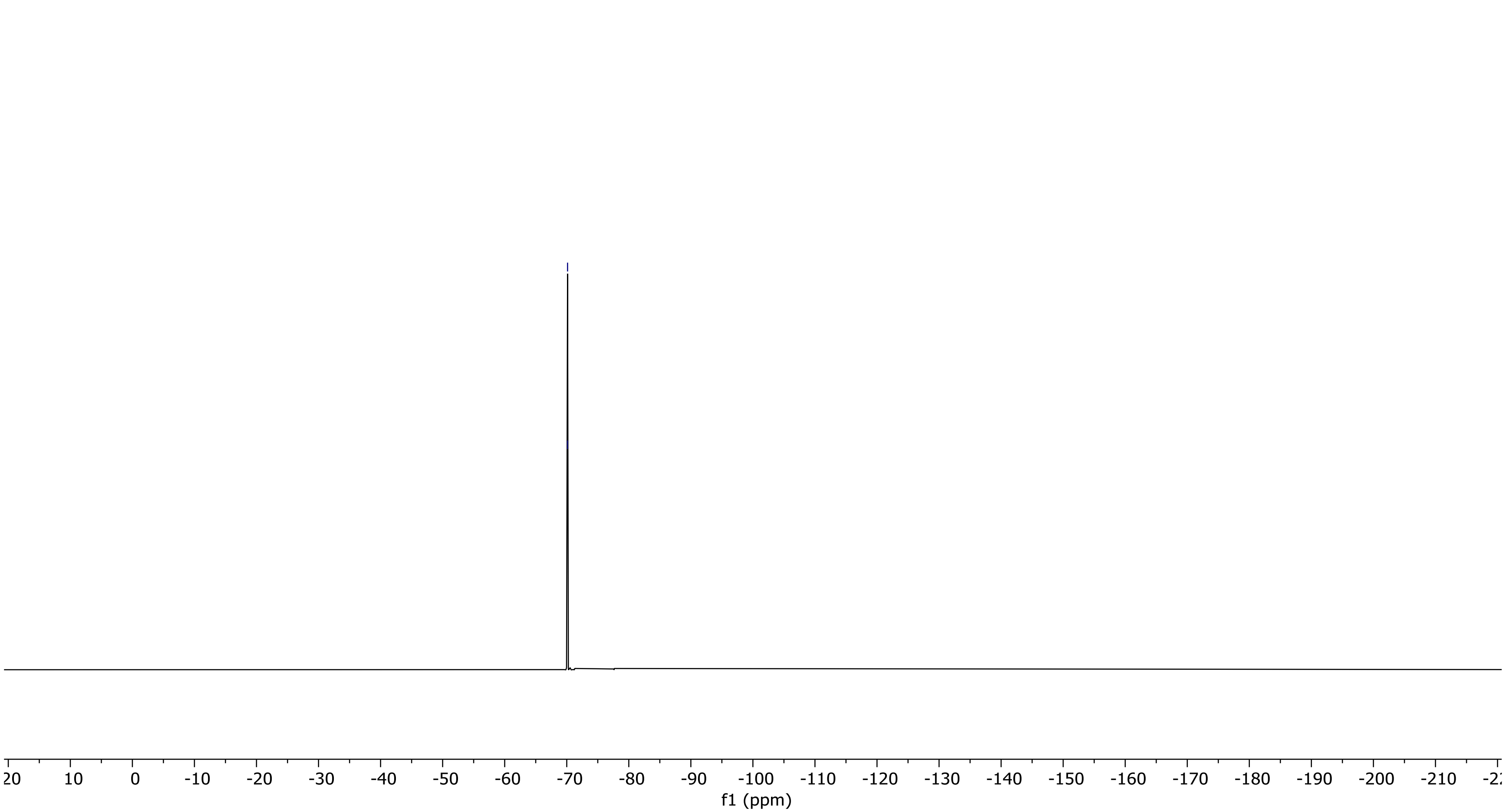

^{1}H NMR of bicyclo[1.1.1]pentylether 18

$CDCl_3$, 298 K

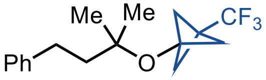

18

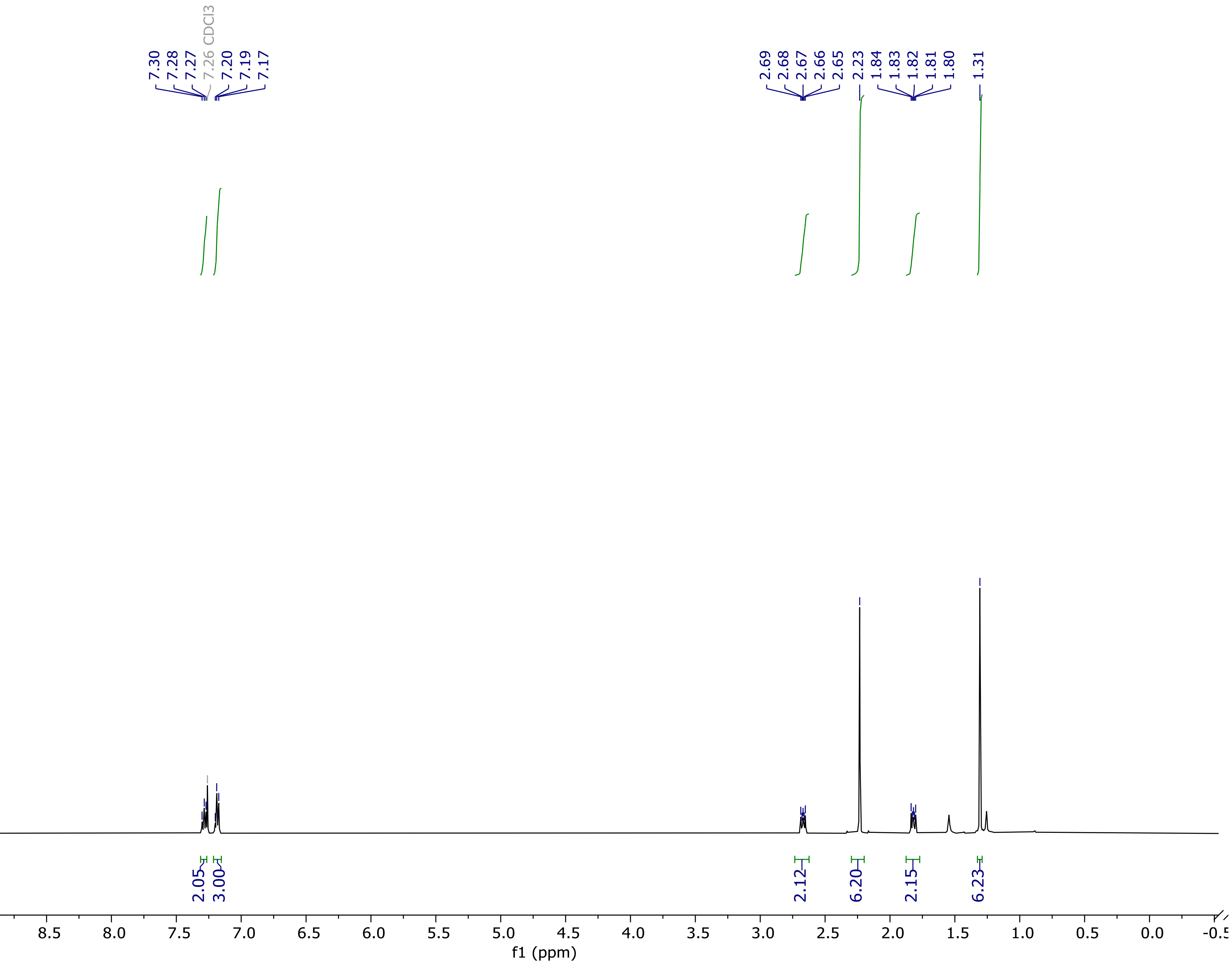

^{13}C NMR of bicyclo[1.1.1]pentylether 18

CDCl₃, 298 K

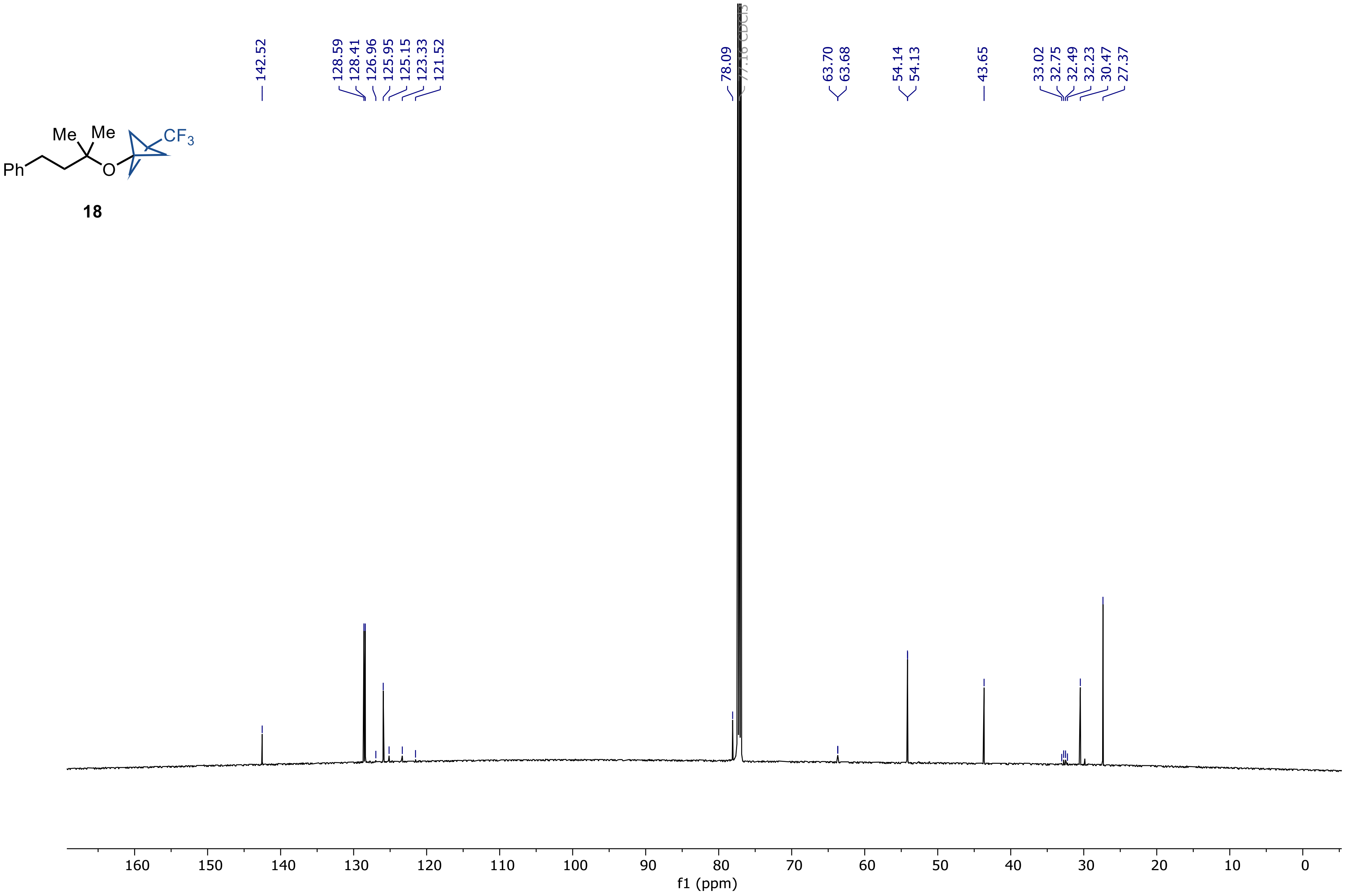

^{19}F NMR of bicyclo[1.1.1]pentylether 18

CDCl$_3$, 298 K

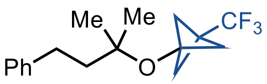

18

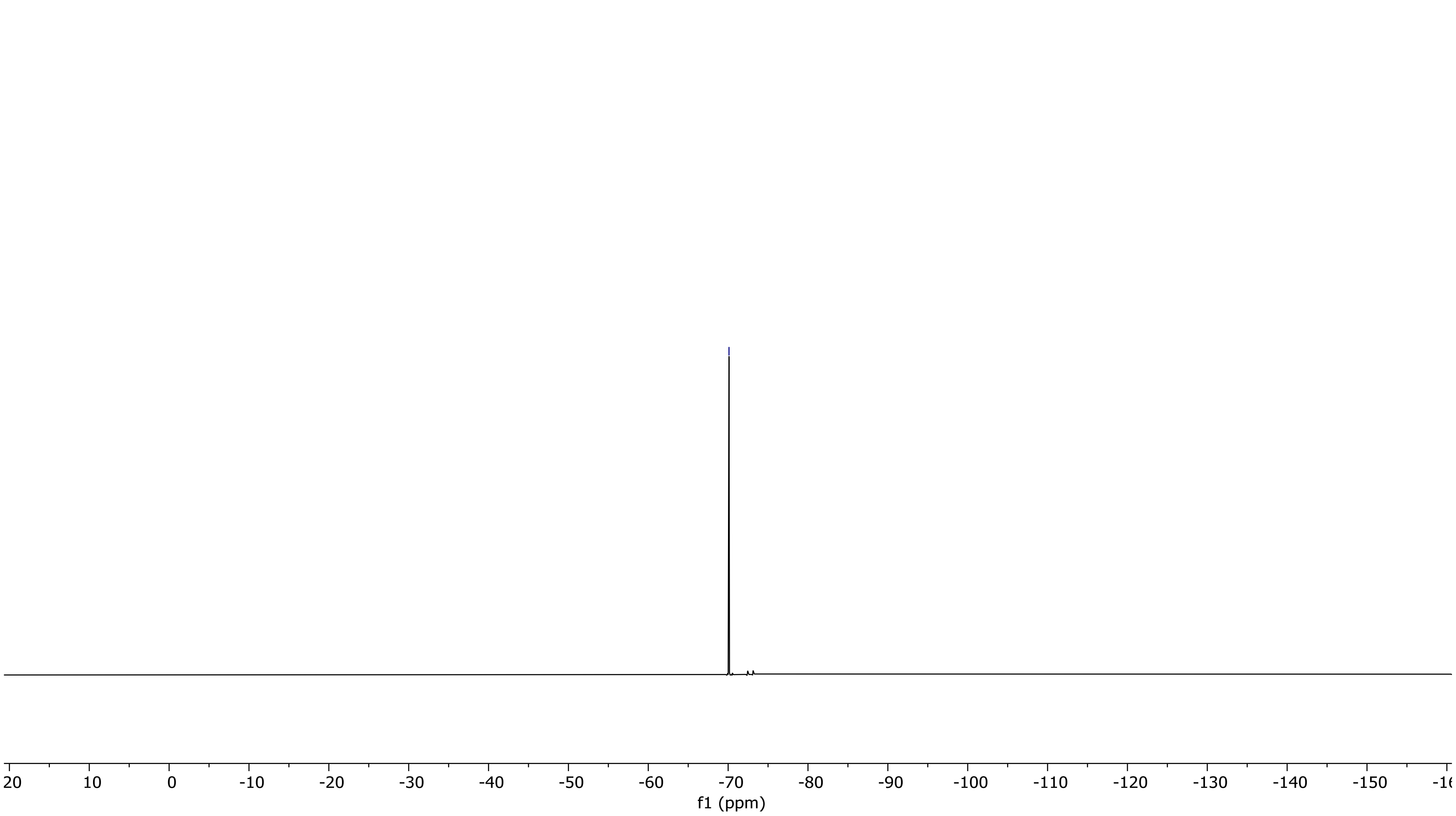

^{1}H NMR of bicyclo[1.1.1]pentylether 19

$CDCl_3$, 298 K

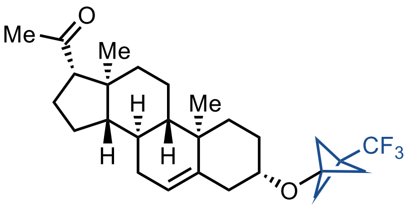

19

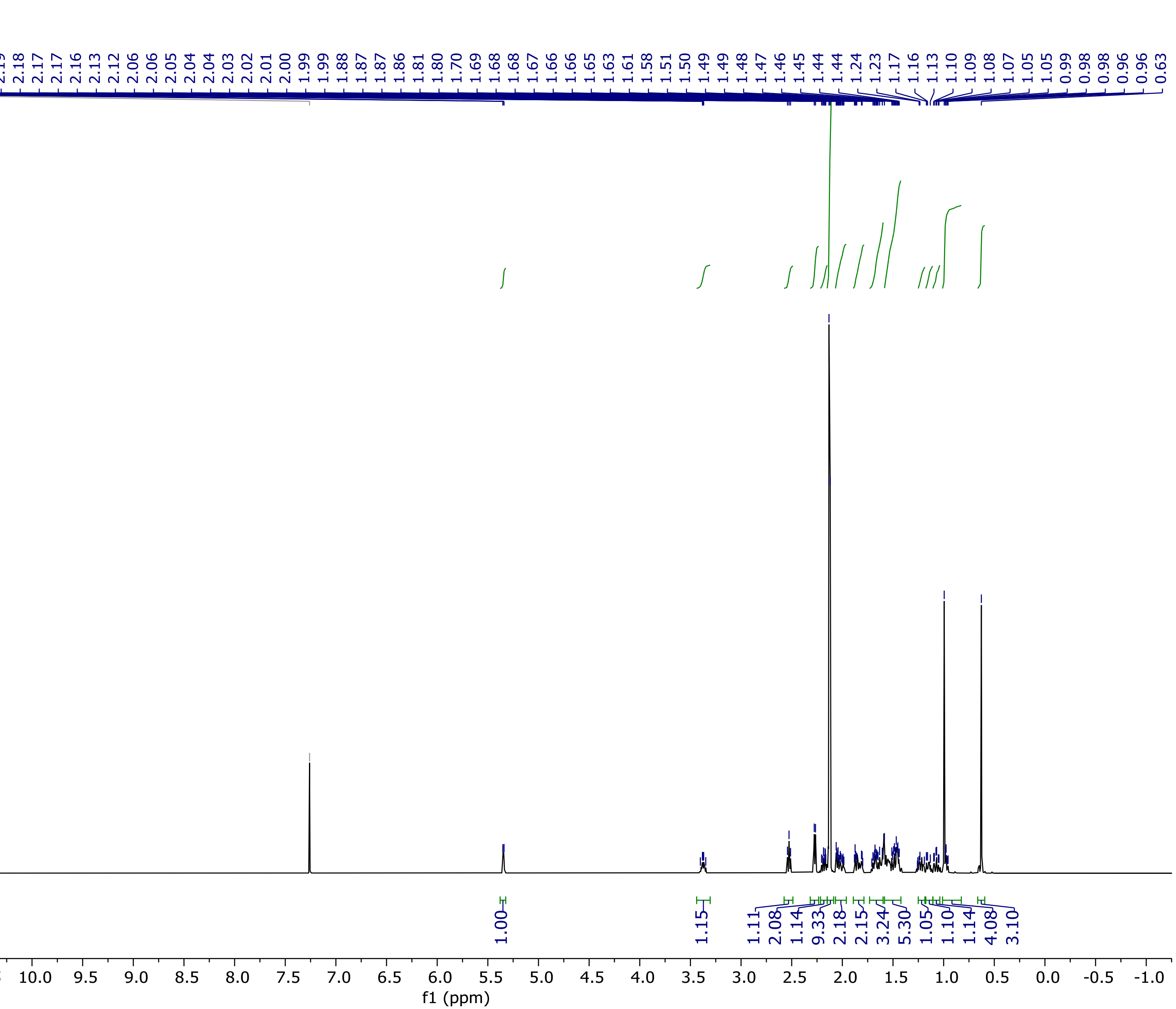

^{13}C NMR of bicyclo[1.1.1]pentylether 19

CDCl$_3$, 298 K

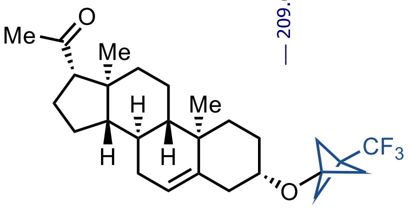

19

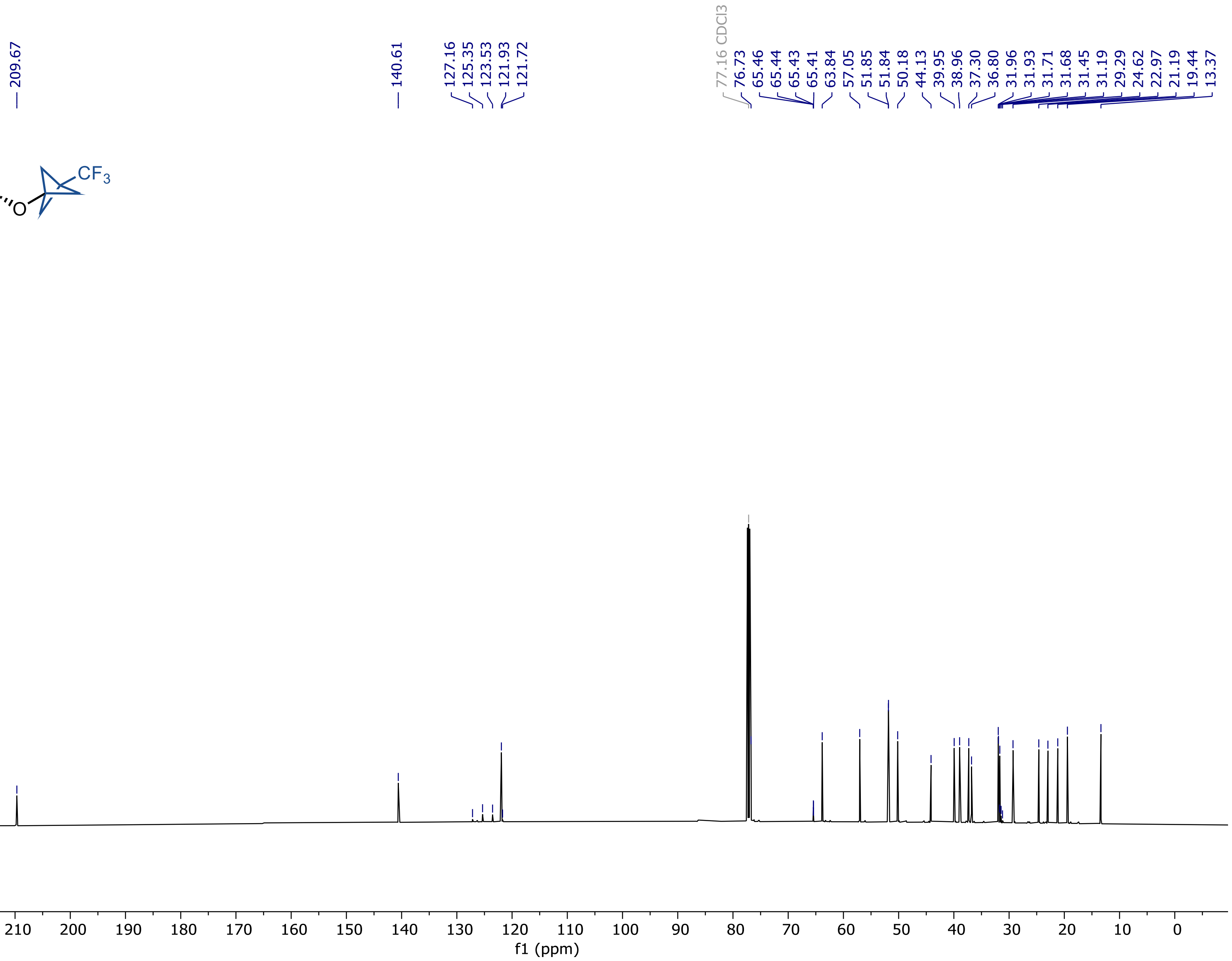

^{19}F NMR of bicyclo[1.1.1]pentylether 19

$CDCl_3$, 298 K

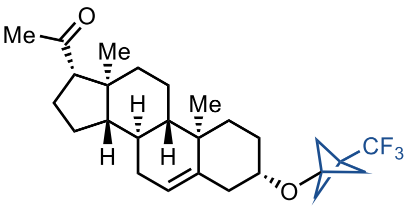

19

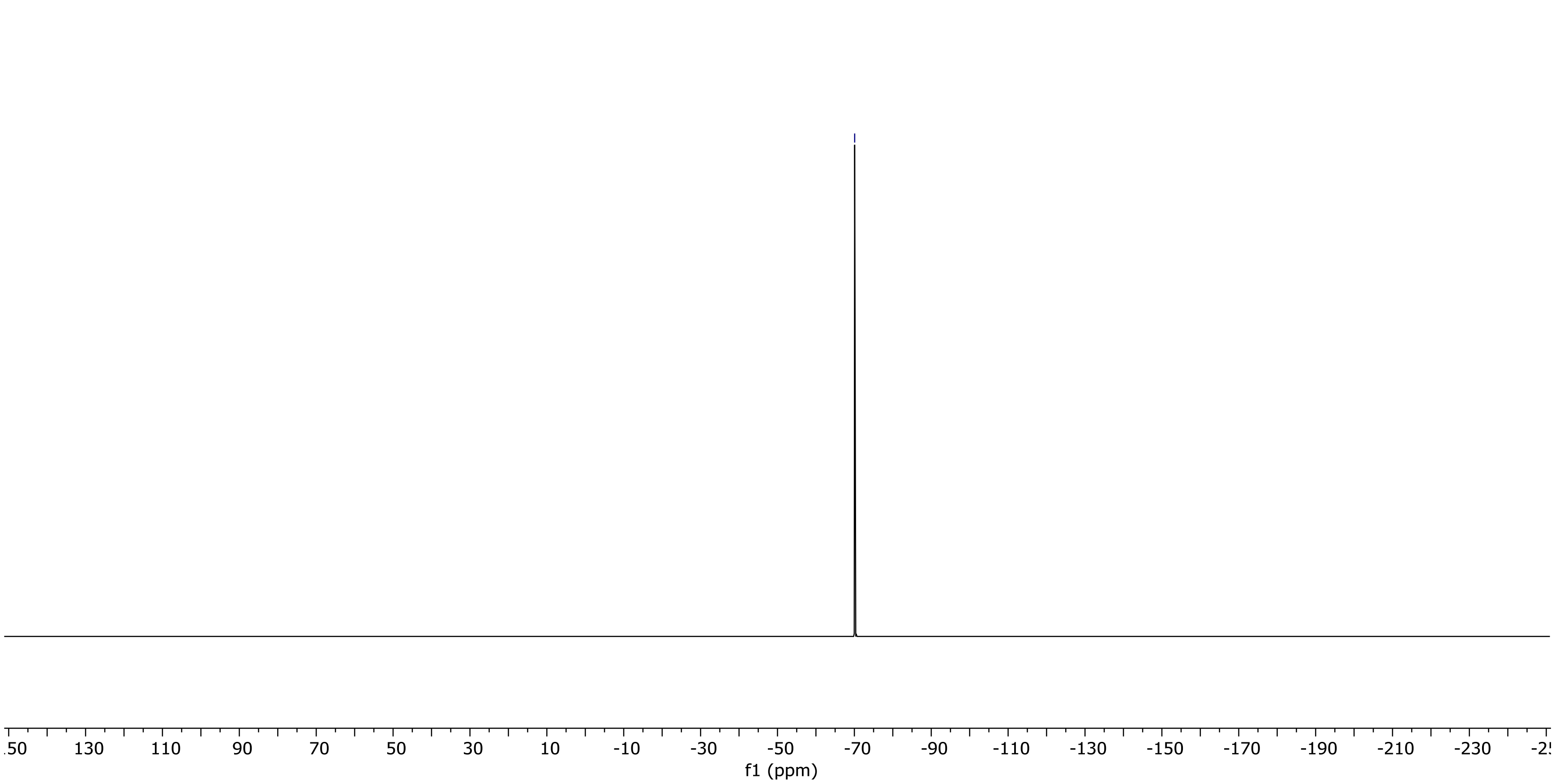

^{1}H NMR of bicyclo[1.1.1]pentylether 20

CDCl$_3$, 298 K

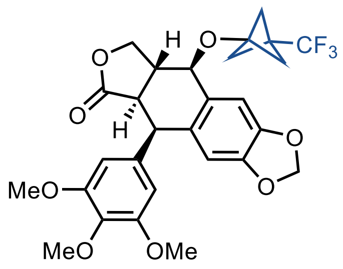

20

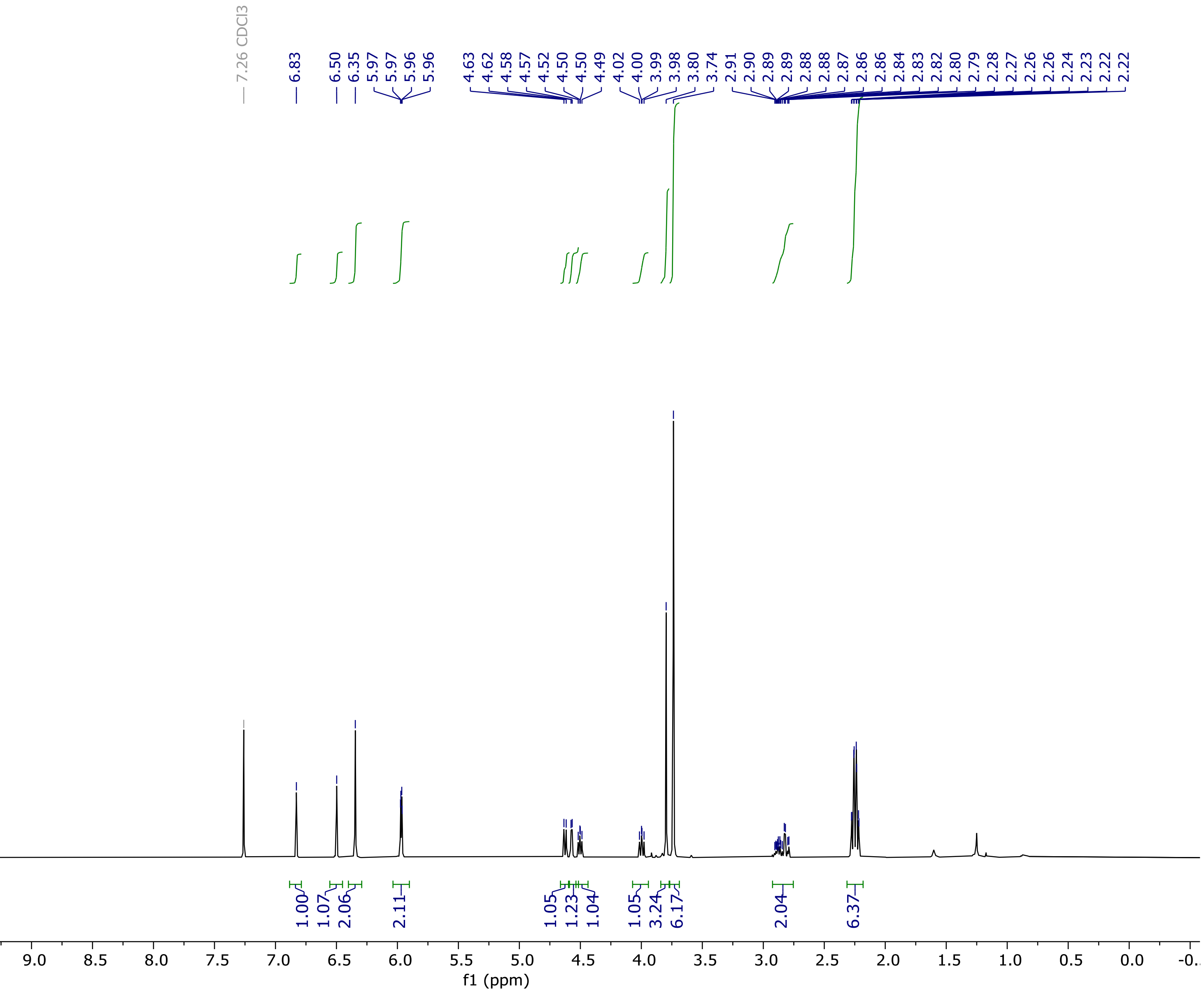

^{13}C NMR of bicyclo[1.1.1]pentylether 20

CDCl$_3$, 298 K

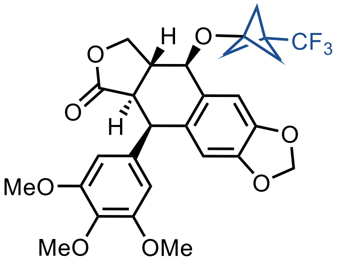

20

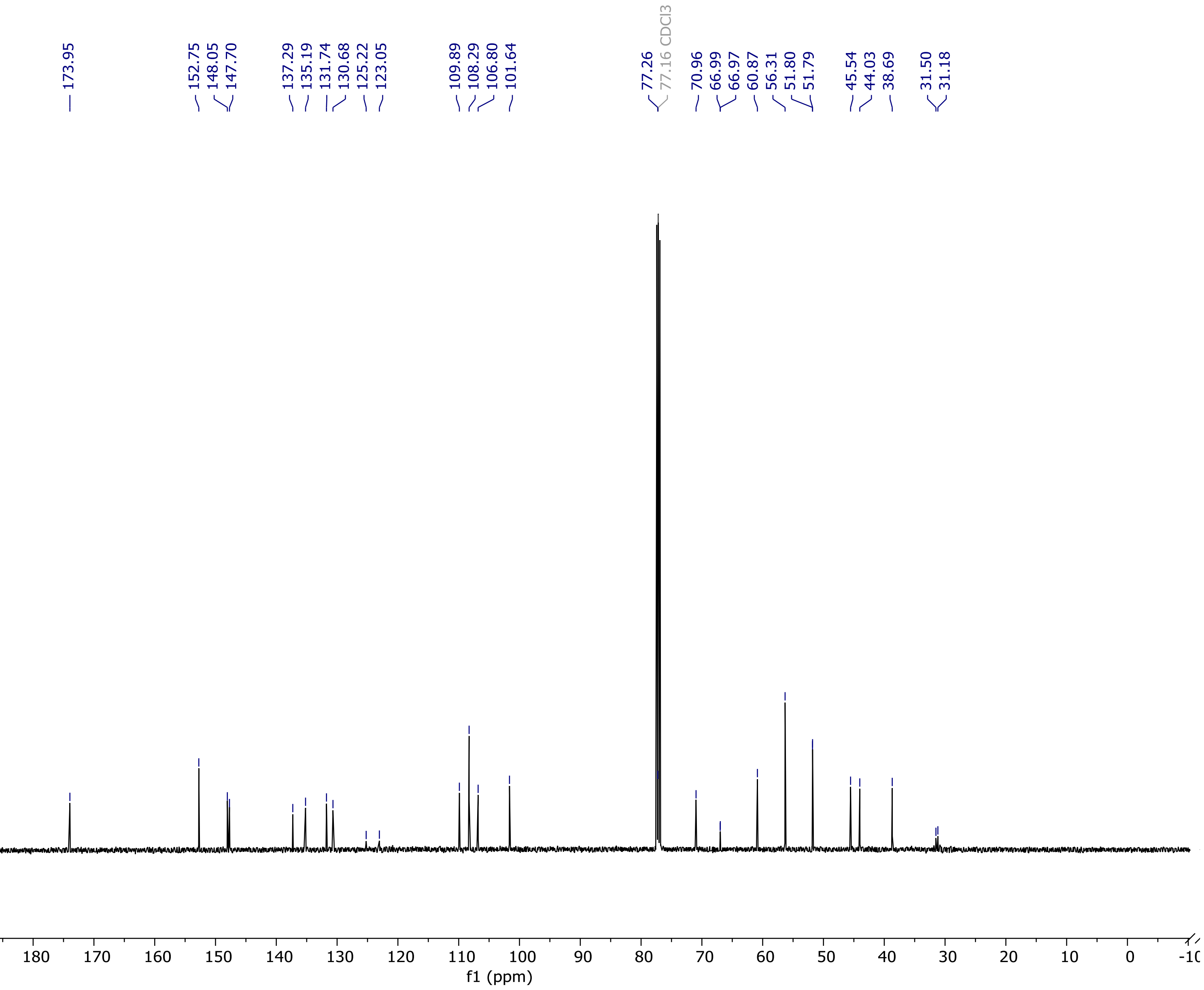

^{19}F NMR of bicyclo[1.1.1]pentylether 20

$CDCl_3$, 298 K

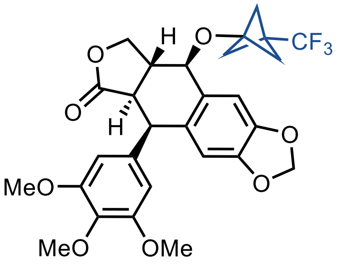

20

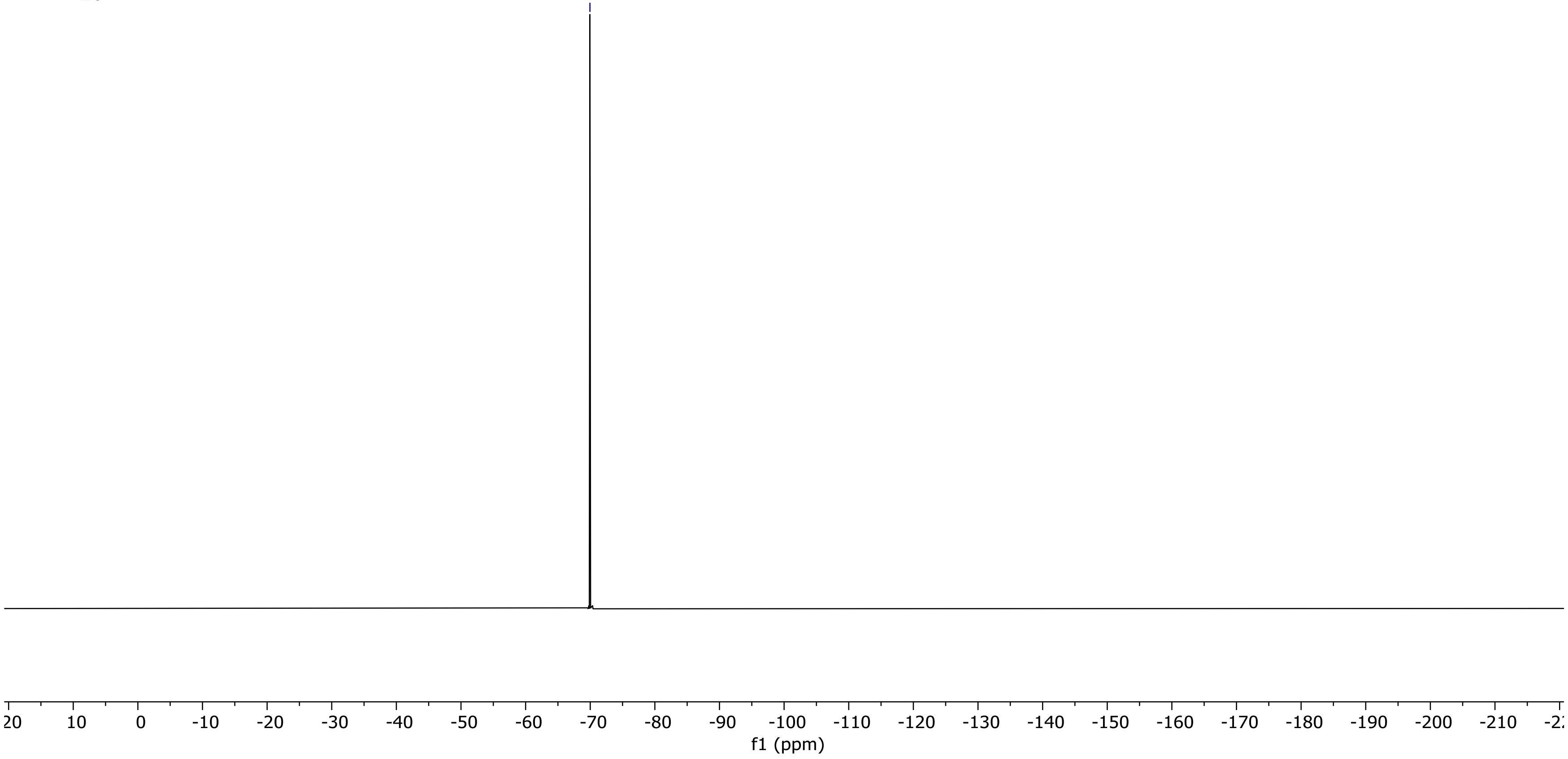

^{1}H NMR of bicyclo[1.1.1]pentylether 21

$CDCl_3$, 298 K

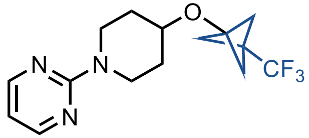

21

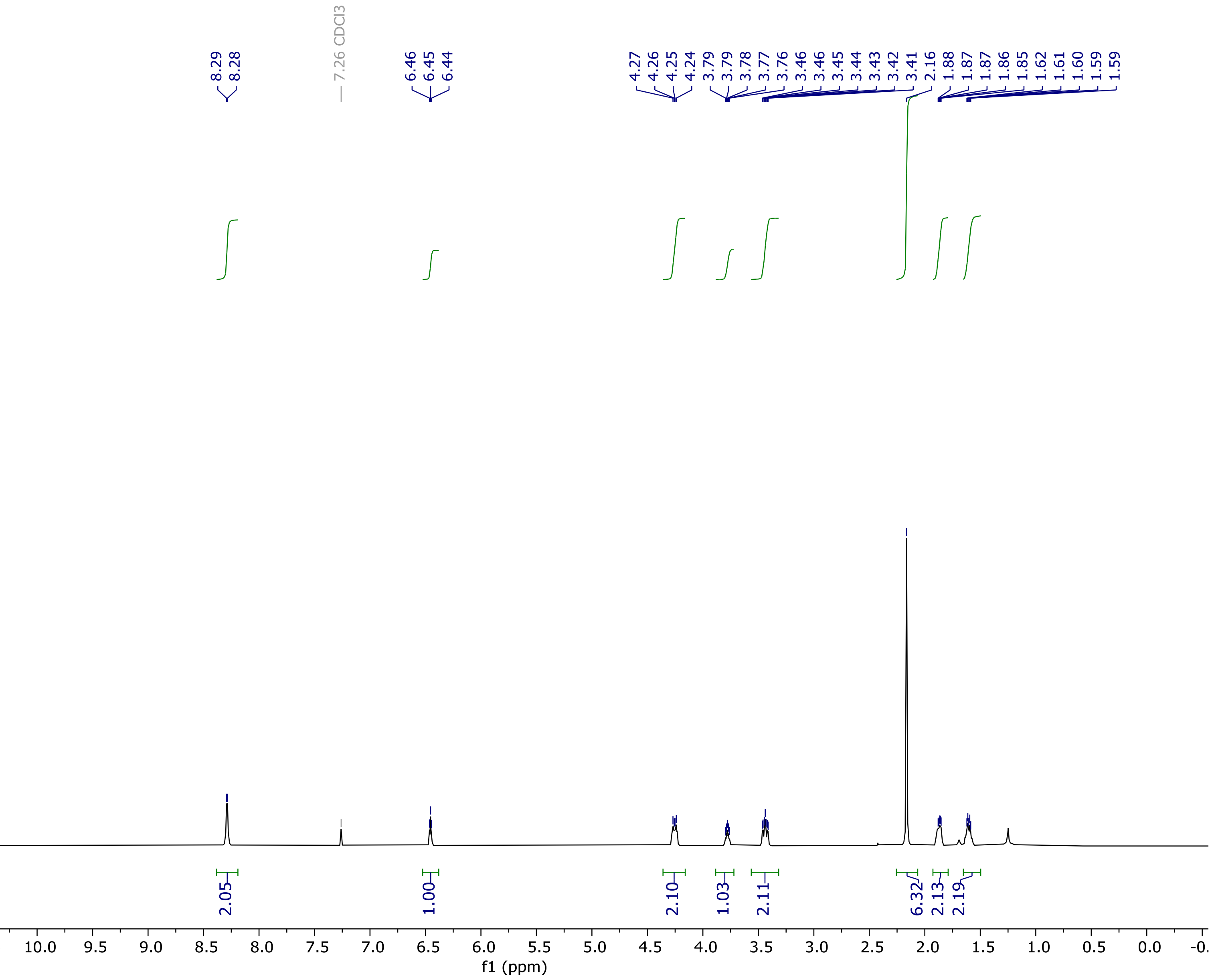

^{13}C NMR of bicyclo[1.1.1]pentylether 21

$CDCl_3$, 298 K

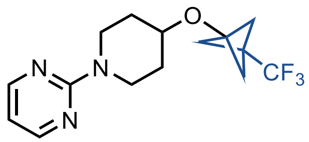

21

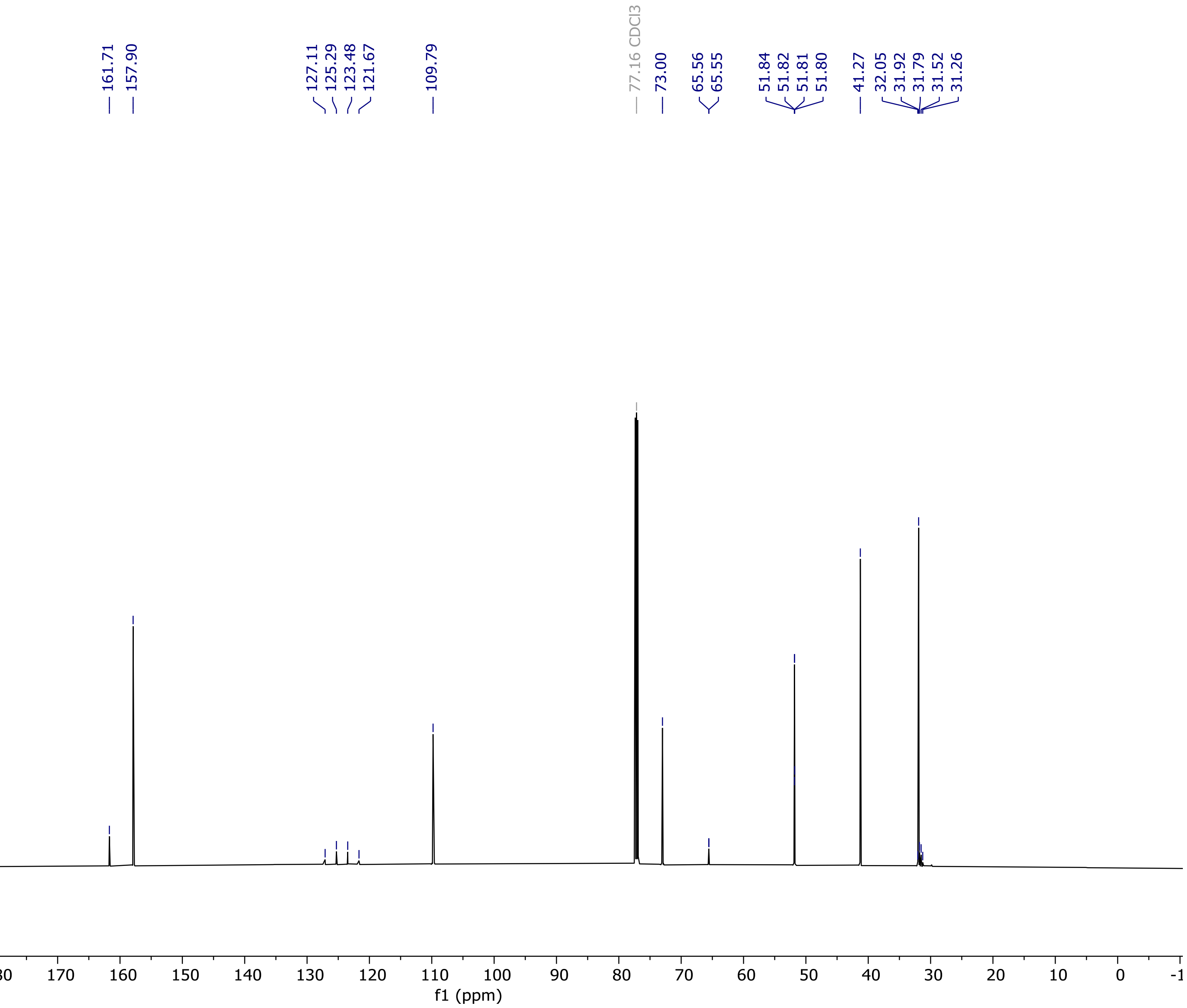

^{19}F NMR of bicyclo[1.1.1]pentylether 21

$CDCl_3$, 298 K

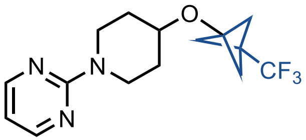

21

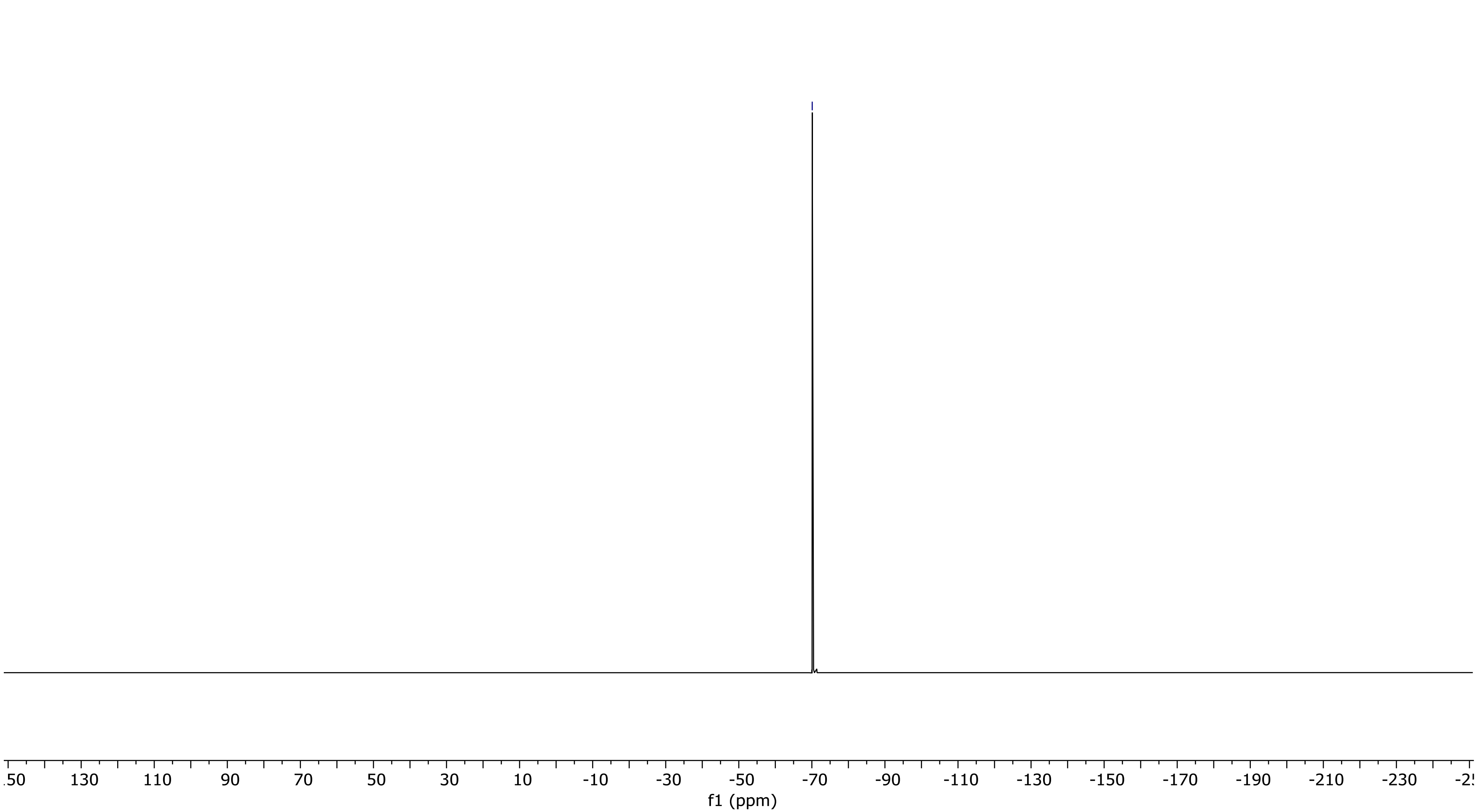

^{1}H NMR of bicyclo[1.1.1]pentylether 22

CDCl$_3$, 298 K

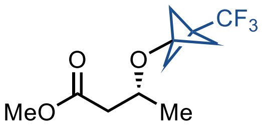

22

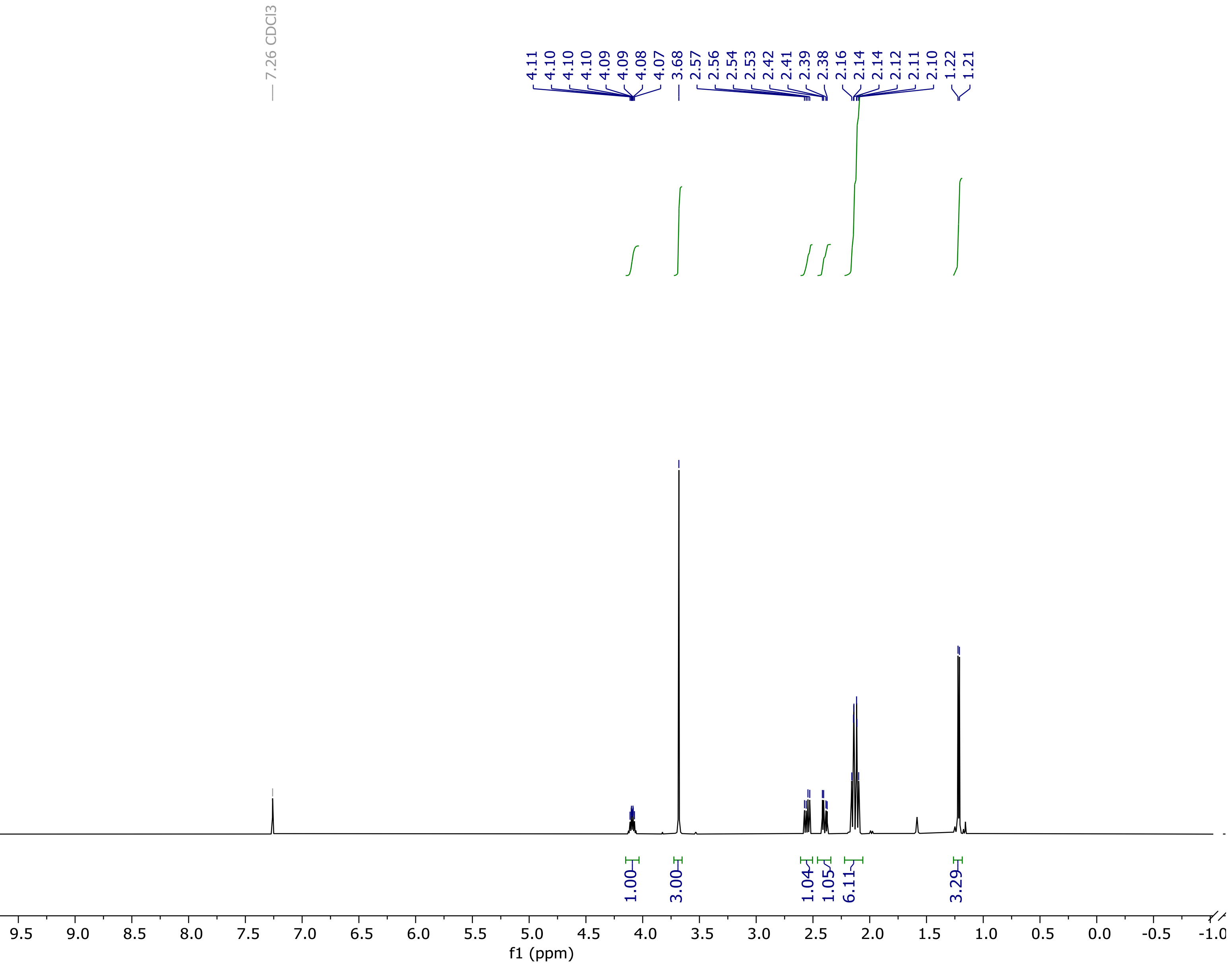

^{13}C NMR of bicyclo[1.1.1]pentylether 22

CDCl$_3$, 298 K

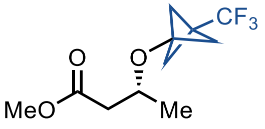

22

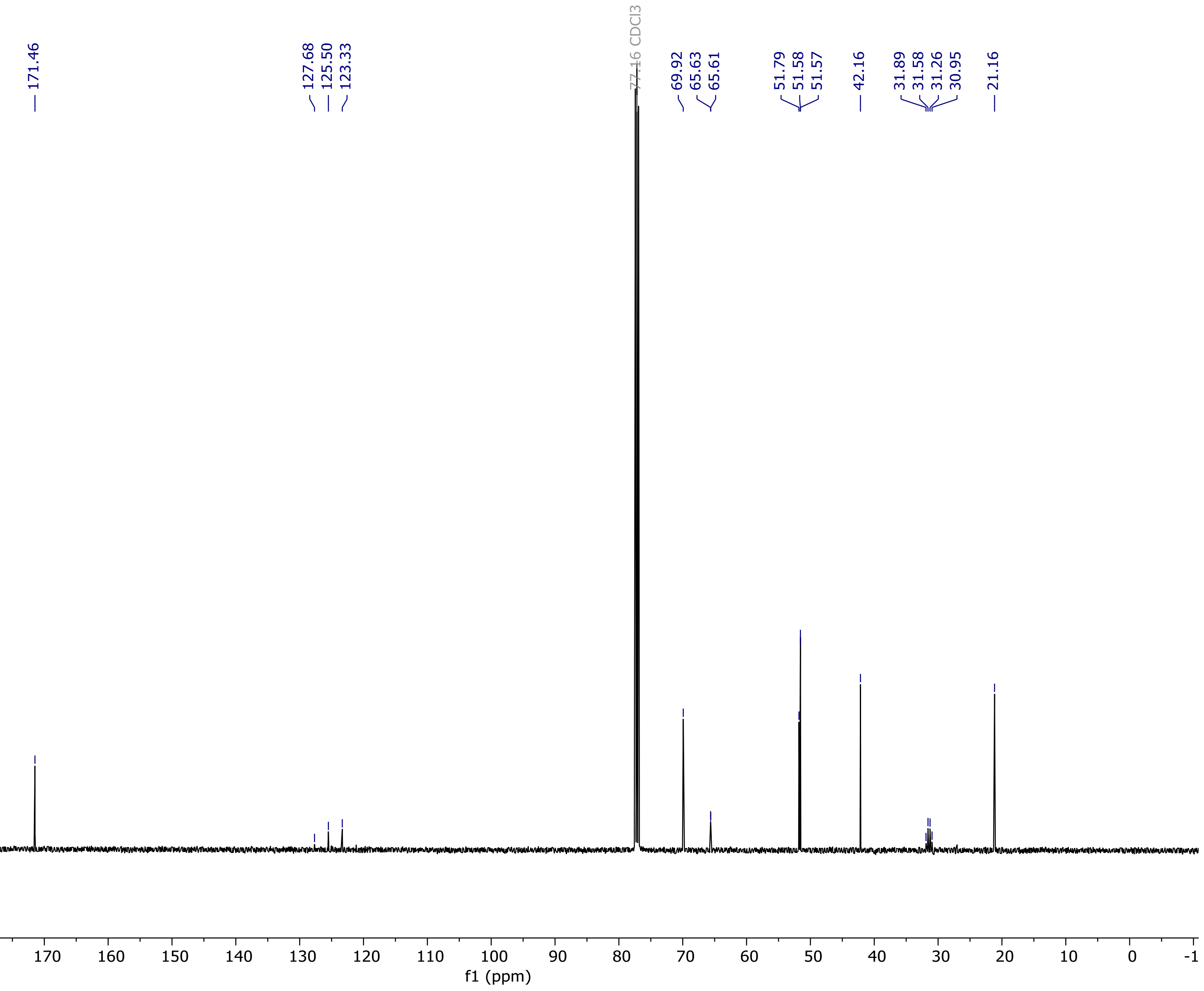

^{19}F NMR of bicyclo[1.1.1]pentylether 22

CDCl$_3$, 298 K

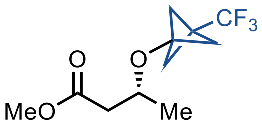

22

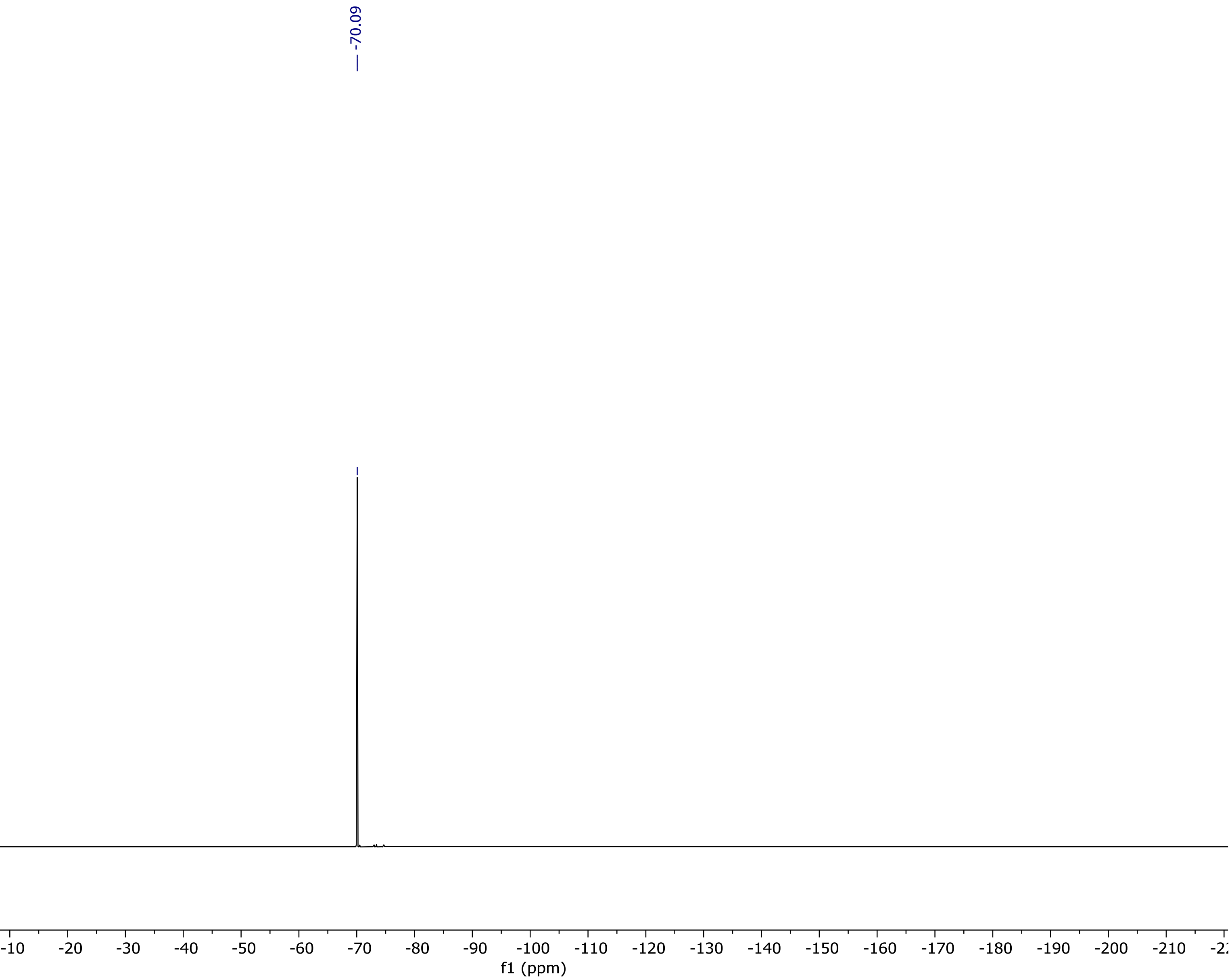

^{1}H NMR of bicyclo[1.1.1]pentylether 23

$CDCl_3$, 298 K

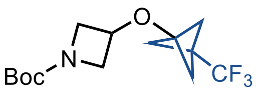

23

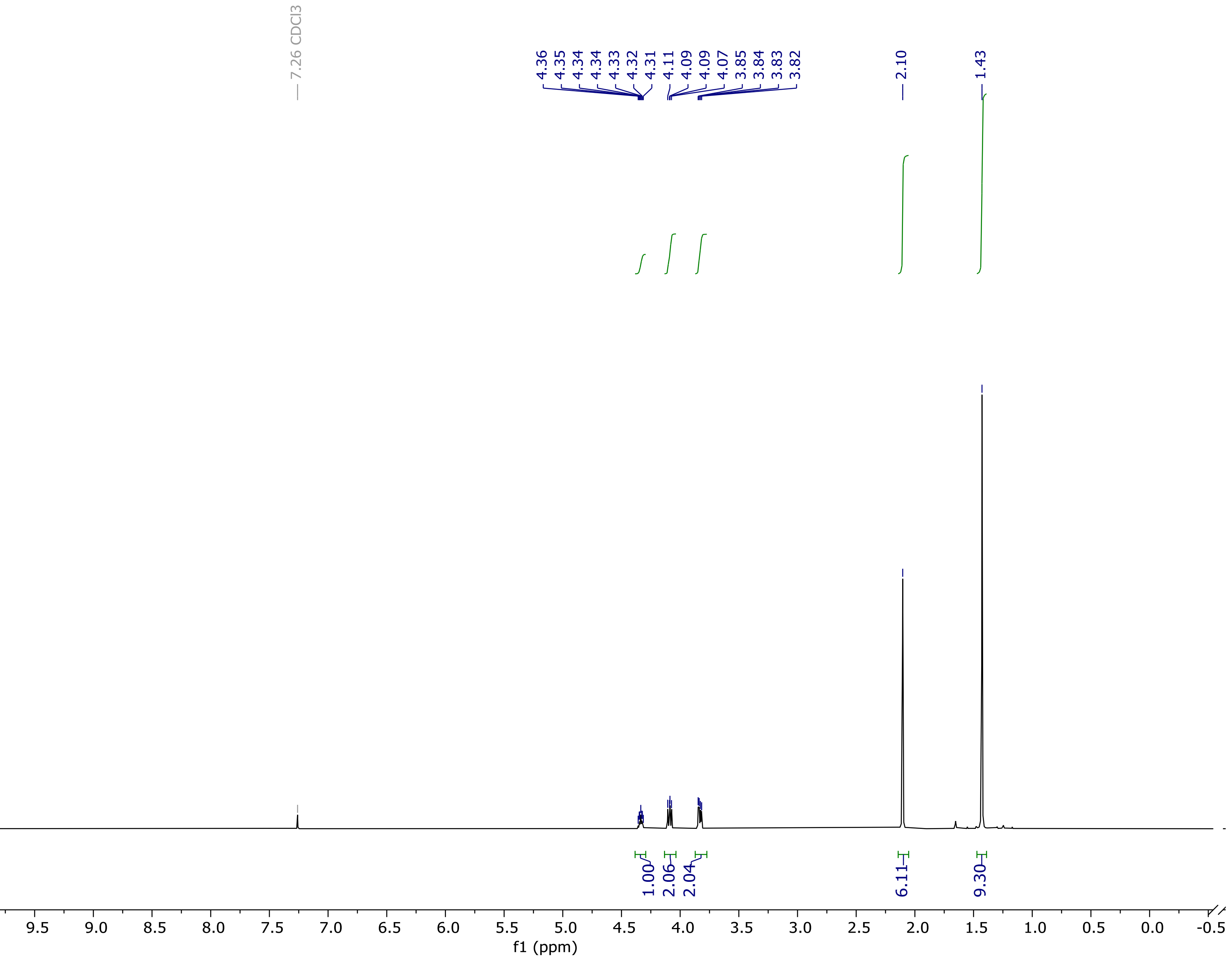

^{13}C NMR of bicyclo[1.1.1]pentylether 23

$CDCl_3$, 298 K

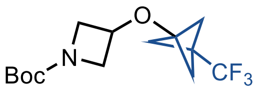

23

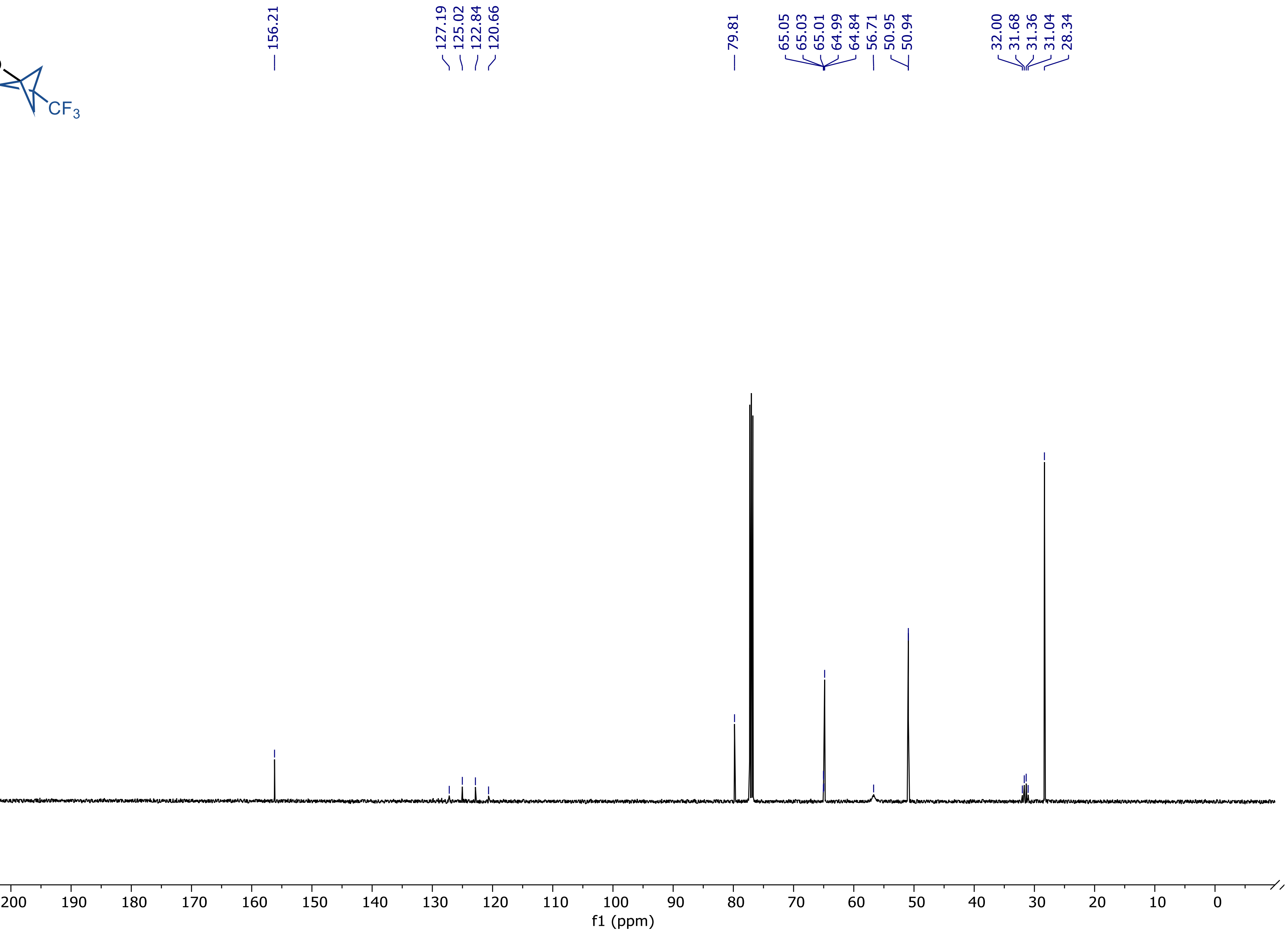

^{19}F NMR of bicyclo[1.1.1]pentylether 23

$CDCl_3$, 298 K

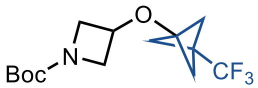

23

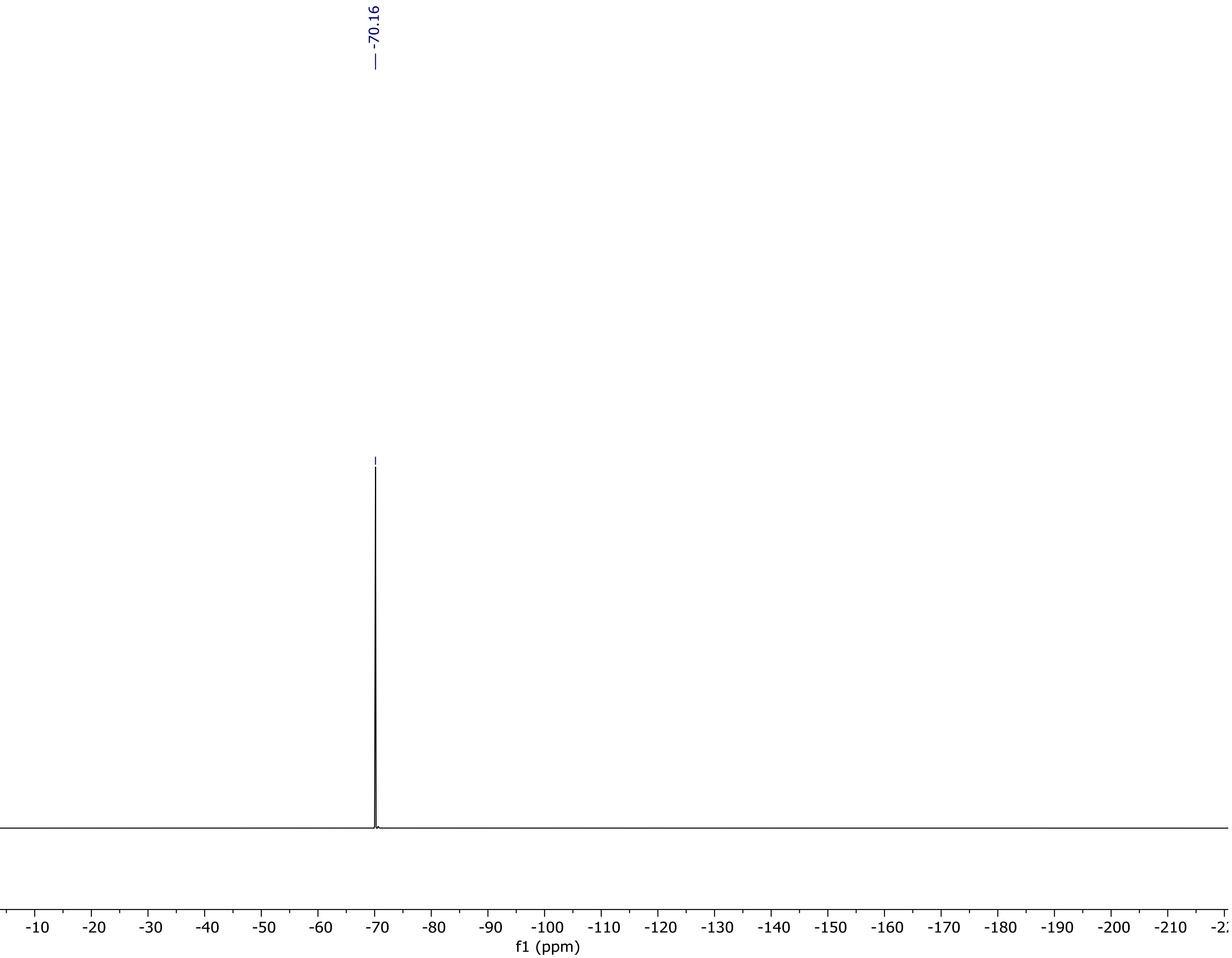

^{1}H NMR of bicyclo[1.1.1]pentylether 24

$CDCl_3$, 298 K

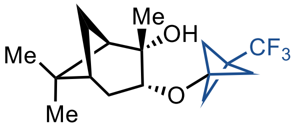

24

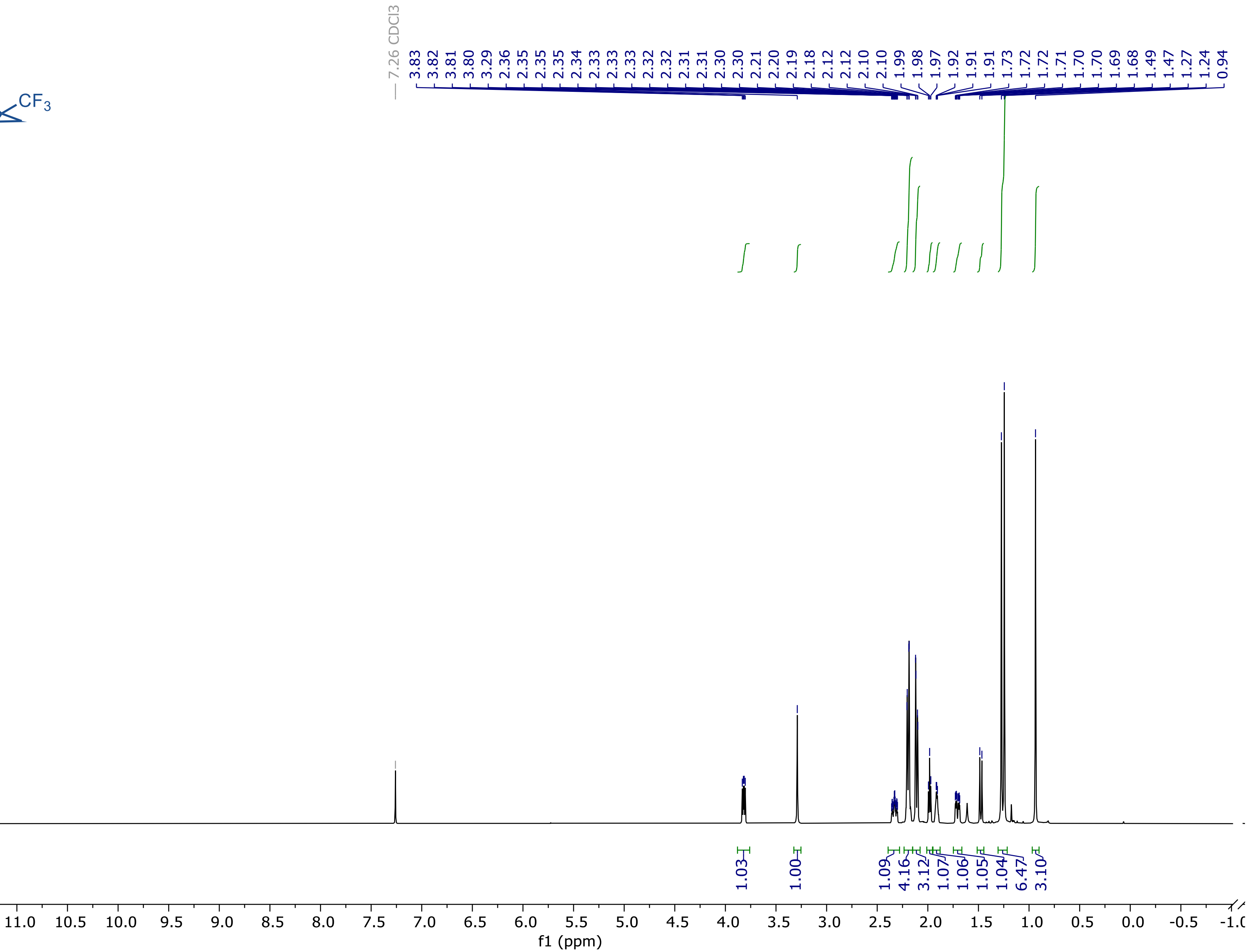

^{13}C NMR of bicyclo[1.1.1]pentylether 24

CDCl₃, 298 K

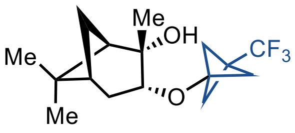

24

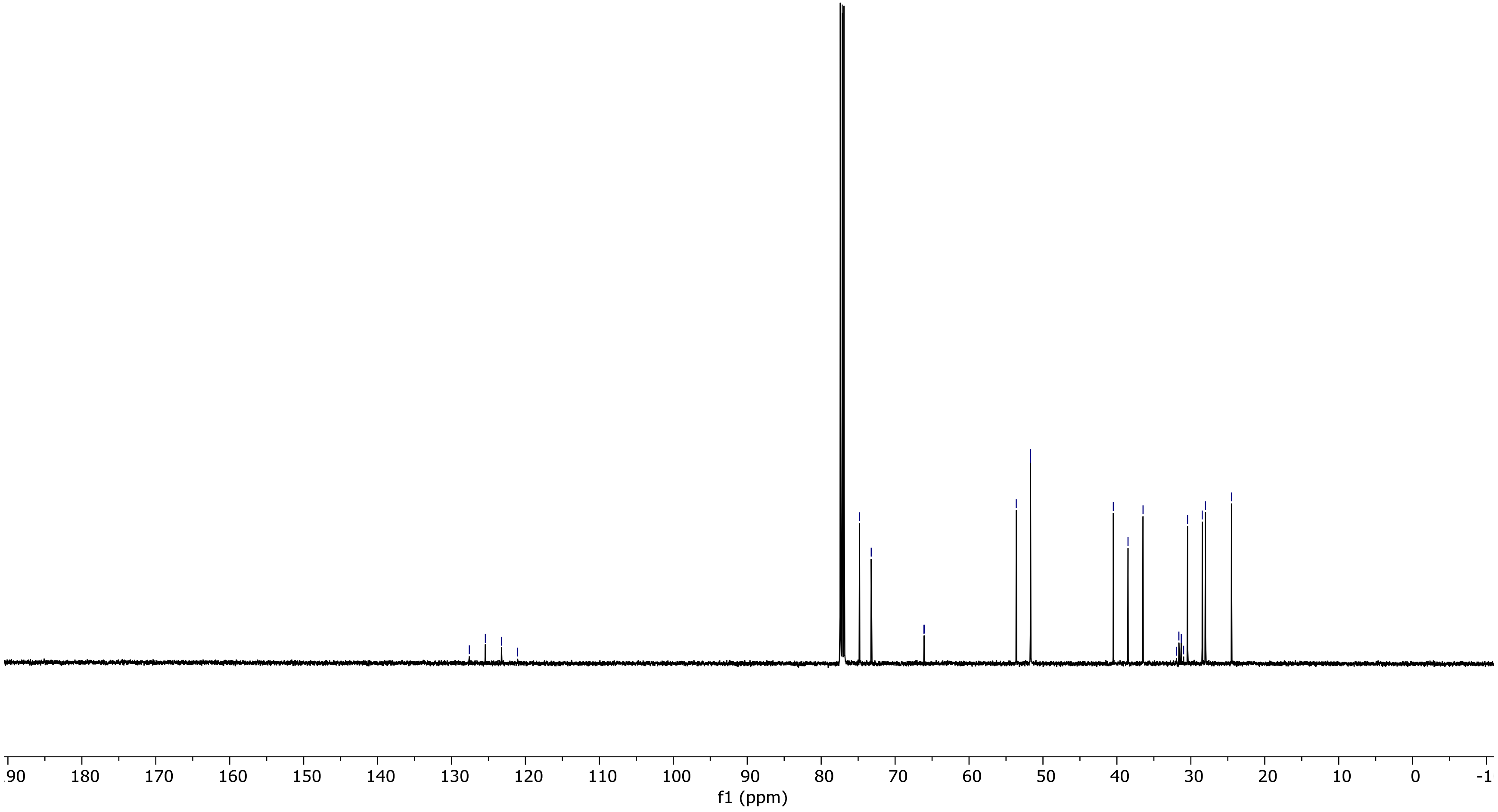

^{19}F NMR of bicyclo[1.1.1]pentylether 24

$CDCl_3$, 298 K

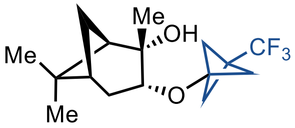

24

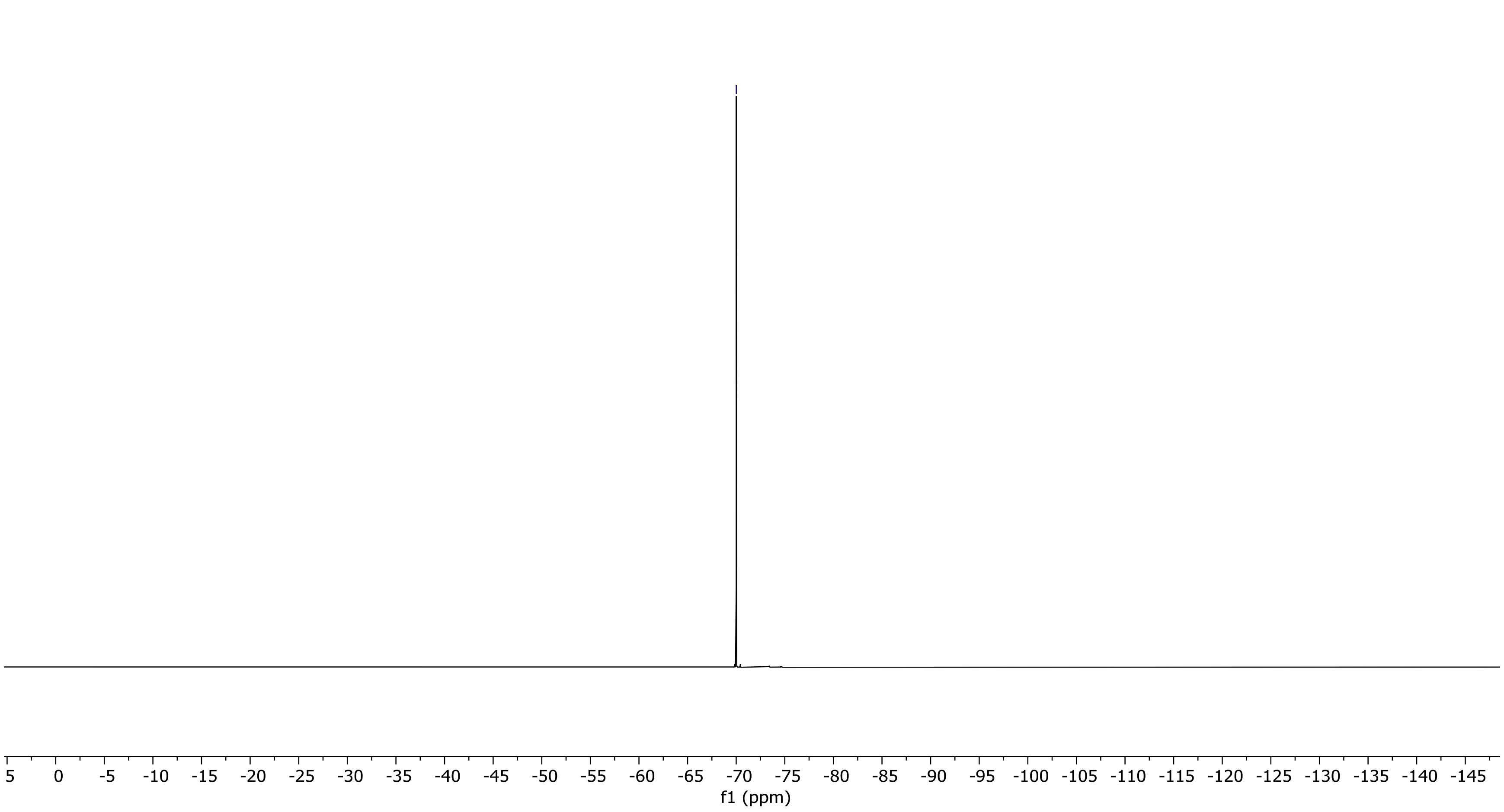

^{1}H NMR of bicyclo[1.1.1]pentylether 25

$CDCl_3$, 298 K

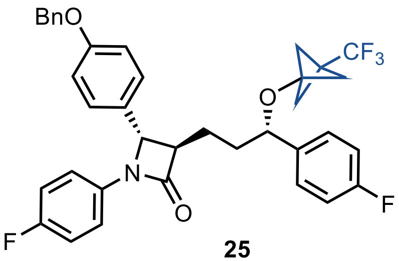

25

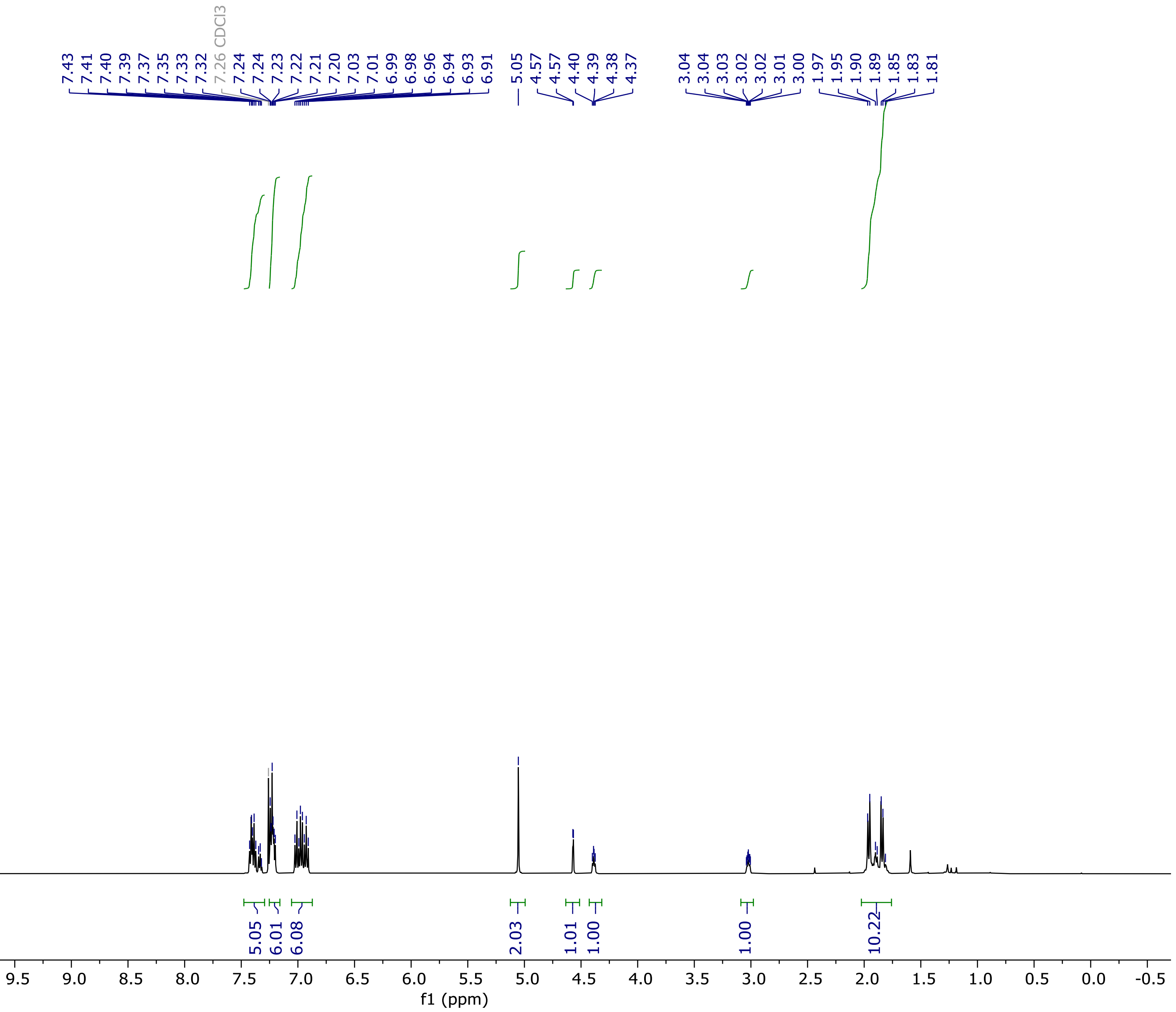

^{13}C NMR of bicyclo[1.1.1]pentylether 25

$CDCl_3$, 298 K

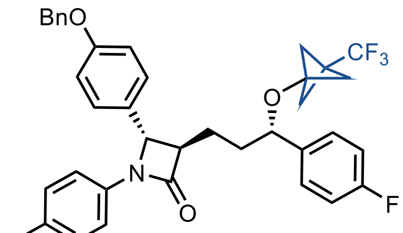

25

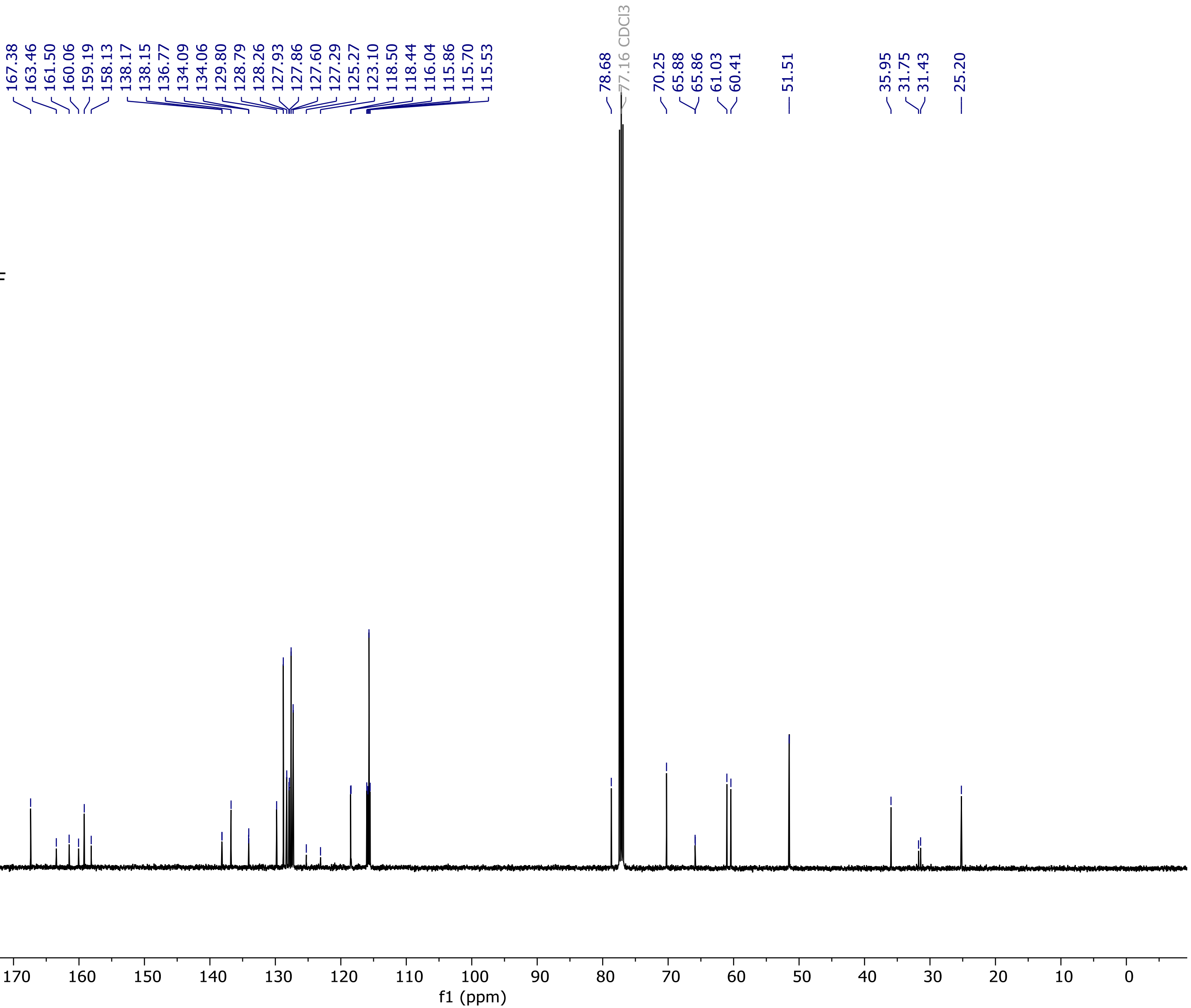

^{19}F NMR of bicyclo[1.1.1]pentylether 25

CDCl$_3$, 298 K

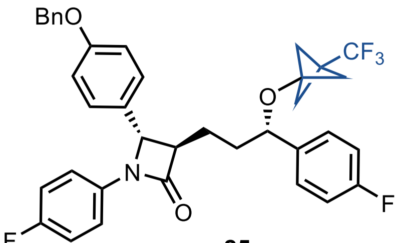

25

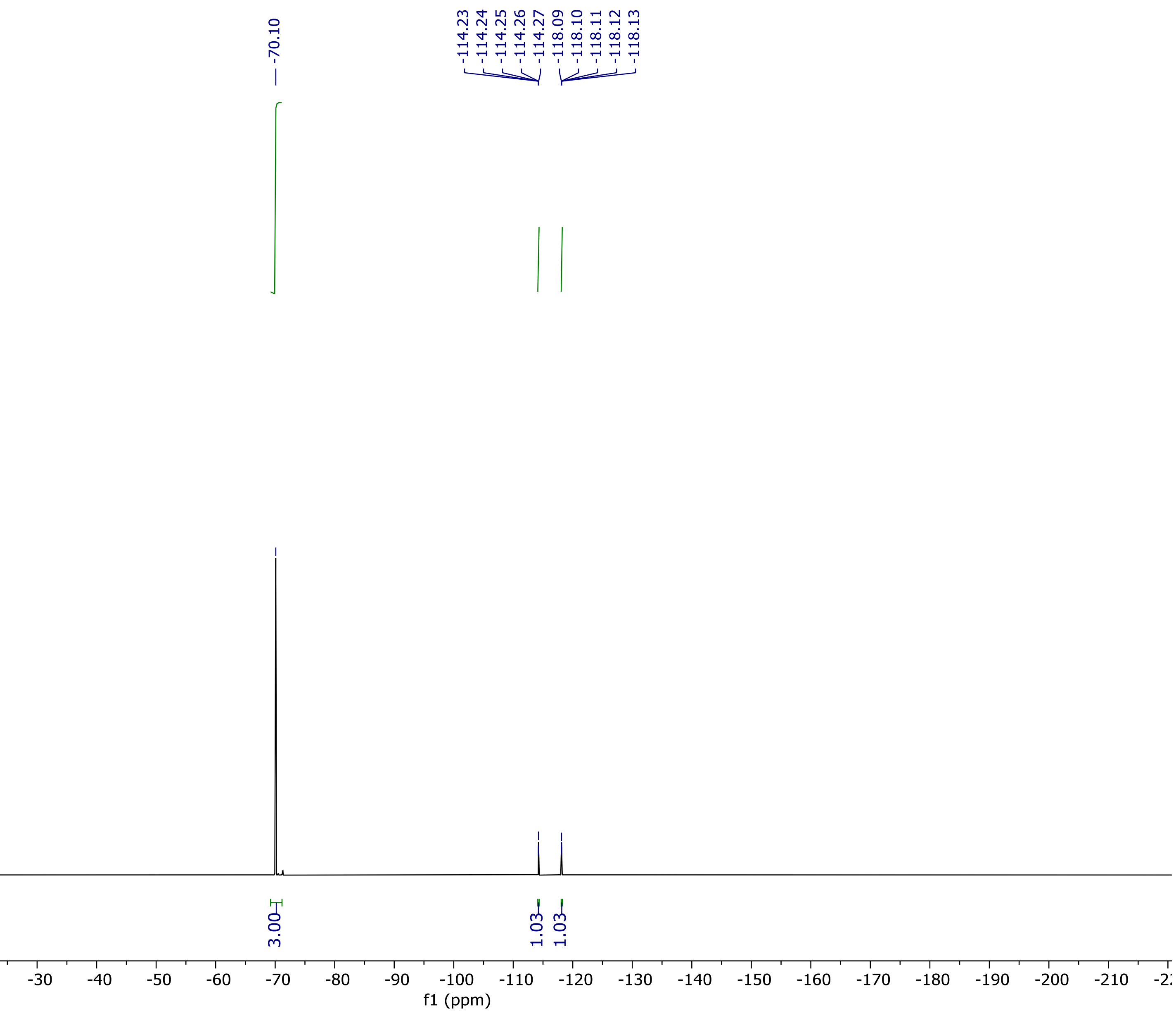

^{1}H NMR of bicyclo[1.1.1]pentylether 26

$CDCl_3$, 298 K

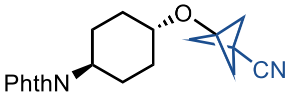

26

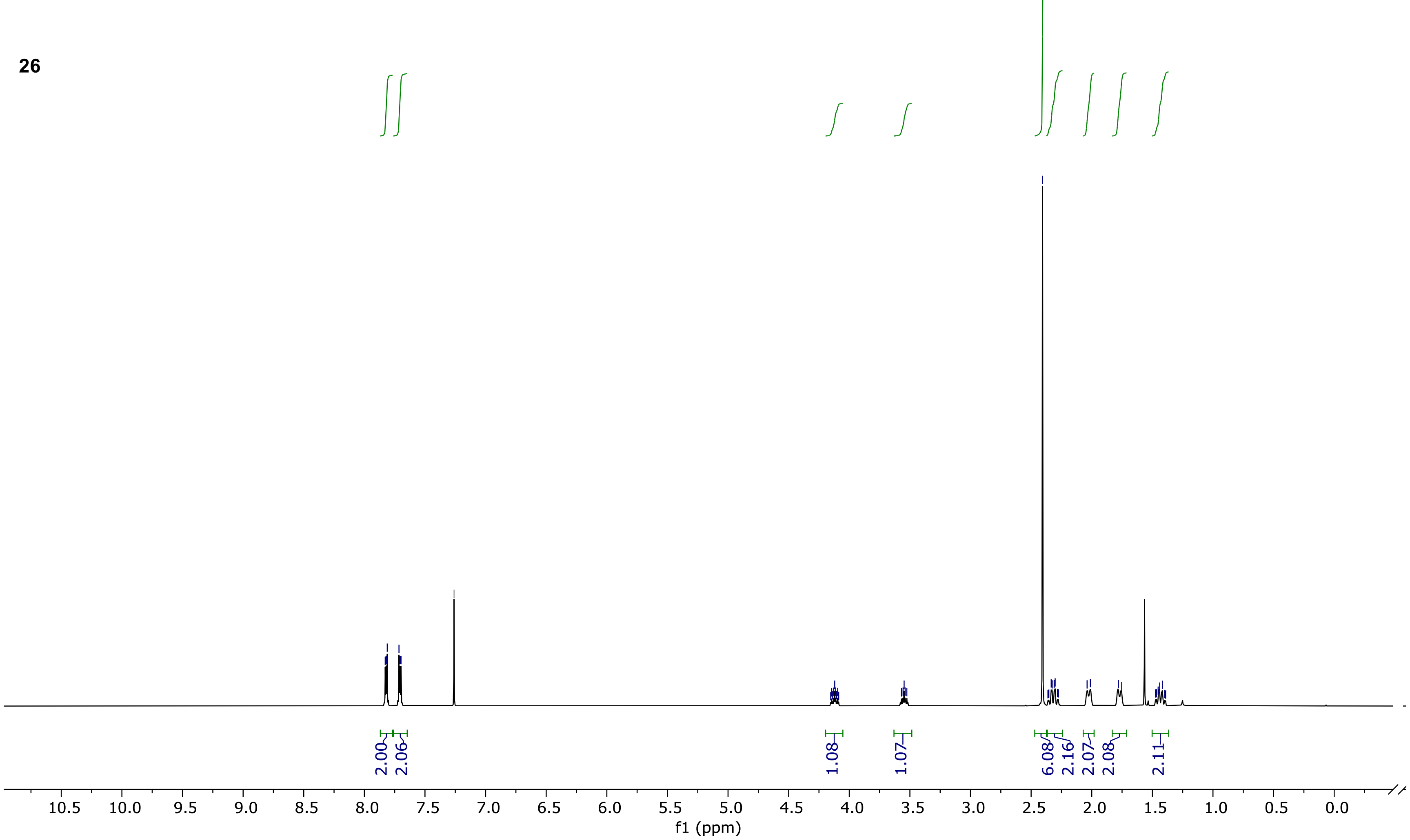

^{13}C NMR of bicyclo[1.1.1]pentylether 26

$CDCl_3$, 298 K

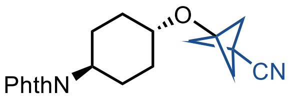

26

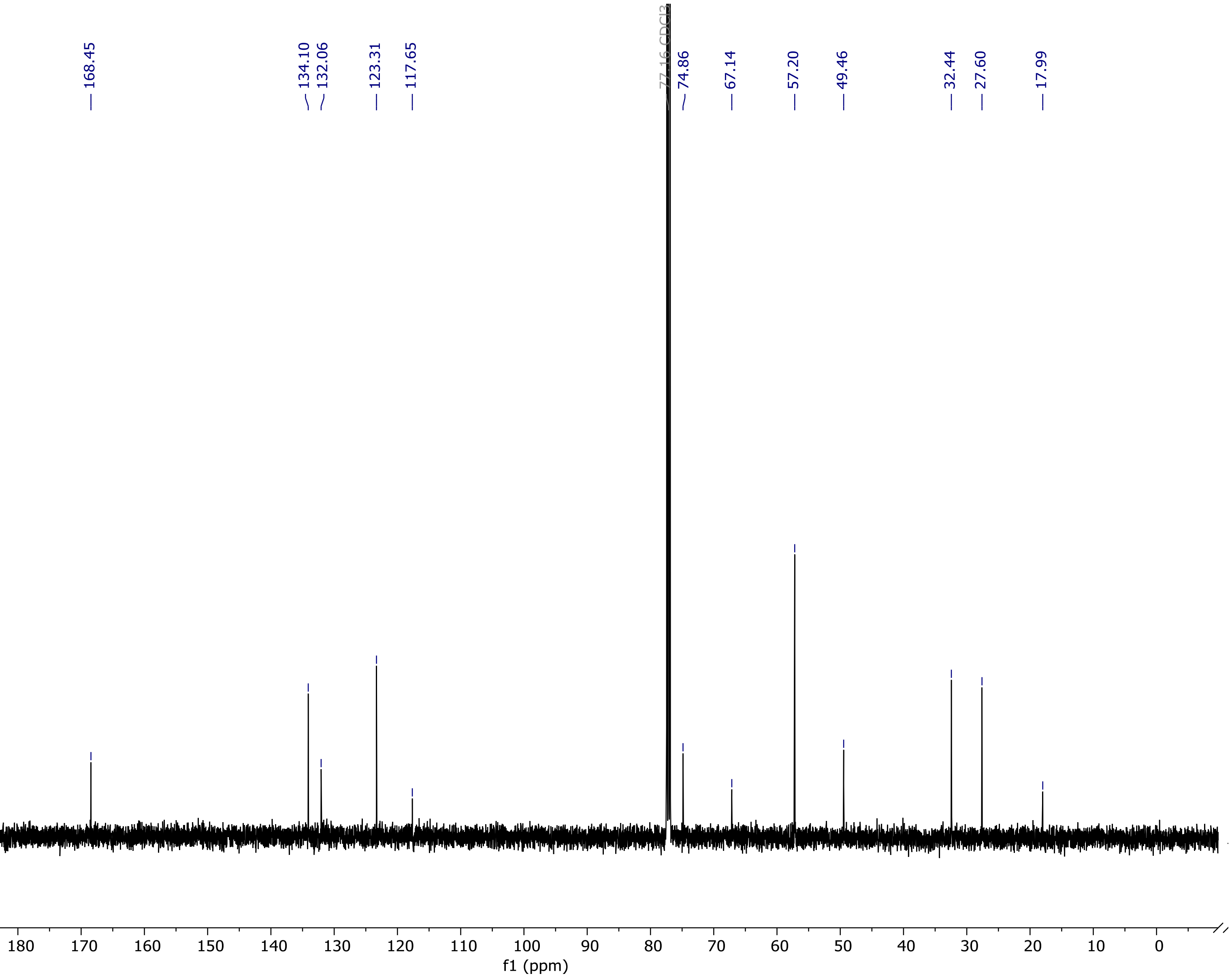

^{1}H NMR of bicyclo[1.1.1]pentylether 27

CDCl3, 298 K

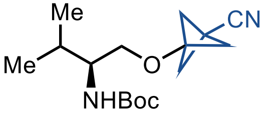

27

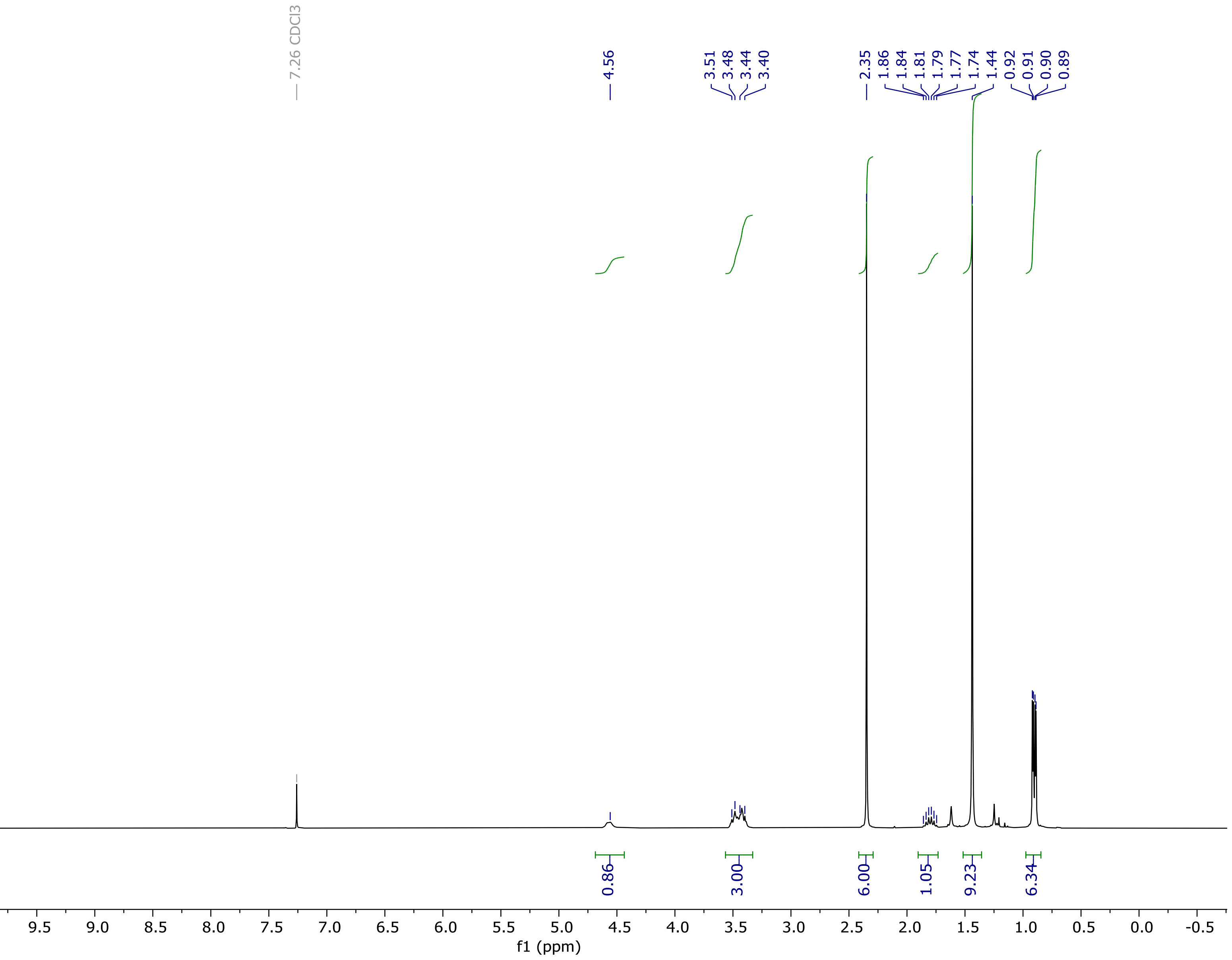

^{13}C NMR of bicyclo[1.1.1]pentylether 27

CDCl$_3$, 298 K

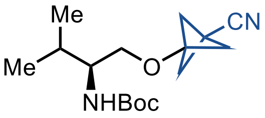

27

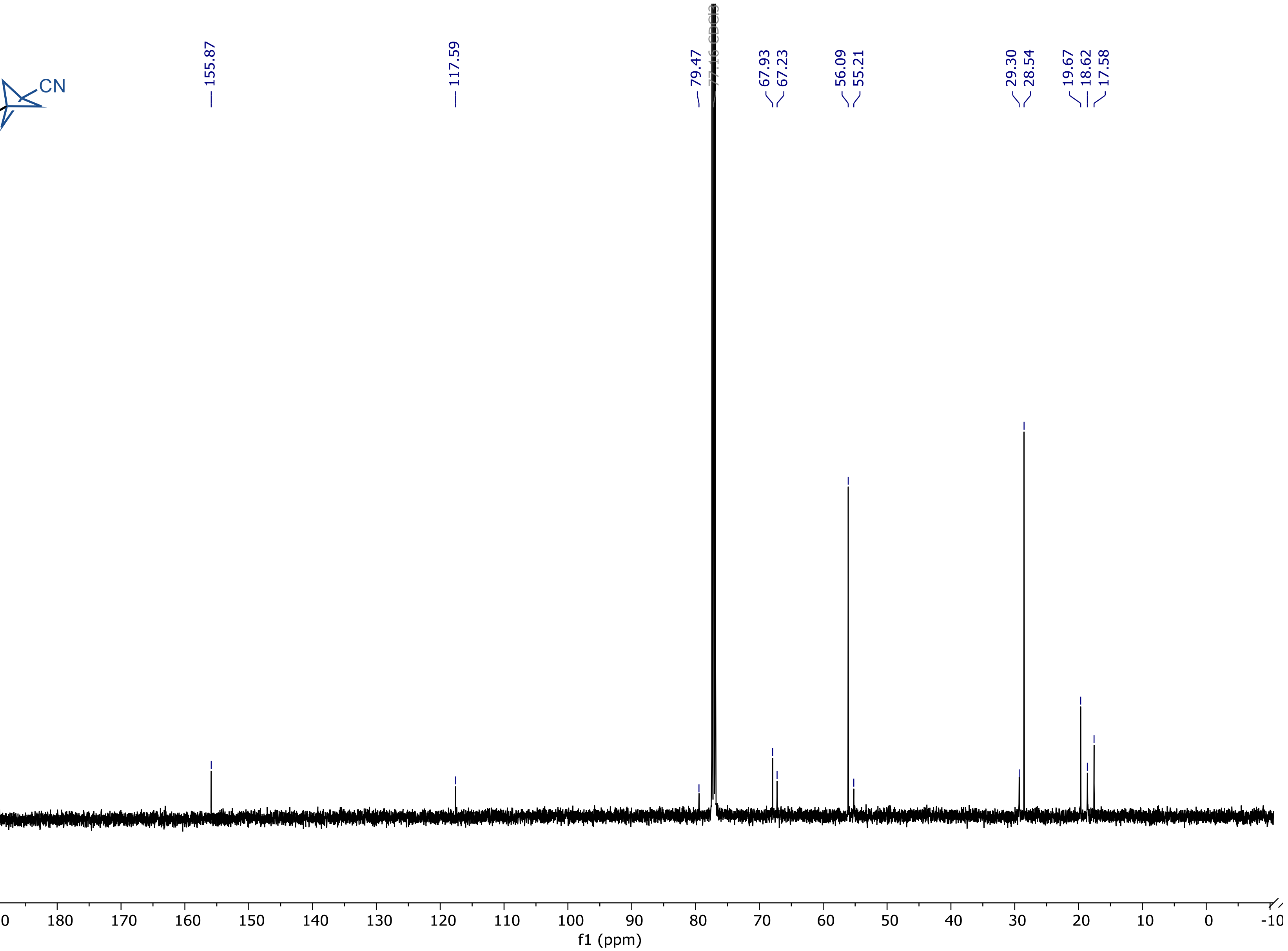

^{1}H NMR of bicyclo[1.1.1]pentylether 28

$CDCl_3$, 298 K

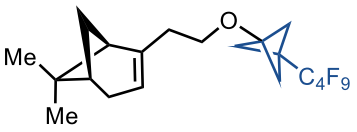

28

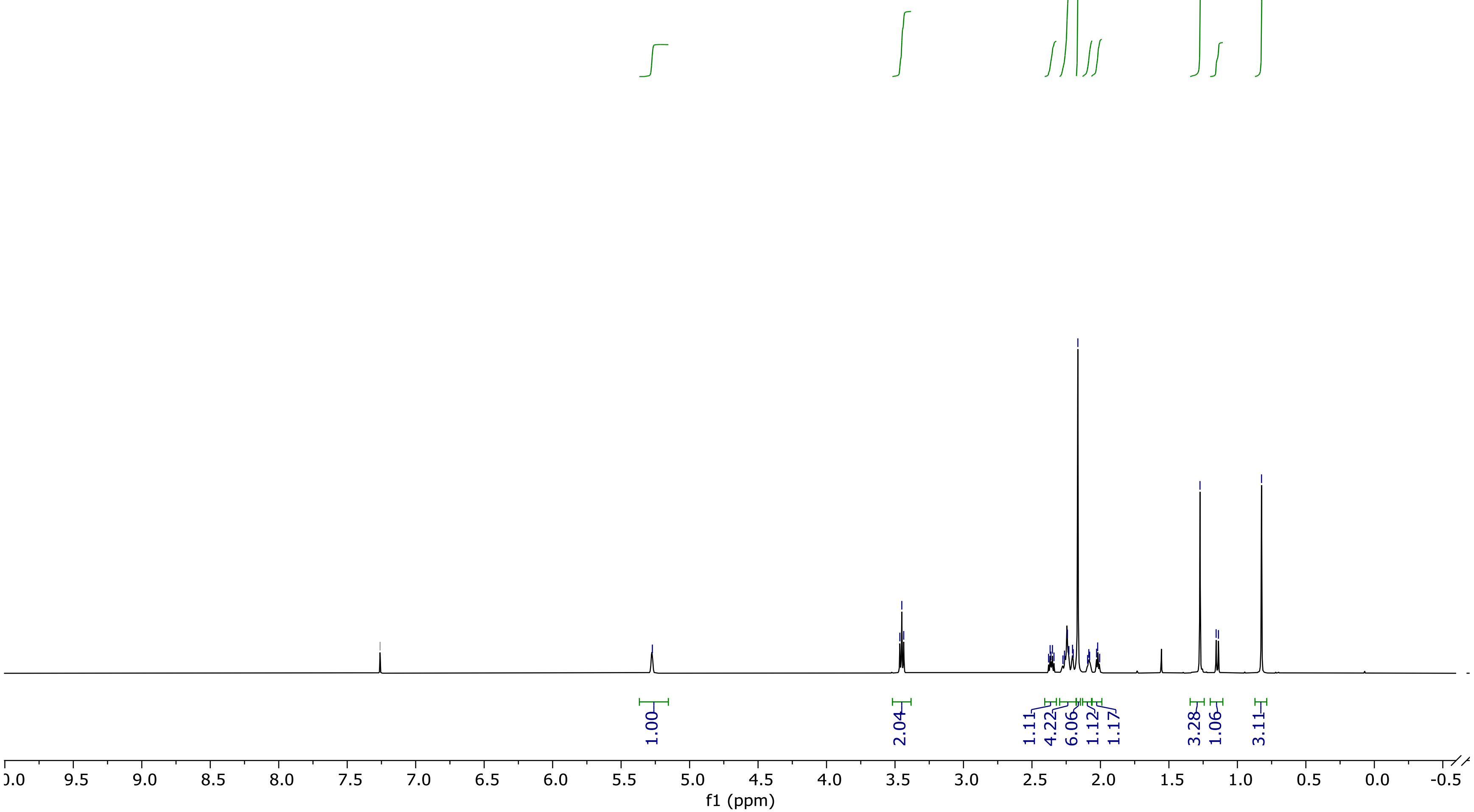

^{13}C {1H, 19F} NMR of bicyclo[1.1.1]pentylether 28

$CDCl_3$, 298 K

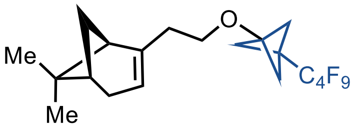

28

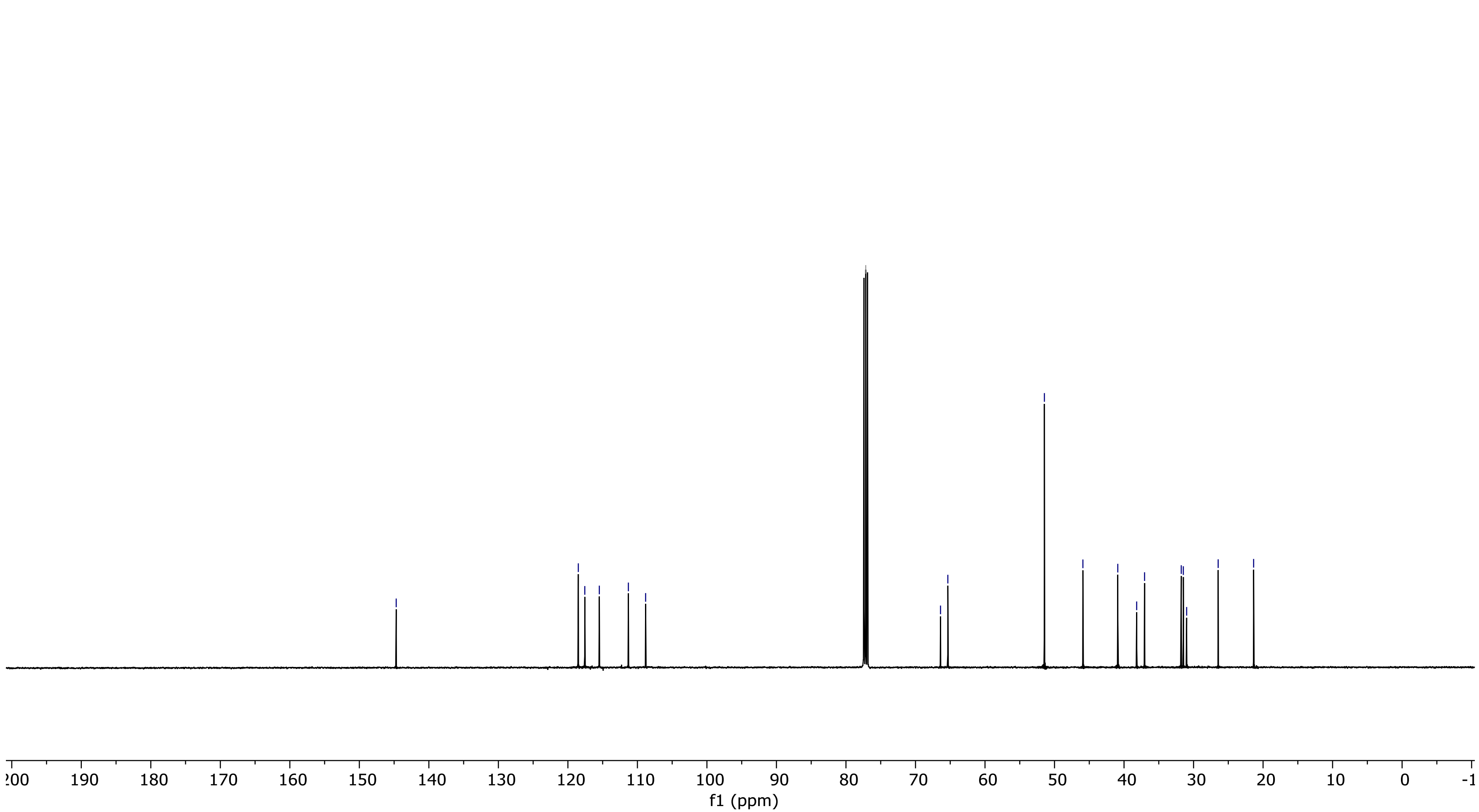

^{19}F NMR of bicyclo[1.1.1]pentylether 28

$CDCl_3$, 298 K

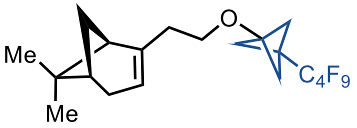

28

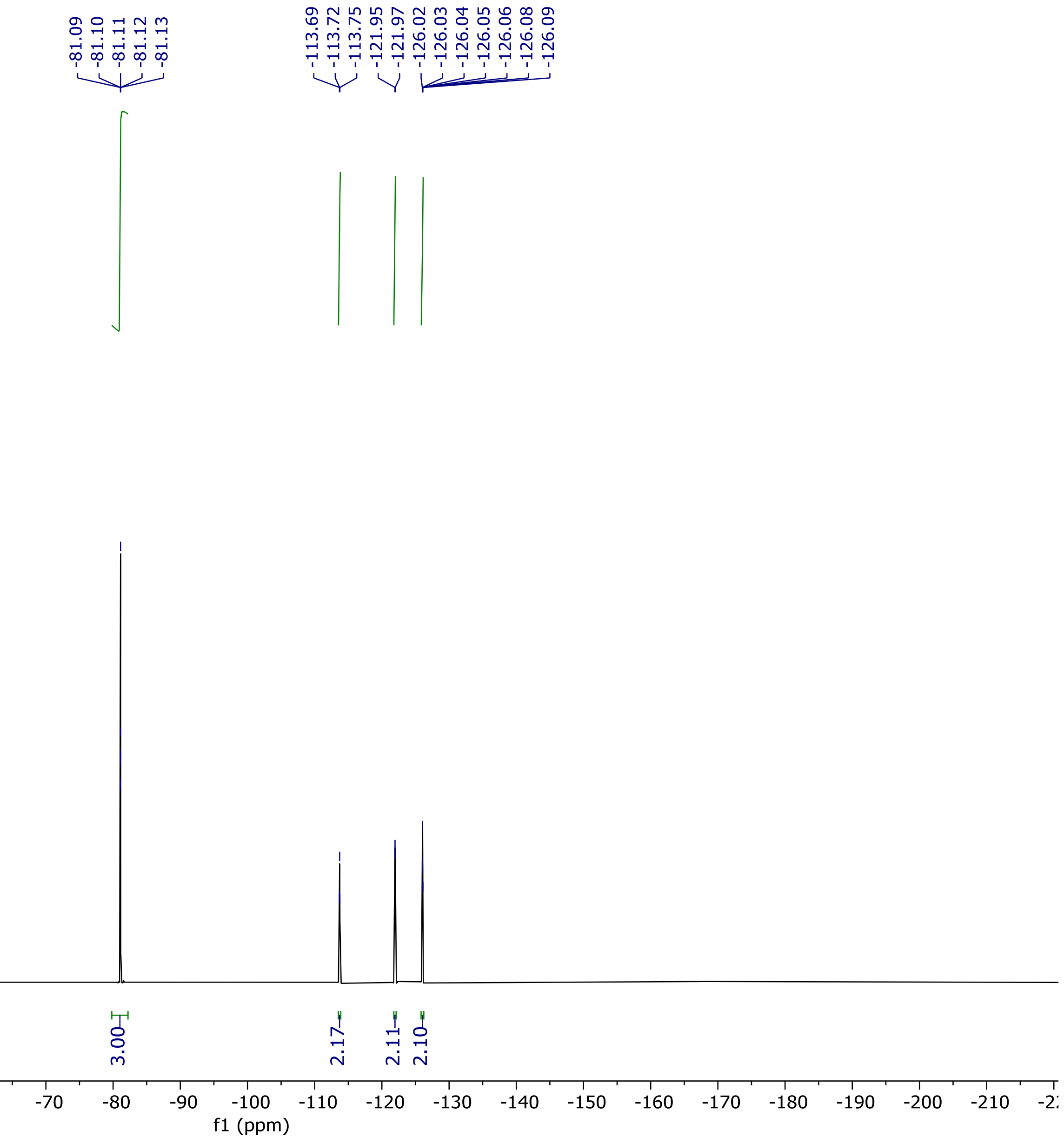

^{1}H NMR of bicyclo[1.1.1]pentylether 29

$CDCl_3$, 298 K

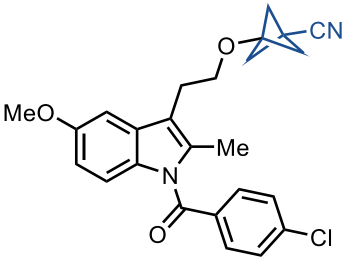

29

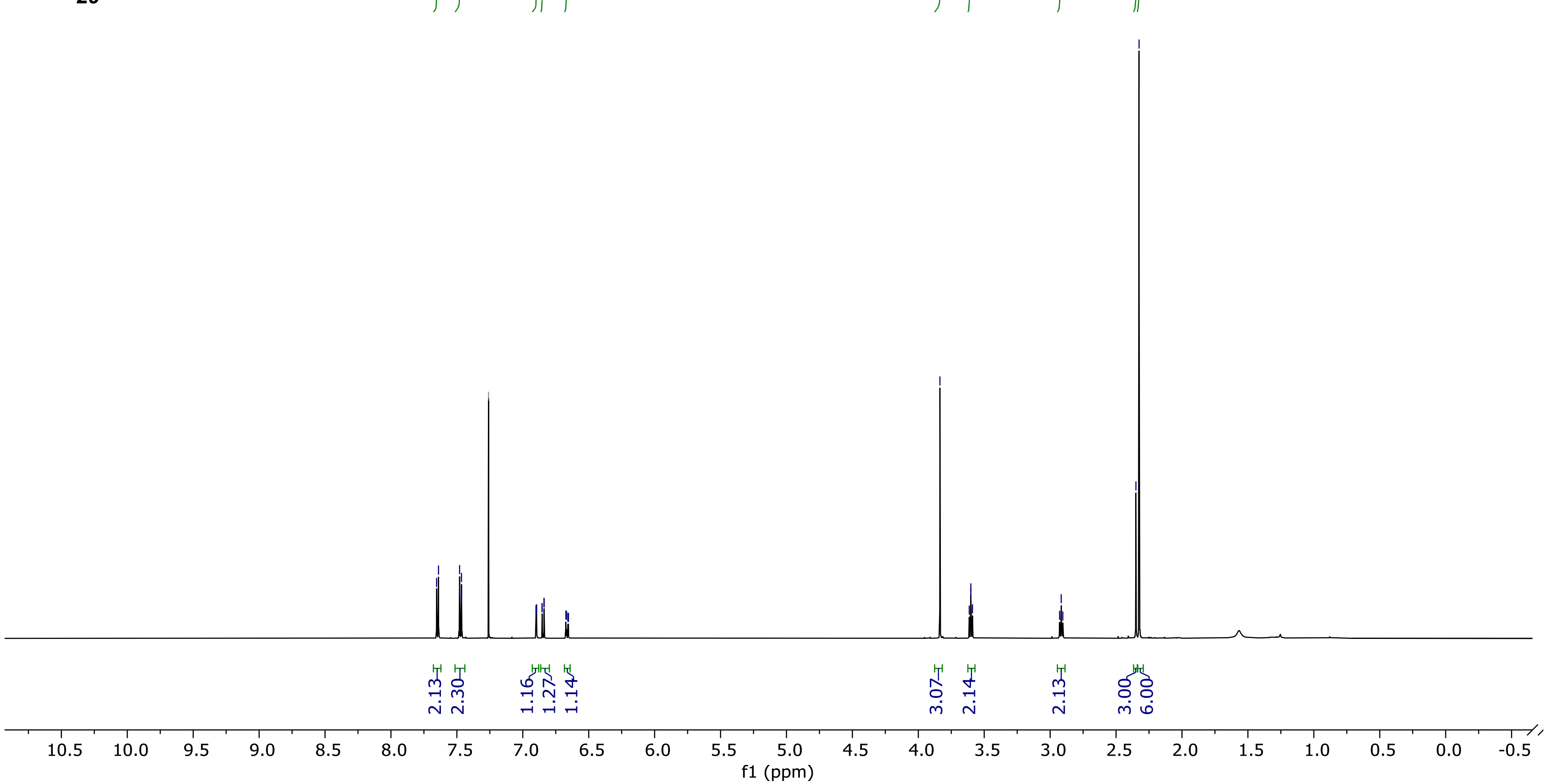

^{13}C NMR of bicyclo[1.1.1]pentylether 29

CDCl$_3$, 298 K

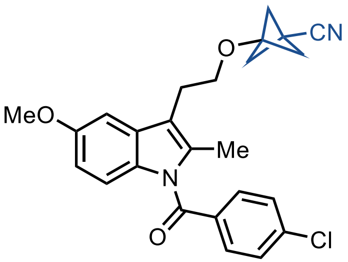

29

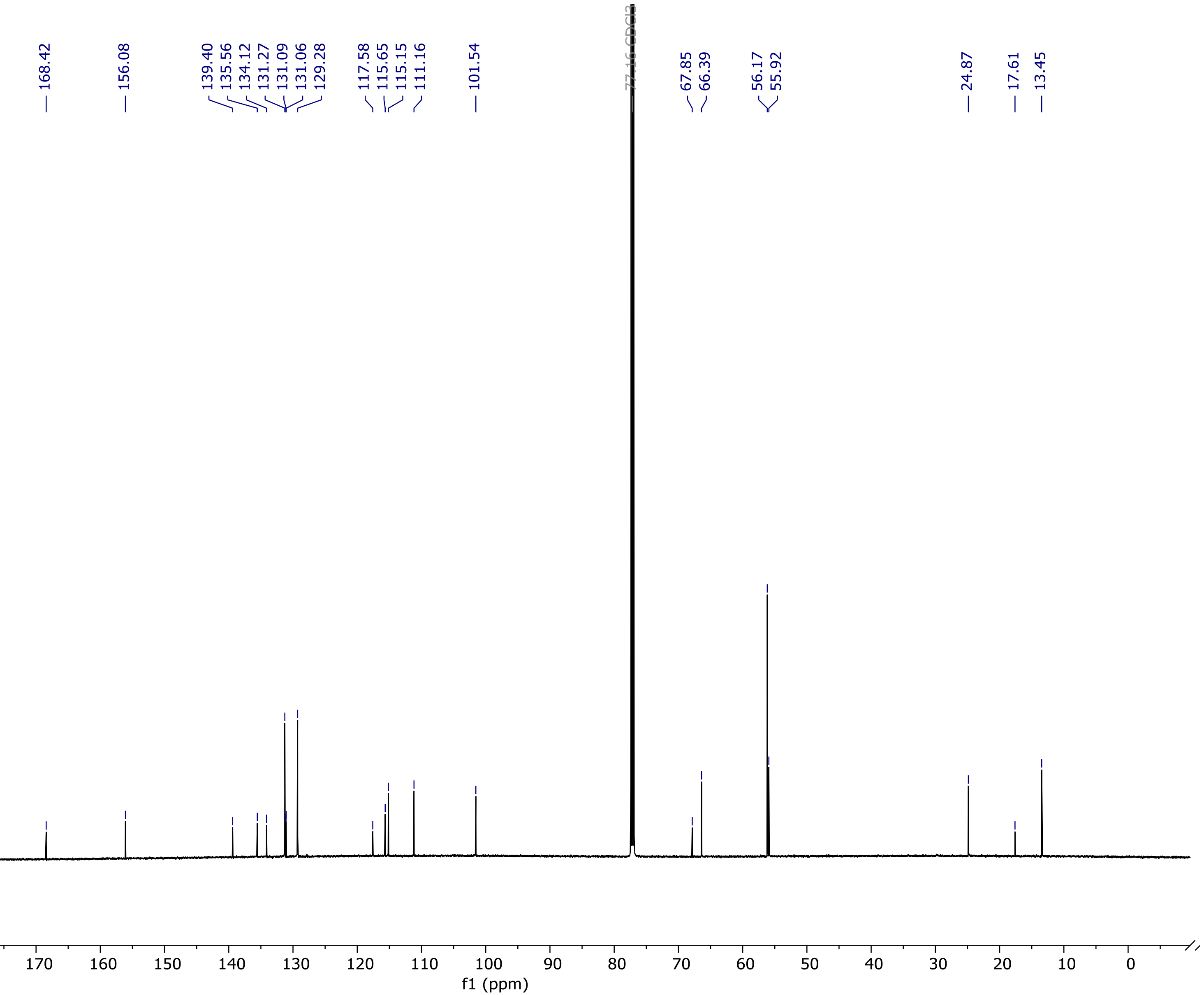

^{1}H NMR of bicyclo[1.1.1]pentylether 30

CDCl3, 298 K

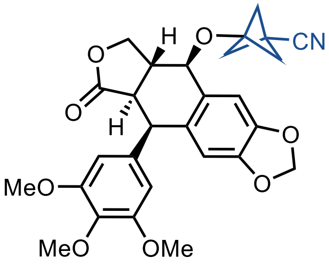

30

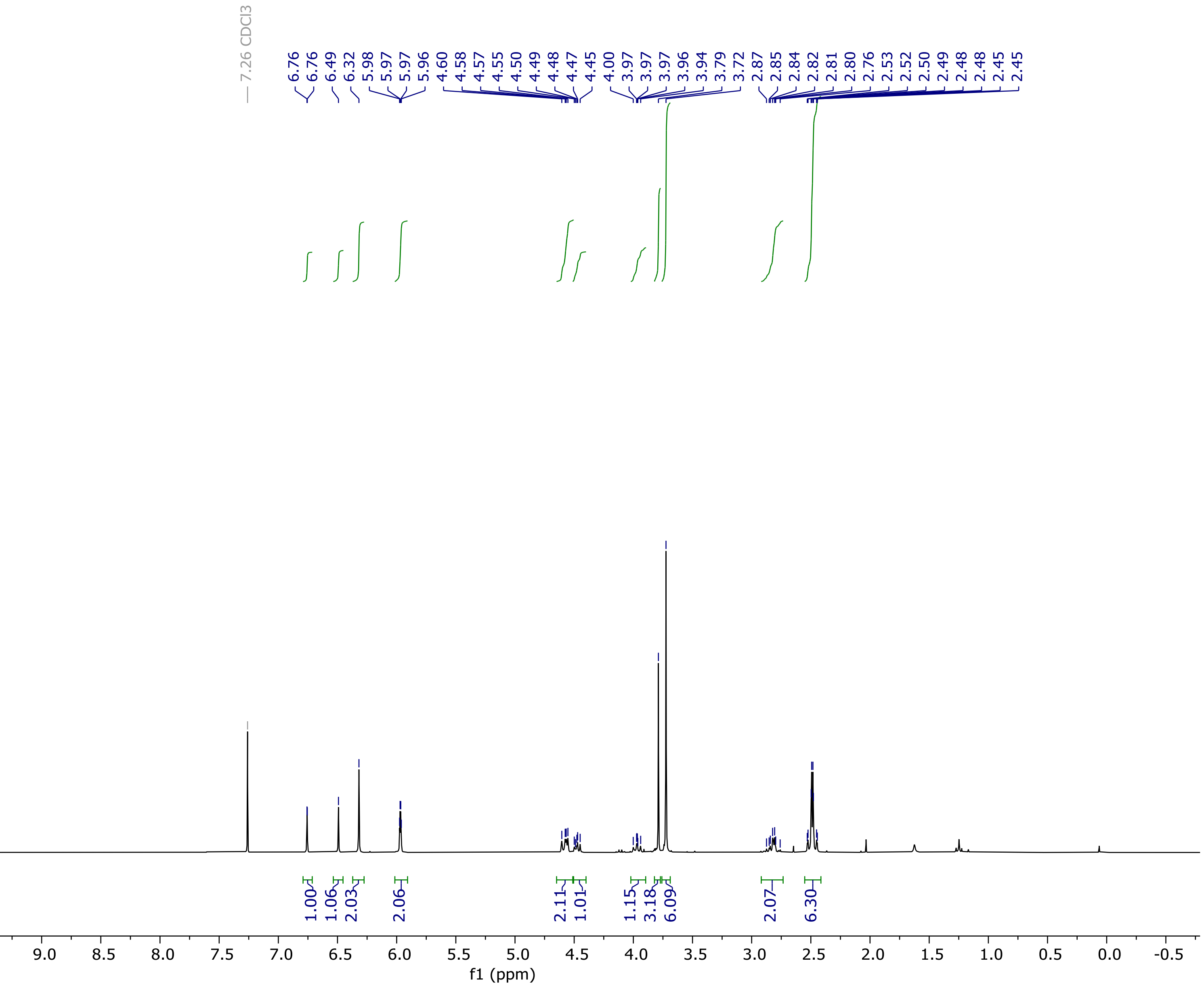

^{13}C NMR of bicyclo[1.1.1]pentylether 30

$CDCl_3$, 298 K

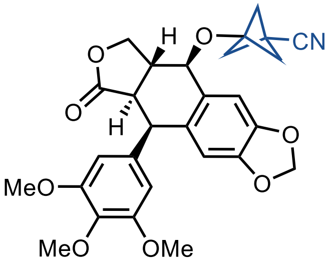

30

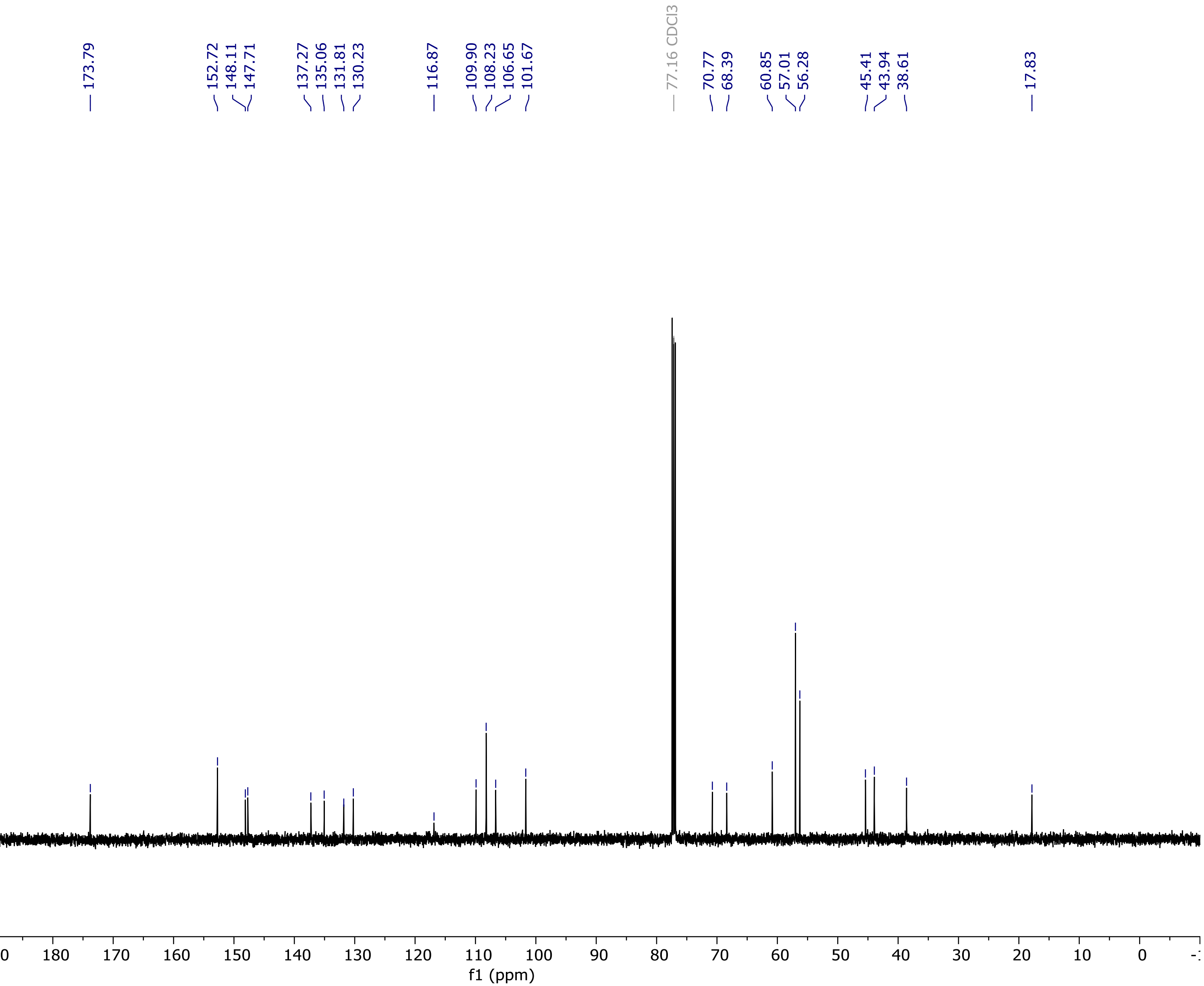

^{1}H NMR of bicyclo[1.1.1]pentylether 35

$CDCl_3$, 298 K

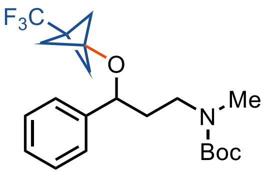

35

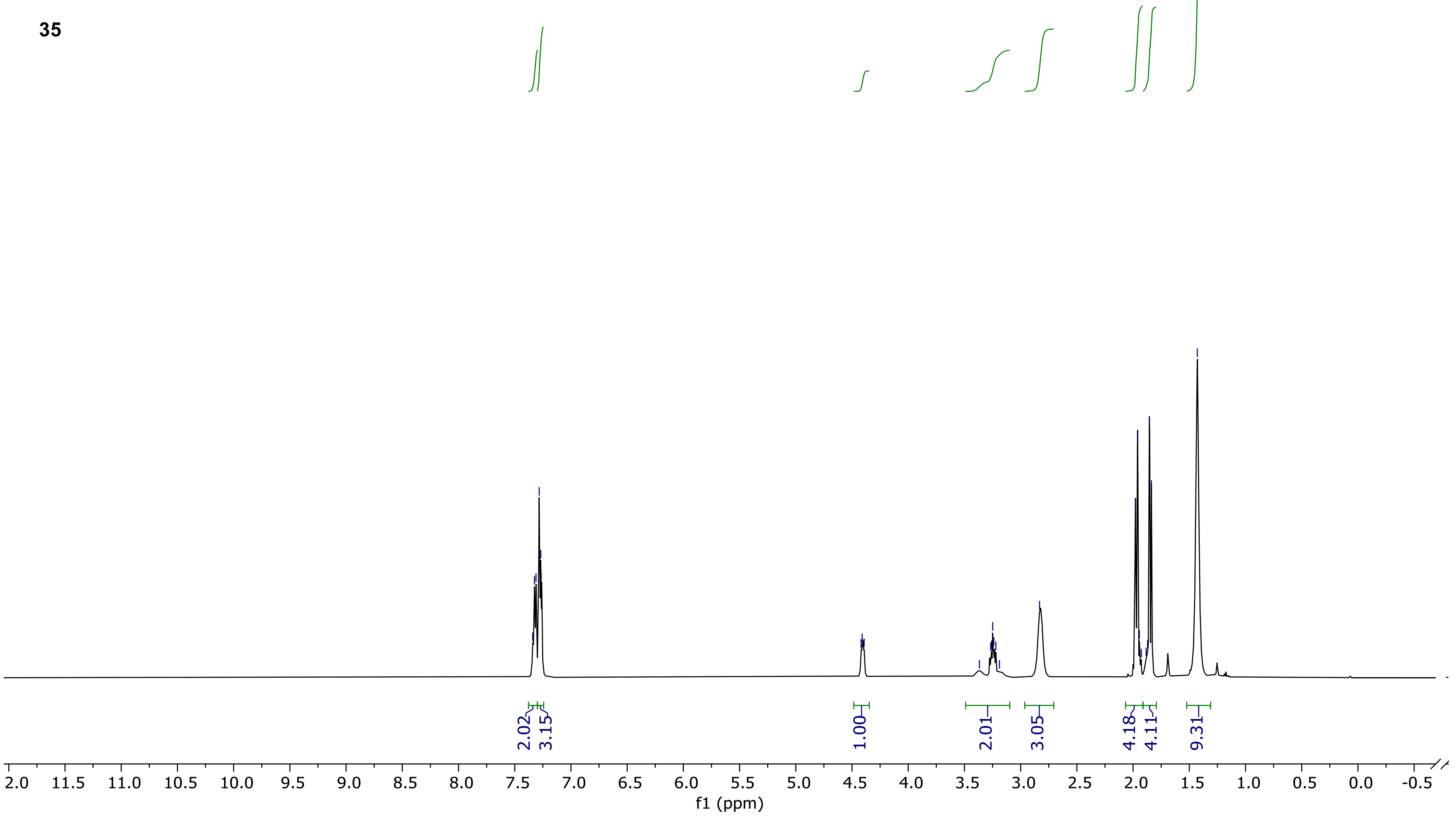

^{13}C NMR of bicyclo[1.1.1]pentylether 35

$CDCl_3$, 298 K

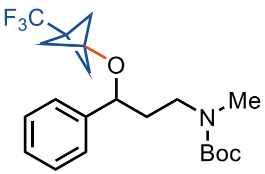

35

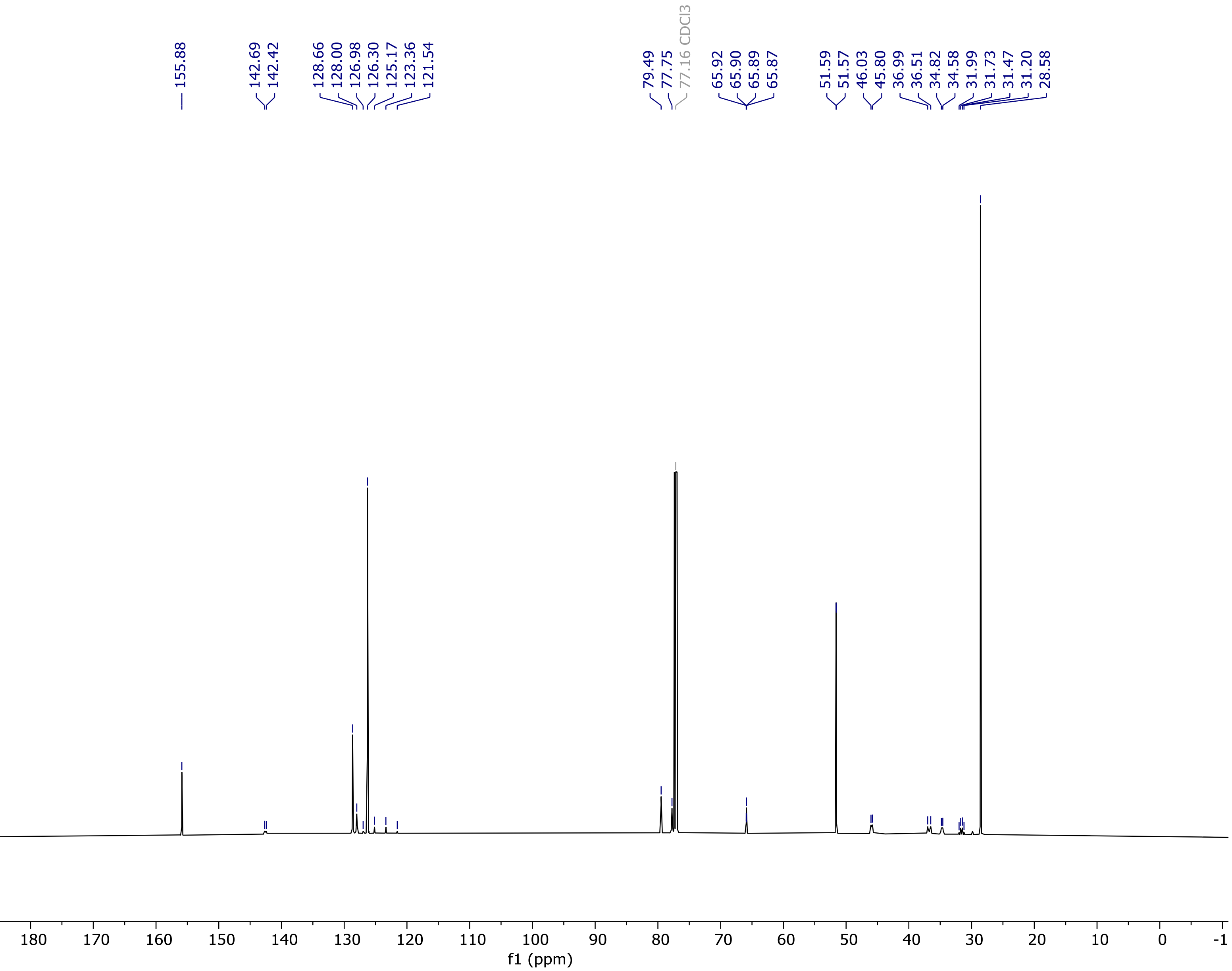

^{19}F NMR of bicyclo[1.1.1]pentylether 35

$CDCl_3$, 298 K

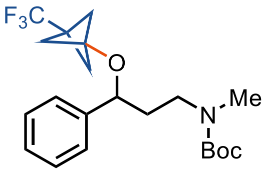

35

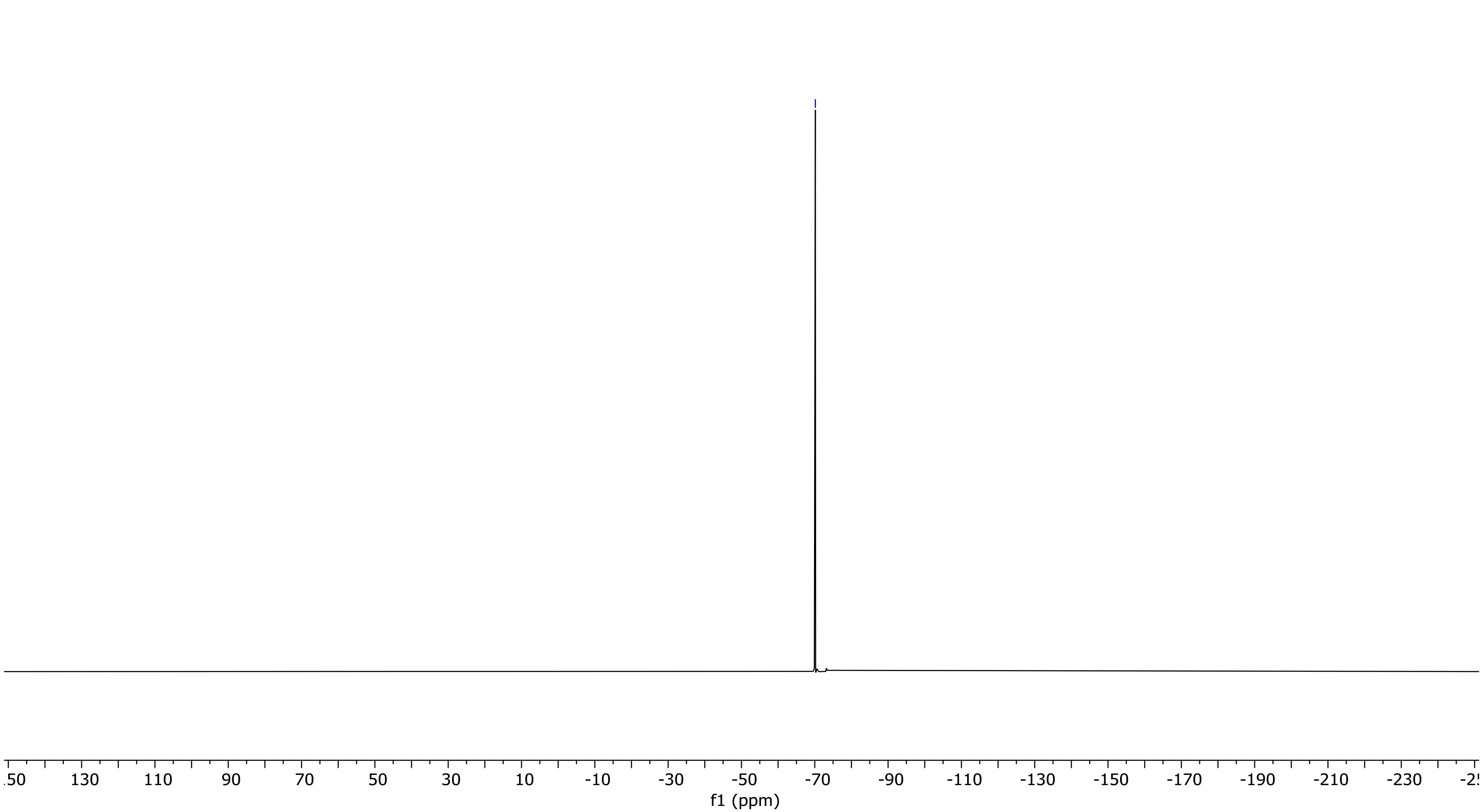

^{1}H NMR of bicyclo[1.1.1]pentylether 36

$CDCl_3$, 298 K

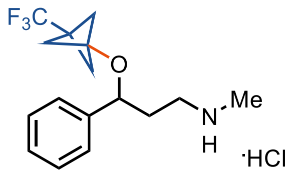

36

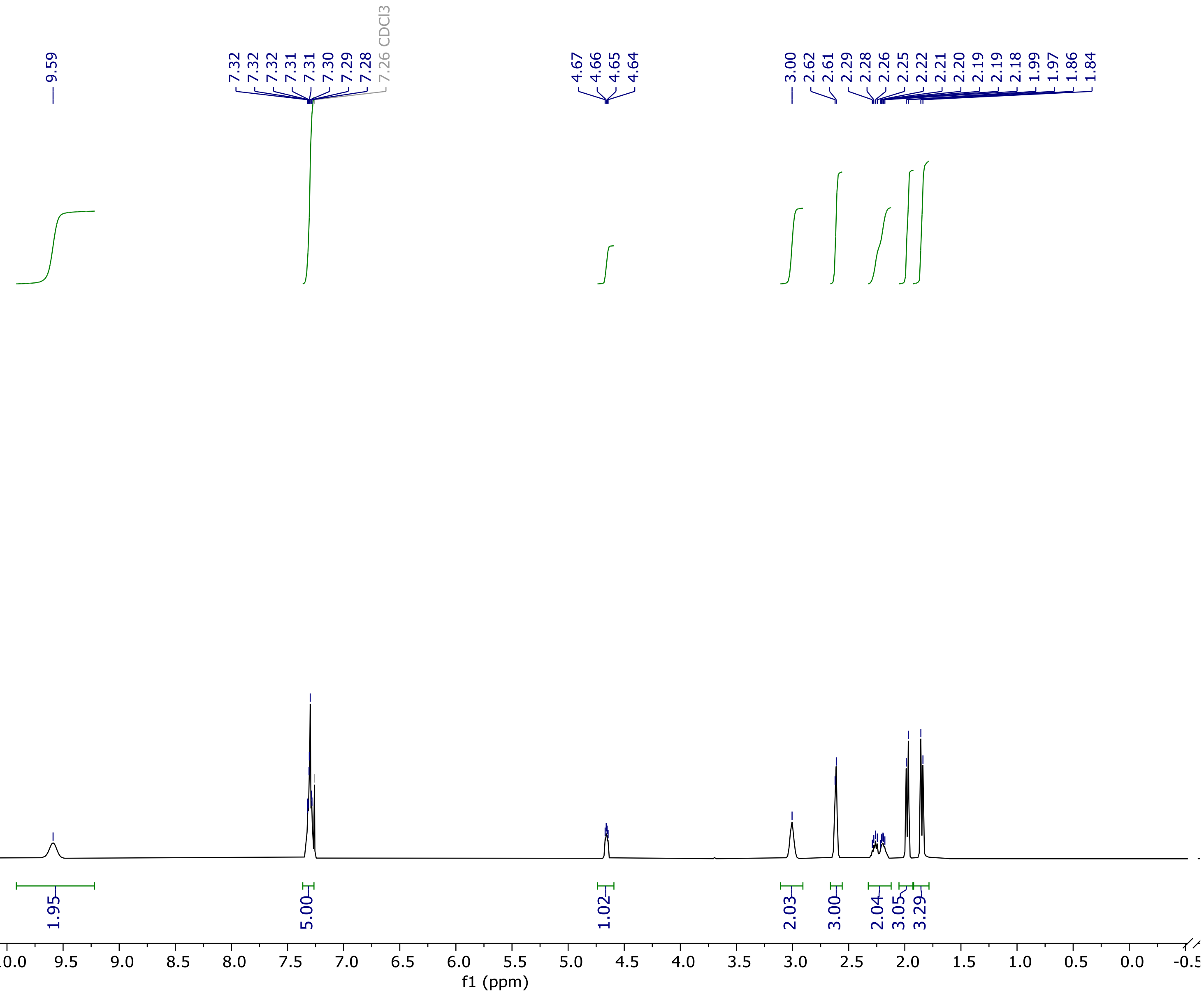

^{13}C NMR of bicyclo[1.1.1]pentylether 36

$CDCl_3$, 298 K

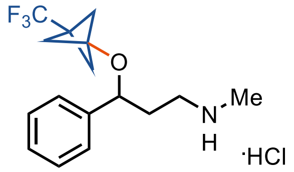

36

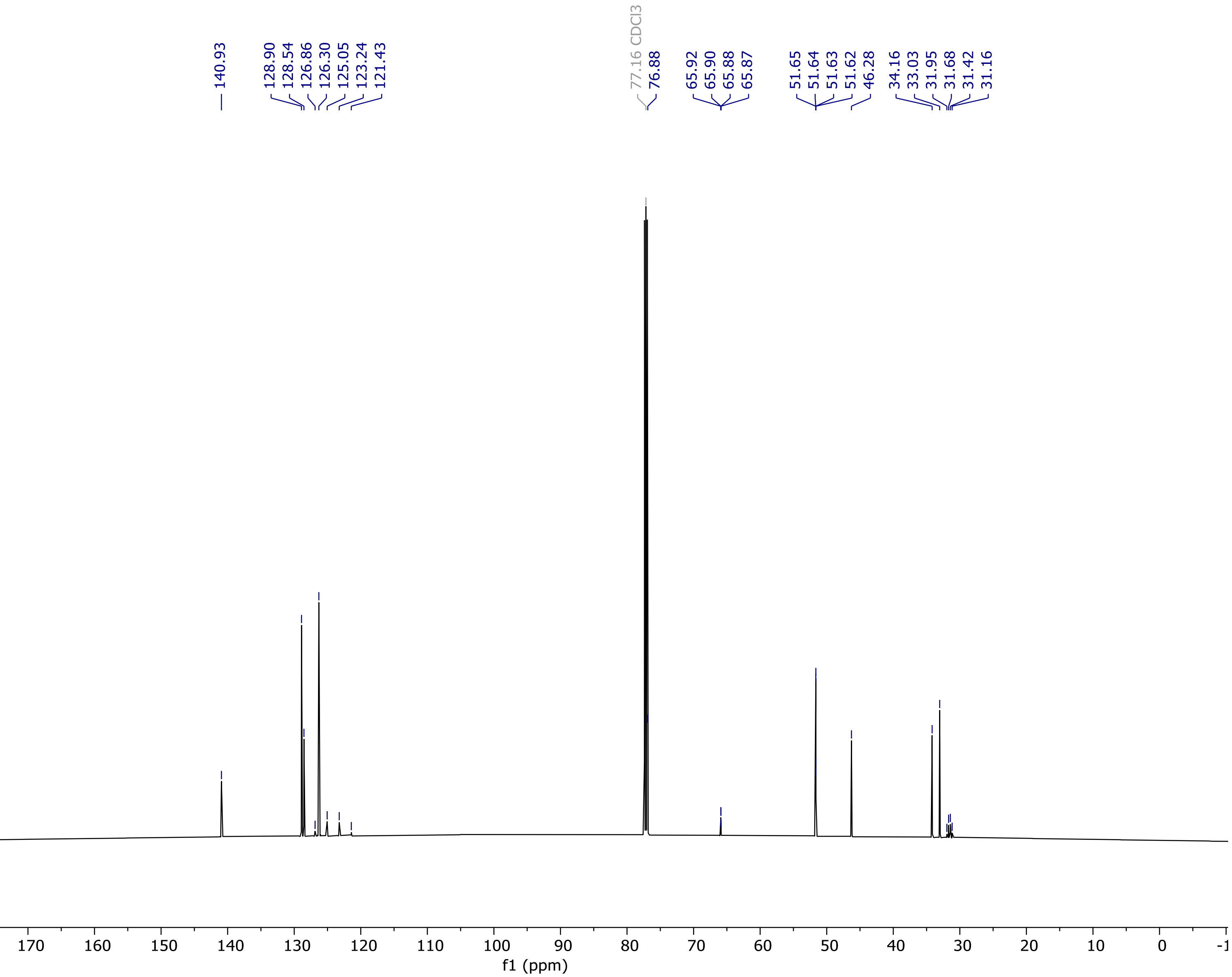

^{19}F NMR of bicyclo[1.1.1]pentylether 36

$CDCl_3$, 298 K

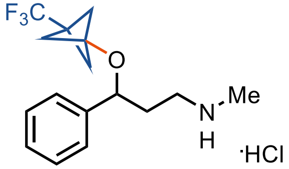

36

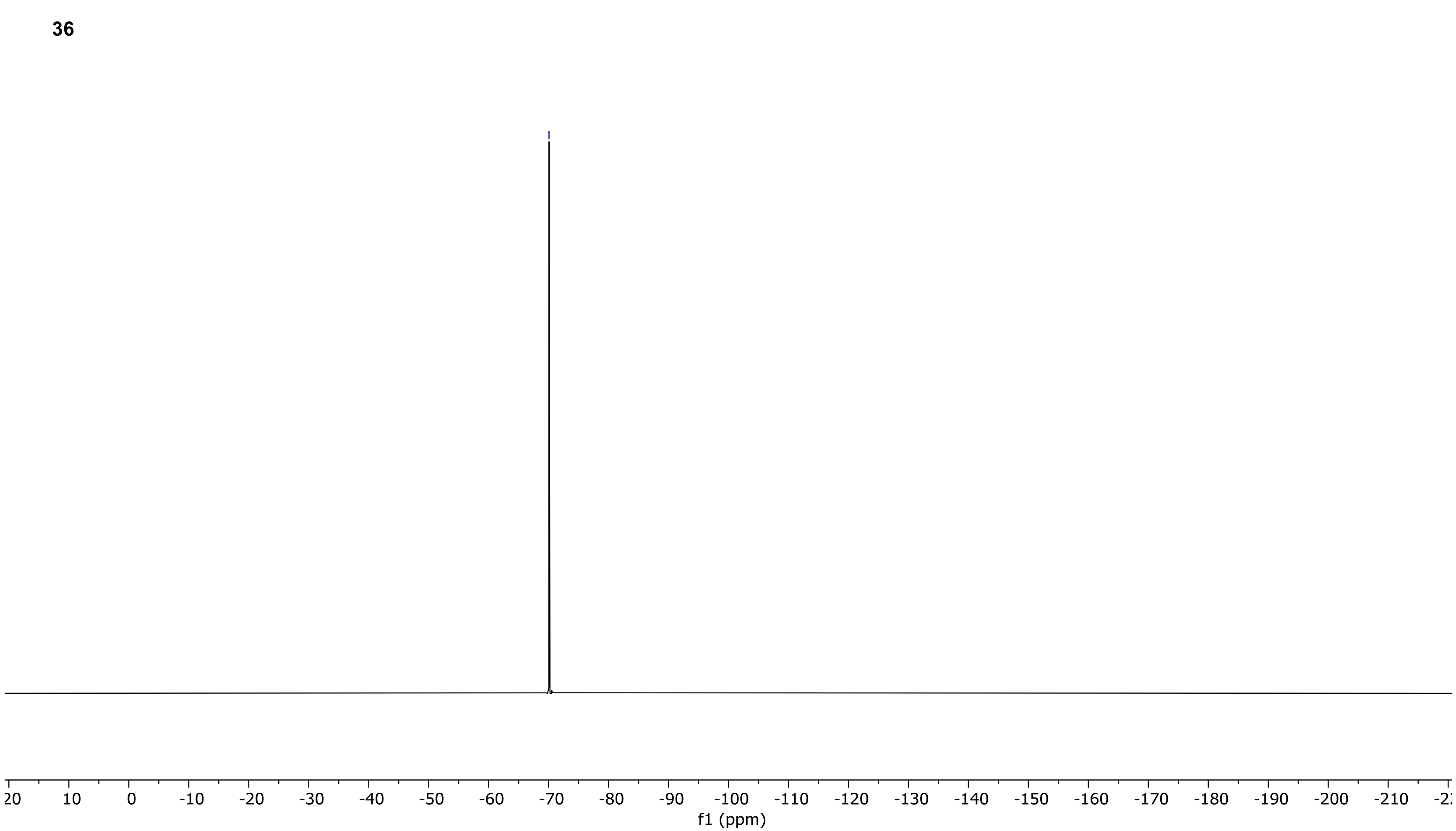

^{1}H NMR of bicyclo[1.1.1]pentylether 37

CDCl$_3$, 298 K

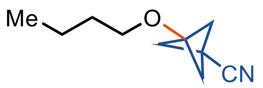

37

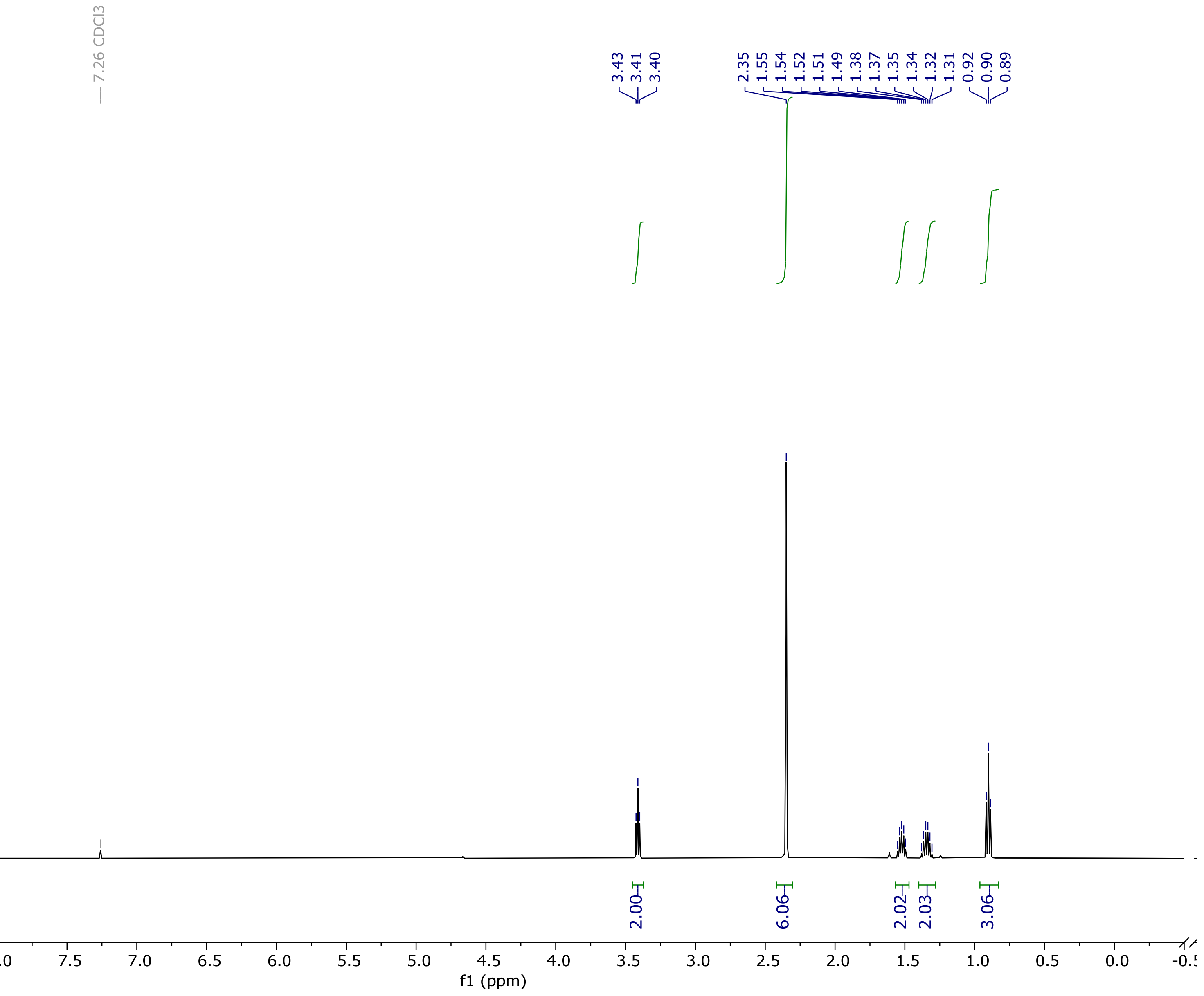

^{13}C NMR of bicyclo[1.1.1]pentylether 37

CDCl$_3$, 298 K

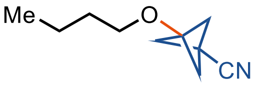

37

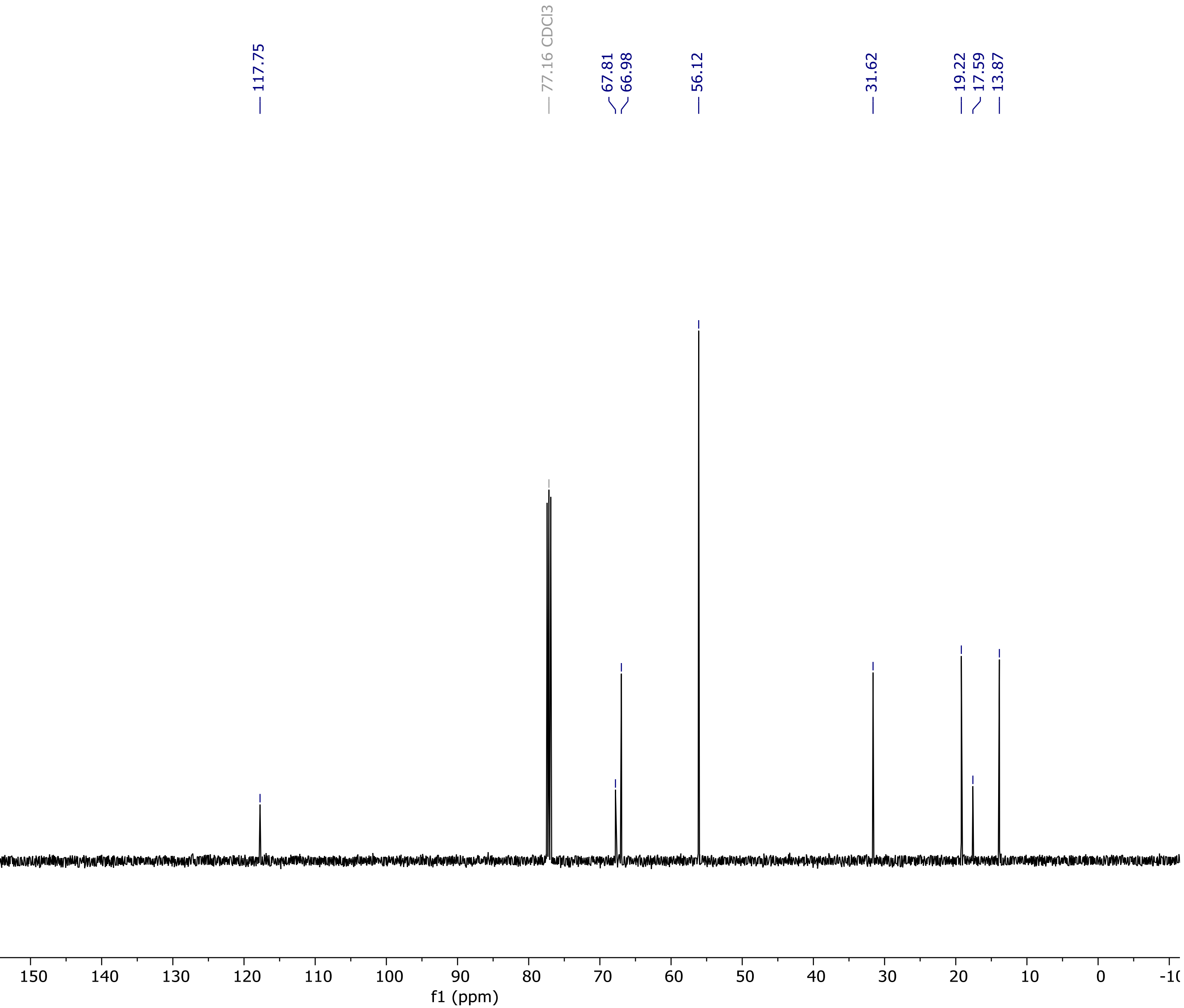

^{1}H NMR of bicyclo[1.1.1]pentylether 37-1

$CDCl_3$, 298 K

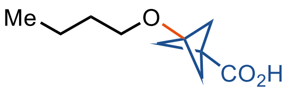

37-1

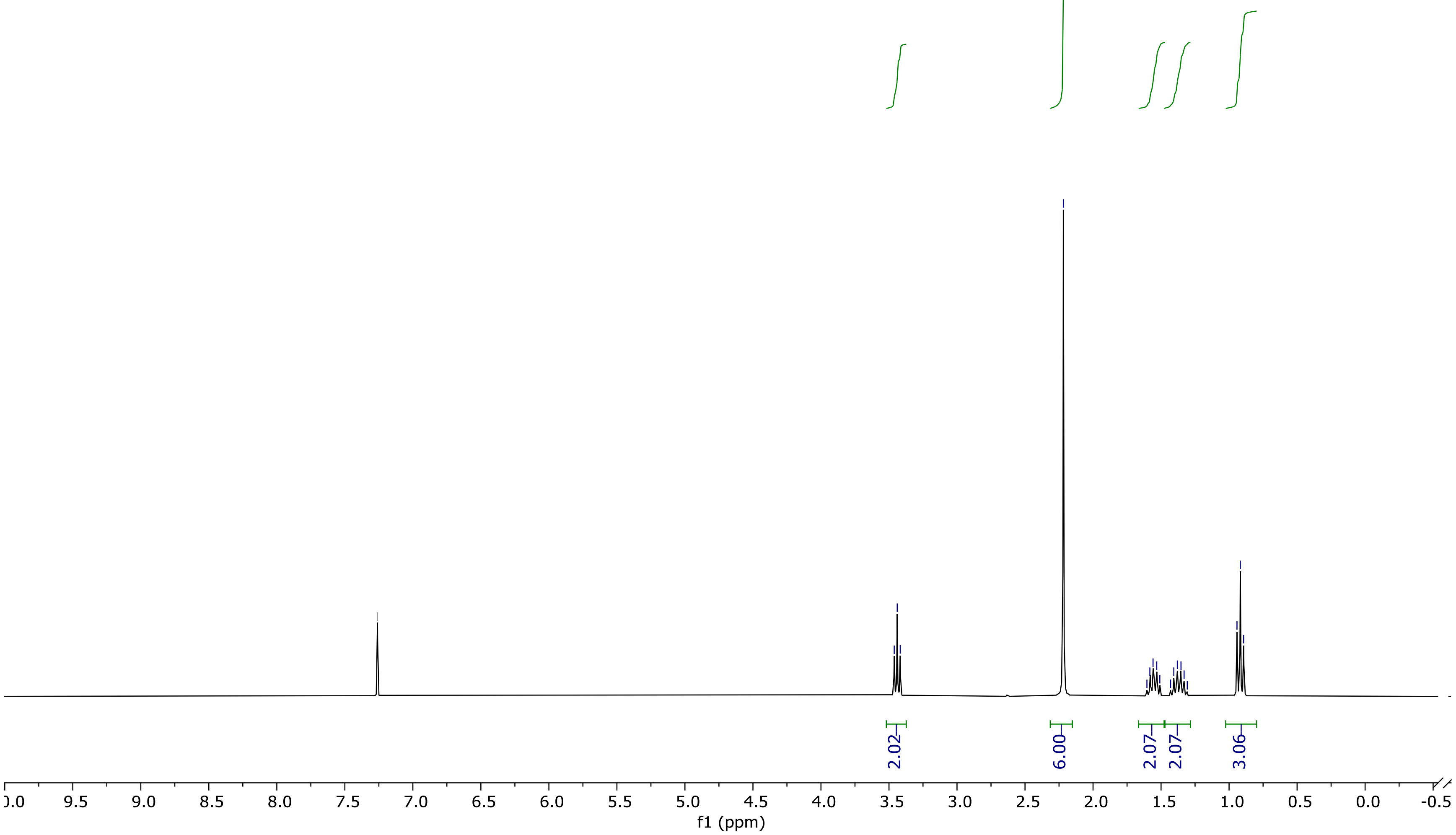

^{13}C NMR of bicyclo[1.1.1]pentylether 37-1

$CDCl_3$, 298 K

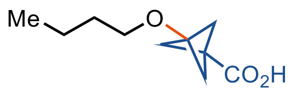

37-1

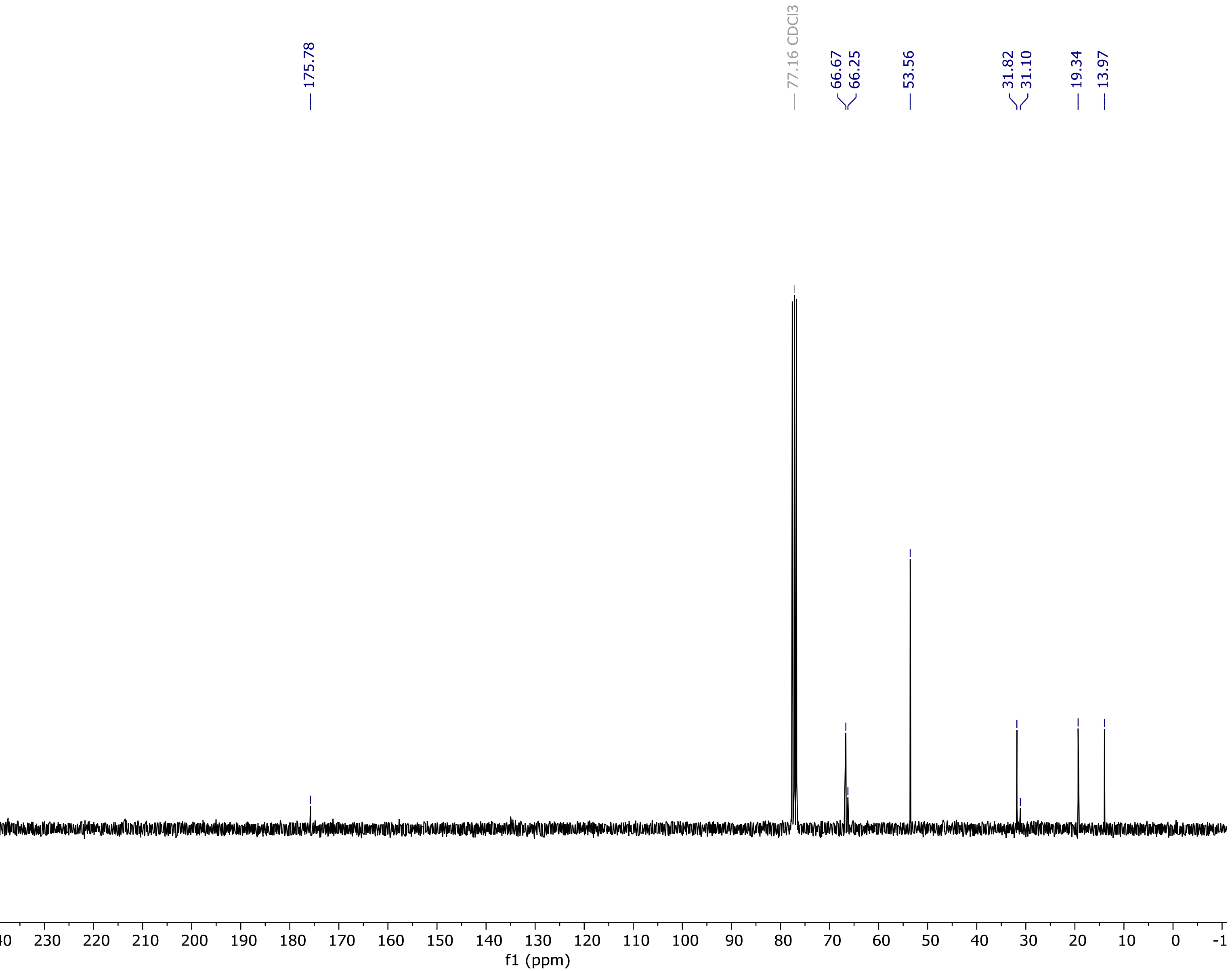

^{1}H NMR of bicyclo[1.1.1]pentylether 38

CDCl$_3$, 298 K

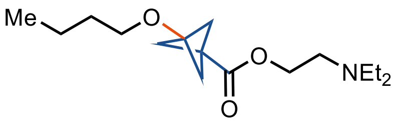

38

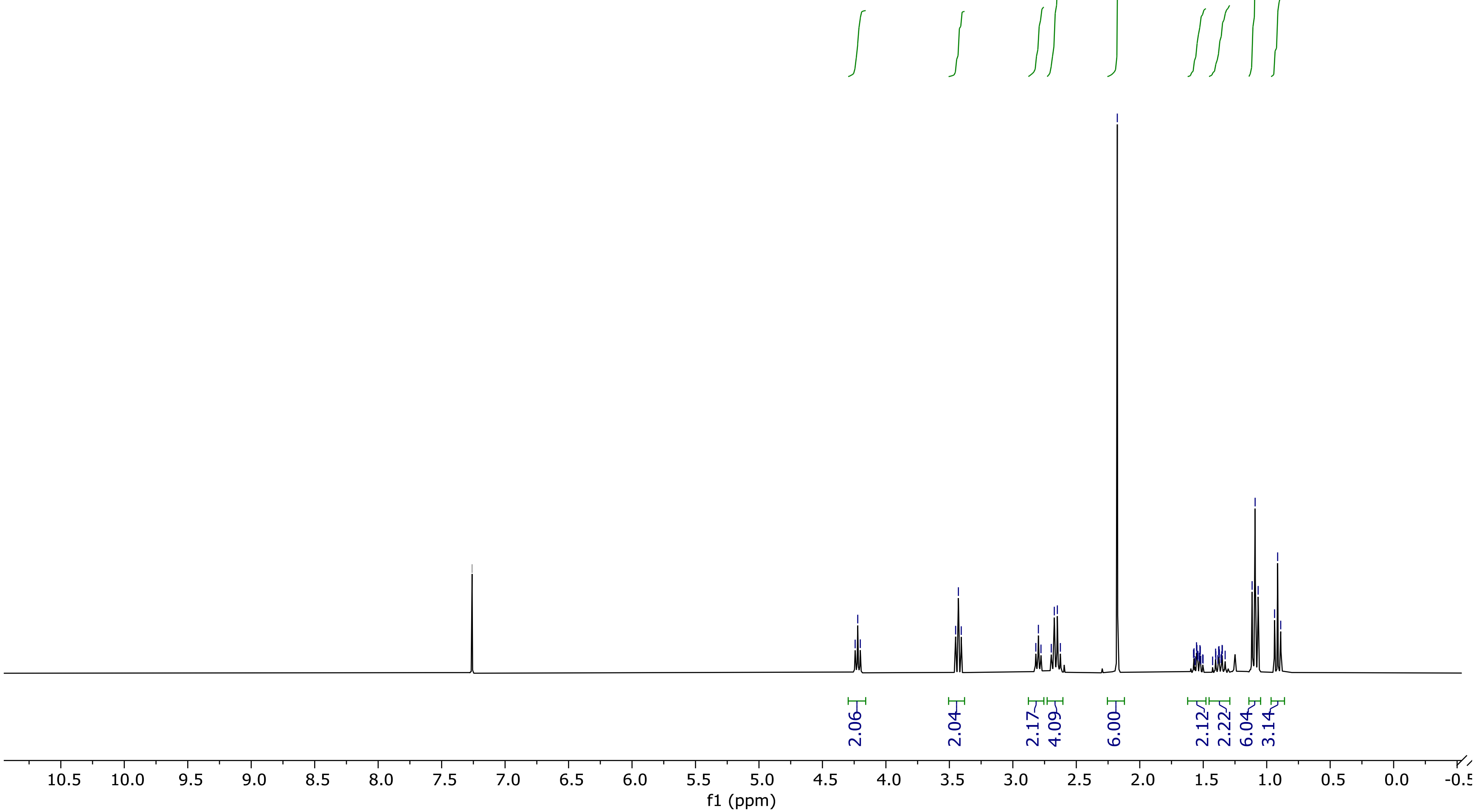

^{13}C NMR of bicyclo[1.1.1]pentylether 38

$CDCl_3$, 298 K

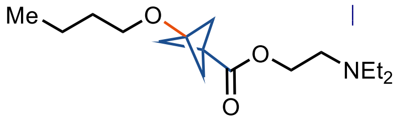

38

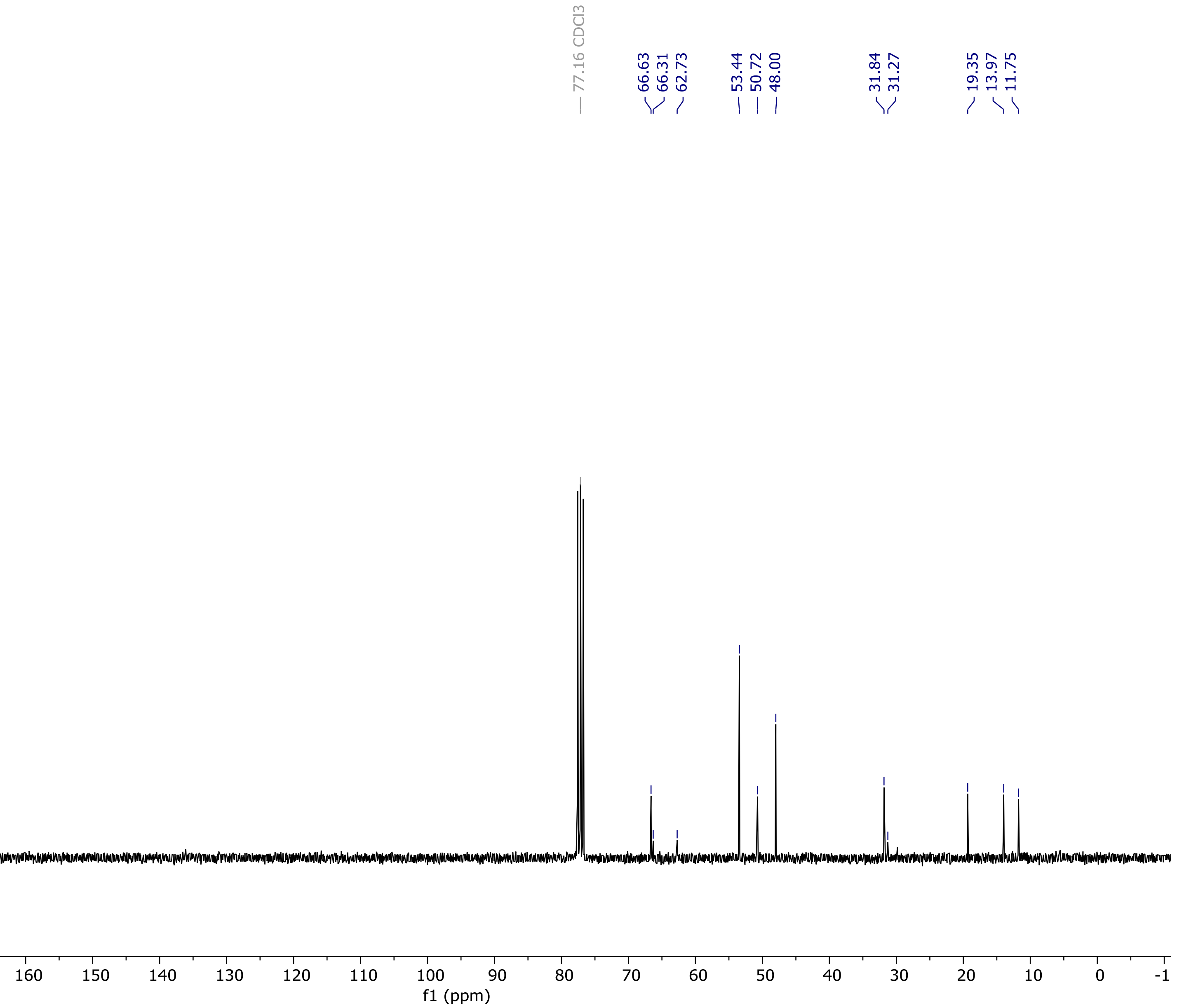

^{1}H NMR of bicyclo[1.1.1]pentylether 39

$CDCl_3$, 298 K

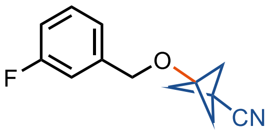

39

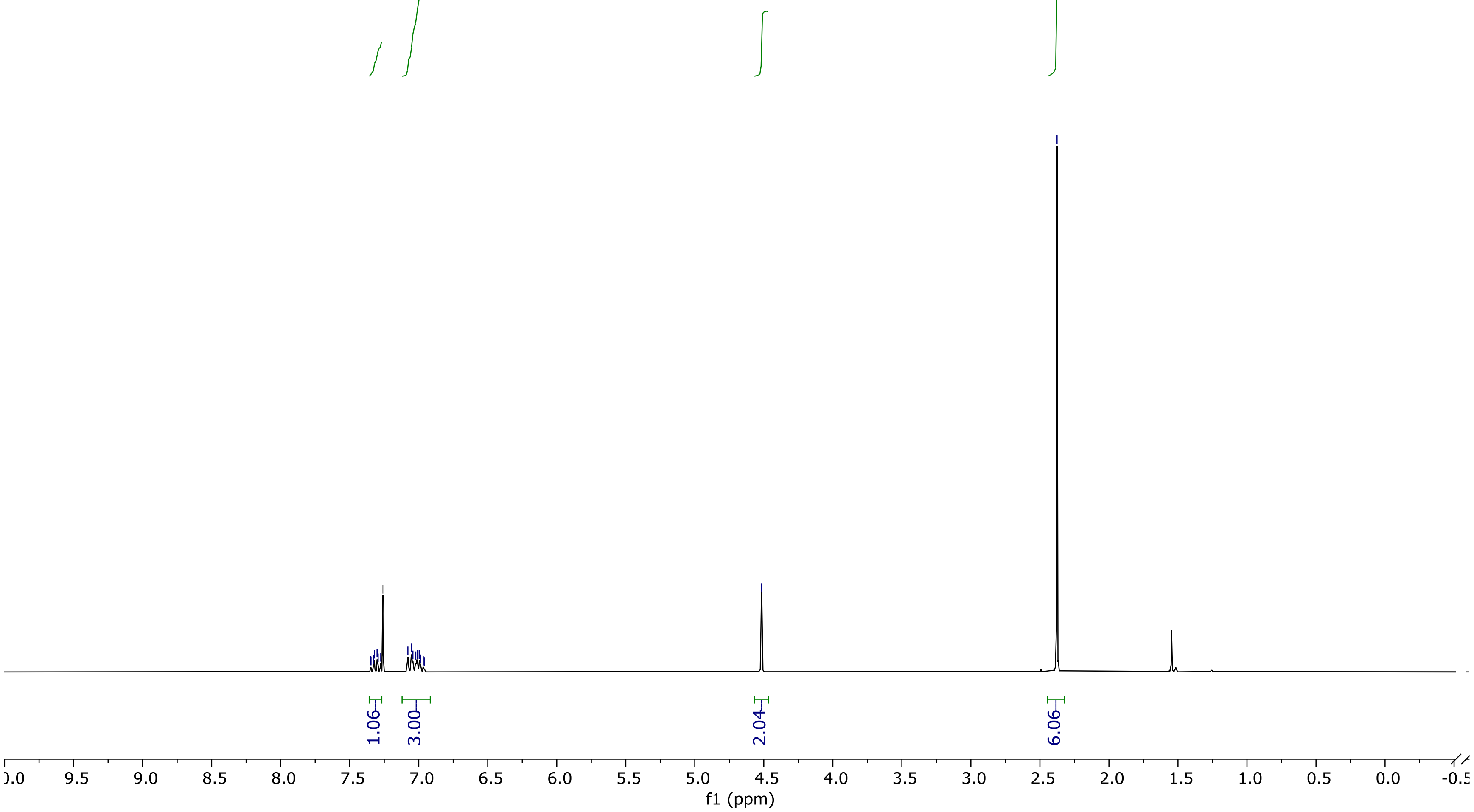

^{13}C NMR of bicyclo[1.1.1]pentylether 39

$CDCl_3$, 298 K

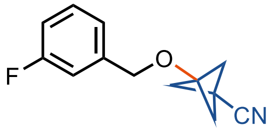

39

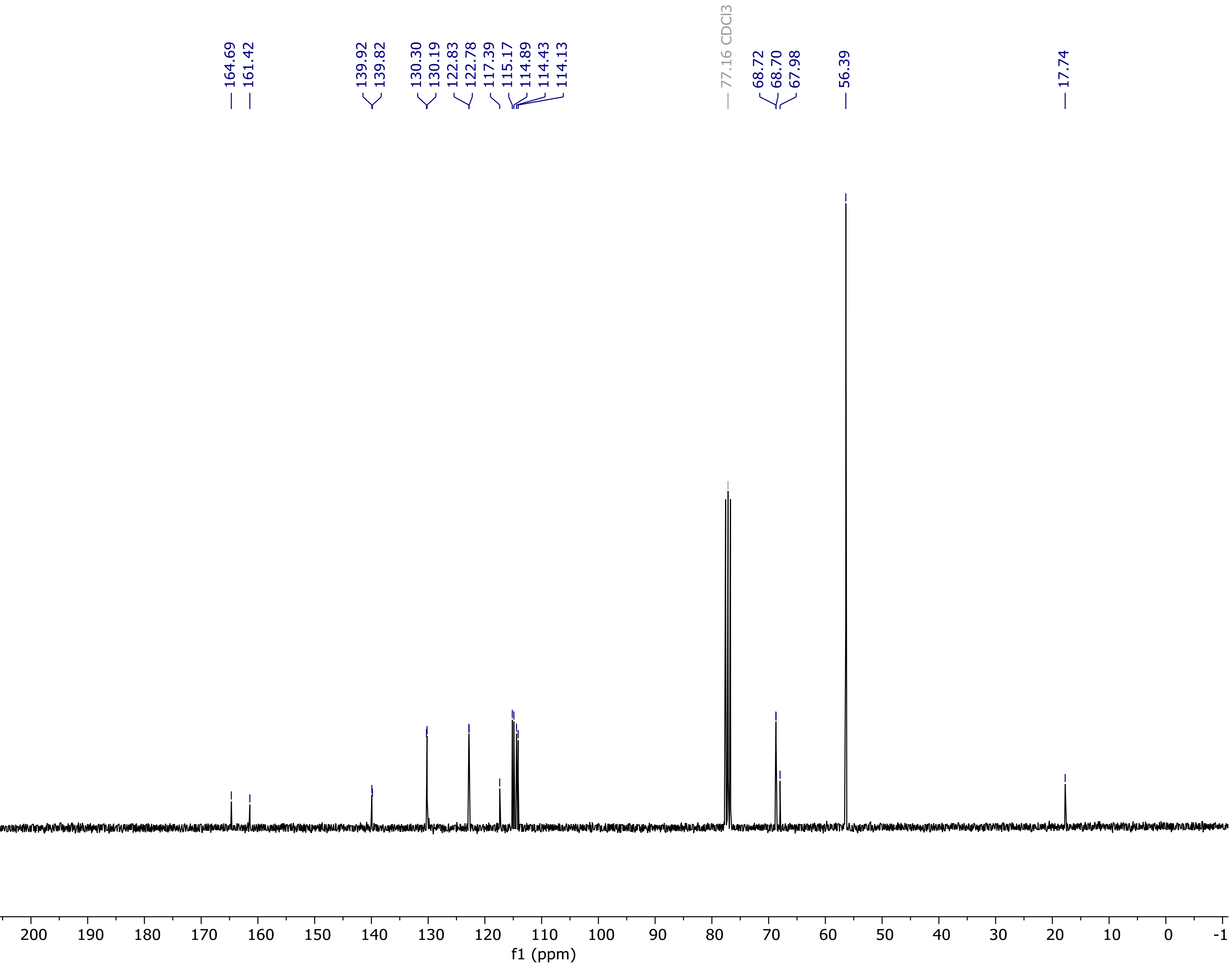

^{19}F NMR of bicyclo[1.1.1]pentylether 39

$CDCl_3$, 298 K

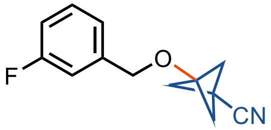

39

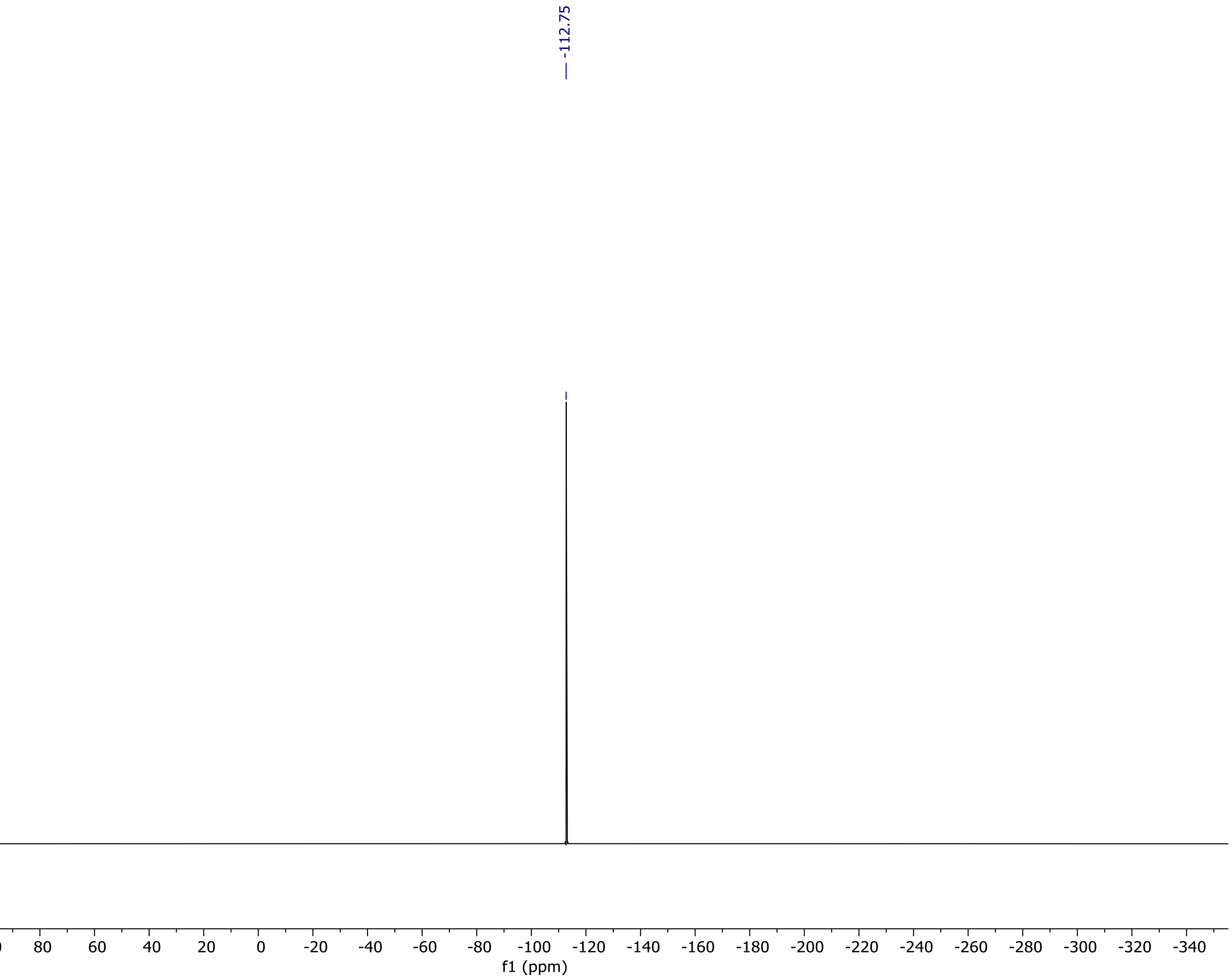

^{1}H NMR of bicyclo[1.1.1]pentylether 39-1

CDCl$_3$, 298 K

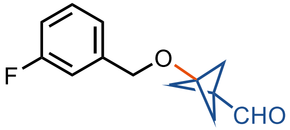

39-1

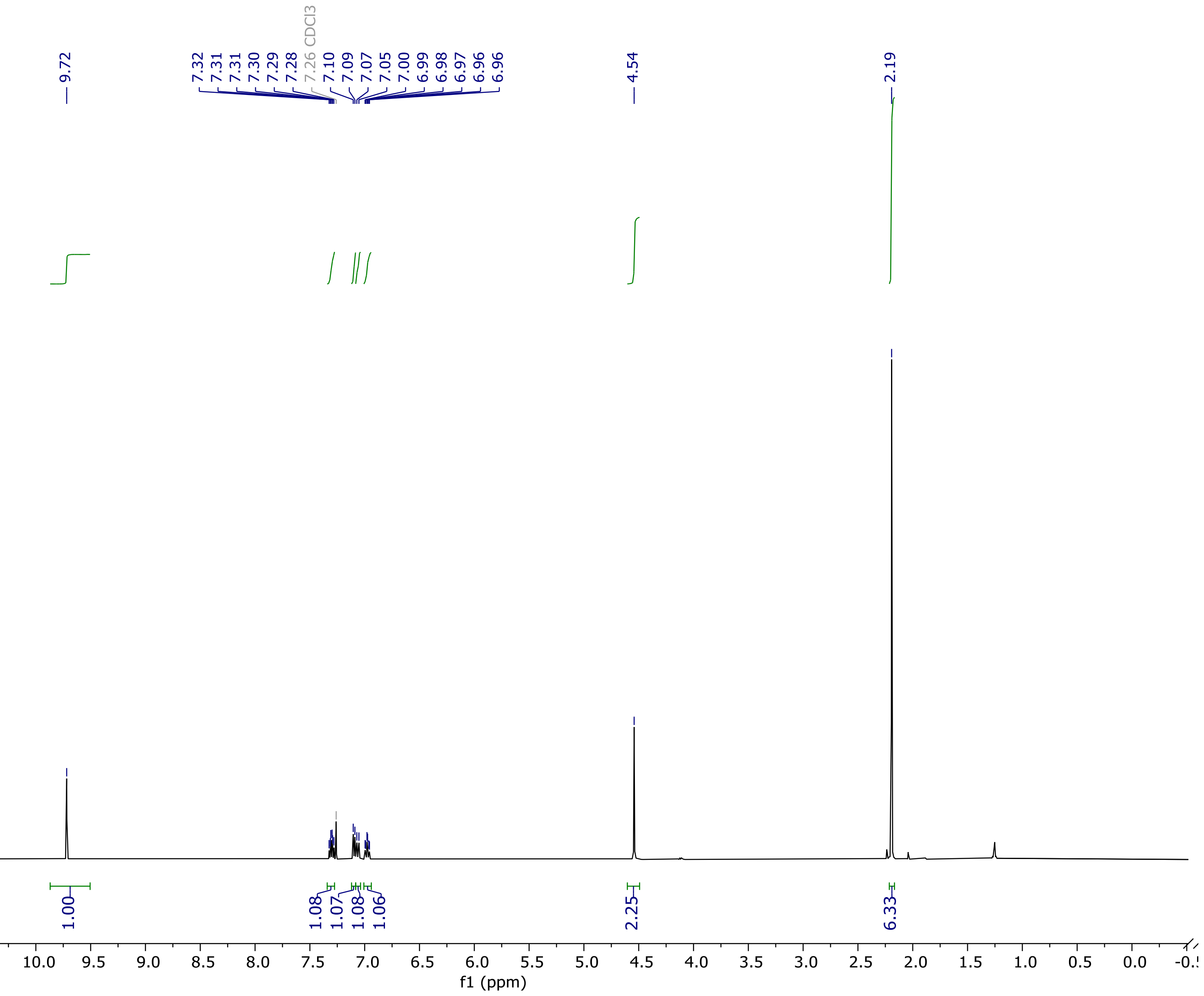

^{13}C NMR of bicyclo[1.1.1]pentylether 39-1

CDCl$_3$, 298 K

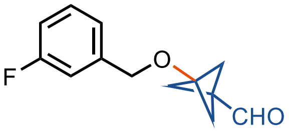

39-1

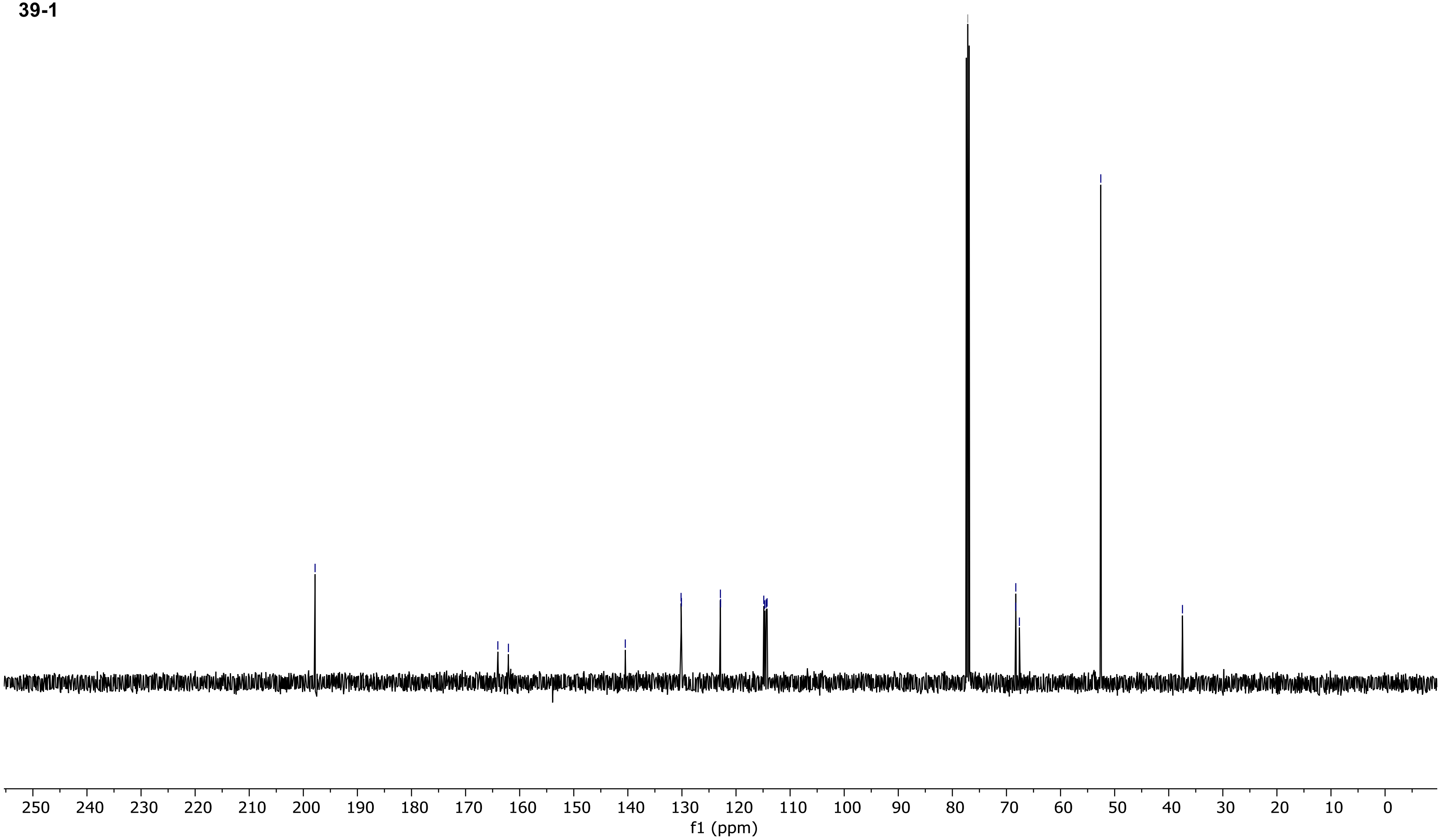

^{19}F NMR of bicyclo[1.1.1]pentylether 39-1

CDCl$_3$, 298 K

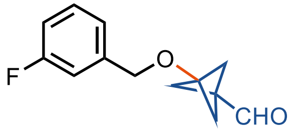

39-1

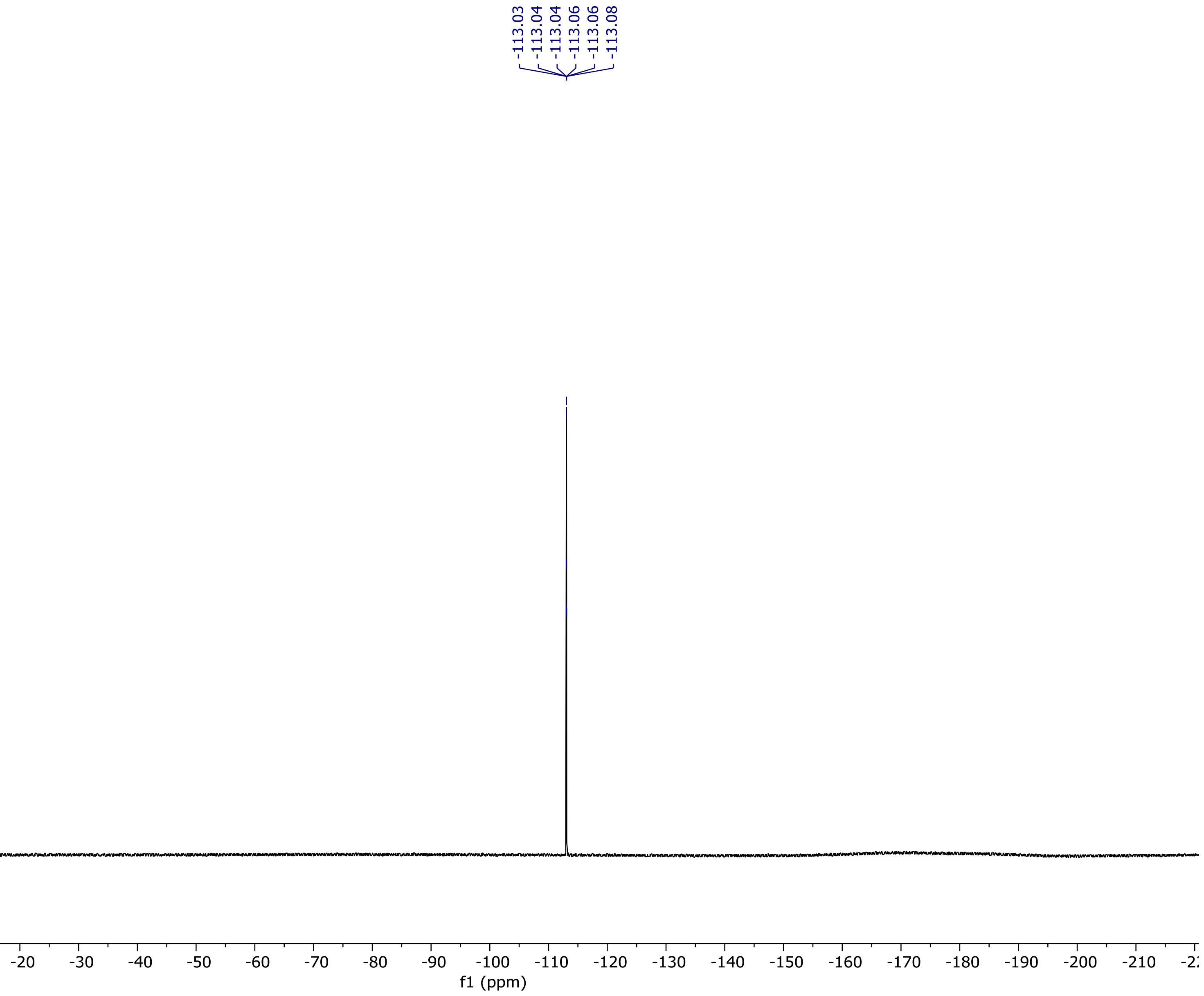

^{1}H NMR of bicyclo[1.1.1]pentylether 40

$CDCl_3$, 298 K

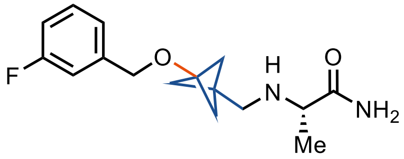

40

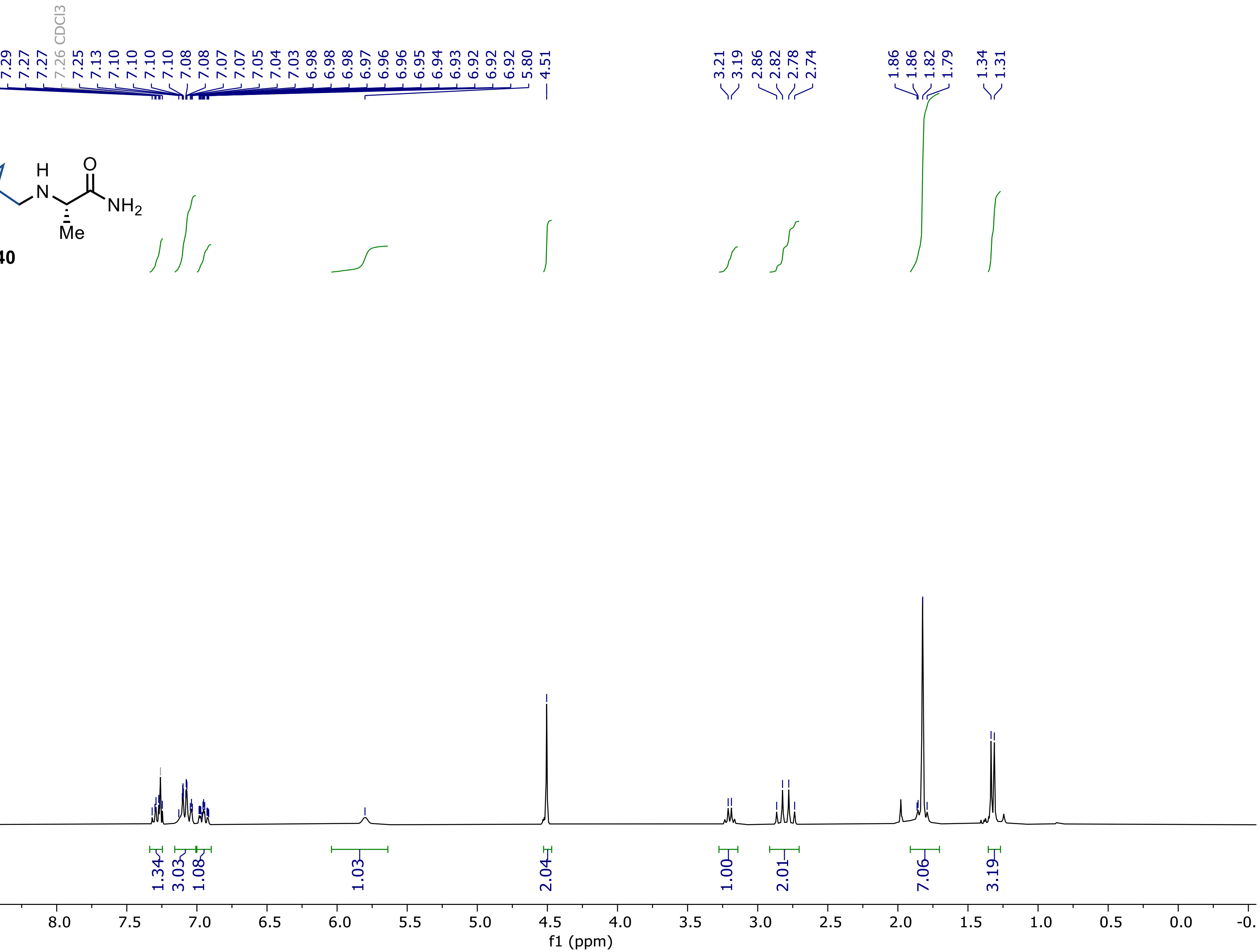

^{13}C NMR of bicyclo[1.1.1]pentylether 40

$CDCl_3$, 298 K

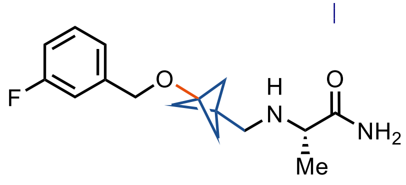

40

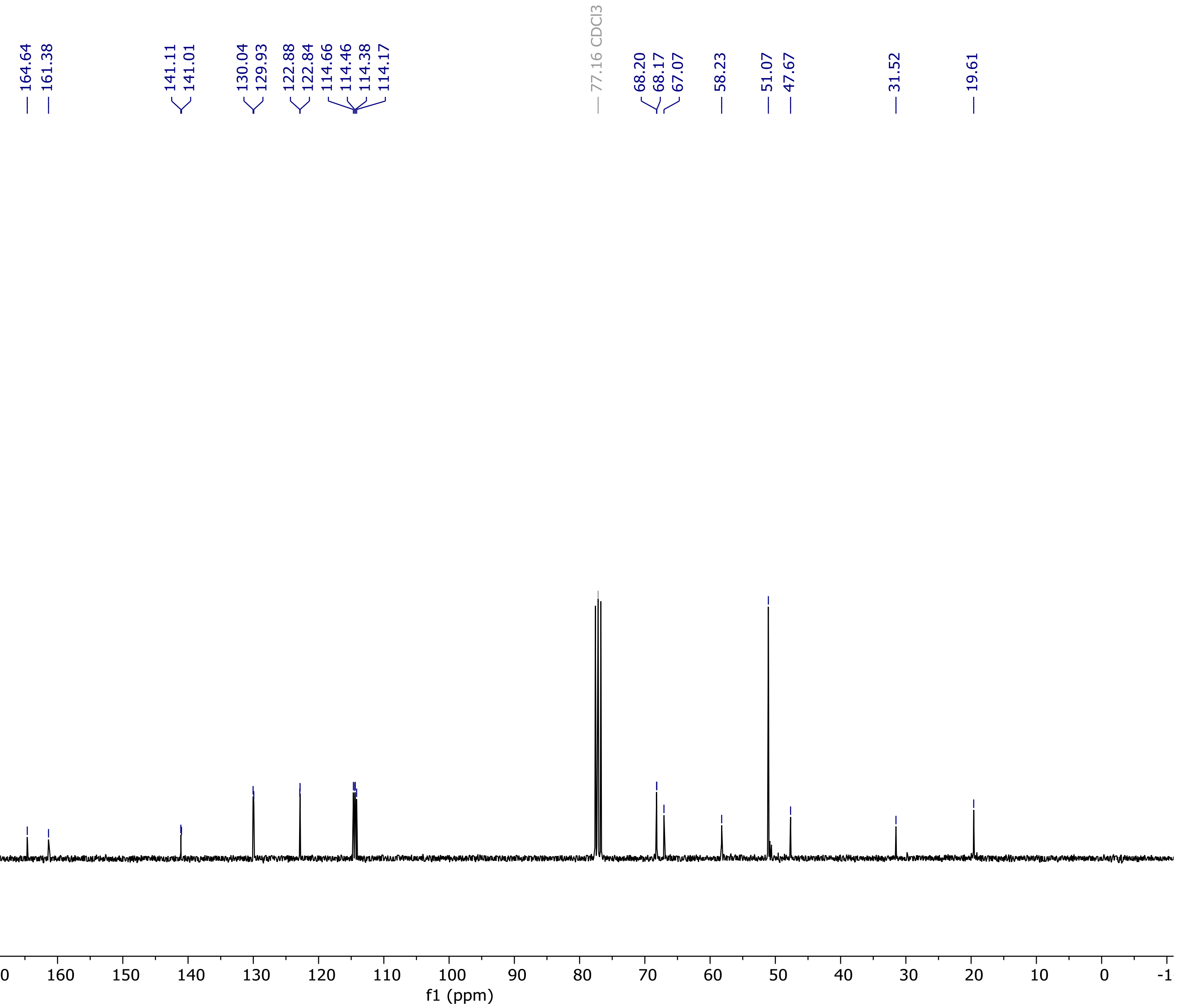

^{19}F NMR of bicyclo[1.1.1]pentylether 40

CDCl$_3$, 298 K

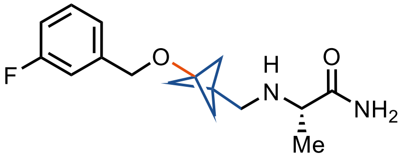

40

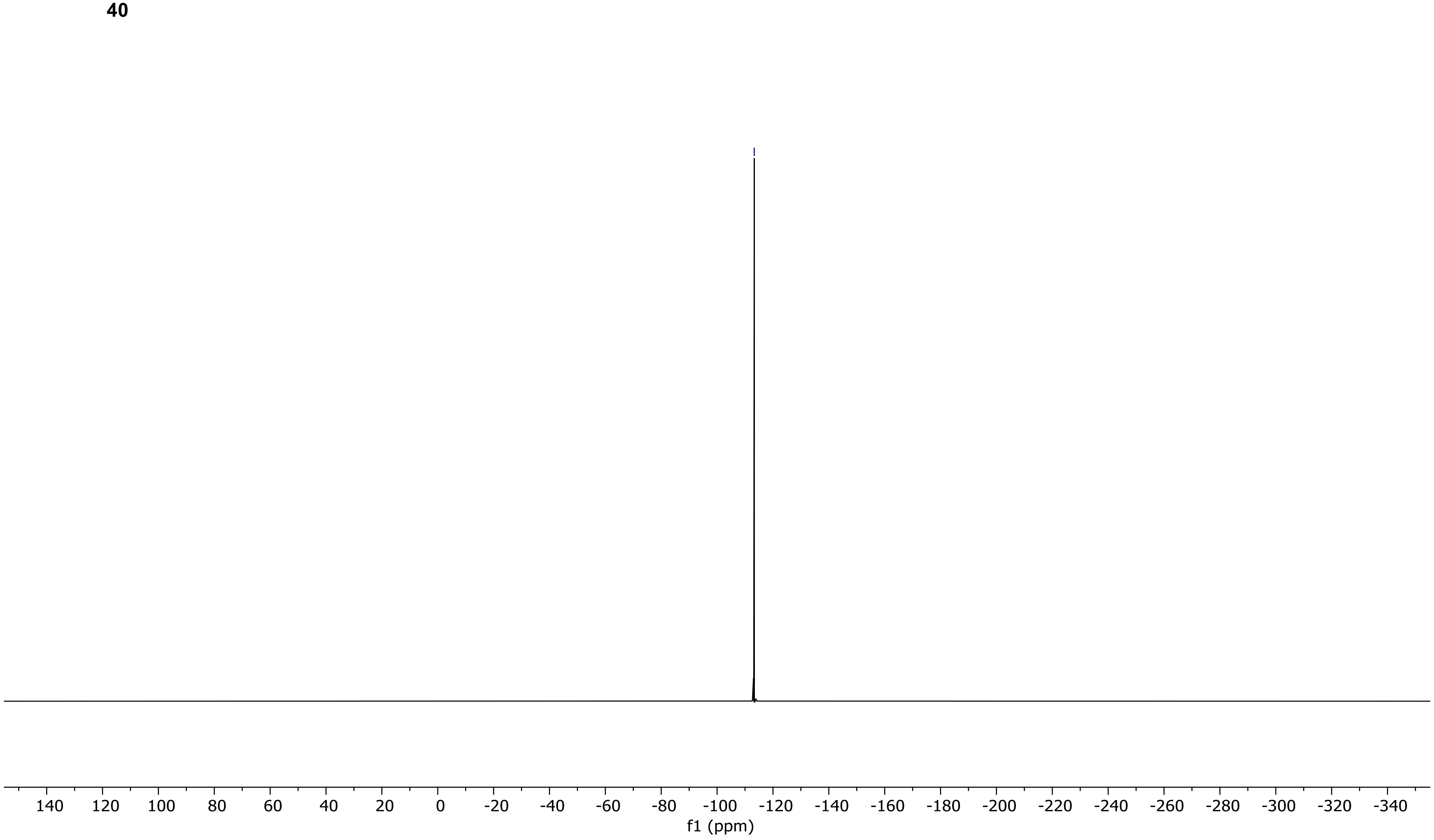

^{1}H NMR of bicyclo[1.1.1]pentylether 41

CDCl$_3$, 298 K

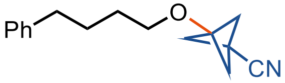

41

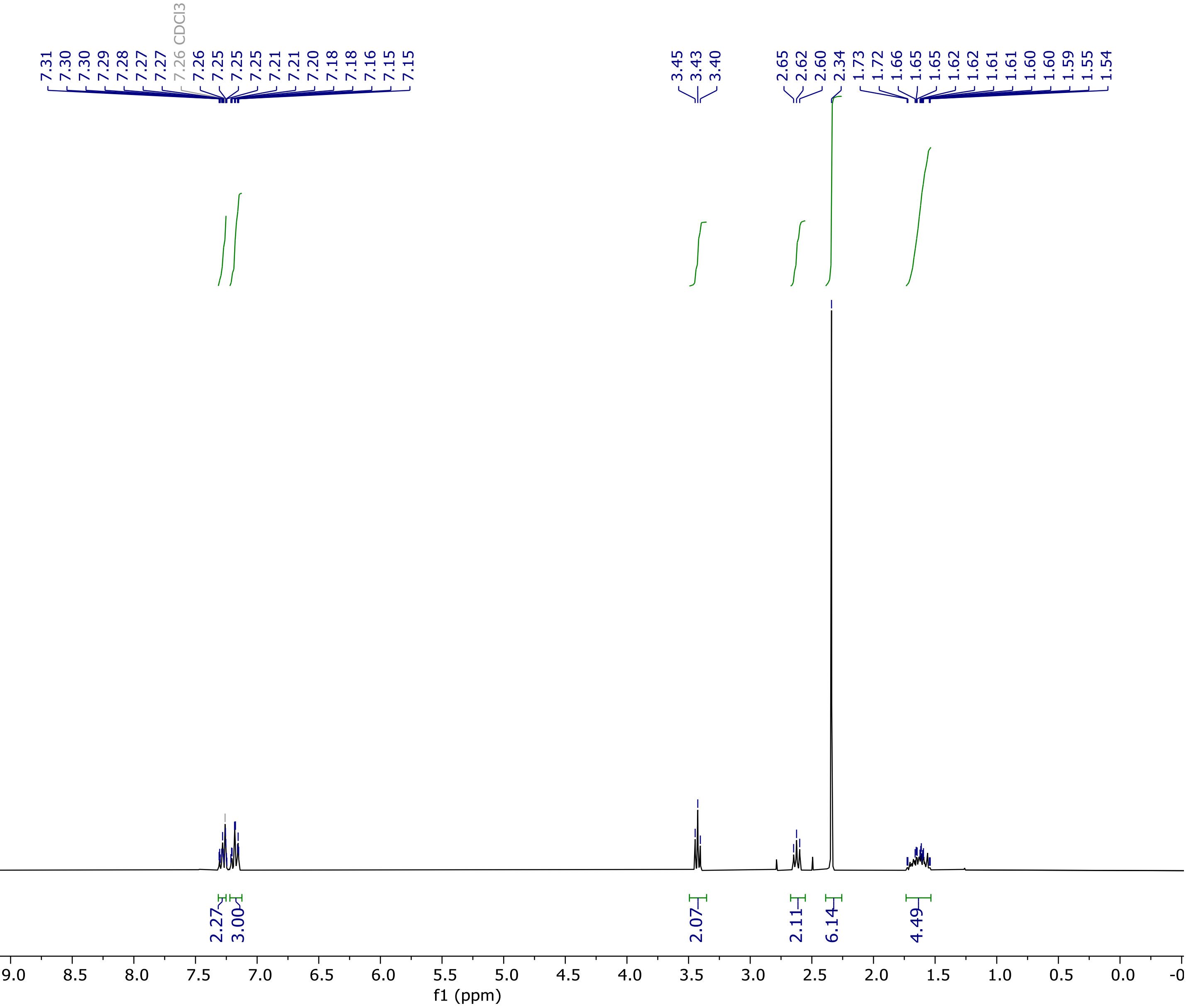

^{13}C NMR of bicyclo[1.1.1]pentylether 41

$CDCl_3$, 298 K

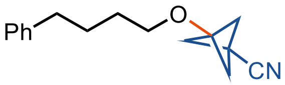

41

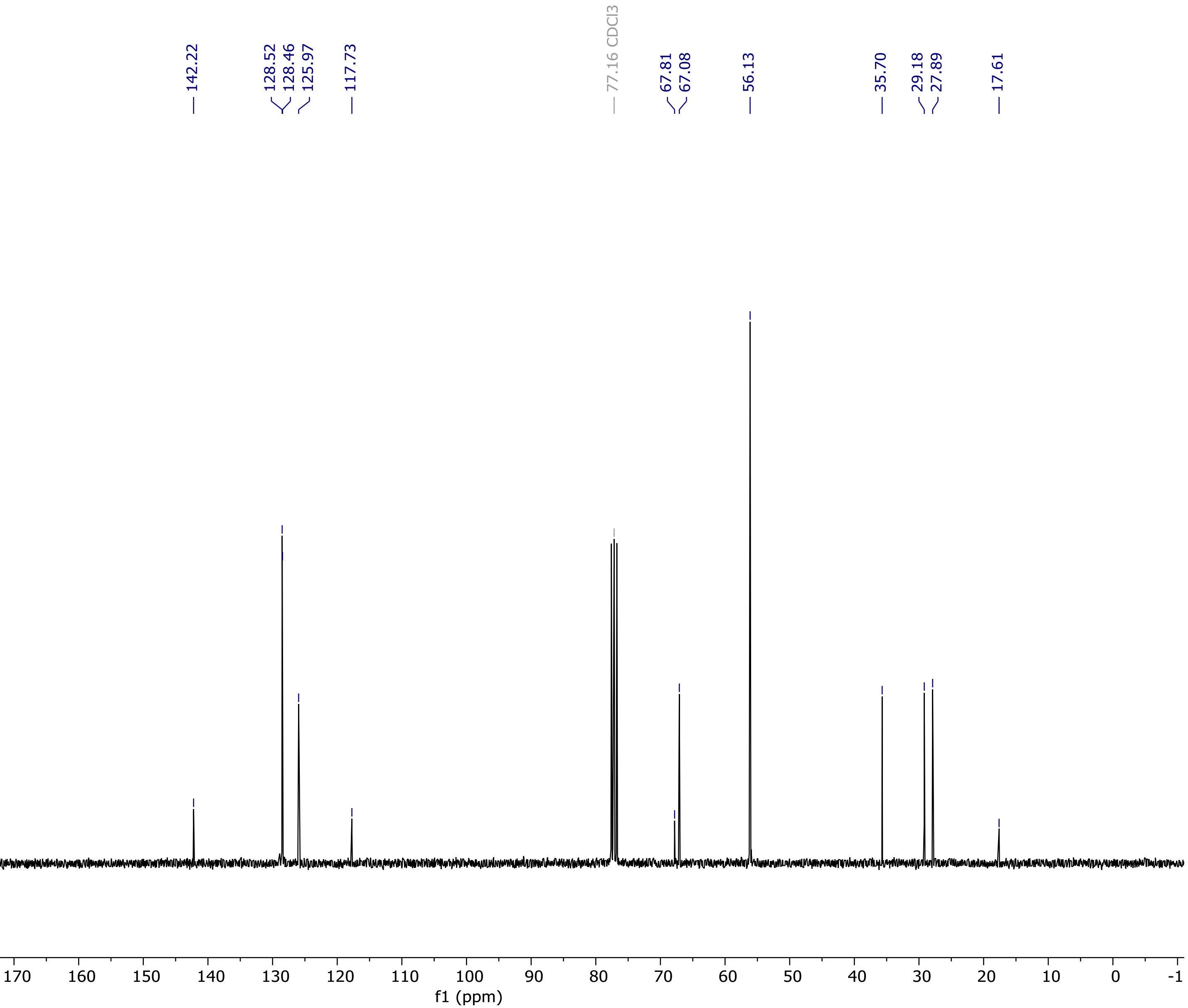

^{1}H NMR of bicyclo[1.1.1]pentylether 41-1

$CDCl_3$, 298 K

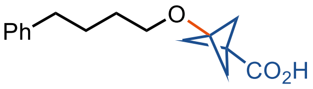

41-1

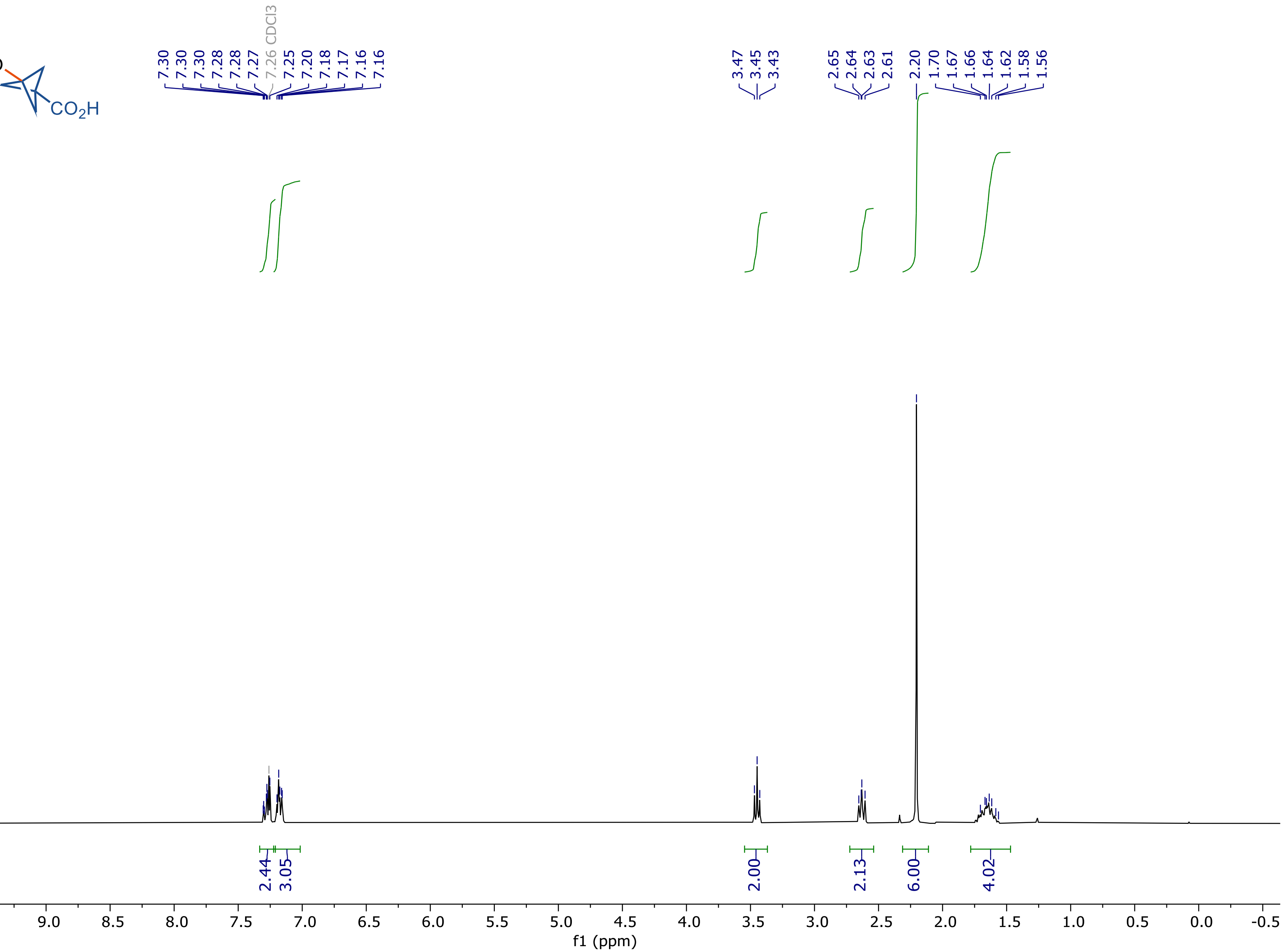

^{13}C NMR of bicyclo[1.1.1]pentylether 41-1

$CDCl_3$, 298 K

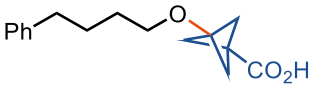

41-1

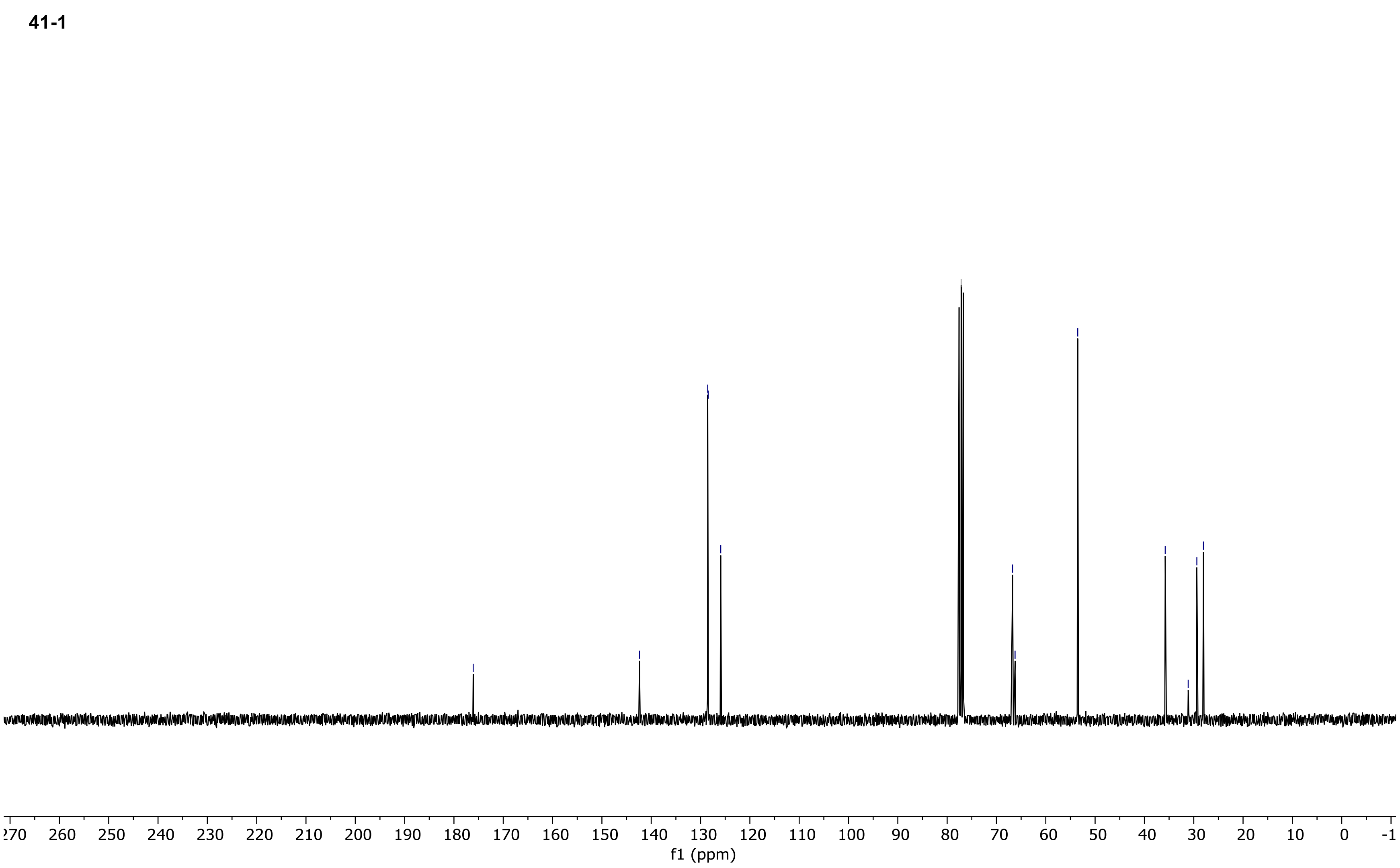

^{1}H NMR of bicyclo[1.1.1]pentylether 42

MeOH-d4, 298 K

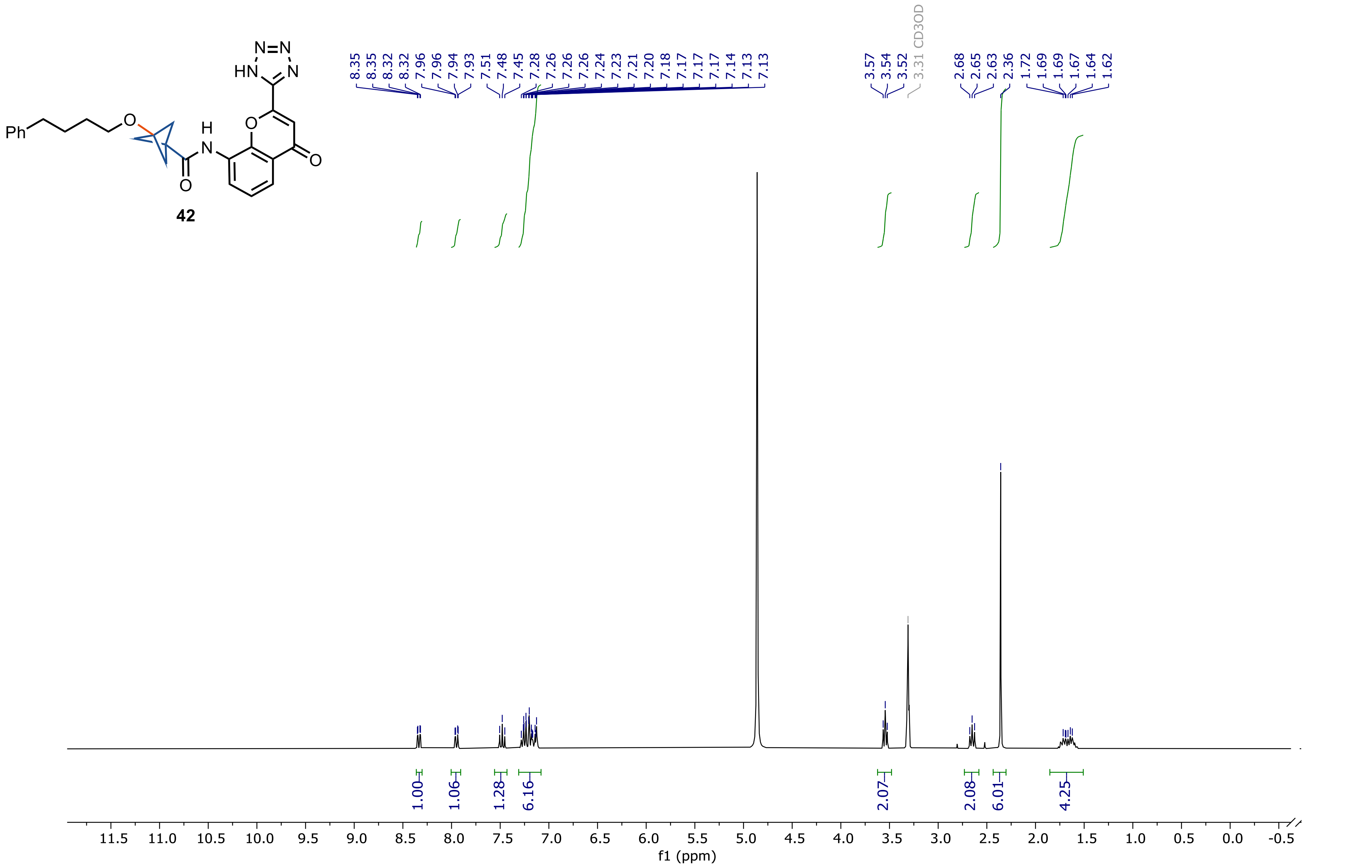

^{13}C NMR of bicyclo[1.1.1]pentylether 42

MeOH-d4, 298 K

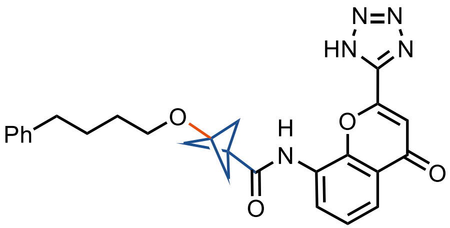

42

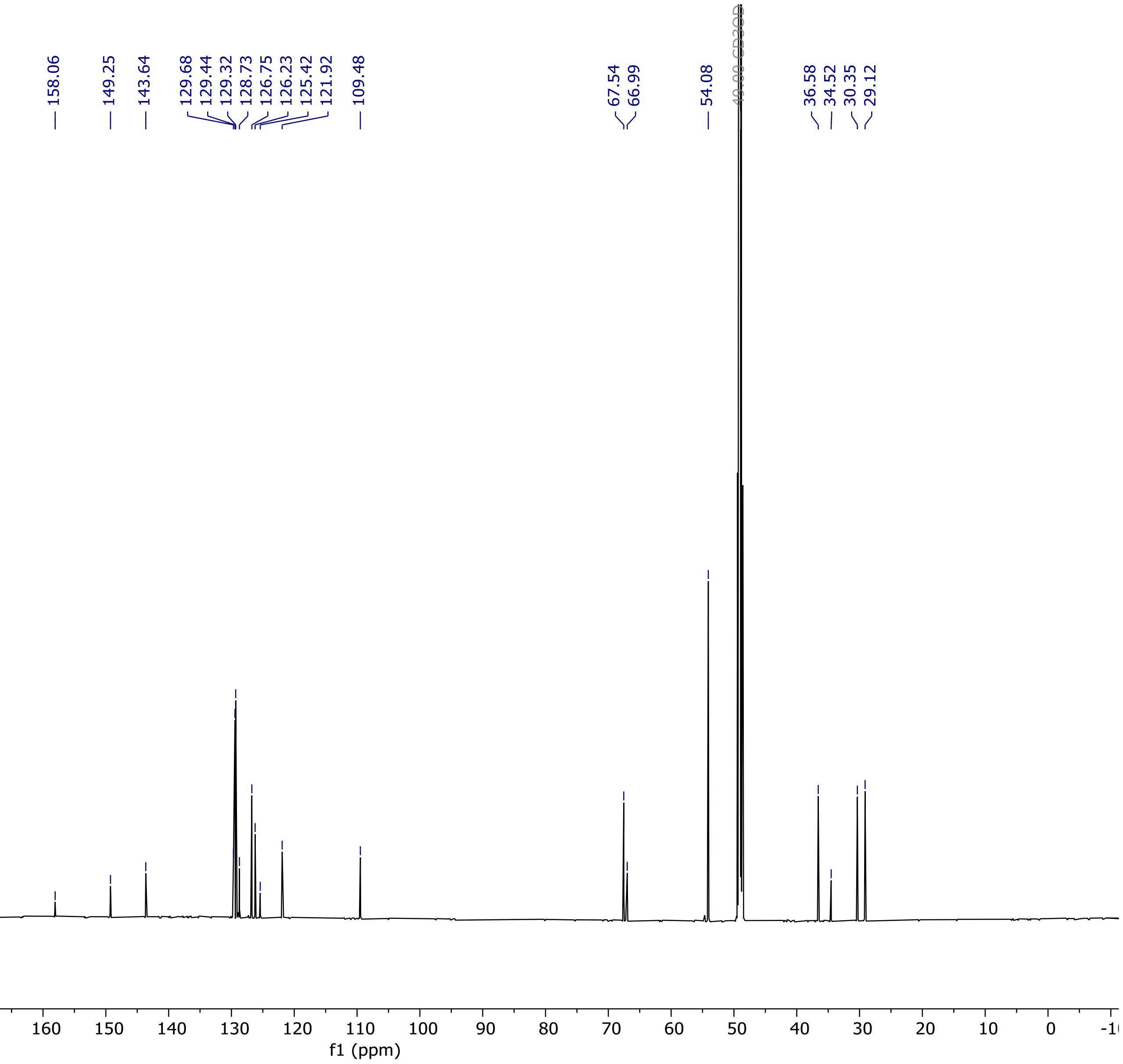

^{1}H NMR of bicyclo[1.1.1]pentylether 43

CDCl$_3$, 298 K

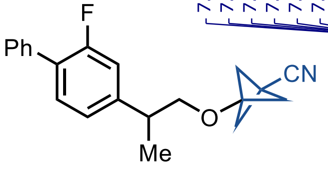

43

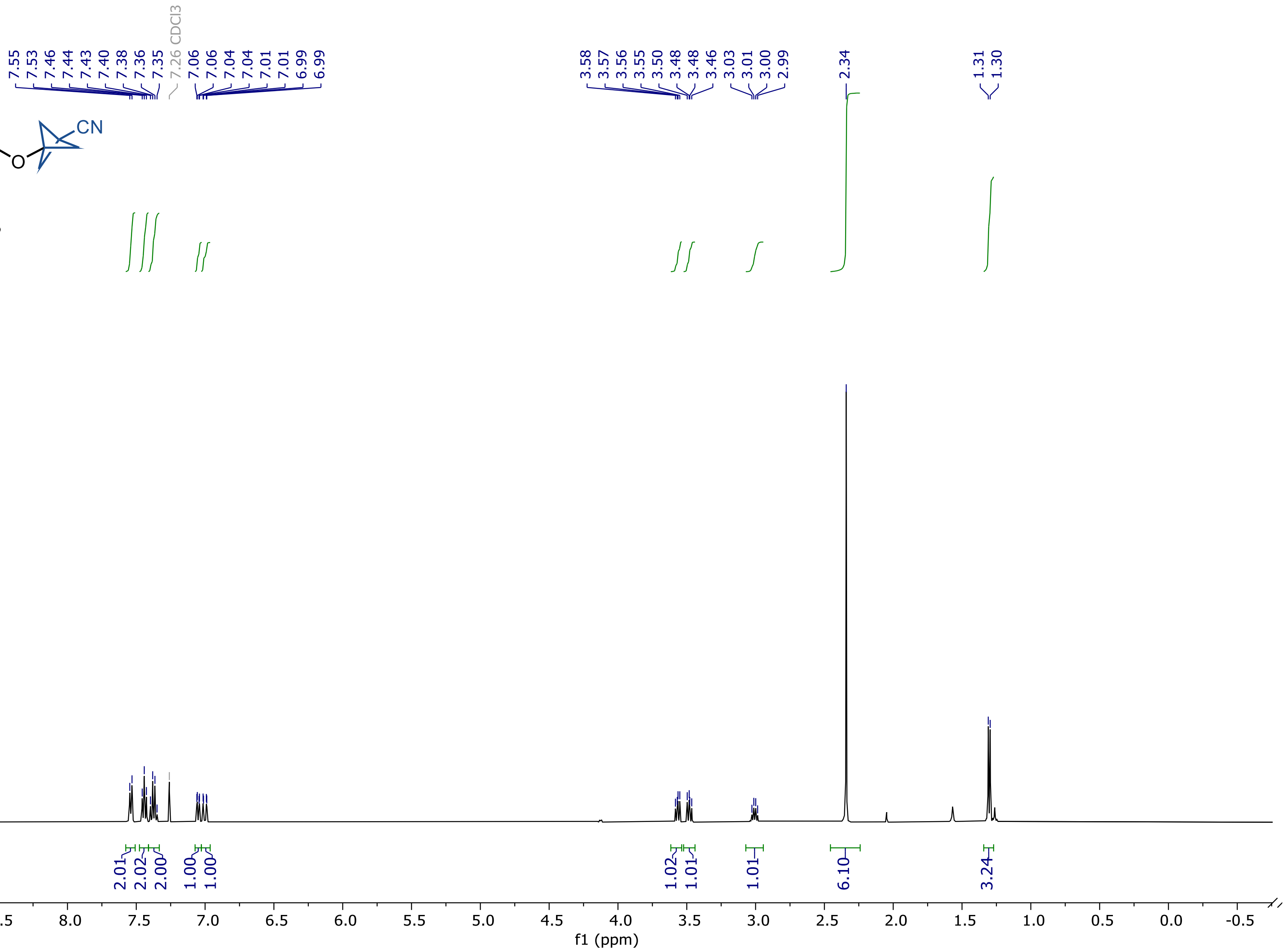

^{13}C NMR of bicyclo[1.1.1]pentylether 43

$CDCl_3$, 298 K

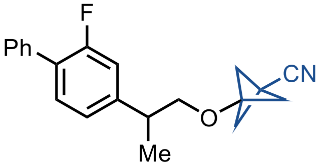

43

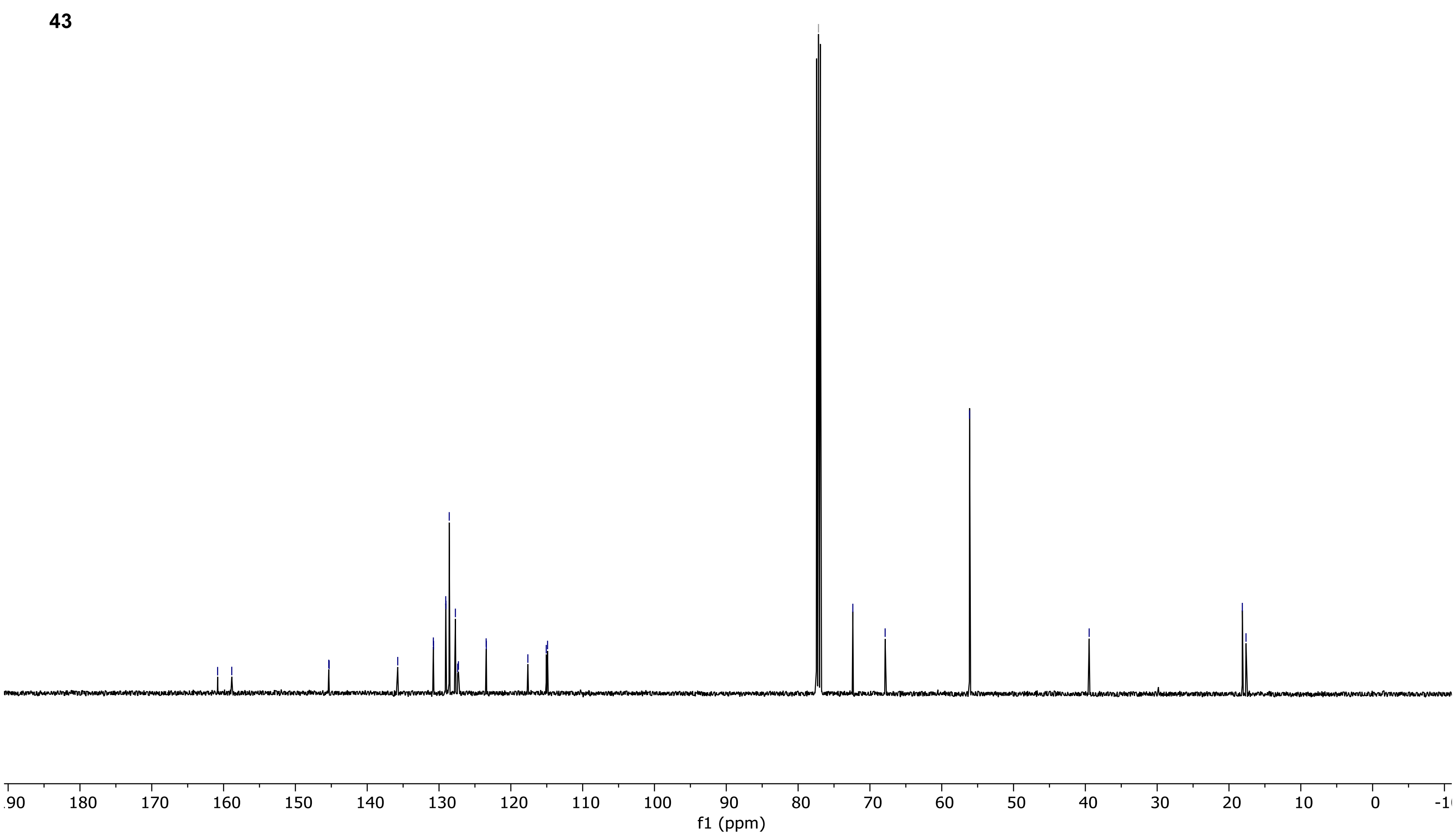

^{19}F NMR of bicyclo[1.1.1]pentylether 43

$CDCl_3$, 298 K

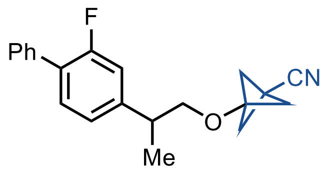

43

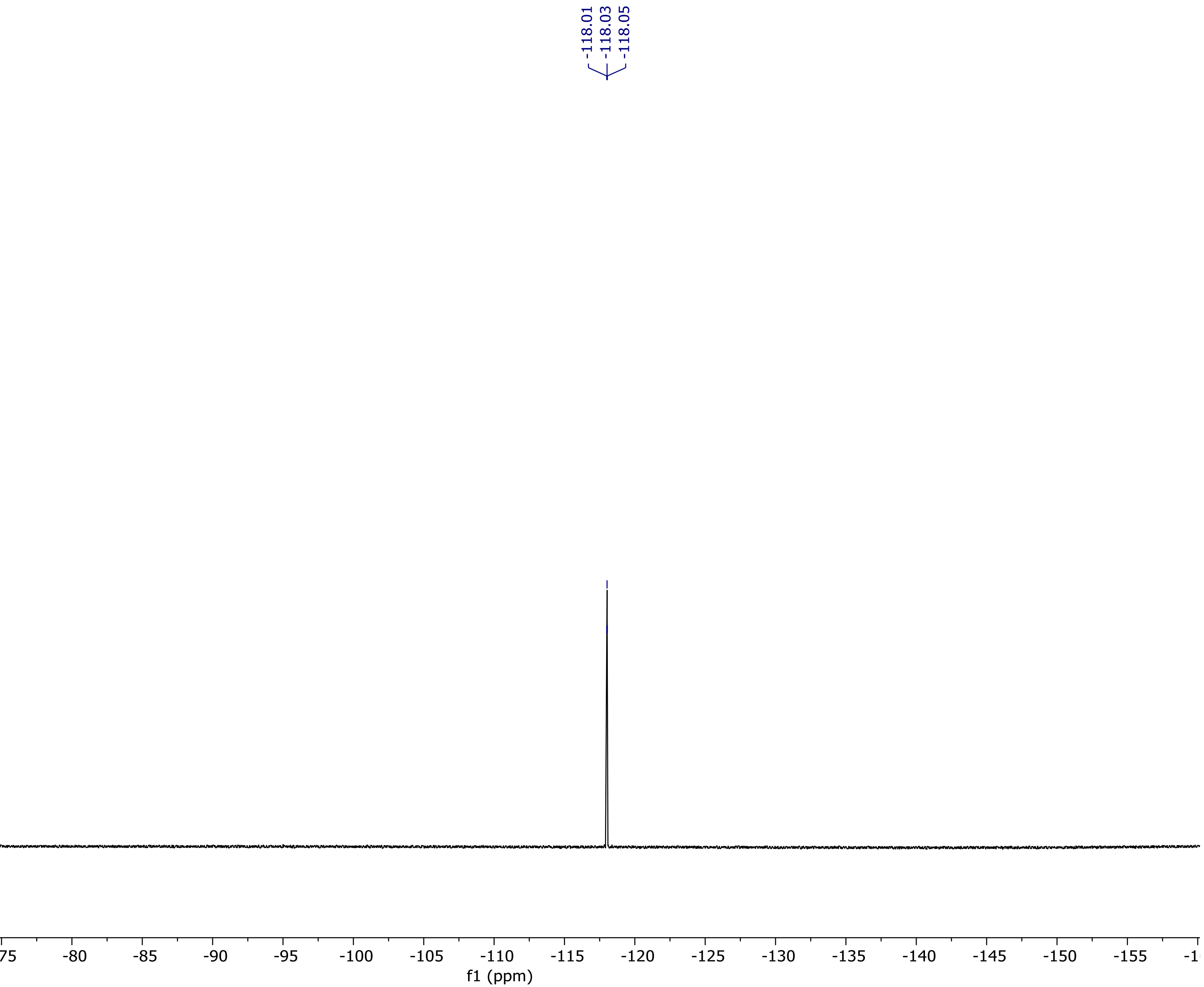

^{1}H NMR of bicyclo[1.1.1]pentylether 44

CDCl$_3$, 298 K

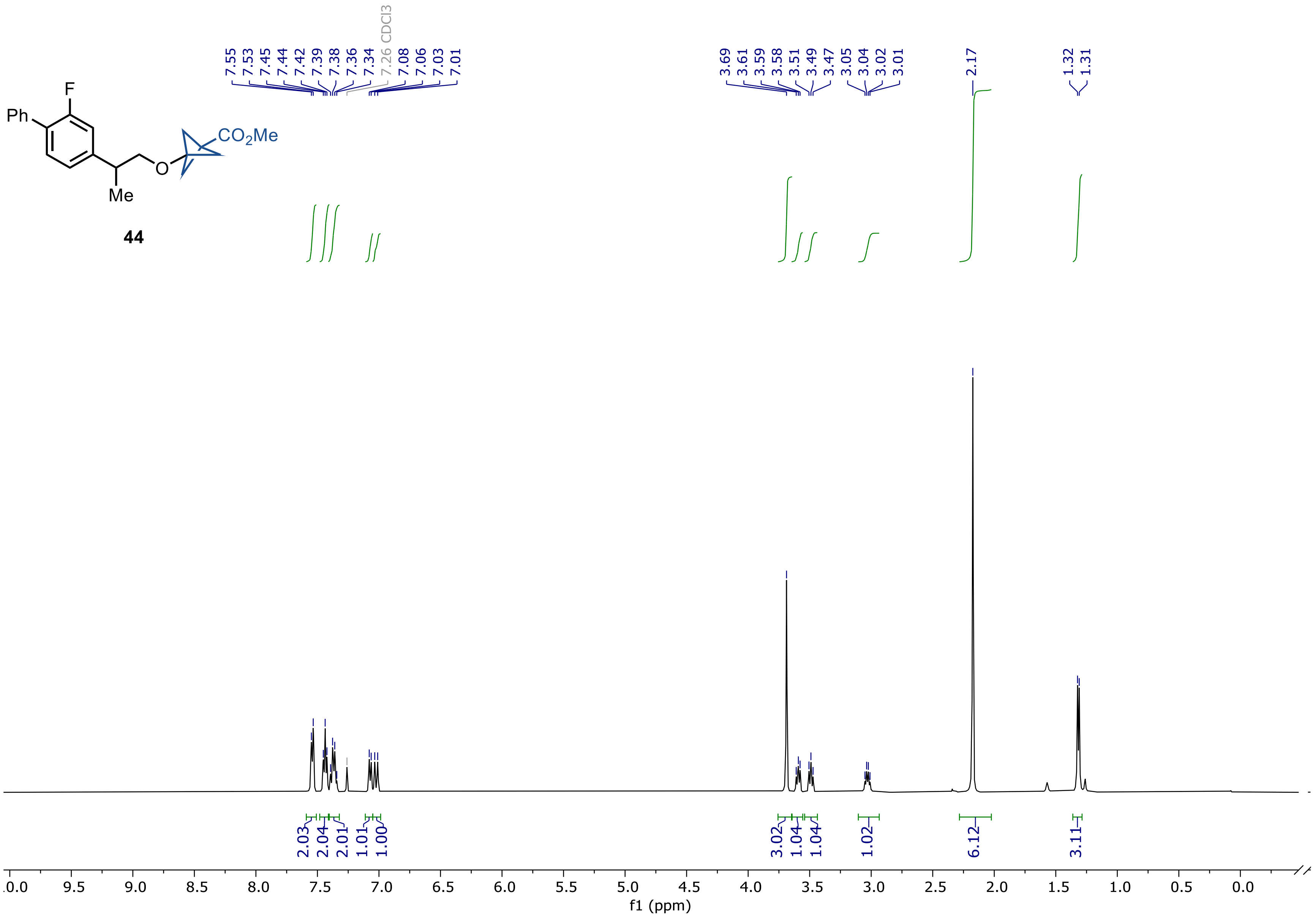

^{13}C NMR of bicyclo[1.1.1]pentylether 44

$CDCl_3$, 298 K

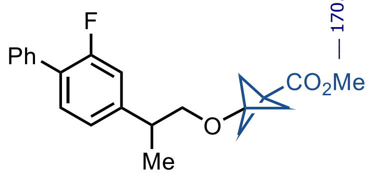

44

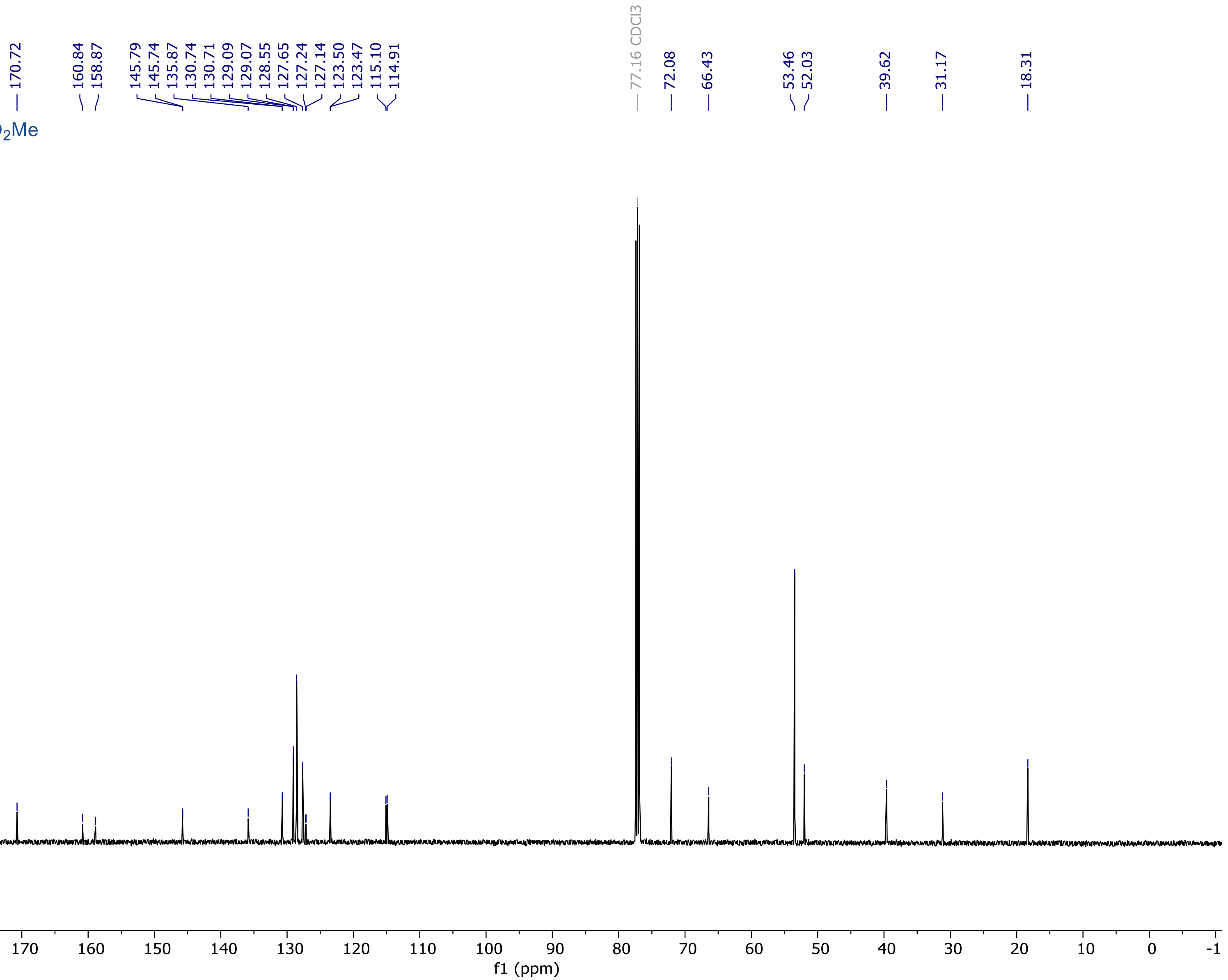

^{19}F NMR of bicyclo[1.1.1]pentylether 44

$CDCl_3$, 298 K

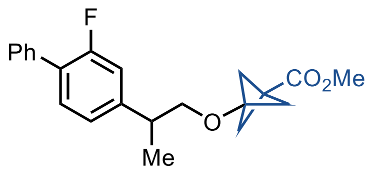

44

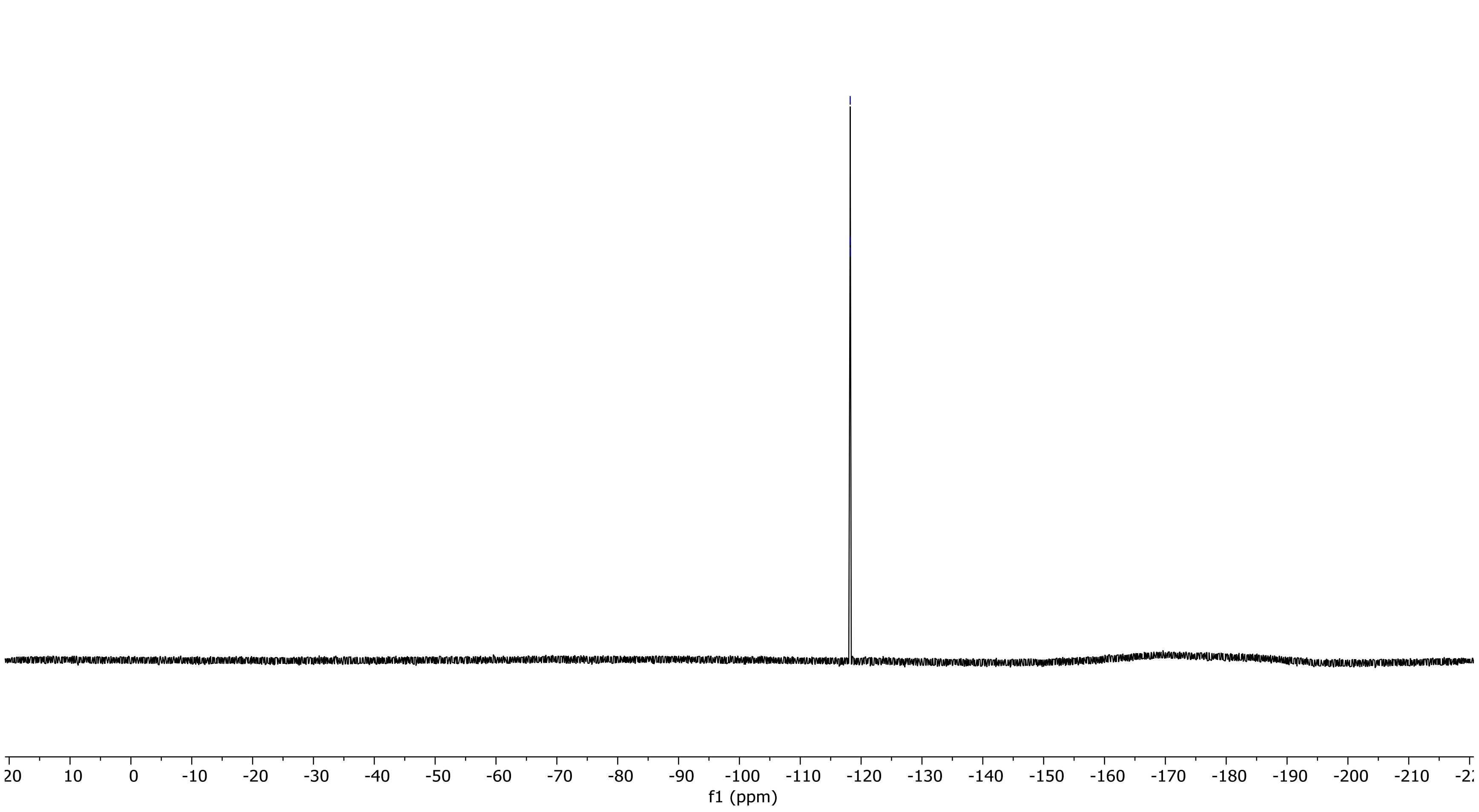

^{1}H NMR of bicyclo[1.1.1]pentylether 45

CDCl$_3$, 298 K

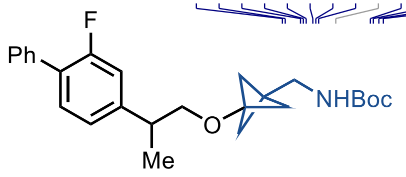

45

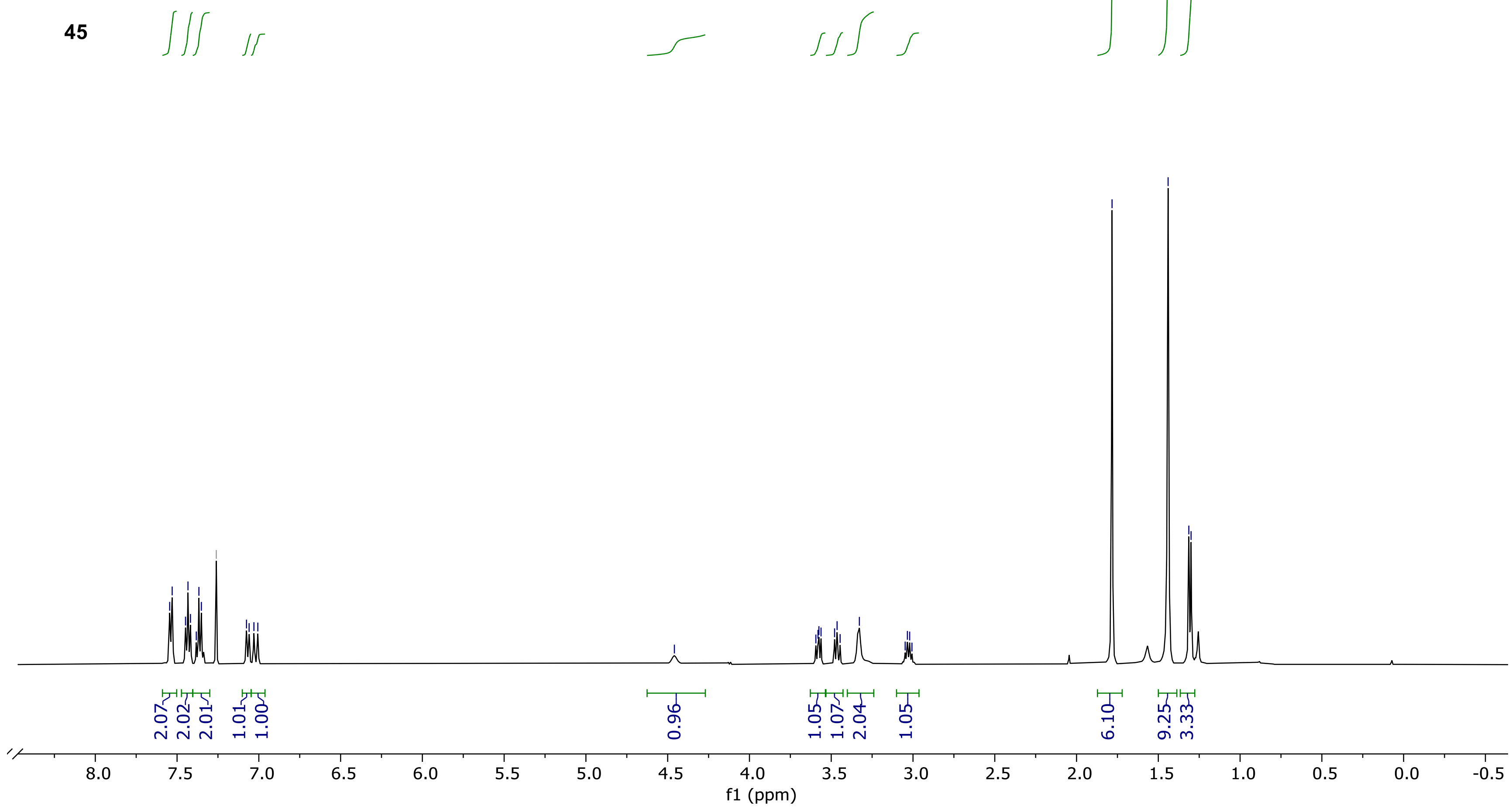

^{13}C NMR of bicyclo[1.1.1]pentylether 45

$CDCl_3$, 298 K

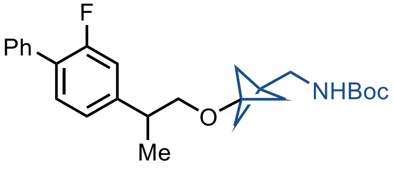

45

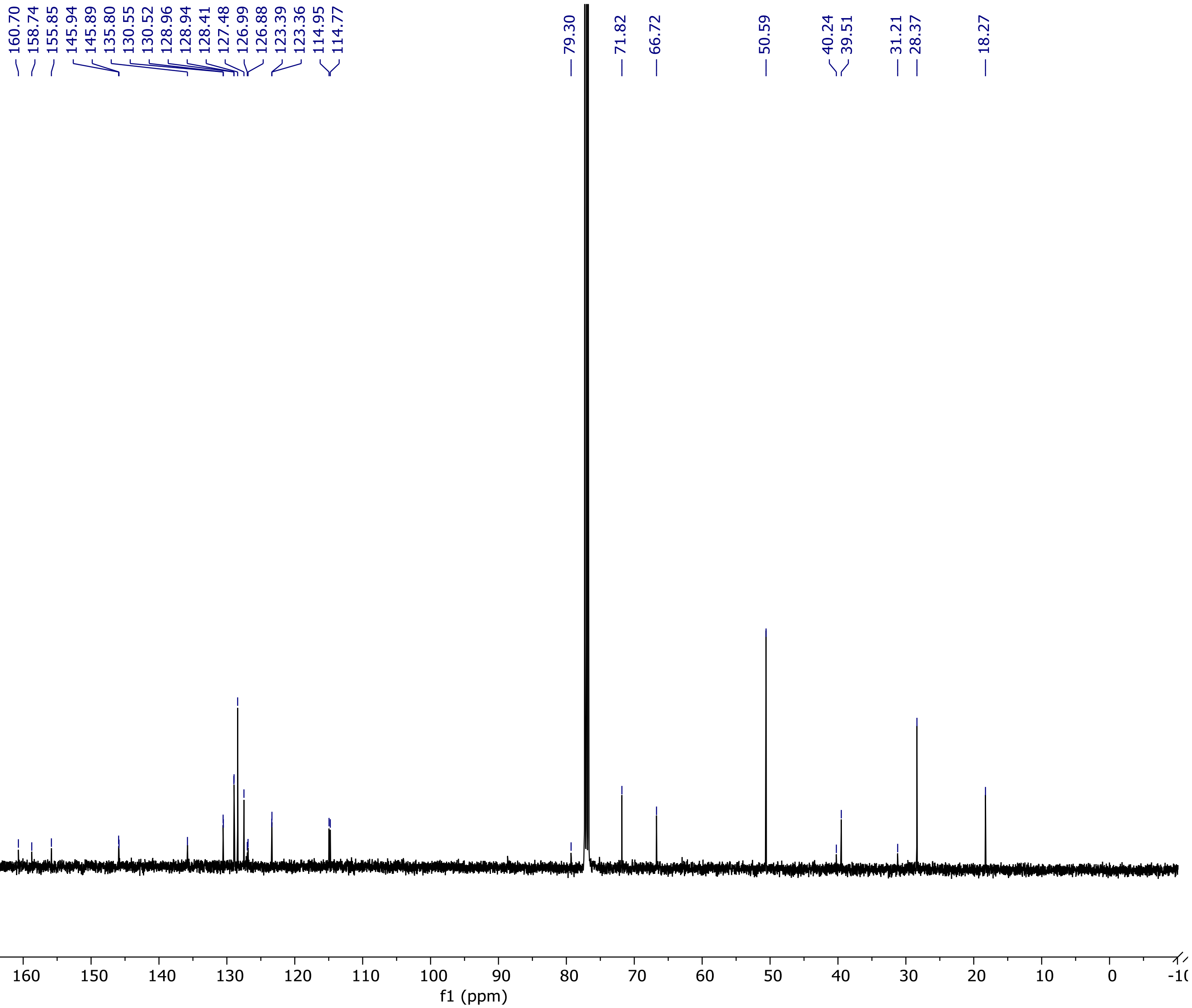

^{19}F NMR of bicyclo[1.1.1]pentylether 45

CDCl$_3$, 298 K

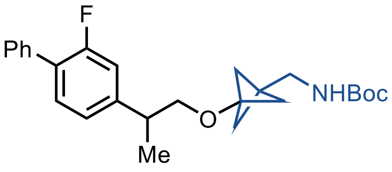

45

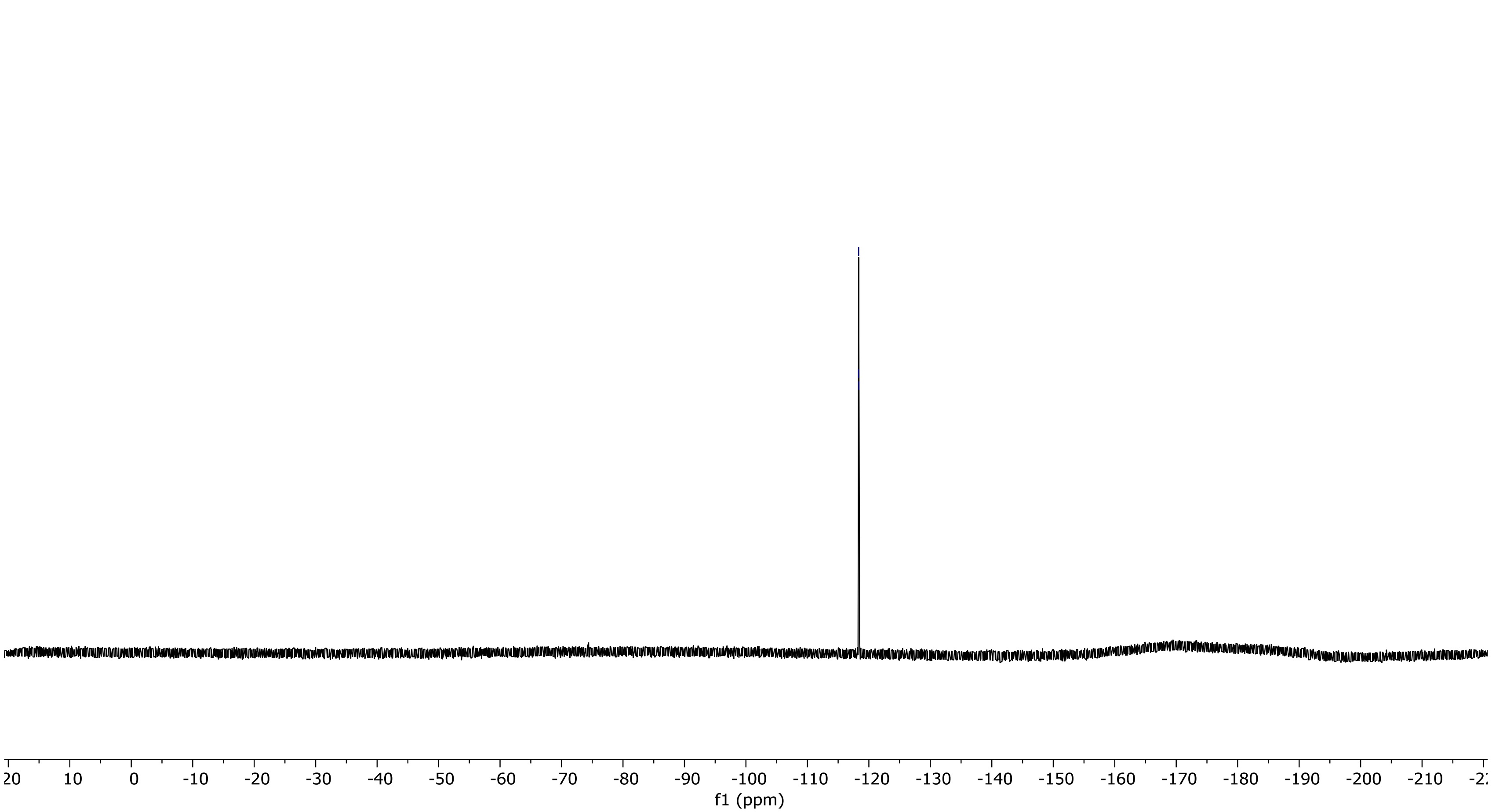

^{1}H NMR of bicyclo[1.1.1]pentylether 46

$CDCl_3$, 298 K

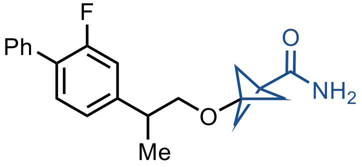

46

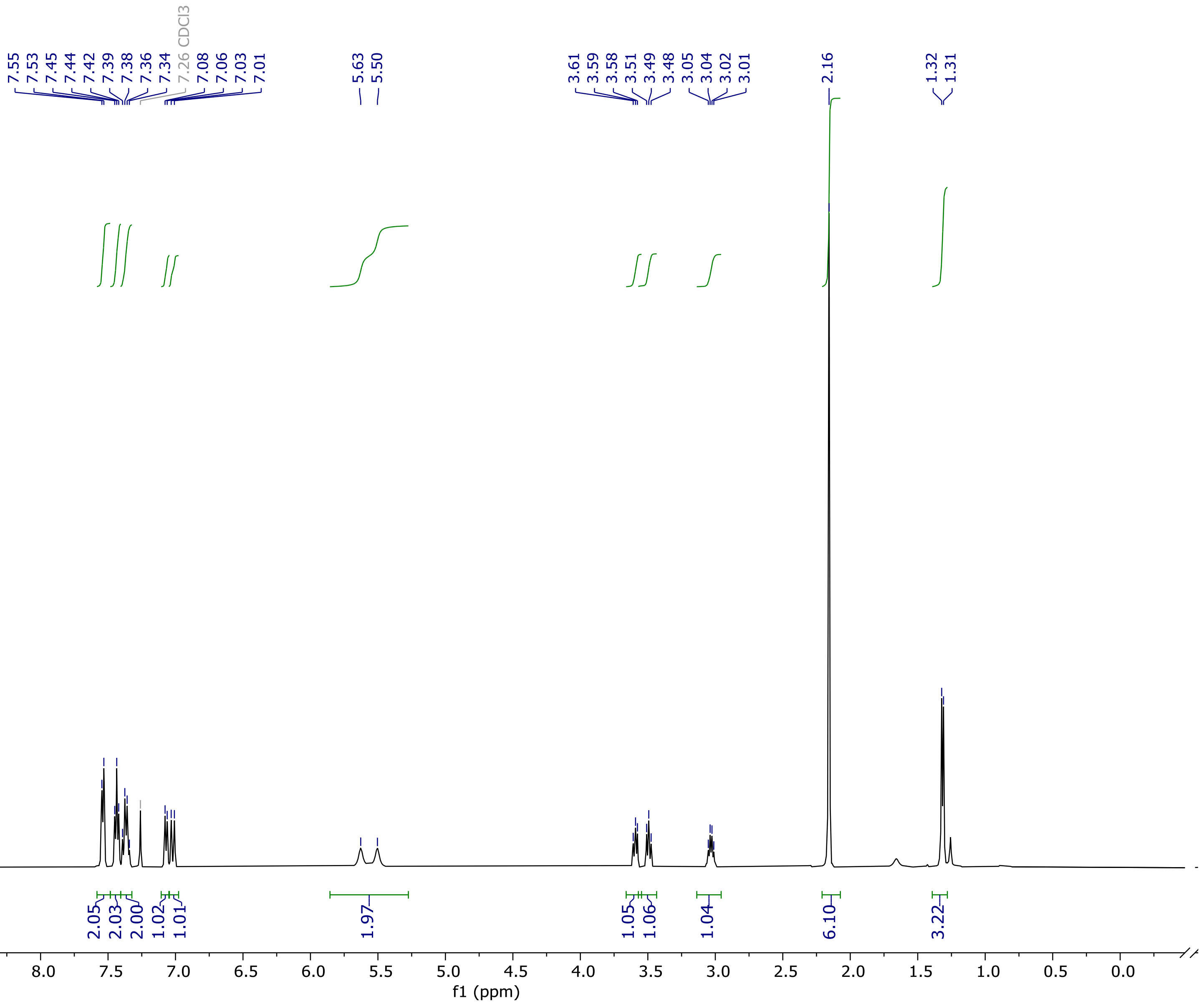

^{13}C NMR of bicyclo[1.1.1]pentylether 46

$CDCl_3$, 298 K

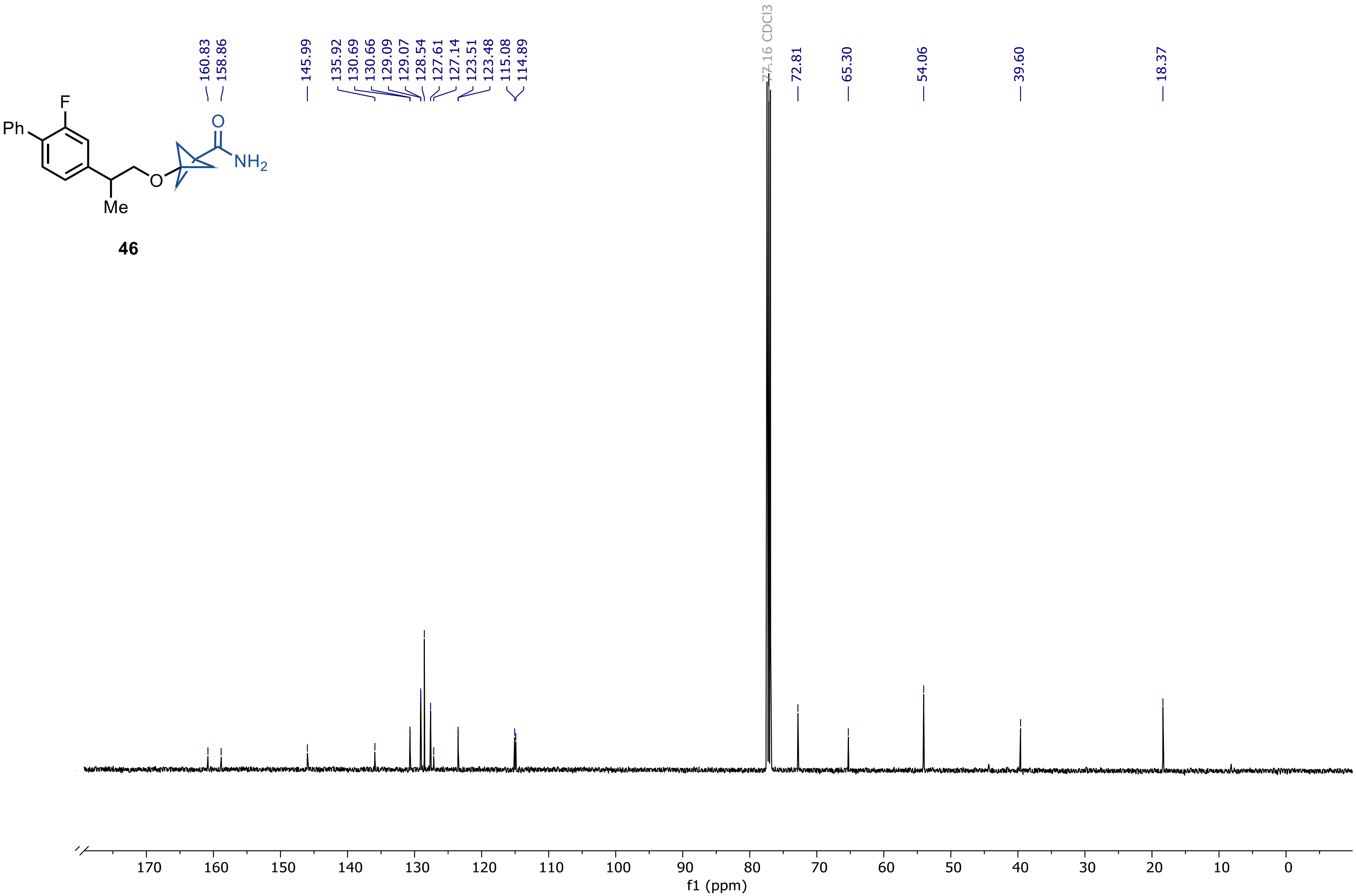

46

^{19}F NMR of bicyclo[1.1.1]pentylether 46

$CDCl_3$, 298 K

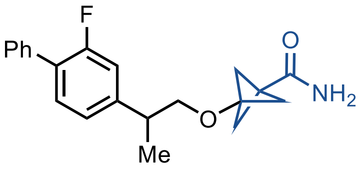

46

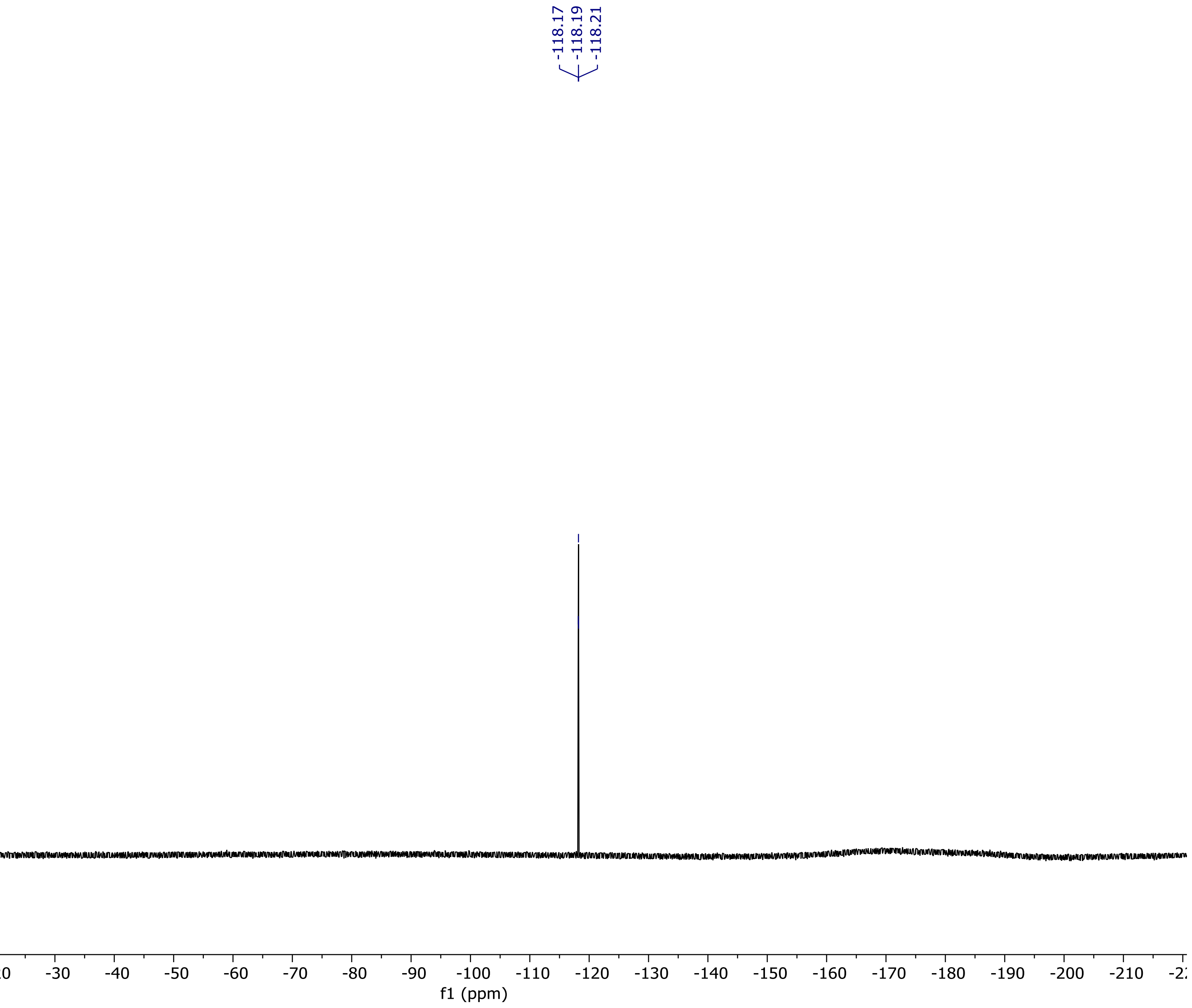

^{1}H NMR of bicyclo[1.1.1]pentylether 47

$CDCl_3$, 298 K

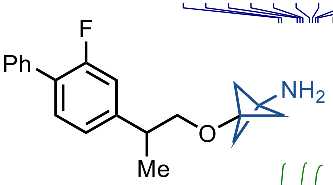

47

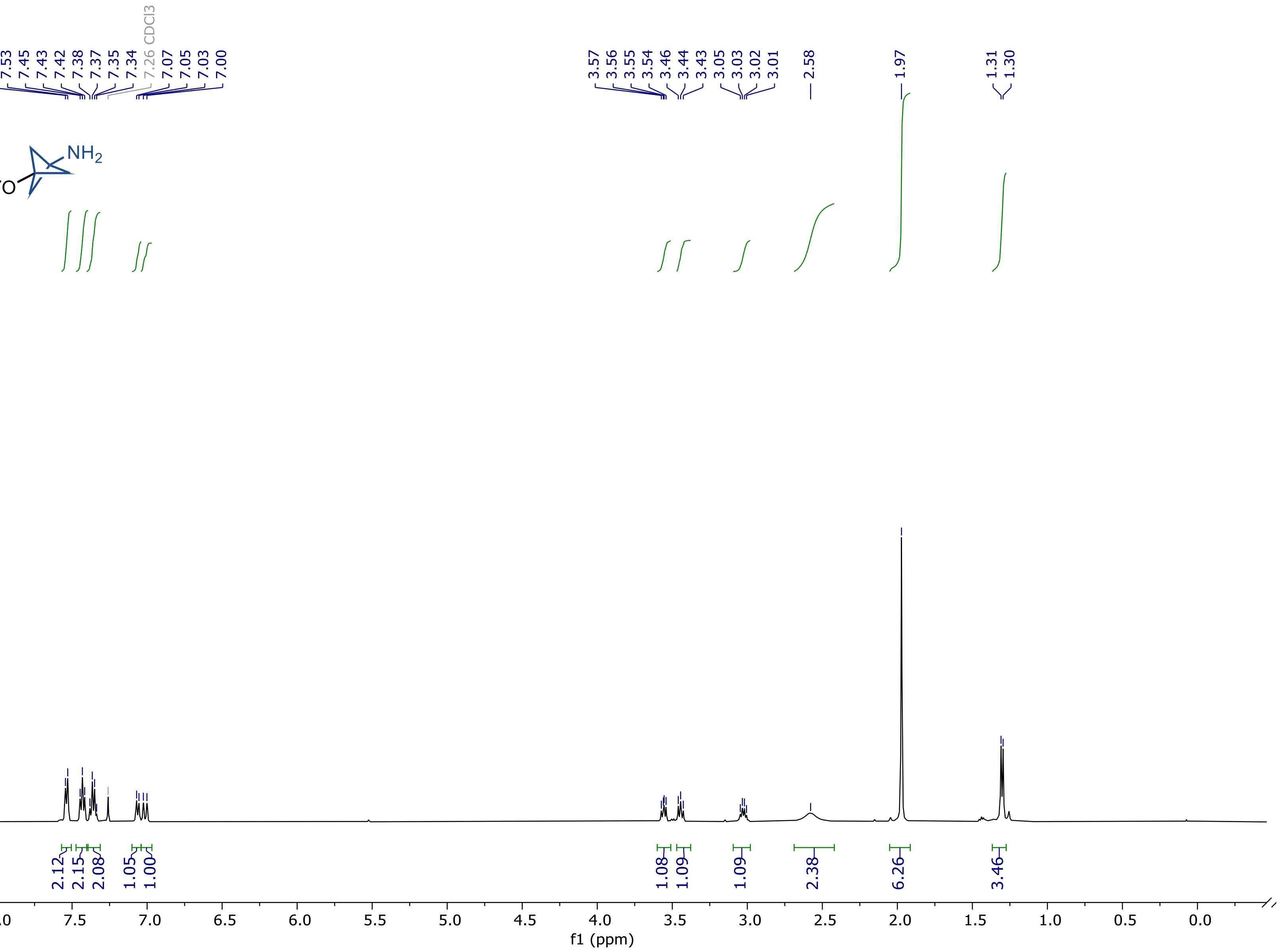

^{13}C NMR of bicyclo[1.1.1]pentylether 47

$CDCl_3$, 298 K

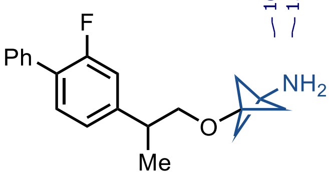

47

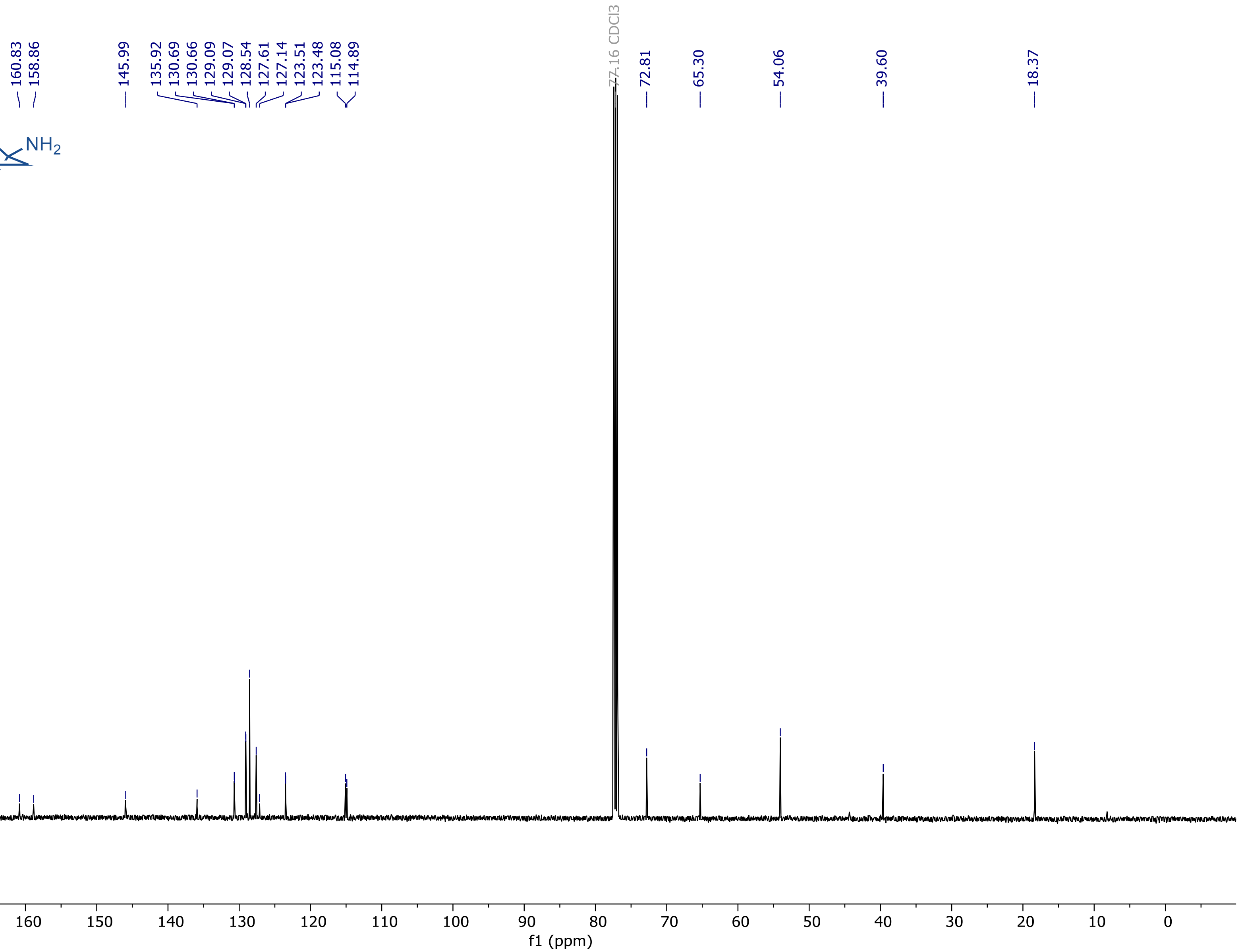

^{19}F NMR of bicyclo[1.1.1]pentylether 47

$CDCl_3$, 298 K

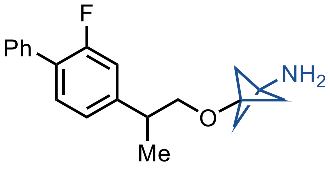

47

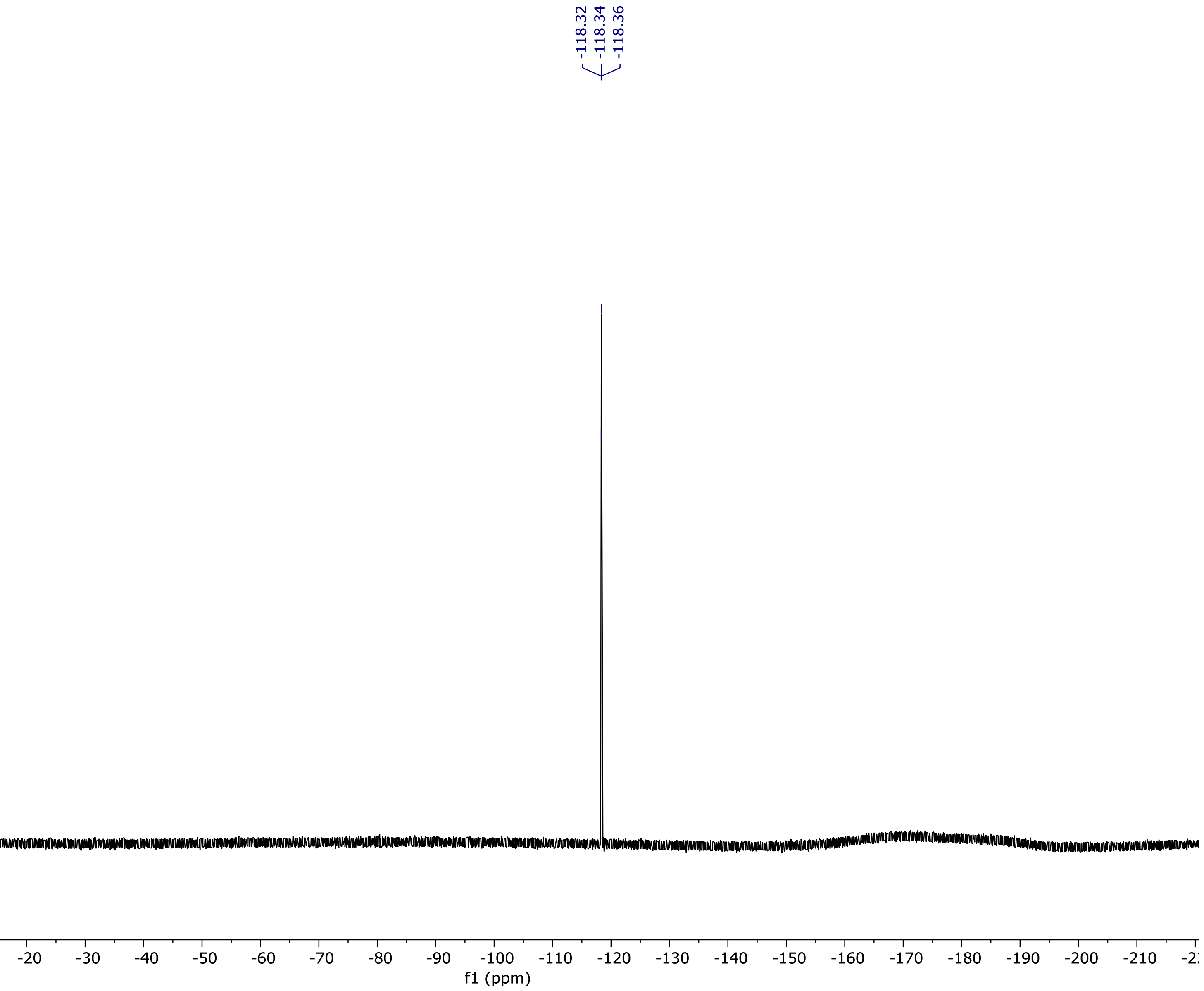

REFERENCES

1. Fulmer, G. R.; Miller, A. J. M.; Sherden N. H.; Gottlieb, H. E.; Nudelman, A.; Stoltz, B. M.; Bercaw, J. E.; Goldberg, K. I. NMR chemical shifts of trace impurities: Common laboratory solvents, organics, and gases in deuterated solvents relevant to the organometallic chemist. *Organometallics* **2010**, *29*, 2176–2179.
2. Harris, R. K.; Becker, E. D.; Cabral de Menezes, S. M.; Goodfellow, R.; Granger, P. NMR nomenclature: Nuclear spin properties and conventions for chemical shifts. IUPAC Recommendations 2001. *Solid State Nucl. Magn. Reson.* **2002**, *22*, 458–483.
3. Alvarez, E. M.; Bai, Z.; Pandit, S.; Frank, N.; Torkowski, L.; Ritter, T. *O*-, *N*- and *C*-bicyclopentylation using thianthrenium reagents. *Nat. Synth.* **2023,** *2,* 548–556.
4. Lowry, M. S.; Goldsmith, J. I.; Slinker, J. D.; Rohl, R.; Pascal, R. A., Jr.; Malliaras, G. G.; Bernhard, S. Single-Layer Electroluminescent Devices and Photoinduced Hydrogen Production from an Ionic Iridium(III) Complex. *Chem. Mater.* **2005**, *17*, 5712−5719.
5. Borissevitch, I. E. More about the inner filter effect: Corrections of Stern-Volmer fluorescence quenching constants are necessary at very low optical absorption of the quencher. *Journal of Luminescence* **1999**, *81*, 219–214.
6. Li, J.; Chen, J.; Sang, R.; Ham, W.-S.; Plutschack, M. B.; Berger, F.; Chabbra, S.; Schnegg, A.; Genicot, C.; Ritter, T. Photoredox catalysis with aryl sulfonium salts enables site-selective late-stage fluorination. *Nat. Chem.* **2020**, *12*, 56–62.
7. Neese, F. WIREs The ORCA program system. *Comput. Mol. Sci.* **2012**, *2*, 73–78.
8. Lee, C.; Yang, W.; Parr, R. G. Development of the Colle-Salvetti correlation-energy formula into a functional of the electron density. *Phys. Rev. B.* **1988**, *37*, 785–789.
9. Grimme, S.; Antony, J.; Ehrlich, S.; Krieg, H. A consistent and accurate ab initio parametrization of density functional dispersion correction (DFT-D) for the 94 elements H-Pu. *J. Chem. Phys.* **2010**, *132*, 154104.
10. Grimme, S.; Ehrlich, S.; Goerigk, L. Effect of the damping function in dispersion corrected density functional theory. *J. Comput. Chem.* **2011**, *32*, 1456–1465.
11. Weigend, F. Accurate Coulomb-fitting basis sets for H to Rn. *Phys. Chem. Chem. Phys.* **2006**, *8*, 1057–1065.
12. Weigend, F.; Ahlrichs, R. Balanced basis sets of split valence, triple zeta valence and quadruple zeta valence quality for H to Rn: Design and assessment of accuracy. *Phys. Chem. Chem. Phys.* **2005**, *7*, 3297–3305.
13. Barone, V.; Cossi, M. Quantum Calculation of Molecular Energies and Energy Gradients in Solution by a Conductor Solvent Model. *J. Phys. Chem. A* **1998**, *102*, 1995–2001.
14. Hanwell, M. D.; Curtis, D. E.; Lonie, D. C.; Vandermeersch, T.; Zurek, E.; Hutchison, G. R. Avogadro: an advanced semantic chemical editor, visualization, and analysis platform. *J. Cheminf.* **2012**, *4*, 1–17.
15. Zhurko, G. A.; Zhurko, D. A. ChemCraft: Tool for treatment of chemical data, Lite version build 08 (freeware). **2005**, 1.
